# Discovery of Natural Resorcylic Acid Lactones as Novel Potent Copper Ionophores Covalently Targeting PRDX1 to Induce Cuproptosis for Triple-Negative Breast Cancer Therapy

**DOI:** 10.1021/acscentsci.4c02188

**Published:** 2025-02-10

**Authors:** Li Feng, Ti-Zhi Wu, Xin-Rui Guo, Yun-Jie Wang, Xin-Jia Wang, Shao-Xuan Liu, Rui Zhang, Yi Ma, Ning-Hua Tan, Jin-Lei Bian, Zhe Wang

**Affiliations:** † State Key Laboratory of Natural Medicines, School of Traditional Chinese Pharmacy, China Pharmaceutical University, Nanjing 211198, China; ‡ State Key Laboratory of Natural Medicines, School of Pharmacy, China Pharmaceutical University, Nanjing 211198, China; § State Key Laboratory of Natural Medicines, School of Engineering, China Pharmaceutical University, Nanjing 211198, China

## Abstract

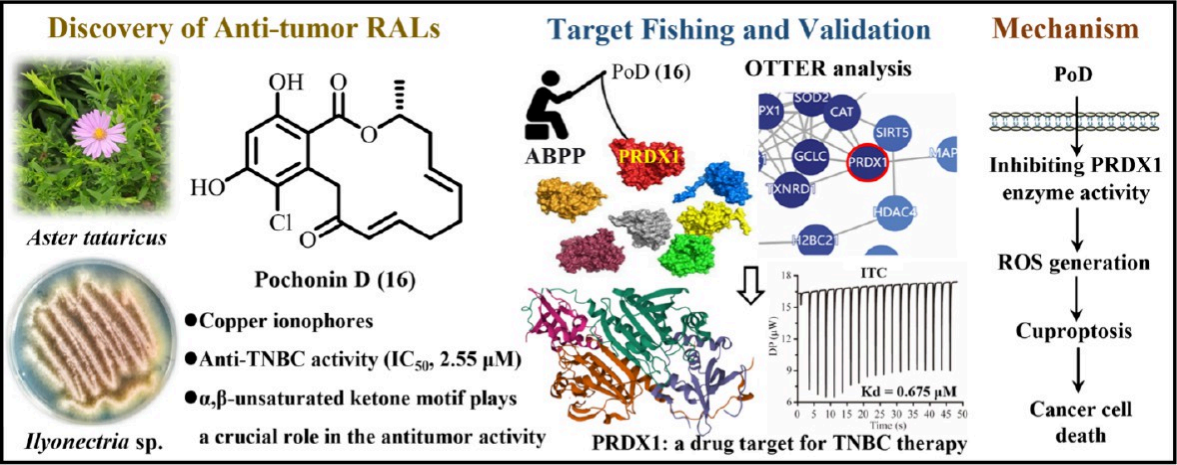

Triple-negative breast cancer (TNBC) is a highly aggressive subtype of breast cancer. Cuproptosis, a novel identified cell death form, is triggered by the direct binding of copper to lipoylated components of the tricarboxylic acid cycle. Identifying new effective drug targets and copper ionophores inducing cuproptosis for TNBC therapy is an urgent clinical need. In this study, a total of 24 resorcylic acid lactones (RALs, **1**–**24**), including 9 previously unreported ones, were isolated from the endophyte *Ilyonectria* sp. Various assays demonstrated that pochonin D (**16**, PoD) effectively inhibited the proliferation of TNBC cells *in vivo* and *in vitro*. Further investigations, including transcriptomics, proteomics, bioinformatics analysis, CMap, OTTER, clinical samples, and the use of PoD as molecular probe, revealed that PRDX1 is associated with cuproptosis and served as a potential target in TNBC. Mechanistically, PRDX1 was involved in the process of cuproptosis, and PoD bound to the Cys173 site of PRDX1, inhibited its enzymatic activity, and intervened with cuproptosis, thereby exerting anti-TNBC activity. Our study revealed that PRDX1 is not only a promising biomarker associated with cuproptosis but also a therapeutic target for TNBC, and PoD is a novel copper ionophore capable of inducing cuproptosis in TNBC cells by targeting PRDX1.

## Abstract 2

24 RALs were identified from *Ilyonectria* sp., in which PoD (**16**) exhibited potent anti-TNBC activity. PoD was used as probe to identify PRDX1 as a drug target involved in cuproptosis for TNBC therapy.

## Introduction

Breast cancer (BC) remains a significant global health challenge and a leading cause of morbidity among women.^1^ Triple-negative breast cancer (TNBC) is a highly aggressive BC subtype, characterized by the absence of estrogen receptor (ER), progesterone receptor (PR), and human epidermal growth factor receptor 2 (HER2) expression.^2^ As a specific subtype of BC, TNBC exhibits aggressive clinical behavior, with approximately 45% of patients experiencing distant metastases and significantly reduced survival. While molecular targeted therapy is currently the primary treatment for BC, it is usually ineffective for TNBC due to the lack of ER, PR, and HER2 expression,^3^ highlighting an urgent clinical need to identify new therapeutic targets and develop effective drugs.

Cuproptosis is a novel identified cell death form and the target for cancer treatment, whose concept was proposed for the first time in 2022.^4^ Cuproptosis is induced by the accumulation of copper in mitochondria, which promotes the generation of lipoylated dihydrolipoamide S-acetyltransferase (DLAT), an important component of the pyruvate dehydrogenase complex associated with the mitochondrial tricarboxylic acid (TCA) cycle. This process leads to oxidative stress, mitochondrial dysfunction, and ultimately cell death.^5^ Cuproptosis predominantly occurs in cells relying on oxidative phosphorylation (OXPHOS) as their primary energy-generating metabolic pathway and is characterized by the aggregation of DLAT and the loss of iron–sulfur cluster (Fe–S) proteins.^6−8^ Copper ionophores such as elesclomol and disulfiram are lipid-soluble molecules that reversibly bind copper ions. They have played a significant role in the discovery and application of cuproptosis and may be involved in clinical treatment as antitumor agents.^9,10^

Reactive oxygen species (ROS) primarily originate from intracellular redox reactions, with mitochondria playing a central role,^11^ which is consistent with the relationship between cuproptosis and mitochondrial metabolism. Research indicated that an excessive Cu^2+^ level elevates ROS and mediates cell death via mitochondria-related physiological processes. For example, copper transport reduces mitochondrial protein levels, increases ROS, and inhibits tumor cell proliferation in melanoma.^12^ Besides, Cu^2+^ amplifies ROS levels by oxidizing molecules like ascorbic acid or interacting with the mitochondrial respiratory chain, leading to significant cytotoxic effects.^13,14^

Tumor cells exhibit active metabolic features that lead to the accumulation of ROS in the cells. Peroxiredoxins (PRDXs), a family of intracellular antioxidant enzymes,^15,16^ are crucial regulators of cellular processes including cell growth, differentiation, and metabolism.^17^ PRDX1 plays a pivotal role in cancer progression by reducing ROS levels, inhibiting ferroptosis, and regulating pathways such as PI3K/AKT/TRAF1.^18−20^ PRDX1 could enhance the aggressive survival of cancer cells from oxidative stress damage, promote malignant transformation, and confer increased resistance to chemotherapy and radiotherapy.^21,22^ Based on the above analysis, we speculated that PRDX1 may be involved in cuproptosis. Currently, only a limited number of PRDX1 inhibitors have been reported, including celastrol, adenanthin, parvifoline AA, piericidin A, frenolicin B, H7, ainsliadimer A, and 18-β-glycyrrhetinic acid.^22−28^ However, most of them lack PRDXs’ isoform selectivity or moderate enzymatic potency, limiting their potential for clinical application.

Natural products (NPs) are highly valued for their structural diversity, biological activity, and low toxicity and have proven instrumental in targeted cancer therapy. Approximately 50% of current anticancer drugs are derived directly or indirectly from NPs.^29^ Resorcylic acid lactones (RALs) are a class of fungal-derived polyketides that feature a β-resorcylic acid moiety incorporated into a macrolactone ring.^30^ The first RAL, radicicol, was isolated in 1953 from the fungus *Monocillium nordinii*.^31^ Since then, more than 200 different RALs have been reported from various fungal genera such as *Ilyonectria*, *Aigialus*, *Curvularia*, *Lasiodiplodia*, *Penicillium*, and *Pochonia*.^32^ RALs exhibit a range of biological activities, including anticancer, antimicrobial, and antimalarial effects, as well as inhibition of Hsp90 and protein tyrosine kinase inhibition. Recently, we have concentrated our efforts on investigating secondary metabolites produced by plant endophytic fungi, exploring their antitumor activities and underlying mechanisms, resulting in the isolation of several structurally and biologically interesting NPs.^33−41^

In this study, we aimed to explore the chemical diversity of *Ilyonectria* sp. FL-710 using a metabolomics approach combined with antitumor activity-guided separation, identified new potential antitumor agents, and uncovered their novel target mechanisms for TNBC treatment. A total of 24 RALs (**1**–**24**), including 9 previously unreported ones, were isolated. The pharmacological mechanisms of pochonin D (PoD, **16**) were investigated with proteomics, bioinformatics, CMap, OTTER, clinical tissue samples, and a series of functional assays. Mechanistically, PRDX1 was involved in the process of cuproptosis, and PoD bound to PRDX1, inhibited its enzymatic activity, and intervened with cuproptosis to exert anti-TNBC activity *in vivo* and *in vitro*.

## Results

### Screening Endophytic Fungi Fermentation Extracts for Antitumor Activity

**Figure 1:**
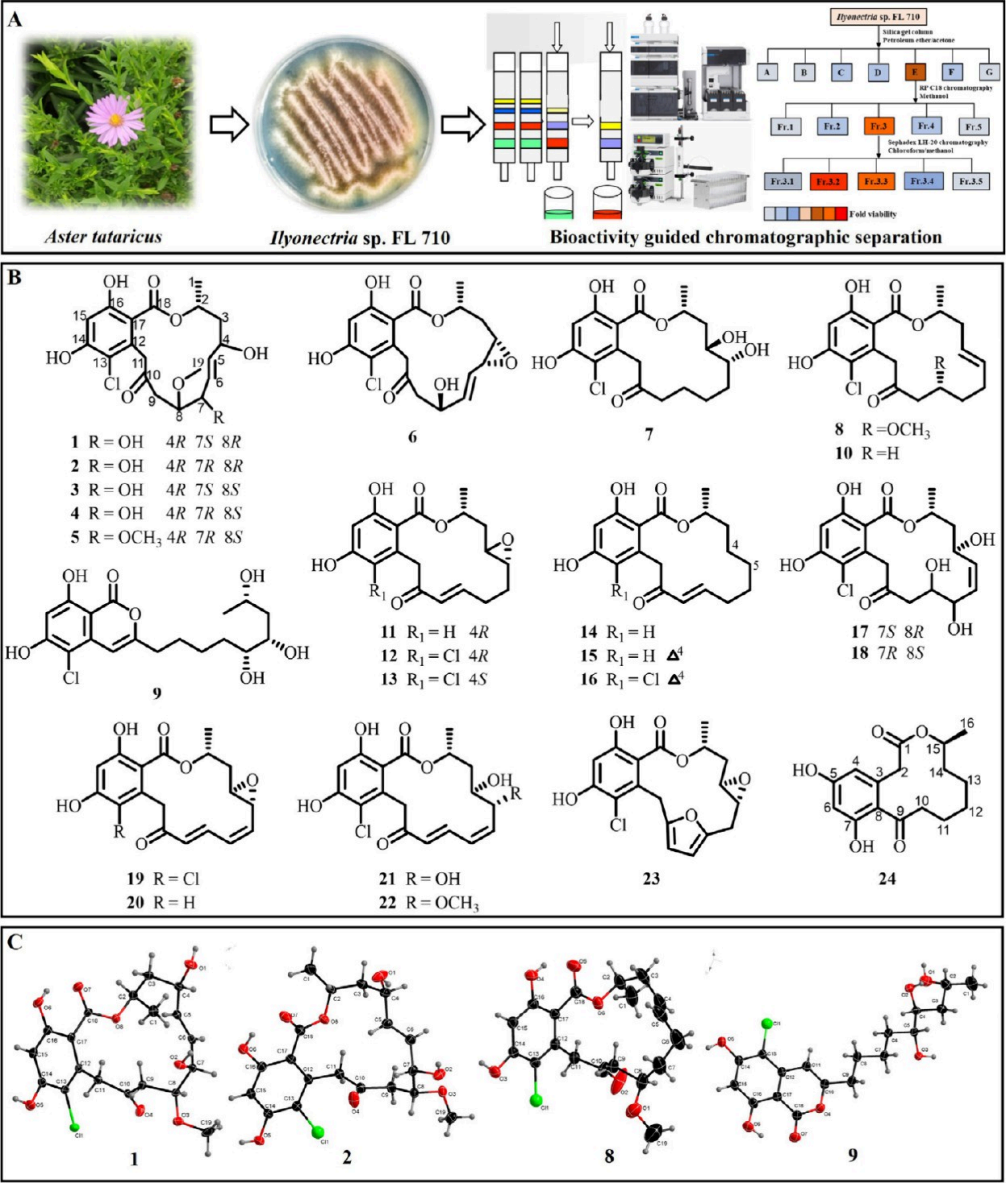
Antitumor activities-directed separation of RALs. (A) Schematic diagram of separation and purification of natural RALs. (B) Chemical structures of **1**–**24**. (C) X-ray diffraction crystal structures of **1**, **2**, **8**, and **9**.

In the preliminary study,^37,40,41^ we obtained more than 1,600 endophytic fungi from traditional Chinese medicine, including *Rubia cordifolia*, *R. podantha*, *Aster tataricus*, and *Pseudostellaria heterophylla*. Bioassay-guided isolation was commonly employed to discover potential clinically effective antitumor molecules. As part of this process, the rice fermentation extracts of these endophytic fungi were obtained and screened for their antineoplastic activity. As shown in Table S1, several extracts exhibited potent cytotoxic effects on the tested cancer cell lines. Based on these results, combined with a metabolomics map (Figure S1), strain *Ilyonectria* sp. FL-710 from *A. tataricus* was chosen for further study of its secondary metabolites (Figure 1A).

### Isolation and Structure Elucidation

A total of 24 RALs (**1**–**24**) including 9 previously unreported ones (**1**–**9**) were isolated from the plant endophytic *Ilyonectria* sp. FL-710 (Figure 1B). Their structures with full configurations were determined by comprehensive spectroscopic analysis, NMR and ECD calculations, and X-ray crystallography (Figures 1C and S2–S6).

Ilyolactone A (**1**) was obtained as colorless crystals, whose molecular formula was established as C_19_H_23_ClO_8_ (8 degrees of unsaturation) based on HRESIMS ([M + Na]^+^, 437.09760, calcd 437.09737) and supported by ^1^H and ^13^C NMR spectroscopic data (Table S2). The IR spectrum showed absorption bands at 2991, 2891, 1384, 1240, 1050, and 654 cm^–1^, which indicated the presence of hydroxyl, carbonyl, and double bond functional groups. The UV spectrum exhibited maximum absorption bands at λ_max_ = 216, 250, and 313 nm, suggesting that **1** contained a conjugated double bond system. The 1D NMR spectra and HSQC spectrum of **1** revealed the presence of two sp^3^ methyl 1-CH_3_ (δ_H_/δ_C_ 1.41/20.8) and 19-CH_3_ (δ_H_/δ_C_ 3.42/58.3); three sp^3^ methylene 3-CH_2_ (δ_H_/δ_C_ 2.20, 2.03/43.2), 9-CH_2_ (δ_H_/δ_C_ 2.89, 2.68/43.3), and 11-CH_2_ (δ_H_/δ_C_ 4.61, 4.21/47.3); four sp^3^ methine 2-CH (δ_H_/δ_C_ 5.52/71.3), 4-CH (δ_H_/δ_C_ 4.33/70.8), 7-CH (δ_H_/δ_C_ 4.34/73.7), and 8-CH (δ_H_/δ_C_ 3.78/80.6); six aromatic groups 12-C (δ_C_ 109.2), 13-C (δ_C_ 116.3), 14-C (δ_C_ 162.8), 15-CH (δ_H_/δ_C_ 6.48/104.0), 16-C (δ_C_ 159.3), and 17-C (δ_C_ 136.3); one trans-double bond 5-CH (δ_H_/δ_C_ 5.49/136.1) and 6-CH (δ_H_/δ_C_ 5.67/131.0); one keto carbonyl CO-10 (δ_C_ 206.7) and one ester carbonyls COO-18 (δ_C_ 171.3). Preliminary analysis of the NMR data indicated that **1** has a RALs macrolide skeleton structure. As shown in Figure S2, the ^1^H–^1^H COSY correlations (H-1/H-2/H-3/H-4/H-5/H-6/H-7/H-8/H-9) led to the identification of fragments from C-1 to C-9. The HMBC correlations (from H-15 to C-13, C-14, and C-16; from H-11 to C-10, C-12, C-13, and C-17; from H-2 to C-18; and from H-9 to C-10) combined with chemical shift values indicated that the benzene moiety was fused with a 14-membered lactone ring through C-12–C-17, suggesting **1** should be a RAL_14_ (Figure 1B). The downfield ^13^C NMR chemical shifts of C-4, C-7, C-8, C-14, and C-16, along with the HMBC correlations from H-19 to C-8, exhibited the presence of 4-OH, 7-OH, 8-OCH_3_, 14-OH, and 16-OH. The ^13^C NMR chemical shift of C-13 and the HRESIMS data indicated that the substituent in C-13 should be a chlorine atom. Due to the flexible structure of **1**, the ROESY spectrogram could not be used for its relative configuration identification. Therefore, the absolute configuration of **1** was determined to be 2*R*, 4*R*, 7*S*, and 8*R* by single-crystal X-ray diffraction analysis (Cu Kα) with a Flack parameter of 0.091(15). Thus, the structure of **1** was elucidated (Figure 1B) and named ilyolactone A.

Detailed structure elucidation of **2**–**24** is depicted in the Supporting Information. Compounds **1**–**6** represent the first examples of RALs with *E* configurations in C-5–C-6 and C-6–C-7.

### Discovery of Pochonin D as Potent Inhibitor of TNBC *In Vivo* and *In Vitro*

**Figure 2:**
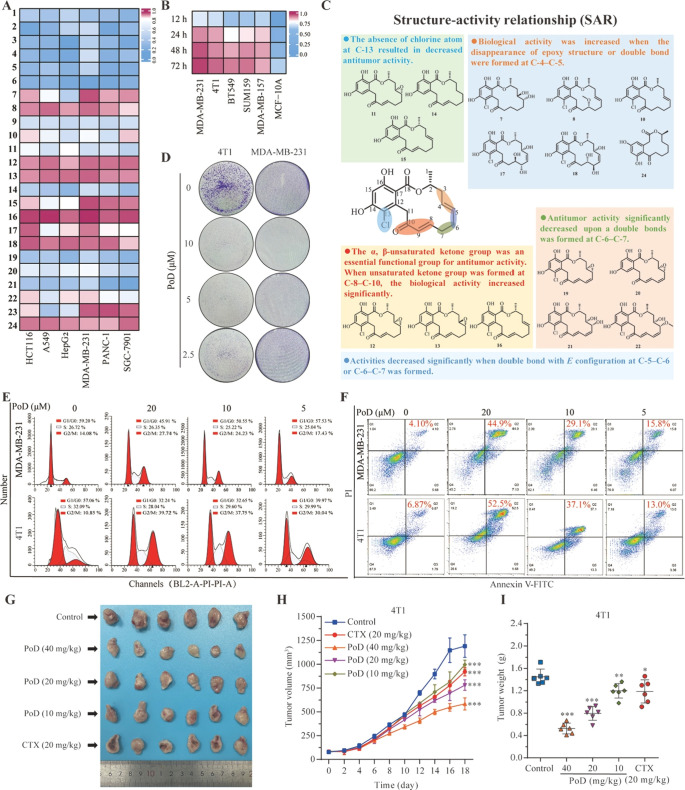
Pochonin D inhibits TNBC cell growth *in vivo* and *in vitro*. (A) The cytotoxicity of isolated compounds against six cancer cell lines, including HCT116, A549, HepG2, MDA-MB-231, PANC-1, and SGC-7901 cells. (B) The cell viability of PoD-treated breast cancer cells. The cells were exposed to various concentrations of PoD for 12, 24, 48, or 72 h. The cell viability was measured using the SRB assay. (C) Structure–activity relationship analysis of RALs. (D) Representative images of colonies formed after treatment with PoD. The cells were exposed to various concentrations of PoD for 24 h and then cultured for an additional 14 days, and the numbers of colonies were photographed. (E) PoD influenced the cell cycle in MDA-MB-231 and 4T1 cells. The cell cycle was analyzed by PI staining and detected using flow cytometry. (F) PoD induced apoptosis in MDA-MB-231 and 4T1 cells. The cells were treated with indicated concentrations of PoD for 24 h followed by staining with Annexin V/PI, and apoptosis was determined by flow cytometry. (G) Tumors removed were photographed. Female BALB/c mice bearing 4T1 xenograft tumors were intraperitoneally injected with various doses of PoD or a control every other day. CTX (20 mg/kg) group as a positive control. (H and I) The effects of PoD on the growth curves of xenografts of 4T1 and the effects on the tumor weight. * *p* < 0.05, ** *p* < 0.01, *** *p* < 0.001 vs control.

RALs were natural small-molecules attracting great attention as potential therapeutics for cancer treatment, so we first tried to assess their antitumor activity. All obtained compounds were evaluated for their cytotoxicity in six human cancer cell lines (HCT116, A549, HepG2, MDA-MB-231, PANC-1, and SGC-7901 cells). The results showed that pochonin D (PoD, **16**) displayed the most pronounced activities on all tested cells (Figure 2A and Table S5), which was superior to the first-line drug cyclophosphamide, cis-platin, and 5-FU (Table S6). Further analysis indicated that PoD exhibited dose- and time-dependent inhibitory activity on TNBC cell lines (MDA-MB-231, 4T1, BT549, SUM159, and MDA-MB-157), but was less toxic to normal breast cell MCF-10A (Figure 2B and Table S7).

In order to investigate the molecular features responsible for the antitumor activity of the isolated RALs, a detailed structure–activity relationship (SAR) study (Figure 2C) was conducted and is summarized as follows: (i) The α,β-unsaturated ketone group was an essential functional group for antitumor activity. When an unsaturated ketone group was formed at C-8–C-10, the biological activity increased significantly. (ii) Biological activity was also increased when an epoxy structure and double bond were formed at C-4–C-5. (iii) The absence of a chlorine atom at C-13 resulted in decreased antitumor activity. (iv) Antitumor activity significantly decreased when a double bond was formed at C-6–C-7. These SAR findings will benefit structural modifications and drug development of RALs.

Based on the cytotoxic data and universalities of TNBC cell lines in the preclinical study, we chose MDA-MB-231 and 4T1 cells to investigate the potential antitumor mechanism of PoD. A clonogenic assay was performed to evaluate its effects on cell proliferation, and the results showed that PoD could dose-dependently reduce the clonogenicity of MDA-MB-231 and 4T1 cells (Figure 2D). In addition, we performed a cell cycle assay using a flow cytometer, revealing that PoD obviously accumulated the cells in the G_2_/M phase while decreasing the cells in the G_1_/G_0_ phase (Figure 2E). Moreover, PoD could also induce cell apoptotic death (Figure 2F). To determine the *in vivo* antitumor efficacy of PoD, we established TNBC xenograft mouse models. As shown in Figure 2G–I, PoD dose-dependently suppressed the growth of TNBC xenograft tumors with obvious reduction in the tumor weight and volume compared to the vehicle group. Meanwhile, there were no significant differences in body weight, hematoxylin and eosin staining, or the levels of some representative serum markers for liver damage between the vehicle and the PoD-treated groups (Figure S7). Taken together, these results demonstrated that PoD exhibited potent cytotoxicity and induced apoptosis in TNBC tumor cells, ultimately leading to tumor necrosis *in vivo* without notable toxicity at effective doses.

### Pochonin D Triggers Cuproptosis in TNBC Cells

**Figure 3:**
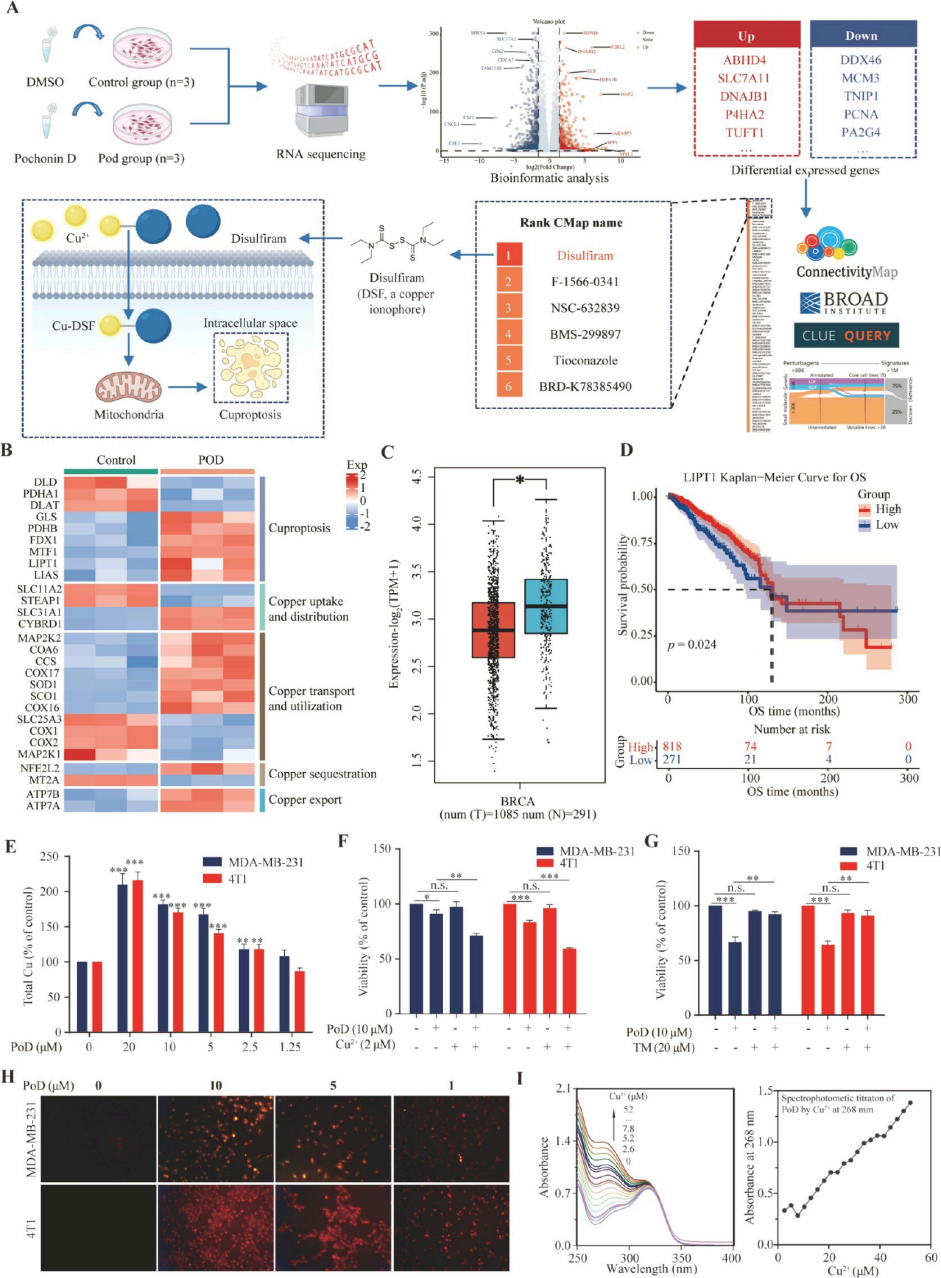
**Pochonin D triggers cuproptosis in TNBC cells**. (A) The query genes of PoD were sequenced and submitted to the CMap database for analysis. (B) Heatmap of differentially expressed genes related to cuproptosis of PoD-treated MDA-MB-231 cells. (C) Statistic analysis of LIPT1 expression in breast cancer. (D) Overall survival and the cumulative recurrence-free survival curves of patients with high or low LIPT1 expression in breast cancer tissues were evaluated by Kaplan–Meier curves. (E) Addition of PoD leads to an increase in total cellular copper for 24 h in MDA-MB-231 and 4T1 cells. (F) Cu^2+^ could enhance the cytotoxicity of PoD on TNBC cells. MDA-MB-231 and 4T1 cells were cultured in serum-free medium, pretreated with 2 μM Cu^2+^, and incubated with indicated concentrations of PoD for 12 h, and the cell viability was measured. (G) TM could rescue the cytotoxicity of PoD on TNBC cells. MDA-MB-231 and 4T1 cells were cultured in serum-free medium, pretreated with 20 μM TM, and incubated with indicated concentrations of PoD for 18 h, and the cell viability was measured. (H) Fluorescence microscope images of the RBH probe in MDA-MB-231 and 4T1 cells after treatment with PoD. (I) Spectrophotometric titration of PoD by Cu^2+^. UV–vis spectral changes observed when microliter amounts of CuCl_2_ were added to 20 μM PoD in Tris buffer (10 mM, pH 7.4, 25% DMSO) at 37 °C in a 1 cm spectrophotometer cell. * *p* < 0.05, ** *p* < 0.01, *** *p* < 0.001 vs control.

PoD inhibited the cell growth of TNBC cells *in vivo* and *in vitro*, but its specific mechanisms were unknown. To further explore the potential mechanism of PoD in TNBC cells, RNA-sequencing of PoD-treated MDA-MB-231 cells was performed, and their differentially expressed genes were analyzed. The Connectivity Map (CMap), a groundbreaking tool in biomedical research, provides a data-driven and systematic framework for uncovering relationships among genes, chemicals, and biological conditions, including diseases. Since its introduction in 2006, CMap has shown immense promise in accelerating drug discovery and clarifying the mechanisms of novel natural products. The gene expression profiles of MDA-MB-231 cells in response to PoD including 225 query genes (≥1.2-fold change and *p* < 0.05; 128 upregulated and 97 downregulated) were submitted to the CMap database for analysis (Figure 3A). The results showed that PoD was most positively correlated to disulfiram, a known cuproptosis inducer that led to cancer cell death. Therefore, we further analyzed the differentially expressed genes of cuproptosis using heatmaps, and the results showed that PoD could significantly influence cuproptosis-related regulatory genes (Figure 3B). Among these differentially expressed genes, lipoyltransferase 1 (LIPT1) has been recently identified as a cuproptosis-related gene in TNBC. Evaluation of LIPT1 expression in breast cancer using TCGA data revealed that LIPT1 was significantly decreased in breast cancer, and upregulation of LIPT1 expression was associated with significantly higher survival, which was analyzed with the 220-month data (*p* < 0.05) (Figure 3C,D). Then, we investigated whether PoD affected copper homeostasis. We employed a cell copper (Cu^2+^) colorimetric assay kit to measure the total intracellular Cu^2+^ content, and the results showed that PoD increased intracellular copper content (Figure 3E). Co-treatment with PoD and Cu^2+^ modestly reduced cell viability, whereas the cytotoxicity would be weakened after exposure to PoD and copper chelator TM (Figures 3F and 3G). In addition, the total Cu^2+^ levels in MDA-MB-231 and 4T1 cells treated with PoD were also determined by RBH fluorescent probes with fluorescence microscope imaging (Figure 3H). In order to characterize complex formation, PoD was titrated with CuCl_2_, and the titration was monitored by spectrophotometry in Tris buffer (10 mM, pH 7.4, 37 °C) containing 25% (v/v) DMSO. As shown in Figure 3I, the absorbance at the 268 nm shoulder systematically increased with the addition of Cu^2+^. Besides, when PoD coincubated with Cu^2+^, the latter will promote the chemical shift value of PoD to the higher field (Figure S8). These findings suggested that PoD promoted cell deaths by inducing intracellular copper accumulation.

### PRDX1 is Identified as a Direct Target of Pochonin D and is Highly Expressed in TNBC Correlating with Poor Survival

**Figure 4:**
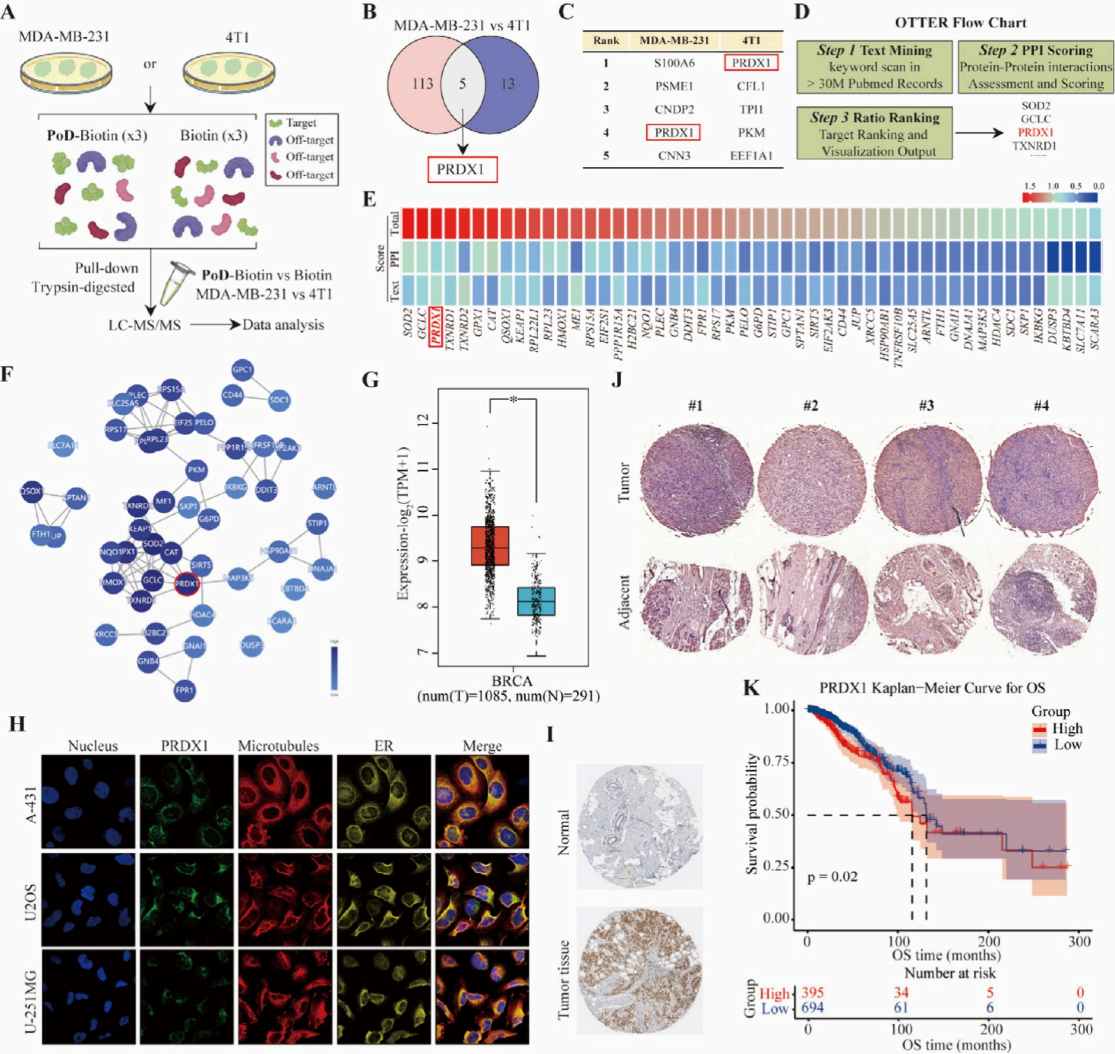
**PRDX1 is identified as a direct target of pochonin D and is highly expressed in TNBC correlating with poor survival**. (A) Overall scheme of the target fishing of PoD in MDA-MB-231 and 4T1 cells. (B) Potential PoD-targeted protein in both MDA-MB-231 and 4T1 cells presented with a Venn diagram. (C) The top 5 candidate proteins identified by mass spectrometry analysis in MDA-MB-231 and 4T1 cells, respectively. (D) The workflow of OTTER consists of three steps, including text mining in PubMed abstracts, scoring with protein–protein interactions, and ranking with visualization. (E) The rule was used by OTTER for protein–protein interaction assessment. After the calculation of the Text Scores, these genes are scored with the protein–protein interaction records from the STRING database. The genes involved in more protein–protein interactions with other genes on the list were given higher PPI Scores. The top-ranking differentially expressed genes according to total scores calculated by OTTER, using the RNA sequencing data from cells treated with PoD and the keyword “reactive oxygen species”. (F) The interactive plot was generated by OTTER, using the top-ranking 50 differentially expressed genes according to final scores. Colored nodes represent top-ranking genes after enrichment, while the lines represent protein–protein interactions between these genes. Nodes with higher scores are colored darker blue. (G) Statistic analysis of PRDX1 expression in breast cancer and adjacent tissues. (H) Representative images of immunofluorescence staining for PRDX1 protein from the HAP database for the A-431, U2OS, and U-251MG cell lines. (I) Expression of PRDX1 in immunohistochemical staining of breast cancer from the TCGA Database. (J) Representative immunohistochemical staining of human TNBC samples from a clinical breast tissue microarray. After antigen retrieval and blocking, sections were incubated overnight at 4 °C with primary antibodies against PRDX1 (Proteintech, Cat no: 15816–1-AP). After being washed with PBS, sections were incubated with a secondary antibody for 2 h, visualized using diaminobenzidine (DAB), stained with hematoxylin, and mounted with neutral resin. All stained sections were examined under a microscope. (K) Overall survival and cumulative recurrence-free survival curves of patients with high or low expression of PRDX1 in breast cancer tissues were evaluated by Kaplan–Meier curves.

The prominent effects of PoD triggering cuproptosis in TNBC prompted us to identify its specific anticancer targets. Various target identification techniques and mass spectrometry (MS) analysis were accordingly performed (Figure 4A–F). To explore and identify the cellular targets of PoD, a biotin-tagged PoD probe (PoD-biotin) was designed and synthesized (Figures S9 and S10), which retained cytotoxic activity comparable to that of PoD. We incubated PoD-biotin in MDA-MB-231 and 4T1 cell lysates using biotin as the control. The treated cell lysates were subsequently subjected to pull-down and trypsin digestion, followed by MS analysis. Then, the MS data from each cell type were analyzed to calculate the relative abundance ratio between the two groups (PoD-biotin *vs* biotin in MDA-MB-231 and PoD-biotin *vs* biotin in 4T1). After applying a cutoff of 1.5 for the relative abundance ratio, we found that PRDX1 was the only protein (top five ranks) in both comparison experiments (Figure 4B,C). This approach effectively enabled the identification of the potential targets by minimizing false positives from indirect or nonspecific binding to the probes.

A common strategy employed by existing computational target discovery tools is to compare the chemical structures of active compounds with those of previously reported inhibitors for known target proteins. Omics and text-based target enrichment and ranking (OTTER) is a new computational tool to facilitate the target discovery.^22^ To provide the required gene list as input by OTTER, RNA sequencing data from MDA-MB-231 cells treated with PoD were analyzed. Given the involvement of reactive oxygen species (ROS) in cuproptosis, the keyword “ROS” was selected to enrich redox-related targets of PoD using OTTER (Figure 4D). Notably, several genes, including PRDX1, were identified in the enrichment analysis. Additionally, other proteins known to interact with PRDX1, such as TXNRD1, TXNRD2, SOD2, GCLC, and DDIT3,^42−44^ were also enriched (Figure 4E,F).

Considering that all the above evidence provides preliminary validation of PRDX1 as the potential target of PoD, we further investigated the uncharted relationships between PRDX1 with TNBC and PoD. The expression of PRDX1 in breast cancer was evaluated using TCGA data, which revealed that PRDX1 is highly expressed in breast cancer and localized in the cytoplasm (Figure 4G–I). Additionally, an immunohistochemistry assay was also performed on a clinical tissue microarray containing both adjacent normal and breast tumor samples from human patients. As illustrated in Figure 4J, a significant upregulation of PRDX1 expression was observed in tumor tissues. Notably, BC patients with high expression of PRDX1 have significantly poorer prognosis and shorter survival (*p* < 0.05) compared to those with low expression (Figure 4K). Taken together, these results indicated that PRDX1 is highly expressed in TNBC and correlates with poor survival.

### Pochonin D Exhibits Potent Binding Affinity for PRDX1

**Figure 5:**
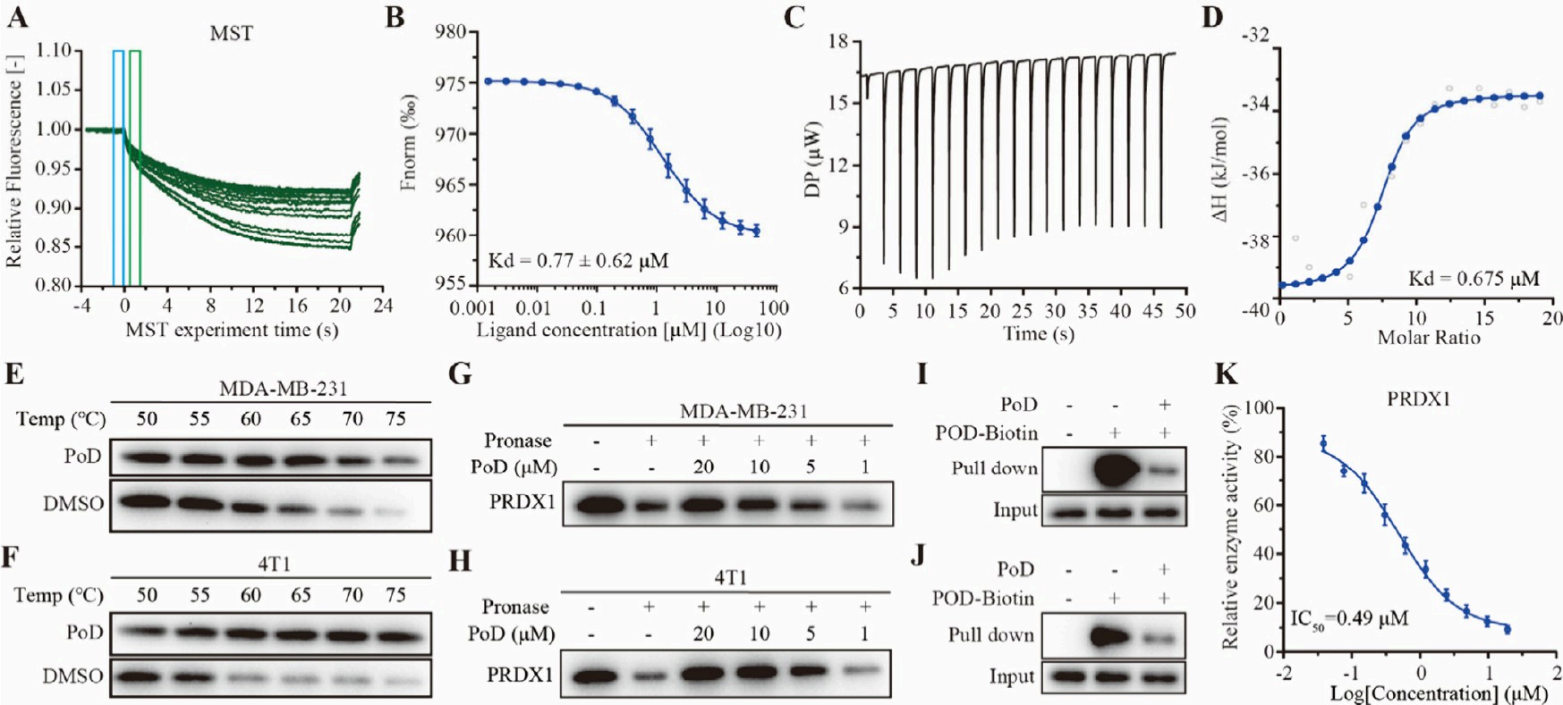
**Pochonin D exhibits a potent binding affinity for PRDX1**. (A and B) MST analysis of interactions between PoD and PRDX1. (C and D) ITC analysis of interactions between PoD and PRDX1. (E and F) CETSA analysis of intracellular binding between PoD and PRDX1. Protein levels were investigated at different temperatures under the treatment of PoD (20 μM) in MDA-MB-231 and 4T1 cells. (G and H) DARTS analysis between PoD and PRDX1. (I and J) PoD and PRDX1 competitive ligand binding assay. The recombinant PRDX1 protein treated with PoD-biotin in the absence or presence of PoD for the competitive binding, and followed by protein affinity pull-down assay. The PRDX1 bound to the PoD-biotin were detected by immunoblotting. (K) The inhibitory enzyme activity of PoD against PRDX1.

In order to further verify the interaction between PoD and PRDX1, microscale thermophoresis (MST) analysis was performed. The results showed that PoD could bind to PRDX1 with a higher affinity (*K*_d_ = 0.77 μM) (Figure 5A,B). This binding was further validated through isothermal titration calorimetry (ITC) experiments, which revealed *K*_d_ values of 0.675 μM (Figure 5C,D). Additionally, a cellular thermal shift assay (CETSA) was employed to evaluate their binding affinity. The results indicated that PoD stabilized PRDX1 at higher temperatures compared to the DMSO treatment (Figure 5E,F). The drug affinity responsive target stability (DARTS) assay was also conducted, and as anticipated, the PRDX1 protein showed increased protease resistance in the presence of PoD (Figure 5G,H), confirming a direct interaction between PoD and PRDX1. Notably, the binding of PoD to PRDX1 was inhibited when cells were preincubated with PoD in both MDA-MB-231 and 4T1 cell lysates (Figure 5I,J). The enzymatic activity of PRDXs was evaluated, and the results showed that PoD inhibited PRDX1 activity with an IC_50_ value of 0.49 μM (Figure 5K). In comparison, PoD exhibited relatively lower enzyme inhibitory activity against other PRDX isoforms, with IC_50_ values of 10.47 μM for PRDX2, 5.06 μM for PRDX3, 2.07 μM for PRDX4, 9.74 μM for PRDX5, and 3.98 μM for PRDX6 (Figure S11). Under physiological conditions, a moderate increase in ROS promotes cellular proliferation and differentiation. However, excessive ROS levels induce oxidative damage to DNA, proteins, and lipids. Peroxiredoxins scavenge ROS via their peroxidase activity by reducing hydrogen peroxide and a wide range of organic hydroperoxides. Therefore, we further investigated whether PoD could influence the expression level of intracellular ROS. First, the levels of ROS in MDA-MB-231 and 4T1 cells after treatment of PoD were detected using the Carboxy-H_2_DCFDA fluorescent probes, and the results showed that PoD markedly enhanced the generation of ROS (Figure S12). N-acetylcysteine (NAC) and l-glutathione (GSH), two common antioxidants, could eliminate PoD-induced ROS production and rescue the cell viability (Figure S13). Collectively, these findings strongly suggest that PRDX1 is a specific target of PoD, and PoD exerted antitumor activity via inducing ROS accumulation in MDA-MB-231 and 4T1 cells.

### Cysteine-173 Is the Covalent Binding Site for Pochonin D on PRDX1

**Figure 6:**
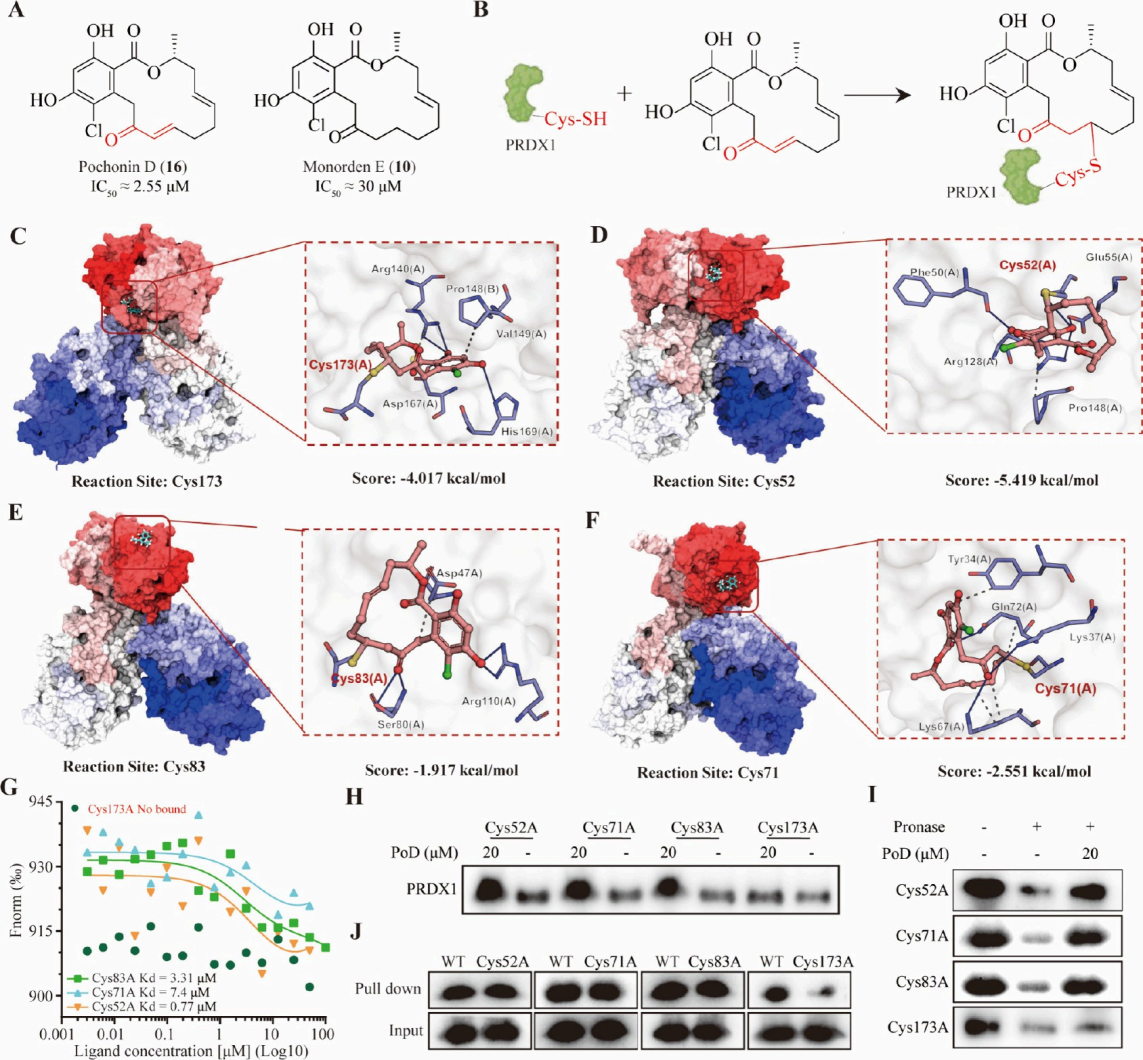
**Cysteine-173 is the covalent binding site for pochonin D on PRDX1.** (A) Structures of pochonin D and monorden E (α,β-unsaturated ketone moiety was reduced). (B) Proposed mechanism of interaction between PoD and PRDX1. (C–F) The interaction of PoD with PRDX1 protein with Cys173, Cys52, Cys83, and Cys71 as covalent binding site and detailed protein–ligand complex interactions. (G) MST analysis of interactions between PoD and different mutant PRDX1 proteins. (H and I) CETSA and DARTS analysis of binding between PoD and different PRDX1 mutant proteins. (J) The mutant PRDX1 proteins were incubated with PoD, followed by protein affinity pull-down assay, and detected by immunoblotting.

The α,β-unsaturated ketone moiety of PoD functions as a reactive Michael acceptor to form a covalent bond with cysteine residues in proteins. To further certify that PoD covalently binds to PRDX1 via its α,β-unsaturated ketone moiety, we compared the cytotoxicity of PoD with monordene E (**10**), a derivative lacking this functional group. Strikingly, monorden E had an obviously lower effect on the proliferation of tested cancer cell lines (Figure 6A), suggesting that the cytotoxicity of PoD depended on the α,β-unsaturated ketone moiety (Figure 6B). PRDX1 contains four cysteines (Cys52, Cys71, Cys83, and Cys173). To identify the binding site of PoD, docking simulations were performed to model its interaction with each cysteine residue. As shown in Figure 6C–F, PoD covalently bound to the PRDX1 protein by contacts with scores of −5.419 kcal/mol for Cys52, −4.017 kcal/mol for Cys173, −1.917 kcal/mol for Cys83, and −2.551 kcal/mol for Cys71. To validate which cysteine residue serves as the actual binding site, an MST assay was conducted with PRDX1 mutants where each cysteine was individually replaced with alanine. As shown in Figure 6G, mutations at Cys52, Cys71, and Cys83 had less influence on the *K*_d_ value. However, when Cys173 was mutated, the binding between PoD and PRDX1 was completely abolished. Additionally, CETSA, DARTS, and pull-down experiments were also performed. As expected, compared with other mutant-type PRDX1, PRDX1-Cys173A had no binding to PoD (Figure 6H–J). In conclusion, these results suggested that PoD covalently binds to PRDX1 through its α,β-unsaturated ketone moiety, specifically targeting Cys173 of the protein.

### PRDX1 Involved in Physiological Function of TNBC and Pochonin D Exhibits Anti-TNBC Activity by Targeting PRDX1 to Stimulate Cuproptosis

**Figure 7:**
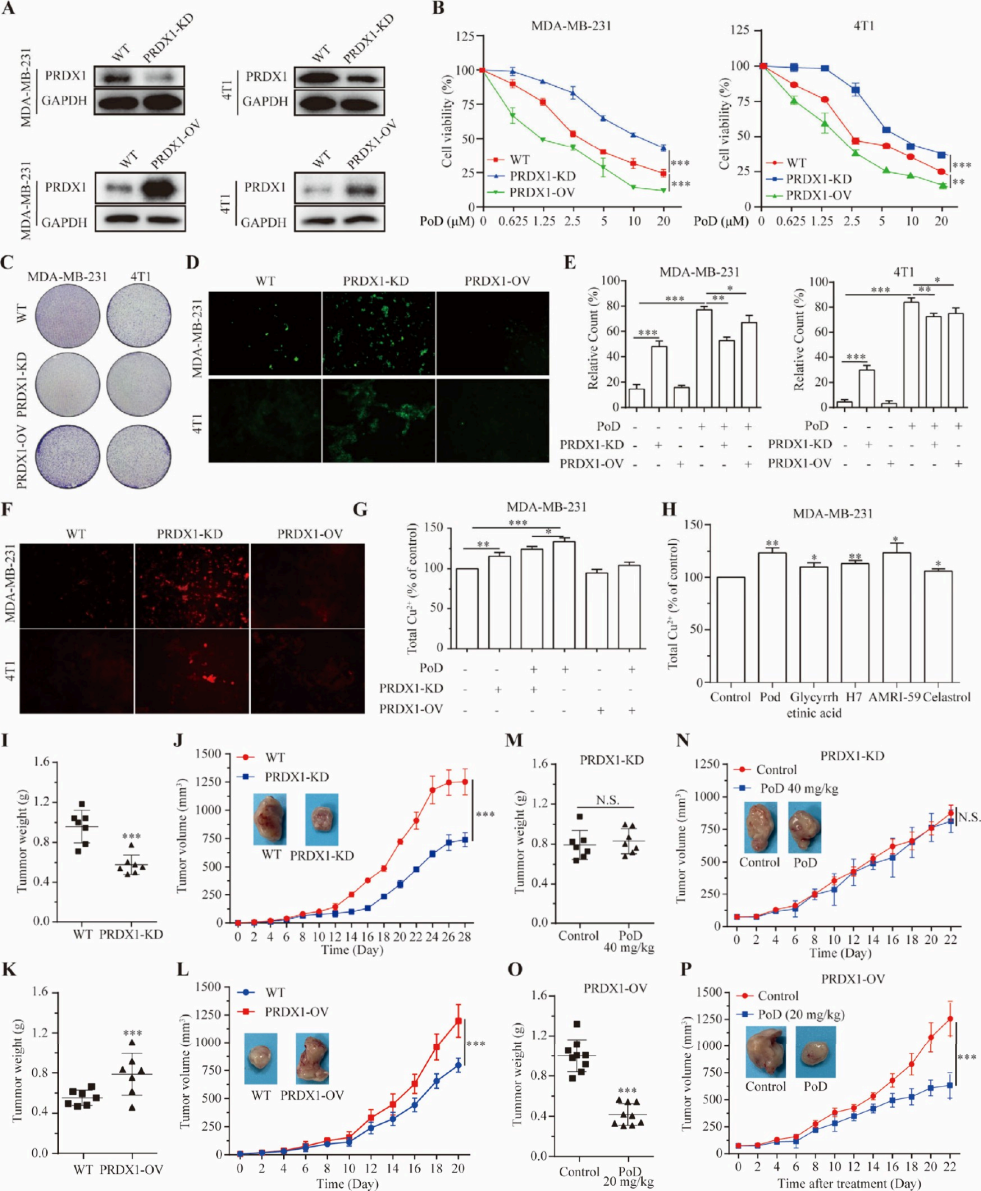
**PRDX1 involved in physiological function of TNBC and pochonin D exhibits anti-TNBC activity by targeting PRDX1 to stimulate ROS and cuproptosis.** (A) Building PRDX1-KD or PRDX1-OV TNBC cells. (B) The cell viability of PoD against WT, PRDX1-KD, or PRDX1-OV TNBC cells. (C) Cell proliferative activity of WT, PRDX1-KD, and PRDX1-OV TNBC cells after culture for 21 days with the colony formation assay. (D) The levels of ROS production in WT, PRDX1-KD, or PRDX1-OV TNBC cells were evaluated by fluorescence microscope. (E) The effects of PoD (10 μM) on the ROS production in WT, PRDX1-KD, or PRDX1-OV TNBC cells were evaluated by a flow cytometer. (F) The contents of Cu^2+^ in WT, PRDX1-KD, and PRDX1-OV TNBC cells were evaluated by fluorescence microscope. (G) The effects of PoD on the contents of Cu^2+^ in WT, PRDX1-KD, and PRDX1-OV TNBC cells after treatment with or without PoD (10 μM) were evaluated by a cell copper (Cu^2+^) colorimetric assay kit. (H) The effects of PRDX1 inhibitors on the contents of Cu^2+^ in MDA-MB-231 cells were evaluated by cell copper (Cu^2+^) colorimetric assay kit. (I) Tumor weight of WT and PRDX1-KD 4T1 cells in mouse model. (J) Tumor volume and represent images of WT and PRDX1-KD 4T1 cells in mouse model. (K) Tumor weight of WT and PRDX1-OV 4T1 cells in mouse model. (L) Tumor volume and represent images of WT and PRDX1-OV 4T1 cells in mouse model. (M) Tumor weight of PRDX1-KD 4T1 cells after treatment with PoD (40 mg/kg) in mouse model. (N) Tumor volume and represent images of PRDX1-KD 4T1 cells after treatment with PoD (20 mg/kg) in mouse model. (O) Tumor weight of PRDX1-OV 4T1 cells after treatment with PoD (20 mg/kg) in mouse model. (P) Tumor volume and represent images of PRDX1-OV 4T1 cells after treatment with PoD (20 mg/kg) in mouse model. * *p* < 0.05, ** *p* < 0.01, *** *p* < 0.001 vs control.

To further investigate the role of PRDX1 in TNBC cells, we generated PRDX1 knockdown (PRDX1-KD) and overexpressed (PRDX1-OV) MDA-MB-231 or 4T1 cells. The results of immunoblotting confirmed the effective knockdown or overexpression of PRDX1 in both TNBC cells (Figure 7A). Reduced and increased PRDX1 expression notably inhibited and promoted TNBC cell proliferation, respectively (Figure 7B,C). Specifically, knockdown of PRDX1 substantially decreased the inhibitory effect of PoD on cell proliferation, while overexpression of PRDX1 enhanced the sensitivity of MDA-MB-231 and 4T1 cells to PoD. The ROS levels were also elevated in PRDX1-KD TNBC cells (Figure 7D,E), which were consistent with the effects observed following PoD treatment. In view of the close association between cuproptosis and ROS, we further examined the relationship between PRDX1 and cuproptosis. As shown in Figure 7F,G, knockdown of PRDX1 led to an increase in Cu^2+^ levels. Furthermore, the PoD-induced elevation of Cu^2+^ was significantly attenuated in the PRDX1-KD cells. Since PoD has been shown to induce cuproptosis, we explored whether other PRDX1 inhibitors (Figure S14) could elicit a similar effect in TNBC cells. We then purchased or synthesized (Figure S15) a series of PRDX1 inhibitors and evaluated their impact on cuprotease in MDA-MB-231 cells. The results demonstrated that these PRDX1 inhibitors could induce varying degrees of copper ion accumulation (Figure 7H**)**, suggesting their potential role as copper ionophores capable of triggering cuproptosis.

To further investigate the *in vivo* biological function of PRDX1, we established a mouse model using PRDX1-KD and PRDX1-OV 4T1 cells. As shown in Figures 7I–L, S16A, and S16B, PRDX1 deficiency significantly suppressed tumor proliferation, while PRDX1-OV promoted tumor growth. Moreover, PoD exhibited no significant inhibitory effect on tumor proliferation in the PRDX1-KD 4T1 mouse model (Figures 7M,N and S16C). In contrast, the tumor sensitivity of PoD on PRDX1-OV 4T1 tumors was increased (Figures 7O,P and S16D). These results further demonstrated that PRDX1 could influence cell growth *in vivo* and that the antitumor effect of PoD on TNBC cells *in vivo* is PRDX1-dependent.

Collectively, PRDX1 is closely associated with ROS generation and cuproptosis and PoD exerts anti-TNBC activity by targeting PRDX1 to induce cuproptosis.

## Discussion

Triple-negative breast cancer (TNBC) is a highly aggressive subtype of breast cancer with a high mortality rate characterized by the absence of specific molecular targets. Cuproptosis, a copper-triggered modality of mitochondrial cell death, has been implicated in the progression of various cancers. Identifying novel effective drug targets and copper ionophores capable of inducing cuproptosis for TNBC therapy is an urgent need and a key focus in tumor treatment research. In the current study, 24 resorcylic acid lactones (RALs), including 9 previously unreported ones, were isolated from the endophyte *Ilyonectria* sp. FL-710 was derived from the stem of *Aster tataricus*. Compounds **1**–**6** represent the first example of RALs with *E* configurations in C-5–C-6 and C-6–C-7, providing new insights into potential enzyme catalytic mechanisms for chemical research. Macrolides are of great significance in the study of Chemical biology and drug development, with many having been developed into commercially available drugs, such as erythromycin, azithromycin, clarithromycin, quin erythromycin, and telithromycin. As a subclass of macrolides, the isolated RALs were evaluated for their cytotoxicity against six cancer cell lines (HCT116, A549, HepG2, MDA-MB-231, PANC-1, and SGC-7901), and the results showed that many compounds (**7**, **8**, **12**, **13**, **15**–**18**, and **22**–**24**) exhibited a certain level of cytotoxicity. The meaningful SARs were also discussed, which contributed to chemical probe synthesis and mechanism study. Moreover, pochonin D (**16,** PoD) showed promising anti-TNBC efficacy *in vitro* and *in vivo* comparable to first-line anticancer drug molecules including 5-FU, cis-platin, and cyclophosphamide. Besides, PoD exhibited a lower cytotoxicity on normal breast cells (MCF-10A) and no observable toxic effects *in vivo* during the experiments. To investigate whether PoD induces cuproptosis, transcriptome sequencing, connectivity map analysis, and related assays were performed. These results suggested that PoD promoted cell deaths by inducing intracellular copper accumulation. To explore how PoD induces cuproptosis, a biotinylated probe of PoD (PoD-biotin) was synthesized and incubated with TNBC cell lysates. The resulting products were subjected to pull-down and trypsin digestion, followed by LC-MS/MS and OTTER analysis. Thereafter, a series of functional assays, clinical tissue sample analysis, bioinformatics evaluations, and molecular dynamics experiments were conducted to demonstrate that PoD bound to PRDX1, inhibiting its enzymatic activity and inducing cuproptosis. These findings indicated a novel therapeutic approach for TNBC, highlighting PoD’s potential as an effective anticancer agent.

Previous studies have demonstrated that the natural products adenanthin and triptolide target PRDX1 to exhibit antitumor activities. However, the extraction of adenanthin from plants or its chemical synthesis poses significant challenges, and adenanthin lacks selectivity as a PRDX1 inhibitor. Furthermore, the *in vivo* toxicity of triptolide has greatly limited its clinical potential for cancer treatment. These limitations have hindered the exploration of PRDX1 inhibition across different cancer types. In contrast, PoD presents notable advantages due to its high selectivity and favorable *in vivo* safety profile, making it a more suitable tool compound for studying the mechanisms of PRDX1 inhibition. Given the potential connection between redox processes and cuproptosis and recognizing that PRDX1 is a critical redox enzyme, we hypothesized that PRDX1 inhibitors might induce copper ion accumulation in TNBC cells. To verify this hypothesis, we purchased or synthesized several PRDX1 inhibitors and evaluated their effects on copper ion accumulation in MDA-MB-231 cells. The results revealed that these inhibitors could induce copper ion accumulation to varying degrees, suggesting that PRDX1 inhibitors might act as potential copper ionophores. However, further experimental validation is required to explore this process in depth.

All in all, these findings highlighted the novelty of RALs in inducing cuproptosis. Furthermore, the results demonstrated that PRDX1 served as a clinically effective biomarker and therapeutic target associated with cuproptosis in TNBC, with PoD being a novel class of PRDX1 inhibitors. This study provided a promising template for developing novel anti-TNBC targets and effective therapeutic agents in the future.

## Supporting Information Available

Detailed structural elucidation and NMR data of **1**–**24**; experimental section; MS, UV, IR, and NMR spectra of **1**–**9**; calculations of compounds; crystallographic data for **1**, **2**, **8**, and **9** (PDF)
