# Supplementary material for: Discovery of Natural Resorcylic Acid Lactones as Novel Potent Copper Ionophores Covalently Targeting PRDX1 to Induce Cuproptosis for Triple-Negative Breast Cancer Therapy
Source: ACS Cent Sci. 2025 Feb 10;11(2):357–70. doi: 10.1021/acscentsci.4c02188 (PMC11869127; doi:10.1021/acscentsci.4c02188)
Supplement: Supplementary file 1 — oc4c02188_si_001.pdf [file oc4c02188_si_001.pdf]

Supporting Information

Discovery of Natural Resorcylic Acid Lactones as Novel Potent Copper Ionophores Covalently Targeting PRDX1 to Induce Cuproptosis for Triple Negative Breast Cancer Therapy

Li Feng,[†,#] Ti-Zhi Wu,[‡,#] Xin-Rui Guo,[†,#] Yun-Jie Wang,[†] Xin-Jia Wang,[†] Shao-Xuan Liu,[†] Rui Zhang,[†] Yi Ma,[§] Ning-Hua Tan,[†,*] Jin-Lei Bian,[‡,*] and Zhe Wang[†,*]

[†] State Key Laboratory of Natural Medicines, School of Traditional Chinese Pharmacy, China Pharmaceutical University, Nanjing 211198, People's Republic of China

[‡] State Key Laboratory of Natural Medicines, School of Pharmacy, China Pharmaceutical University, Nanjing 211198, China

[§] State Key Laboratory of Natural Medicines, School of Engineering, China Pharmaceutical University, Nanjing 211198, China

[#]These authors contributed equally to this work

*To whom correspondence should be addressed

E-mail: wangzhe@cpu.edu.cn. (Z. Wang)

bianjl@cpu.edu.cn (J.L. Bian)

nhtan@cpu.edu.cn (N.H. Tan)

Table of Contents

Table S1 The inhibitory effects of the endophytic fungi extract on cancer cells……...**8**

Figure S1 UPLC-MS profiles of ethyl acetate extracts of *Ilyonectria* sp. FL-710…………………………………………………………………...**10**

Detailed Structural Elucidation of 2−9……………………………………**11**

Figure S2 Key ^{1}H-^{1}H COSY and HMBC correlations of RALs (**1**−**9**)……………...**11**

Figure S3 All possible configurations of **3-7**………………………………………**13**

Figure S4 Experimental and calculated ^{13}C-NMR of RALs (**3**−**5**)…………………**14**

Figure S5 Experimental and calculated ECD spectra of RALs (**3**−**7**)………………**14**

Figure S6 Experimental and calculated ^{13}C-NMR of RALs (**6** and **7**)……...........**15**

Table S2 Spectroscopic data for compounds **1**–**4** (δ in ppm, J in Hz)………………**17**

Table S3 Spectroscopic data for compounds **5**–**8** (δ in ppm, J in Hz)…...….....……**18**

Table S4 Spectroscopic data for compounds **9** (δ in ppm, J in Hz)……….…...……**19**

Table S5 Cytotoxicity of RALs (**1**–**24**) against human cancer cell lines……………**19**

Table S6 Cytotoxicity of compounds against human cancer cell lines..………………**20**

Table S7 Cytotoxicity of pochonin D against breast cancer cell lines………………**20**

Figure S7 Analysis of potential side effects for treatment of PoD …………………**20**

Figure S8 NMR data of copper ion co-incubation with PoD…………………………**21**

Figure S9 Synthesis scheme and bioactivity evaluation of PoD-biotin……………...**22**

Figure S10 HPLC profiles of PoD-biotin……………………………………………...**23**

Figure S11 The inhibitory enzyme activity of PoD against PRDX2-6……………...**23**

Figure S12 POD induced ROS accumulation in MDA-MB-231 and 4T1 cells……...**24**

Figure S13 NAC and GSH on cell viability in MDA-MB-231 and 4T1 cells………...**24**

Figure S14 Chemical structure of PRDX1 inhibitors……...………………………...**25**

Figure S15 The synthesis scheme for the PRDX1 inhibitor H7……………………...**25**

Figure S16 The images of 4T1 xenograft tumors…………………………….....**26**

Experimental Sections……………………………………………………………**26**

General Experimental Procedures……………………………………………**26**

Isolation and Identification of Fungus………………………………………..…**27**

Extraction, Isolation and Purification……………………………………………**27**

Physical Constants, Spectrometric, and Spectroscopic Data……..………………29
Single Crystal X-ray Diffraction Analysis and Crystallographic Data for Ilyolactone A (**1**), Ilyolactone B (**2**), Ilyolactone H (**8**), and Ilyolactone I (**9**).…**30**
Experiment Methods for Calculational Chemistry…….……………...………**33**
Conformational Analysis ……………………………………………………**33**
ECD Calculation.…………………………….……………………….……...**33**
NMR Calculation.………………………….……………………….……. **34**
Cell Lines and Culture Conditions…….…………………………………..……**34**
Cell Viability Assay……..…………………………….………………….……...**34**
Colony Formation Assay…….…………………...……………………………**35**
Cell Cycle Analysis..………………………………………………………….…...**35**
Annexin V-FITC/PI Double Staining Assay.……….……………………………...**35**
RNA-seq Analysis...……………….………...………………………...………**36**
Connectivity Map Analysis..………………………………………….…….……**36**
Survival Curve Analysis and Expression Analysis.……………....……………..**36**
Evaluation of Intracellular Cu^{2+} Level………………...………………….…..**37**
Spectrophotometric Titration of PoD with Cu^{2+}……..…………………….…..….**37**
Streptavidin–Biotin Affinity Pull-Down Assay and LC-MS Analysis…...……..**37**
Omics and Text based Target Enrichment and Ranking Analysis.………….….. **37**
Clinical Samples and IHC Analysis……………...……………...……………...**38**
Microscale Thermophoresis (MST) Analysis……….……………….………...**38**
Isothermal Titration Calorimetry (ITC) analysis…………………...…………...**38**
Western Blot Analysis…………………………………………………...….**39**
Drug Affinity Responsive Target stability (DARTS) Analysis……...…...…...….**39**
Cellular Thermal Shift Assay (CETSA) Analysis…….....…………………...…...**40**
Pochonin D and PRDX1 Competitive Ligand Binding Assay.…….……………...**40**
Enzyme Activity Assay……………………………............................….**40**
Measurement of ROS………………………..………….............................….**40**
Knockdown and Overexpression of PRDX1……………............................…**41**
Animals and Ethical State……..………..….**41**

Safety Statement..42
Statistical Analysis..42
References...43
Figure S17 ^{1}H NMR spectrum (400 MHz,CD_3OD) of ilyolactone A (**1**) 45
Figure S18 ^{13}C NMR spectrum (400 MHz, CD_3OD) of ilyolactone A (**1**) 45
Figure S19 HSQC spectrum (400 MHz, CD_3OD) of ilyolactone A (**1**) 46
Figure S20 ^{1}H-^{1}H COSY spectrum (400 MHz, CD_3OD) of ilyolactone A (**1**) 46
Figure S21 HMBC spectrum (400 MHz, CD_3OD) of ilyolactone A (**1**) 47
Figure S22 ROESY spectrum (400 MHz, CD_3OD) of ilyolactone A (**1**) 47
Figure S23 ESIMS spectrum of ilyolactone A (**1**) 48
Figure S24 IR spectrum of ilyolactone A (**1**) 48
Figure S25 HRESIMS spectrum of ilyolactone A (**1**) 49
Figure S26 UV spectrum of ilyolactone A (**1**) 50
Figure S27 Optical rotation data of ilyolactone A (**1**) 51
Figure S28 ^{1}H NMR spectrum (400 MHz, DMSO-d_6) of ilyolactone B (**2**) 52
Figure S29 ^{13}C NMR spectrum (400 MHz, DMSO-d_6) of ilyolactone B (**2**) 52
Figure S30 HSQC spectrum (400 MHz, DMSO-d_6) of ilyolactone B (**2**) 53
Figure S31 ^{1}H-^{1}H COSY spectrum (400 MHz, DMSO-d_6) of ilyolactone B (**2**) 53
Figure S32 HMBC spectrum (400 MHz, DMSO-d_6) of ilyolactone B (**2**) 54
Figure S33 ROESY spectrum (400 MHz, DMSO-d_6) of ilyolactone B (**2**) 54
Figure S34 ESIMS spectrum of ilyolactone B (**2**) 55
Figure S35 IR spectrum of ilyolactone B (**2**) 55
Figure S36 HRESIMS spectrum of ilyolactone B (**2**) 56
Figure S37 UV spectrum of ilyolactone B (**2**) 57
Figure S38 Optical rotation data of ilyolactone B (**2**) 58
Figure S39 ^{1}H NMR spectrum (400 MHz, CD_3OD) of ilyolactone C (**3**) 59
Figure S40 ^{13}C NMR spectrum (100 MHz, CD_3OD) of ilyolactone C (**3**) 59
Figure S41 HSQC spectrum (400 MHz, CD_3OD) of ilyolactone C (**3**) 60
Figure S42 ^{1}H-^{1}H COSY spectrum (400 MHz, CD_3OD) of ilyolactone C (**3**) 60
Figure S43 HMBC spectrum (400 MHz, CD_3OD) of ilyolactone C (**3**) 61

Figure S44 ROESY spectrum (400 MHz, CD_3OD) of ilyolactone C (**3**) …........…...**61**

Figure S45 ESIMS spectrum of ilyolactone C (**3**) …....…………………………....**62**

Figure S46 IR spectrum of ilyolactone C (**3**) …....…………………………………....**62**

Figure S47 HRESIMS spectrum of ilyolactone C (**3**) …....……………...………....**63**

Figure S48 UV spectrum of ilyolactone C (**3**) …....…………………...………....**64**

Figure S49 Optical rotation data of ilyolactone C (**3**) …....……………...………....**65**

Figure S50 ^{1}H NMR spectrum (400 MHz, DMSO-d_6) of ilyolactone D (**4**) …....……**66**

Figure S51 ^{13}C NMR spectrum (100 MHz, DMSO-d_6) of ilyolactone D (**4**) …....……**66**

Figure S52 HSQC spectrum (400 MHz, DMSO-d_6) of ilyolactone D (**4**) …....……**67**

Figure S53 ^{1}H-^{1}H COSY spectrum (400 MHz, DMSO-d_6) of ilyolactone D (**4**) ….......**67**

Figure S54 HMBC spectrum of (400 MHz, DMSO-d_6) ilyolactone D (**4**) …......…**68**

Figure S55 ROESY spectrum of (400 MHz, DMSO-d_6) ilyolactone D (**4**) …......…**68**

Figure S56 ESIMS spectrum of ilyolactone D (**4**) …...……**69**

Figure S57 IR spectrum of ilyolactone D (**4**) …..............................……………**69**

Figure S58 HRESIMS spectrum of ilyolactone D (**4**) ….....................................…**70**

Figure S59 UV spectrum of ilyolactone D (**4**) …..……**71**

Figure S60 Optical rotation data of ilyolactone D (**4**) …...................................……**72**

Figure S61 ^{1}H NMR spectrum (400 MHz, DMSO-d_6) of ilyolactone E (**5**) ……..**73**

Figure S62 ^{13}C NMR spectrum (400 MHz, DMSO-d_6) of ilyolactone E (**5**) …….**73**

Figure S63 HSQC spectrum (400 MHz, DMSO-d_6) of ilyolactone E (**5**) ……….**74**

Figure S64 ^{1}H-^{1}H COSY spectrum (400 MHz, DMSO-d_6) of ilyolactone E (**5**)....**74**

Figure S65 HMBC spectrum (400 MHz, DMSO-d_6) of ilyolactone E (**5**) ……….**75**

Figure S66 ROESY spectrum (400 MHz, DMSO-d_6) of ilyolactone E (**5**) …....…**75**

Figure S67 ESIMS spectrum of ilyolactone E (**5**) ………………………………**76**

Figure S68 IR spectrum of ilyolactone E (**5**) ……………………………………**76**

Figure S69 HRESIMS spectrum of ilyolactone E (**5**) ………………...…………**77**

Figure S70 UV spectrum of ilyolactone E (**5**) ……………………...……………**78**

Figure S71 Optical rotation data of ilyolactone E (**5**) ……………………...…**79**

Figure S72 ^{1}H NMR spectrum (400 MHz, DMSO-d_6) of ilyolactone F(**6**) …......…**80**

Figure S73 ^{13}C NMR spectrum (400 MHz, DMSO-d_6) of ilyolactone F (**6**) ……......**80**

Figure S74 HSQC spectrum (400 MHz, DMSO-d_6) of ilyolactone F (**6**) ……......….**81**

Figure S75 ^{1}H-^{1}H COSY spectrum (400 MHz, DMSO-d_6) of ilyolactone F (**6**) ….**81**

Figure S76 HMBC spectrum (400 MHz, DMSO-d_6) of ilyolactone F (**6**) ………...**82**

Figure S77 ROESY spectrum (400 MHz, DMSO-d_6) of ilyolactone F (**6**) ……..….**82**

Figure S78 ESIMS spectrum of ilyolactone F (**6**) ……………………….....….**83**

Figure S79 IR spectrum of ilyolactone F (**6**) ……………………………....….**83**

Figure S80 HRESIMS spectrum of ilyolactone F (**6**) ……………………...….**84**

Figure S81 UV spectrum of ilyolactone F (**6**) ……………………..........….**85**

Figure S82 Optical rotation data of ilyolactone F (**6**) ……………………...….**86**

Figure S83 ^{1}H NMR spectrum (400 MHz, CD_3OD) of ilyolactone G (**7**) ……...**87**

Figure S84 ^{13}C NMR spectrum (400 MHz, CD_3OD) of ilyolactone G (**7**) …….**87**

Figure S85 HSQC spectrum (400 MHz, CD_3OD) of ilyolactone G (**7**) ……….**88**

Figure S86 ^{1}H-^{1}H COSY spectrum (400 MHz, CD_3OD) of ilyolactone G (**7**) ...**88**

Figure S87 HMBC spectrum (400 MHz, CD_3OD) of ilyolactone G (**7**) …....….**89**

Figure S88 ESIMS spectrum of ilyolactone G (**7**) …………………….....….**90**

Figure S89 IR spectrum of ilyolactone G (**7**) ………………...……......….**90**

Figure S90 HRESIMS spectrum of ilyolactone G (**7**) …………………...….**91**

Figure S91 UV spectrum of ilyolactone G (**7**) ………………………….....….**92**

Figure S92 Optical rotation data of ilyolactone G (**7**) ……………………...….**93**

Figure S93 ^{1}H NMR spectrum (400 MHz, CD_3OD) of ilyolactone H (**8**) ………...**94**

Figure S94 ^{13}C NMR spectrum (400 MHz, CD_3OD) of ilyolactone H (**8**) …...…...**94**

Figure S95 HSQC spectrum (400 MHz, CD_3OD) of ilyolactone H (**8**) …………...**95**

Figure S96 ^{1}H-^{1}H COSY spectrum (400 MHz, CD_3OD) of ilyolactone H (**8**) …...**95**

Figure S97 HMBC spectrum (400 MHz, CD_3OD) of ilyolactone H (**8**) ………...**96**

Figure S98 ROESY spectrum (400 MHz, CD_3OD) of ilyolactone H (**8**) ………...**96**

Figure S99 ESIMS spectrum of ilyolactone H (**8**) …………………….……...**97**

Figure S100 IR spectrum of ilyolactone H (**8**) …………………………………...**97**

Figure S101 HRESIMS spectrum of ilyolactone H (**8**) ………………...……...**98**

Figure S102 UV spectrum of ilyolactone H (**8**) ……………………...……...**99**

Figure S103 Optical rotation data of ilyolactone H (**8**) ………………..……...**100**

Figure S104 ^{1}H NMR spectrum (400 MHz, CD_3OD) of ilyolactone I (**9**) ………...**101**
Figure S105 ^{13}C NMR spectrum (400 MHz, CD_3OD) of ilyolactone I (**9**) ………...**101**
Figure S106 HSQC spectrum (400 MHz, CD_3OD) of ilyolactone I (**9**) …………...**102**
Figure S107 ^{1}H-^{1}H COSY spectrum (400 MHz, CD_3OD) of ilyolactone I (**9**) ……**102**
Figure S108 HMBC spectrum (400 MHz, CD_3OD) of ilyolactone I (**9**) ……..…...**103**
Figure S109 ROESY spectrum (400 MHz, CD_3OD) of ilyolactone I (**9**) ………...**103**
Figure S110 ESIMS spectrum of ilyolactone I (**9**) ……………...………..……...**104**
Figure S111 IR spectrum of ilyolactone I (**9**) ……………………………………....**104**
Figure S1112 HRESIMS spectrum of ilyolactone I (**9**) …………..………………....**105**
Figure S113 UV spectrum of ilyolactone I (**9**) ………………...………………....**106**
Figure S114 Optical rotation data of ilyolactone I (**9**) ………………...……......**107**
Table S8 Energies of configurations **3**–**7** at B3LYP/6-311G (d, p) in methanol......**108**
Table S9 Standard orientations of the conformers of **3**–**7** at mPW1PW91/6-311G (2d, p) in gas phase...**112**

Table S1 The inhibitory effects of the endophytic fungi extract on tumor cells

NO.	Inhibition rate (%)		NO.	Inhibition rate (%)	
	HCT116	MDA-MB-231		HCT116	MDA-MB-231
**1-10**	85.77 %	84.75 %	**361-370**	77.83 %	89.79 %
**11-20**	34.76 %	25.91 %	**371-380**	42.84 %	20.86 %
**21-30**	77.93 %	88.82 %	**381-390**	65.78 %	62.83 %
**31-40**	37.78 %	21.91 %	**391-400**	46.88 %	39.78 %
**41-50**	75.81 %	84.91 %	**401-410**	20.80 %	31.90 %
**51-60**	70.87 %	71.83 %	**411-420**	30.83 %	22.81 %
**61-70**	68.82 %	62.90 %	**421-430**	29.90 %	30.92 %
**71-80**	86.77 %	74.80 %	**431-440**	18.88 %	37.80 %
**81-90**	25.93 %	10.93 %	**441-450**	25.88 %	42.79 %
**91-100**	54.90 %	66.85 %	**451-460**	23.89 %	8.81 %
**101-110**	26.87 %	19.92 %	**461-470**	8.90 %	20.84 %
**111-120**	89.85 %	86.78 %	**471-480**	84.77 %	71.87 %
**121-130**	13.90 %	20.82 %	**481-490**	50.84 %	3.76 %
**131-140**	35.80 %	39.84 %	**491-500**	42.79 %	11.79 %
**141-150**	68.93 %	50.93 %	**501-510**	31.92 %	50.92 %
**151-160**	61.90 %	50.93 %	**511-520**	60.86 %	63.76 %
**161-170**	52.88 %	72.91 %	**521-530**	76.77 %	74.75 %
**171-180**	46.85 %	27.85 %	**531-540**	54.93 %	61.82 %
**181-190**	49.89 %	38.87 %	**541-550**	51.77 %	69.82 %
**191-200**	91.83 %	84.86 %	**551-560**	9.92 %	25.78 %
**201-210**	74.83 %	79.89 %	**561-570**	29.83 %	42.90 %
**211-220**	1.75 %	2.81 %	**571-580**	81.75 %	90.78 %
**221-230**	14.93 %	14.75 %	**581-590**	78.84 %	90.82 %
**231-240**	53.93 %	55.81 %	**591-600**	72.76 %	89.91 %
**241-250**	40.86 %	41.91 %	**601-610**	48.82 %	50.75 %
**251-260**	17.89 %	13.80	**611-620**	31.84 %	33.78 %
**261-270**	70.89 %	70.83 %	**621-630**	70.88 %	61.88 %
**271-280**	16.93 %	11.81 %	**631-640**	67.77 %	77.83 %
**281-290**	68.83 %	53.90 %	**641-650**	63.78 %	52.87 %
**291-300**	31.86 %	19.85 %	**651-660**	36.76 %	17.77 %
**301-310**	27.88 %	13.90 %	**661-670**	68.81 %	76.76 %
**311-320**	12.89 %	9.92 %	**671-680**	75.79 %	74.82 %
**321-330**	80.88 %	81.89 %	**681-690**	10.82 %	25.83 %
**331-340**	40.78 %	34.93 %	**691-700**	7.80 %	14.92 %
**341-350**	66.84 %	64.92 %	**701-710**	95.86 %	91.81 %
**351-360**	67.80 %	58.86 %	**711-720**	27.85 %	18.83 %

NO.	Inhibition rate (%)		NO.	Inhibition rate (%)	
	HCT116	MDA-MB-231		HCT116	MDA-MB-231
**721-730**	64.89 %	75.85 %	**1091-1100**	20.90 %	47.86 %
**731-740**	42.91 %	22.82 %	**1111-1120**	21.91 %	29.82 %
**741-750**	40.82 %	46.93 %	**1121-1130**	23.78 %	16.88 %
**751-760**	30.75 %	8.84 %	**1131-1140**	73.91 %	90.82 %
**761-770**	78.87 %	74.80 %	**1141-1150**	24.75 %	12.83 %
**771-780**	67.78 %	57.85 %	**1151-1160**	6.77 %	4.79 %%
**781-790**	5.86 %	20.78 %	**1161-1170**	16.84 %	20.89 %
**791-800**	86.84 %	81.86 %	**1171-1180**	93.77 %	74.86 %
**801-810**	35.79 %	27.91 %	**1181-1190**	10.90 %	38.88 %
**811-820**	37.85 %	35.78 %	**1191-1200**	22.81 %	17.93 %
**821-830**	37.91 %	35.87 %	**1201-1210**	60.79 %	58.92 %
**831-840**	33.82 %	6.80 %	**1211-1220**	61.78 %	51.93 %
**841-850**	47.92 %	43.86 %	**1221-1230**	20.76 %	5.76 %
**851-860**	23.84 %	3.89 %	**1231-1240**	35.89 %	42.80 %
**861-870**	80.78 %	88.77 %	**1241-1250**	80.82 %	88.86 %
**871-880**	86.75 %	81.87 %	**1251-1260**	21.88 %	9.91 %
**881-890**	15.90 %	0.77 %	**1261-1270**	83.85 %	82.79 %
**891-900**	12.90 %	10.93 %	**1271-1280**	44.78 %	35.93 %
**901-910**	59.92 %	52.93 %	**1281-1290**	64.85 %	55.83 %
**911-920**	53.83 %	59.90 %	**1291-1300**	26.85 %	38.90 %
**921-930**	9.92 %	21.91 %	**1301-1310**	46.76 %	20.88 %
**931-940**	11.83 %	13.89 %	**1311-1320**	77.83 %	91.90 %
**941-950**	92.80 %	83.93 %	**1321-1330**	38.89 %	20.85 %
**951-960**	33.87 %	44.75 %	**1331-1340**	9.85 %	13.78 %
**961-970**	48.88 %	53.79 %	**1341-1350**	77.76 %	54.89 %
**971-980**	3.93 %	16.84 %	**1351-1360**	25.93 %	38.78 %
**981-990**	63.80 %	72.88 %	**1361-1370**	21.85 %	4.89 %
**991-1000**	69.92 %	54.80 %	**1371-1380**	82.75 %	76.88 %
**1001-1010**	13.83 %	10.85 %	**1381-1390**	47.85 %	37.80 %
**1011-1020**	93.83 %	84.83 %	**1391-1400**	75.87 %	62.77 %
**1021-1030**	35.88 %	3.88 %	**1401-1410**	26.86 %	12.91 %
**1031-1040**	4.76 %	0.82 %	**1411-1420**	77.88 %	93.88 %
**1041-1050**	78.85 %	91.82 %	**1421-1430**	29.89 %	36.81 %
**1051-1060**	11.41 %	10.76 %	**1431-1440**	51.88 %	52.84 %
**1061-1070**	41.91 %	27.89 %	**1441-1450**	48.86 %	44.84 %
**1071-1080**	92.84 %	73.79 %	**1451-1460**	45.76 %	49.79 %
**1081-1090**	38.90 %	1.84 %	**1461-1470**	87.92 %	71.83 %
**1101-1110**	43.83 %	26.83 %	**1471-1480**	28.87 %	7.75 %

NO.	Inhibition rate (%)		NO.	Inhibition rate (%)	
	HCT116	MDA-MB-231		HCT116	MDA-MB-231
**1481-1490**	61.77 %	56.87 %	**1551-1560**	20.76 %	21.80 %
**1491-1500**	17.85 %	17.79 %	**1561-1570**	22.76 %	50.88 %
**1501-1510**	32.87 %	20.86 %	**1571-1580**	35.88 %	44.90 %
**1511-1520**	85.76 %	90.76 %	**1581-1590**	75.92 %	91.82 %
**1521-1530**	27.93 %	12.77 %	**1591-1600**	14.75 %	27.81 %
**1531-1540**	59.81 %	76.84 %	**1601-1609**	67.86 %	69.93 %
**1541-1550**	56.78 %	58.77 %			

Note: All isolated endophytic fungi on PDA medium at 28 °C for 7 days. Then the agar plugs were inoculated into autoclaved medium in Erlenmeyer flasks (250 mL) containing rice medium (50 mL deionized water and 45 g rice, sterilized in 121 °C for 30 min). After the strain was fermented at 28 28 °C for 28 days, the rice fermented materials were obtained, extracted with EtOAc, and subsequently dissolved in DMSO. Ten strains were in a group. The inhibitory effects of the extracts (40 mg/mL) on cancer cells (HCT116 and MDA-MB-231) were evaluated using the SRB assay. Strain FL-710 was in group 701-710 showing potent growth inhibitory activity.

Figure S1 UPLC-MS profiles of ethyl acetate extracts of *Ilyonectria* sp. FL-710

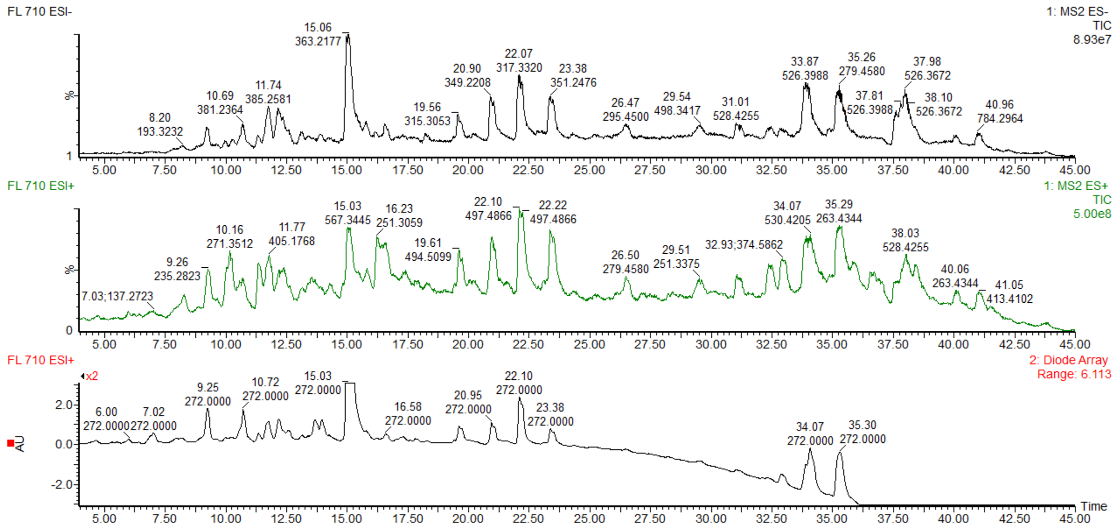

Detailed Structural Elucidation of 2−9

Ilyolactone B (**2**) was obtained as colourless crystals, whose molecular formula was established as $C_{19}H_{23}ClO_8$ (8 degrees of unsaturation) based on HRESIMS ([M+Na]$^+$, 414.10715, calcd 437.09737) and supported by ^{1}H and ^{13}C NMR spectroscopic data (Table S2). The IR spectrum showed absorption bands at 2999, 2853, 1745, 1384, 1241, 1092, and 663 cm^{-1}, which indicated the presence of hydroxyl, carbonyl, and double bond functional groups. The UV spectrum exhibited maximum absorption bands at λ_{max} 217, 250, and 315 nm, suggesting that **2** contained a conjugated double bond system. Comparing the 1D and 2D NMR data of **2** and **1** revealed that they share the same planar structure (Figure S2 and Table S2). The absolute configuration of **2** was determined to be 2*R*, 4*R*, 7*R*, and 8*R* by the single-crystal X-ray diffraction analysis (Cu Kα). Therefore, the structure of **2** was then elucidated (Figure 1B) and named ilyolactone B.

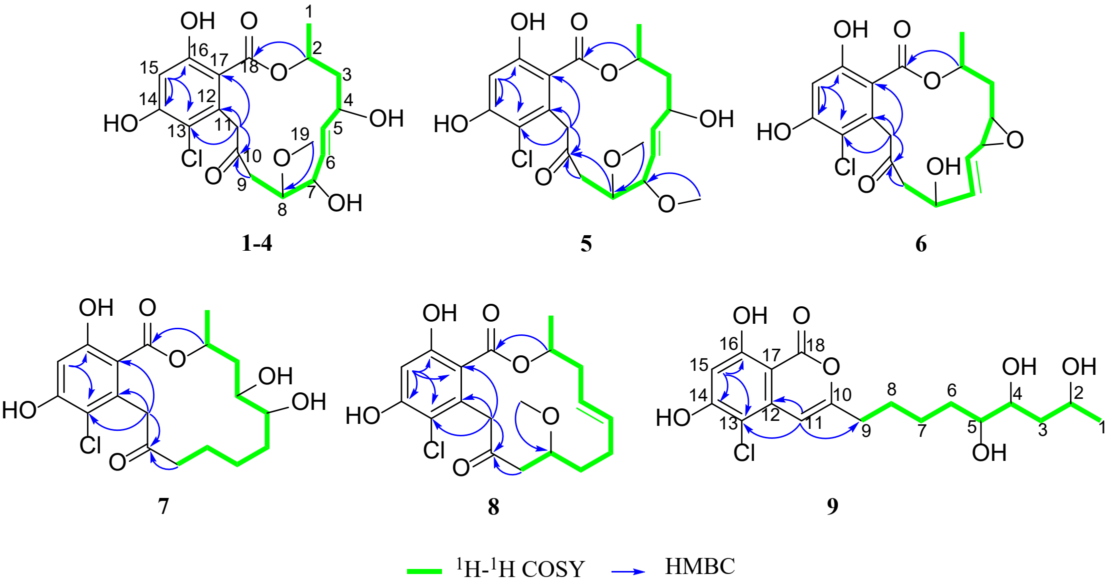

Figure S2 Key ^{1}H-^{1}H COSY and HMBC correlations of RALs (**1**−**9**)

Ilyolactone C (**3**) was obtained as white solid, whose molecular formula was established as $C_{19}H_{23}ClO_8$ (8 degrees of unsaturation) based on HRESIMS ([M+Na]$^+$, 437.09738, calcd 437.09737) and supported by ^{1}H and ^{13}C NMR spectroscopic data (Table S2). The IR spectrum showed absorption bands at 2896, 2833, 1384, 1241, 1117, 1022, and 844 cm^{-1}, which indicated the presence of hydroxyl, carbonyl, and double bond functional groups. The UV spectrum exhibited maximum absorption bands at λ_{max} 220, 264, and 314 nm, suggesting that **3** contained a conjugated double bond system.

Comparing the 1D and 2D NMR data of **3** and **1** revealed that they had the same planar structure (Figure S2 and Table S2).

Ilyolactone D (**4**) was obtained as white solid, whose molecular formula was established as $C_{19}H_{23}ClO_8$ (8 degrees of unsaturation) based on HRESIMS ($[M+Na]^+$, 437.09755, calcd 437.09737) and supported by ^1H and ^{13}C NMR spectroscopic data (Table S2). The IR spectrum showed absorption bands at 2923, 2853, 1736, 1653, 1384, 1241, 1093, and 664 cm^{-1}, which indicated the presence of hydroxyl, carbonyl, and double bond functional groups. The UV spectrum exhibited maximum absorption bands at λ_{max} 220, 265, and 314 nm, suggesting that **4** contained a conjugated double bond system. Comparing the 1D and 2D NMR data of **4** and **1** revealed that they had the same planar structure (Figure S2 and Table S2).

Ilyolactone E (**5**) was obtained as white solid, whose molecular formula was established as $C_{20}H_{25}ClO_8$ (8 degrees of unsaturation) based on HRESIMS ($[M+Na]^+$, 451.11302, calcd 405.07115) and supported by ^1H and ^{13}C NMR spectroscopic data (Table S3). The IR spectrum showed absorption bands at 2922, 1721, 1646, 1356, 1084, 1001, and 773 cm^{-1}, which indicated the presence of hydroxyl, carbonyl, and double bond functional groups. The UV spectrum exhibited maximum absorption bands at λ_{max} 220, 250, 262, and 315 nm, suggesting that **5** contained a conjugated double bond system. Comparing the 1D and 2D NMR data of **5** and **4** revealed that they had the similar planar structure (Figure S2 and Table S3) with the difference in that a methoxy group in C-7 of **5**.

We tried various approaches to determine the absolute configurations of **3**–**5**. It was worth mentioning that comparing the calculated and experimental ECD spectra and NMR data has been demonstrated to be a powerful method to determine the spatial configurations of natural products in recent years. Compounds **3**–**5** contained four stereocenters, and their possible absolute configurations were listed in Figure S3. Then, calculated NMR and ECD spectra of **3**–**5** were generated using the time-dependent density functional theory (TD-DFT) method in Gaussian 09. Fortunately, the calculated data of ^{13}C NMR and ECD spectra (Figures S4 and S5) were consistent for their configurations determination, and the absolute configurations of these compounds were assigned as 2*R*, 4*R*, 7*S*, 8*S* for **3**, 2*R*, 4*R*, 7*R*, 8*S* for **4**, and 2*R*, 4*R*, 7*R*, and 8*S* for **5**.

Ilyolactone F (**6**) was obtained as white solid, whose molecular formula was established as $C_{18}H_{19}ClO_7$ (9 degrees of unsaturation) based on HRESIMS ($[M+Na]^+$, 382.08238, calcd 405.07115) and supported by ^1H and ^{13}C NMR spectroscopic data

(Table S3). The IR spectrum showed absorption bands at 2922, 2853, 1721, 1355, 1240, 1062, and 772 cm^{-1}, which indicated the presence of hydroxyl, carbonyl, and double bond functional groups. The UV spectrum exhibited maximum absorption bands at λ_{max} 250 and 316 nm, suggesting that **6** contained a conjugated double bond system. Further analysis of the 1D and 2D NMR data of **6** (Figure S2 and Table S3) revealed that its planar structure is similar to that of the known radicicol (**12**). The main differences were the reduction of the C-8−C-9 double bond and the replacement of the C-8 position with an oxygen atom in **6**, which was confirmed by mass data and chemical shift values.

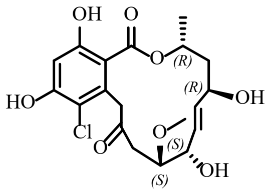
3b

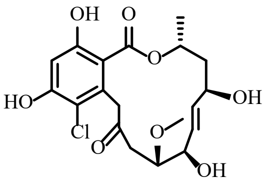
3c

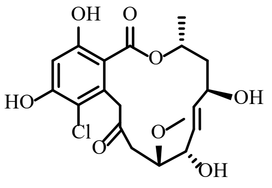
4b

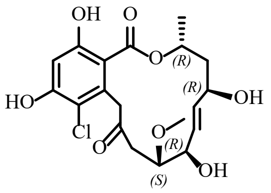
4c

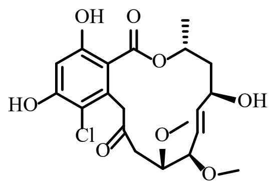
5a

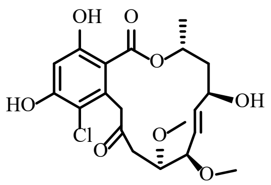
5b

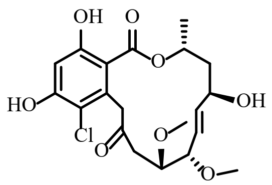
5c

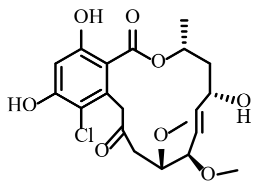
5d

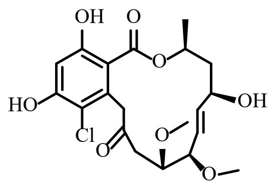
5e

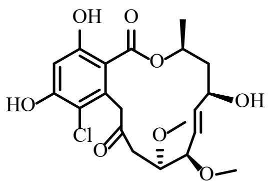
5f

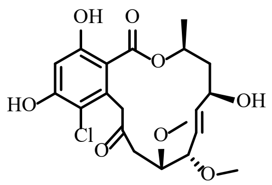
5g

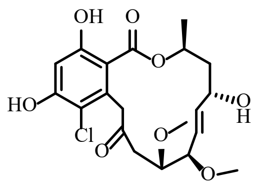
5h

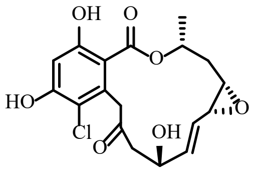
6a

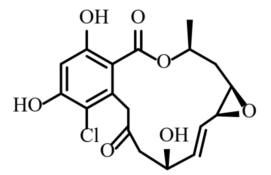
6b

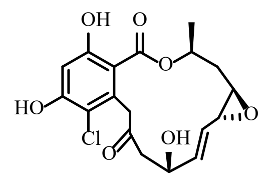
6c

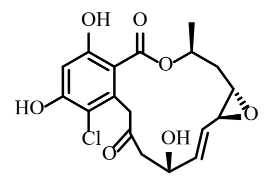
6d

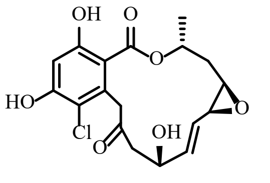
6e

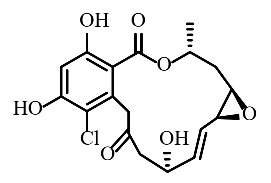
6f

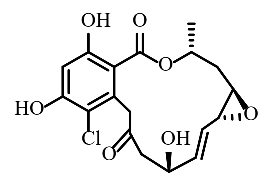
6g

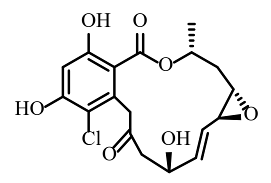
6h

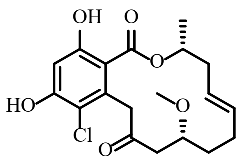
7a

Figure S3 All possible configurations of **3-7**

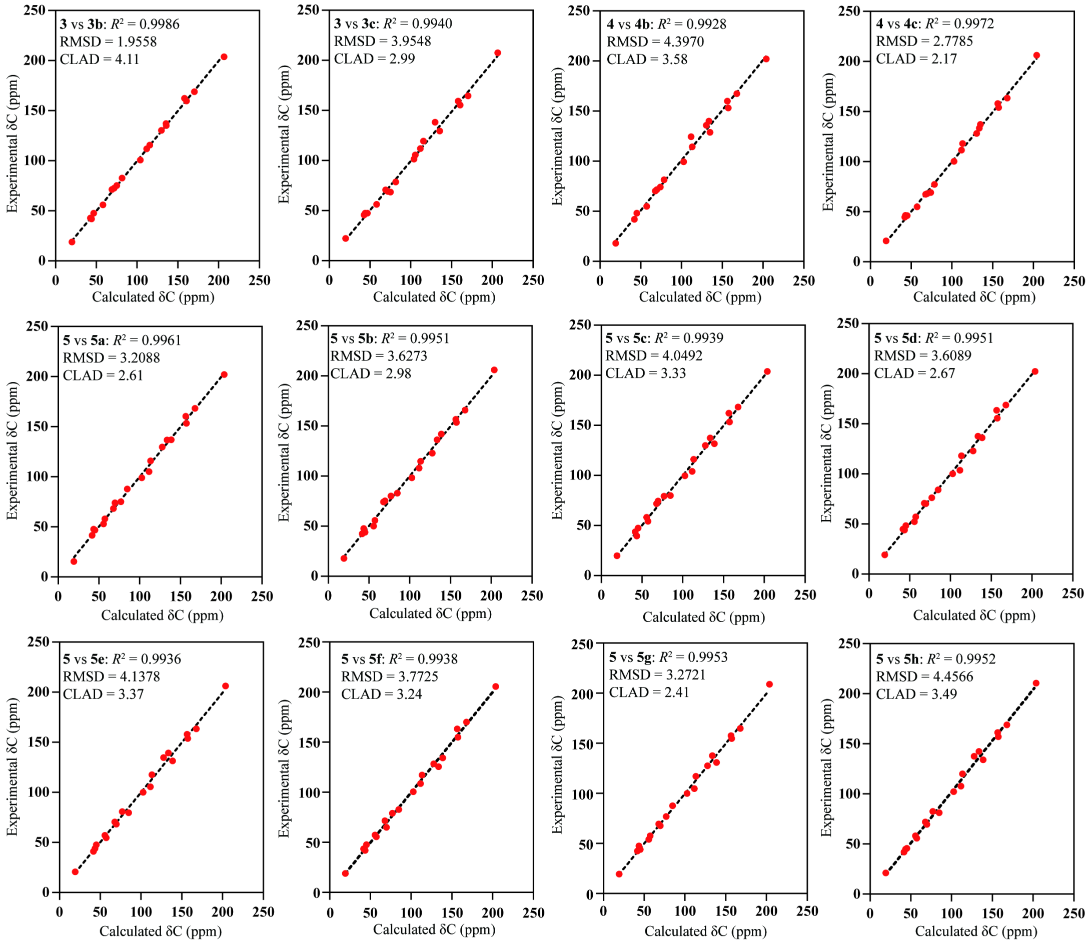

Figure S4 Experimental and calculated ^{13}C-NMR of RALs (**3**−**5**)

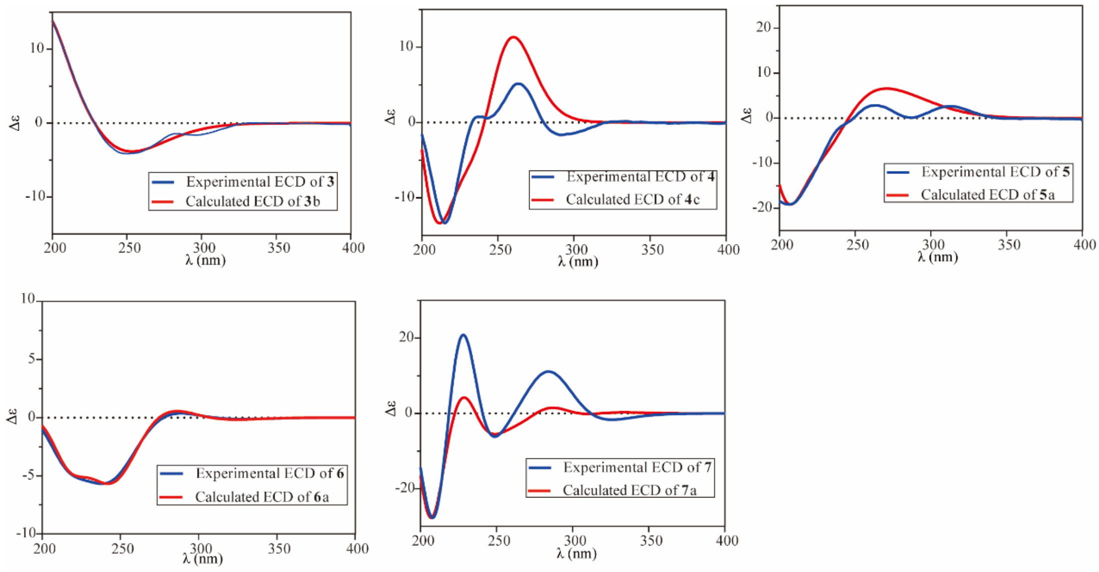

Figure S5 Experimental and calculated ECD spectra of RALs (**3**−**7**)

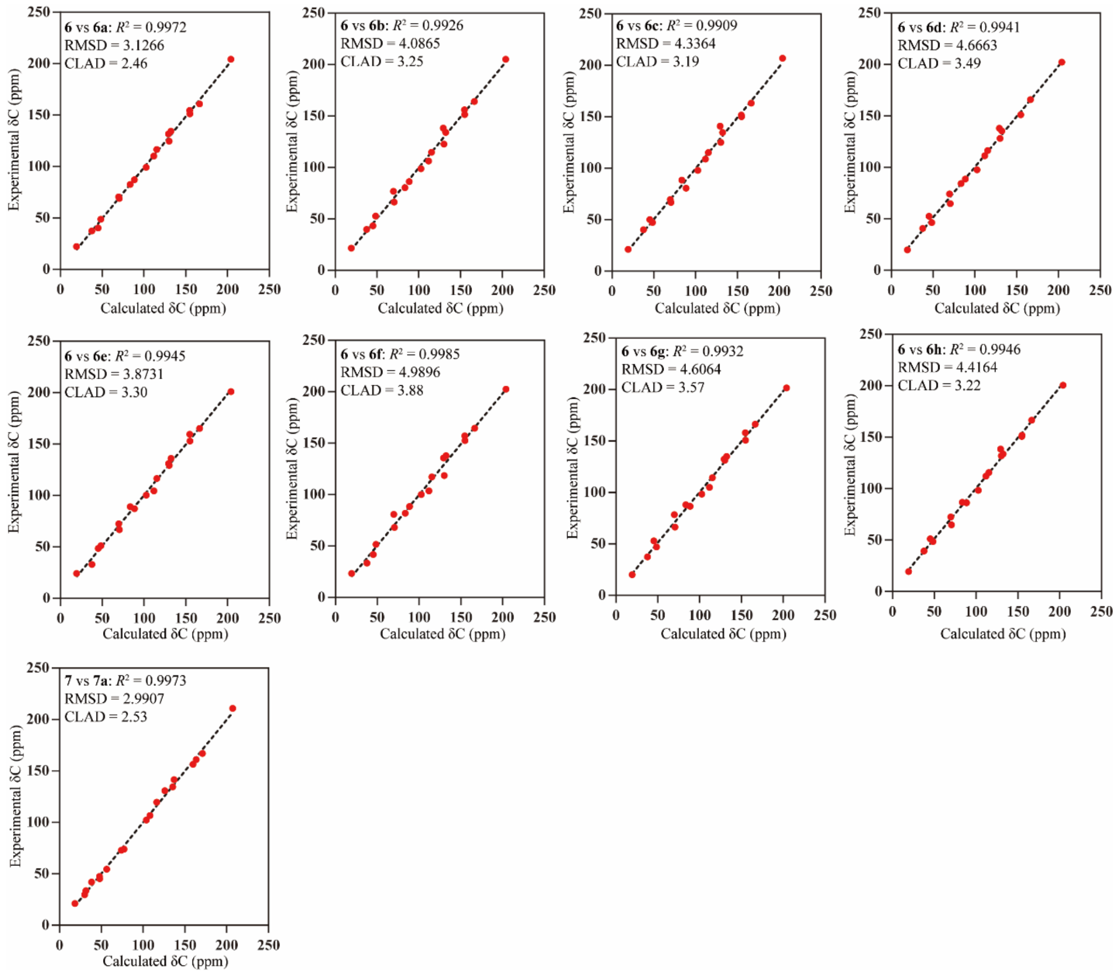

Figure S6 Experimental and calculated ^{13}C-NMR of RALs (**6** and **7**)

Thus, the planar structure of **6** was determined. The double bone was assigned as *E* configuration, and the absolute configurations of **6** were confirmed as 2*R*, 4*S*, 7*R*, 8*S* based on the calculated ECD spectra and NMR data (Figures S3, S5, and, S6).

Ilyolactone G (**7**) was obtained as white solid, whose molecular formula was established as $C_{18}H_{23}ClO_7$ (7 degrees of unsaturation) based on HRESIMS ($[M+Na]^+$, 409.10239, calcd 409.10245) and supported by ^{1}H and ^{13}C NMR spectroscopic data (Table S3). Further analysis of the 1D and 2D NMR data of **7** (Figure S2 and Table S3) revealed that its planar structure is similar to that of the known radicicol B. The main differences were the reduction of the C-6–C-7 and C-8–C-9 double bond in **7**, which was confirmed by mass data and chemical shift values. Thus, the planar structure of **7** was determined and its absolute configurations were also confirmed as 2*R*, 4*S*, 5*R* based on the calculated ECD spectra and NMR data (Figures S5 and S6).

Ilyolactone H (**8**) was obtained as colourless crystals, whose molecular formula was established as $C_{19}H_{23}ClO_6$ (8 degrees of unsaturation) based on HRESIMS ($[M+Na]^+$,

405.10822, calcd 405.10754) and supported by ^{1}H and ^{13}C NMR spectroscopic data (Table S3). The IR spectrum showed absorption bands at 2925, 2853, 1719, 1571, 1356, 1083, and 663 cm^{-1}, which indicated the presence of hydroxyl, carbonyl, and double bond functional groups. The UV spectrum exhibited maximum absorption bands at λ_{max} 219, 234, 249, 261, and 317 nm, suggesting that **8** contained a conjugated double bond system. Further analysis of the 1D and 2D NMR data of **8** (Figure S2 and Table S3) revealed that its planar structure is similar to that of the known monocillin II. The main differences were the reduction of the C-8−C-9 double bond, the substitution of a chlorine atom at the C-13 position, and the replacement of the C-8 position with a methoxy group in **8**, which was confirmed by mass data and chemical shift values. Thus, the planar structure of **8** was determined and its absolute configurations were also confirmed as 2*R*, 8*R* by the single-crystal X-ray diffraction analysis (Cu Kα) with a Flack parameter of 0.042(14) (Figures 1B and 1C).

Ilyolactone I (**9**) was obtained as colourless crystals, whose molecular formula was established as $C_{18}H_{23}ClO_7$ (7 degrees of unsaturation) based on HRESIMS ($[M+Na]^+$, 409.10239, calcd 409.10245) and supported by ^{1}H and ^{13}C NMR spectroscopic data (Table S4). Further analysis of the 1D and 2D NMR data of **9** (Figure S2) revealed that its planar structure is similar to that of **7**. The main differences were the ring opening at ester bond in **9**, which was confirmed by mass data, chemical shift values, and 2D NMR data. The absolute configurations of **9** was determined as 2*S*, 4*S*, and 5*R* by the single-crystal X-ray diffraction analysis (Cu Kα) with a Flack parameter of 0.023(10) (Figures 1B and 1C).

Known compounds were determined as monorden E (**10**) [1], monocillin III (**11**) [2], radicicol (**12**) [3], monorden C (**13**) [4], *O*-desmethylgreensporone C (**14**) [5], monocillin II (**15**) [6], pochonin D (**16**) [6], radicicol C (**17**) [7], ilyolactone J (**18**) [8], pochonin A (**19**) [9], monocillin I (**20**) [7], radicicol B (**21**) [10], radisicol analogus I (**22**) [10], pochonin G (**23**) [11], and curvularin (**24**) [12] by comparing their NMR and ESIMS data with those reported in the literature. Compounds **1**–**6** represented the first example of RALs with *E* configuration in C-5−C-6 and C-6−C-7.

Table S2 Spectroscopic data for compounds **1–4** (δ in ppm, J in Hz)

position	**1**[a]		**2**[b]		**3**[a]		**4**[b]	
	δ_C, type	δ_H (J in Hz)	δ_C, type	δ_H (J in Hz)	δ_C, type	δ_H (J in Hz)	δ_C, type	δ_H (J in Hz)
1	20.8, CH_3	1.41, d, (6.5)	18.9, CH_3	1.21, d, (6.4)	20.2, CH_3	1.42, d, (6.5)	19.4, CH_3	1.41, d, (6.4)
2	71.3, CH	5.52, overlap	69.9, CH	5.12, m	72.0, CH	5.22, m	69.6, CH	5.28, m
3a	43.2, CH_2	2.20, ddd, (15.5, 8.2, 3.3)	42.1, CH_2	1.91, m	42.7, CH_2	2.20, ddd, (15.2, 8.0, 3.6)	42.1, CH_2	2.07, ddd, (14.2, 7.8, 4.1)
3b		2.03, ddd, (15.5, 7.2, 1.5)		1.77, m		1.91, ddd, (15.2, 8.2, 1.9)		1.95, dd, (14.2, 8.8)
4	70.8, CH	4.33, ddd, (8.3, 3.3, 1.5)	68.0, CH	4.05, dt, (8.2, 3.6)	69.1, CH	4.34, m	67.9, CH	4.19, m
5	136.1, CH	5.49, dd, (15.5, 8.3)	130.9, CH	5.49, dd, (15.4, 8.2)	135.6, CH	5.75, dd, (15.5, 5.5)	130.4, CH	5.46, dd, (15.5, 7.9)
6	131.0, CH	5.67, dd, (15.5, 8.4)	132.9, CH	5.38, dd (15.4, 3.2)	129.9, CH	5.63, dd, (15.5, 6.3)	135.1, CH	5.58, dd, (15.5, 7.6)
7	73.7, CH	4.34, dd, (8.4, 3.3)	68.5, CH	4.34, brs	75.2, CH	4.16, overlap	74.1, CH	3.98, m
8	80.6, CH	3.78, ddd, (9.2, 4.6, 3.3)	76.8, CH	3.66, m	81.5, CH	3.53, m	78.5, CH	3.61, m
9a	43.3, CH_2	2.89, dd, (18.2, 4.6)	40.9, CH_2	2.80, dd, (19.5, 5.9)	44.2, CH_2	2.67, m	42.9, CH_2	2.67, m
9b		2.68, dd, (18.2, 9.2)		2.17, d, (19.5)				2.56, dd, (19.0, 3.5)
10	206.7, C		204.6, C		206.8, C		204.0, C	
11a	47.3, CH_2	4.61, m	45.2, CH_2	3.83, d, (17.4)	46.9, CH_2	4.16, overlap	45.5, CH_2	4.09, d, (17.2)
11b		4.21, m		3.71, d, (17.4)		4.12, overlap		3.92, d, (17.2)
12	109.2, C		112.1, C		111.7, C		111.8, C	
13	116.3, C		113.0, C		115.5, C		113.1, C	
14	162.8, C		156.9, C		160.7, C		157.3, C	
15	104.0, CH	6.48, s	102.8 CH	6.53, s	103.9 CH	6.46, s	102.7, CH	6.69, s
16	159.3, C		156.3, C		158.3, C		156.3, C	
17	136.3, C		133.5, C		135.8, C		133.7, C	
18	171.3, C		167.8, C		170.4, C		167.9, C	
19	58.3, CH_3	3.42, s	56.0, CH_3	3.22, s	58.0, CH_3	3.39, s	57.3, CH_3	3.42, s

[a]Recorded at 400 MHz in CD_3OD. [b]Recorded at 400 MHz in DMSO-d_6.

Table S3 Spectroscopic data for compounds **5–8** (δ in ppm, J in Hz)

position	**5**[a]		**6**[a]		**7**[b]		**8**[b]	
	δ_C, type	δ_H (J in Hz)	δ_C, type	δ_H (J in Hz)	δ_C, type	δ_H (J in Hz)	δ_C, type	δ_H (J in Hz)
1	19.2, CH_3	1.25, d, (6.4)	19.3, CH_3	1.32, d, (8.7)	24.2, CH_3	1.20, d, (6.3)	18.5, CH_3	1.34, d, (6.6)
2	69.7, CH	5.09, m	69.8, CH	5.21, m	65.1, CH	3.96, m	74.0, CH	5.33, overlap
3a	41.8, CH_2	1.91, ddd, (12.1, 7.6, 3.9)	37.7, CH_2	2.18, ddd, (14.8, 4.5, 2.1)	42.0, CH_2	1.94, overlap	38.4, CH_2	2.49, ddd, (12.1, 7.6, 3.9)
3b		1.78, dd, (12.1, 8.2)		1.44, ddd, (14.8, 8.2, 3.4)		1.74, ddd, (14.7, 8.0, 2.9)		2.26, dd, (12.1, 8.2)
4	67.9, CH	4.02, m	70.4, CH	3.21, m	77.8, CH	5.11, m	126.2, CH	5.48, m
5	138.7, CH	5.53, dd, (15.4, 8.4)	88.5, CH	4.22, m	72.1, CH	3.68, m	135.4, CH	5.38, overlap
6a	127.7, CH	5.14, dd, (15.4, 8.8)	130.1, CH	5.90, dd, (15.4, 8.8)	31.9, CH_2	1.53, overlap	30.0, CH_2	2.21, overlap
6b								2.05, m
7a	84.8, CH	3.47, m	129.4, CH	6.01, m	25.2, CH_2	1.66, m	31.7, CH_2	1.79, m
7b						1.53, overlap		1.53, m
8	77.0, CH	3.54, m	83.4, CH	5.02, m	23.6, CH_2	1.94, overlap	77.3, CH	3.66, m
9a	43.5, CH_2	2.54, m	45.2, CH_2	2.78, m	42.6, CH_2	2.57, m	48.1, CH_2	2.72, dd, (18.4, 5.0)
9b				2.38, m				2.54, dd, (18.4, 5.4)
10	203.7, C		204.0, C		210.9, C		207.2, C	
11a	45.1, CH_2	3.93, d, (17.6)	48.5, CH_2	3.91, d, (18.2)	47.2, CH_2	4.51, d, (18.9)	47.8, CH_2	4.22, d, (17.8)
11b		3.80, d, (17.6)		3.72, d, (18.2)		4.42, d, (18.9)		4.01, d, (17.8)
12	111.6, C		111.8, C		111.7, C		108.3, C	
13	113.3, C		115.2, C		115.7, C		116.5, C	
14	157.4, C		154.9, C		160.4, C		159.7, C	
15	102.7, CH	6.50, s	102.5, CH	6.51, s	103.7, CH	6.47, s	104.0, CH	6.45, s
16	156.5, C		154.6, C		158.5, C		163.7, C	
17	133.5, C		132.3, C		135.7, C		137.1, C	
18	167.9, C		166.5, C		170.7, C		171.0, C	
19	57.4, CH_3	3.25, s					56.5, CH_3	3.26, s
20	55.7, CH_3	3.15, s						

[a]Recorded at 400 MHz in DMSO-d_6. [b]Recorded at 400 MHz in CD_3OD.

Table S4 Spectroscopic data for compound **9** in CD_3OD (δ in ppm, J in Hz)

Position	δ_C, type	δ_H type, (J in Hz)	Position	δ_C, type	δ_H, type, (J in Hz)
1	24.5, CH_3	1.22, m	9	34.4, CH_2	2.62, t, (7.4)
2	65.5, CH	4.00, m	10	160.3, C	
3a	33.3, CH_2	1.66, overlap	11	101.6, CH	6.71, s
3b		1.48, overlap	12	99.9, C	
4	72.8, CH	3.68, m	13	107.3, C	
5	76.0, CH	3.44, m	14	163.4, C	
6a	42.4, CH_2	1.66, overlap	15	103.1, CH	6.46, s
6b		1.52, m	16	163.2, C	
7a	26.4, CH_2	1.65, overlap	17	137.7, C	
7b		1.48, overlap	18	167.3, C	
8	28.1, CH_2	1.75, m			

Table S5 Cytotoxicity of RALs (**1**–**24**) against human cancer cell lines

No.	IC_{50} (μM)					
	HCT116	A549	HEPG2	MDA-MB-231	PANC-1	SGC-7901
**1**	54.83 ± 4.02	61.55 ± 1.64	52.40 ± 2.76	64.11 ± 4.11	65.92 ± 2.03	73.70 ± 1.29
**2**	80.59 ± 1.11	74.61 ± 3.00	52.12 ± 1.23	45.71 ± 2.80	81.18 ± 2.57	67.40 ± 2.94
**3**	70.92 ± 0.27	73.57 ± 3.35	85.52 ± 3.88	52.35 ± 4.53	68.53 ± 1.37	78.69 ± 2.96
**4**	67.43 ± 5.34	75.39 ± 2.90	73.27 ± 12.63	45.90 ± 3.92	74.79 ± 0.58	74.49 ± 15.03
**5**	72.04 ± 3.10	62.93 ± 1.85	73.31 ± 0.96	51.37 ± 0.84	77.58 ± 1.01	77.25 ± 1.23
**6**	77.80 ± 10.38	71.30 ± 0.91	79.71 ± 2.09	82.30 ± 4.56	78.02 ± 3.56	75.39 ± 4.05
**7**	12.39 ± 1.16	32.75 ± 3.05	54.13 ± 1.35	5.23 ± 0.49	12.10 ± 1.14	10.59 ± 0.52
**8**	11.76 ± 3.52	5.74 ± 2.85	10.27 ± 2.36	8.08 ± 1.69	11.48 ± 0.63	16.75 ± 0.55
**9**	44.69 ± 4.23	42.82 ± 0.60	47.44 ± 1.25	44.25 ± 3.05	47.22 ± 1.72	43.49 ± 1.90
**10**	16.61 ± 5.27	28.46 ± 1.37	45.31 ± 4.47	26.33 ± 4.44	42.49 ± 0.69	15.72 ± 1.89
**11**	26.19 ± 0.88	43.09 ± 3.90	21.93 ± 2.94	42.48 ± 1.49	42.12 ± 3.10	52.41 ± 3.67
**12**	8.39 ± 4.55	8.29 ± 1.19	13.16 ± 0.61	7.67 ± 1.14	7.82 ± 0.36	8.19 ± 1.11
**13**	10.34 ± 1.44	7.67 ± 0.90	7.97 ± 1.59	6.80 ± 1.02	9.09 ± 1.76	10.40 ± 0.66
**14**	53.15 ± 0.10	60.87 ± 0.36	69.77 ± 2.42	43.53 ± 2.34	50.59 ± 0.23	64.16 ± 1.01
**15**	15.06 ± 1.16	27.36 ± 0.30	20.77 ± 0.43	5.09 ± 1.25	7.26 ± 2.01	8.90 ± 0.80
**16**	4.16 ± 0.90	3.72 ± 2.00	7.55 ± 1.46	2.66 ± 0.34	4.57 ± 3.25	4.70 ± 0.32
**17**	6.44 ± 0.81	27.98 ± 2.45	18.14 ± 2.35	8.65 ± 0.74	17.97 ± 2.11	18.29 ± 3.52
**18**	5.96 ± 0.35	8.61 ± 0.21	6.57 ± 0.81	7.98 ± 0.31	8.25 ± 1.78	15.67 ± 2.91
**19**	63.37 ± 4.29	57.15 ± 1.48	69.79 ± 2.27	47.61 ± 4.29	64.22 ± 0.73	73.37 ± 2.70
**20**	33.53 ± 2.11	38.76 ± 3.67	30.79 ± 0.45	24.26 ± 0.73	42.60 ± 1.03	53.39 ± 0.14
**21**	60.87 ± 0.36	64.16 ± 1.01	62.65 ± 2.89	47.66 ± 4.28	74.55 ± 2.80	79.85 ± 2.06
**22**	16.62 ± 2.17	39.02 ± 0.65	17.46 ± 1.41	15.97 ± 0.87	23.65 ± 2.42	29.02 ± 2.44
**23**	16.67 ± 0.27	58.07 ± 4.71	41.71 ± 2.46	6.22 ± 0.99	5.18 ± 0.40	7.36 ± 0.36
**24**	9.26 ± 2.69	9.55 ± 2.68	10.21 ± 1.19	13.03 ± 1.19	11.19 ± 5.21	7.76 ± 1.45
cisplatin	15.15 ± 2.33	21.29 ± 0.74	24.31 ± 0.51	51.24 ± 3.44	66.34 ± 3.41	20.31 ± 0.88

Data represented the mean ± standard deviation (SD) of at least three independent experiments, and each experiment was performed in triplicate. Cisplatin was used as positive control.

Table S6 Cytotoxicity of compounds against human cancer cell lines

	IC50 (μM)			
	24 h		48 h	
	MDA-MB-231	4T1	MDA-MB-231	4T1
Pochonin D (PoD, **16**)	6.31 ± 0.9	8.94 ± 1.65	2.55 ± 1.30	6.34 ± 0.25
5-FU	220.71 ± 16.10	240.33 ±18.48	146.08 ± 24.19	112.72 ±8.57
Cis-platinum	19.93 ± 0.95	16.81 ± 0.36	11.03 ±0.53	13.54 ±1.16
Cyclophosphamide	30.11 ± 1.94	33.41 ± 0.95	21.34 ±0.28	24.97 ±1.43

Data represented the mean ± standard deviation (SD) of at least three independent experiments, and each experiment was performed in triplicate.

Table S7 Cytotoxicity of pochonin D against breast cancer cell lines

	IC50 (μM)					
	MDA-MB-231	4T1	BT549	SUM159	MDA-MB-157	MCF-10A
**12 h**	18.15 ± 2.90	21.73 ± 0.93	26.90 ± 4.13	26.48 ± 2.29	25.18 ± 1.86	73.50 ± 2.64
**24 h**	6.31 ± 0.90	8.94 ± 1.65	17.26 ± 2.68	12.73 ± 1.51	11.34 ± 1.47	46.65 ± 3.74
**48 h**	2.55 ± 1.30	6.34 ± 0.25	8.91 ± 1.62	7.25 ± 1.22	8.08 ± 0.09	35.39 ± 1.56
**72 h**	1.08 ± 0.28	3.34 ± 0.93	5.57 ± 0.41	5.82 ± 2.14	8.68 ± 0.31	26.55 ± 2.75

Data represented the mean ± standard deviation (SD) of at least three independent experiments, and each experiment was performed in triplicate.

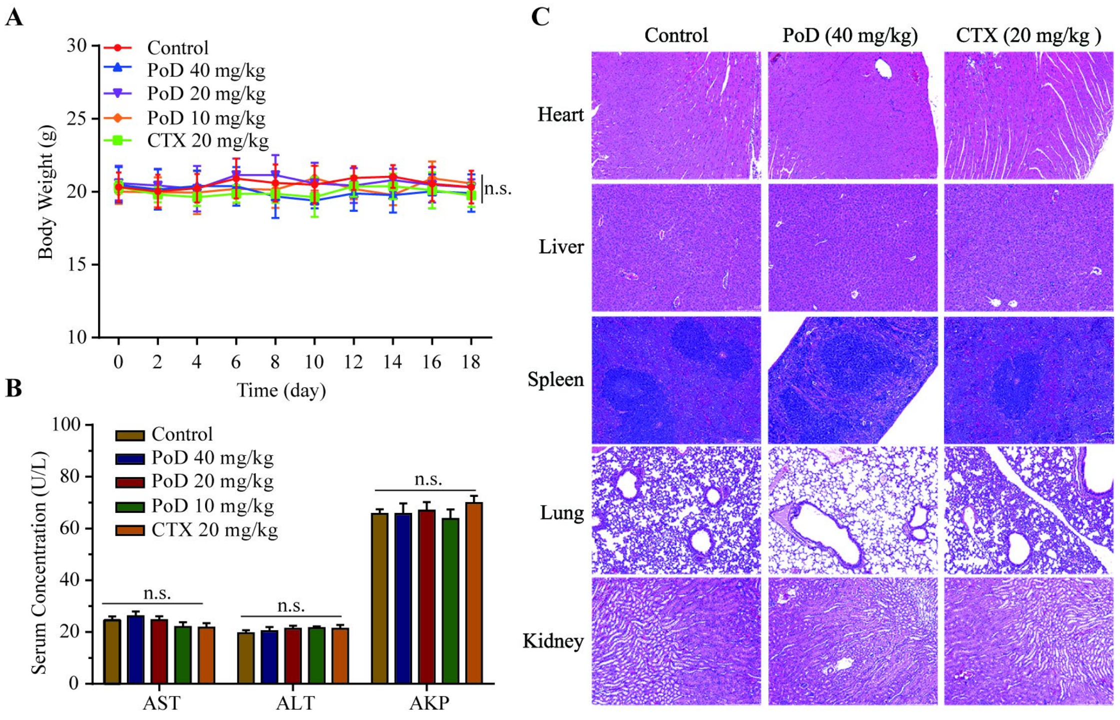

Figure S7 Analysis of potential side effects for treatment of PoD. (A) The change curves of body weights of BALB/c bearing 4T1 xenograft tumors. (B) The evaluation of serum AST, ALT, and AKP for vehicle- and various concentrations of the PoD and CTX-treated groups. (C)

Representative hematoxylin–eosin staining of heart, kidney, spleen, lung, and liver from vehicle- and various concentrations of PoD and CTX-treated groups.

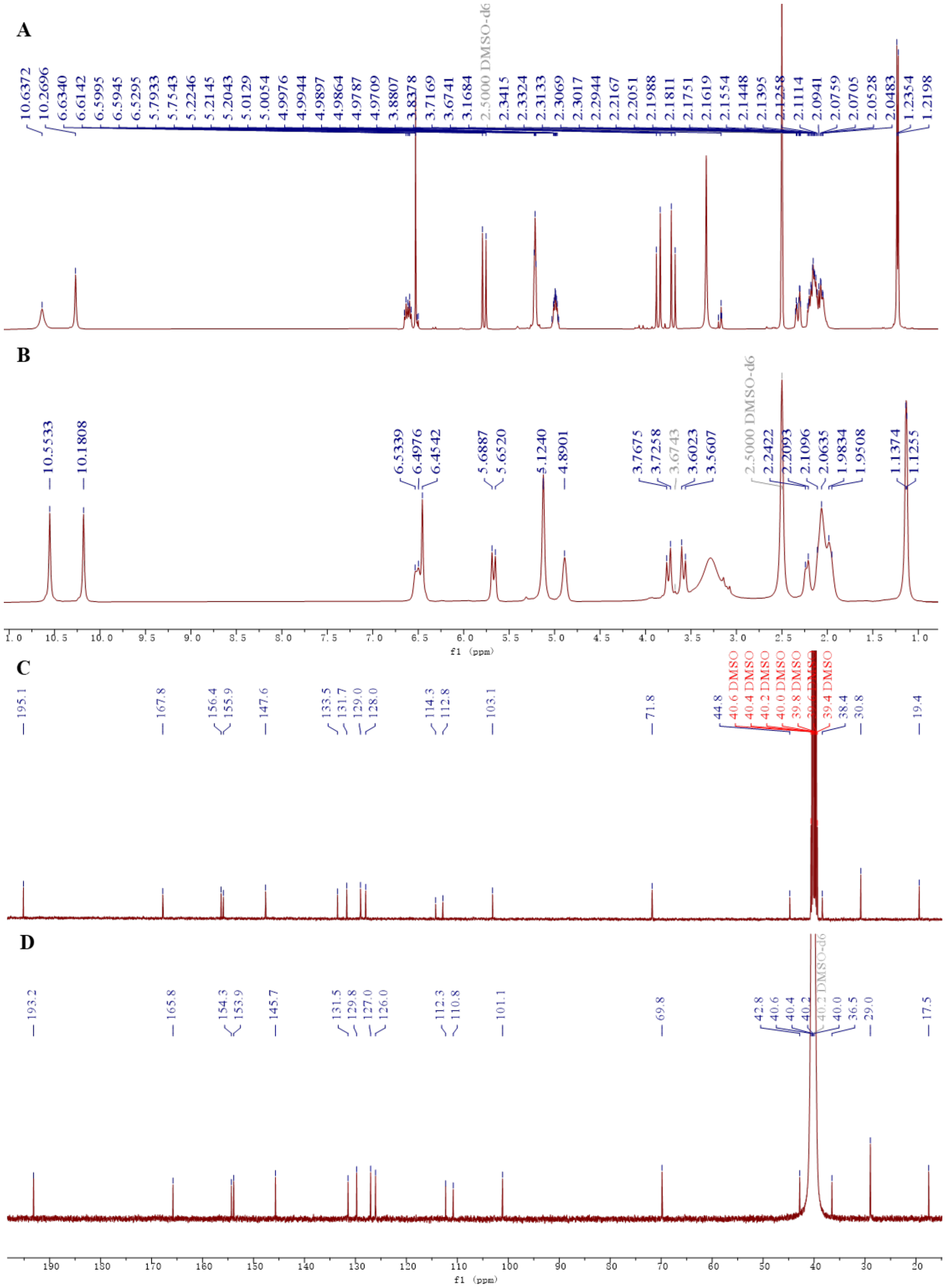

Figure S8 NMR data of copper ion co-incubation with PoD (A) ^{1}H-NMR spectrum of PoD (B) ^{1}H-NMR spectrum of PoD + Cu^{2+} (C) ^{13}C-NMR spectrum of PoD (D) ^{13}C-NMR spectrum of PoD + Cu^{2+}

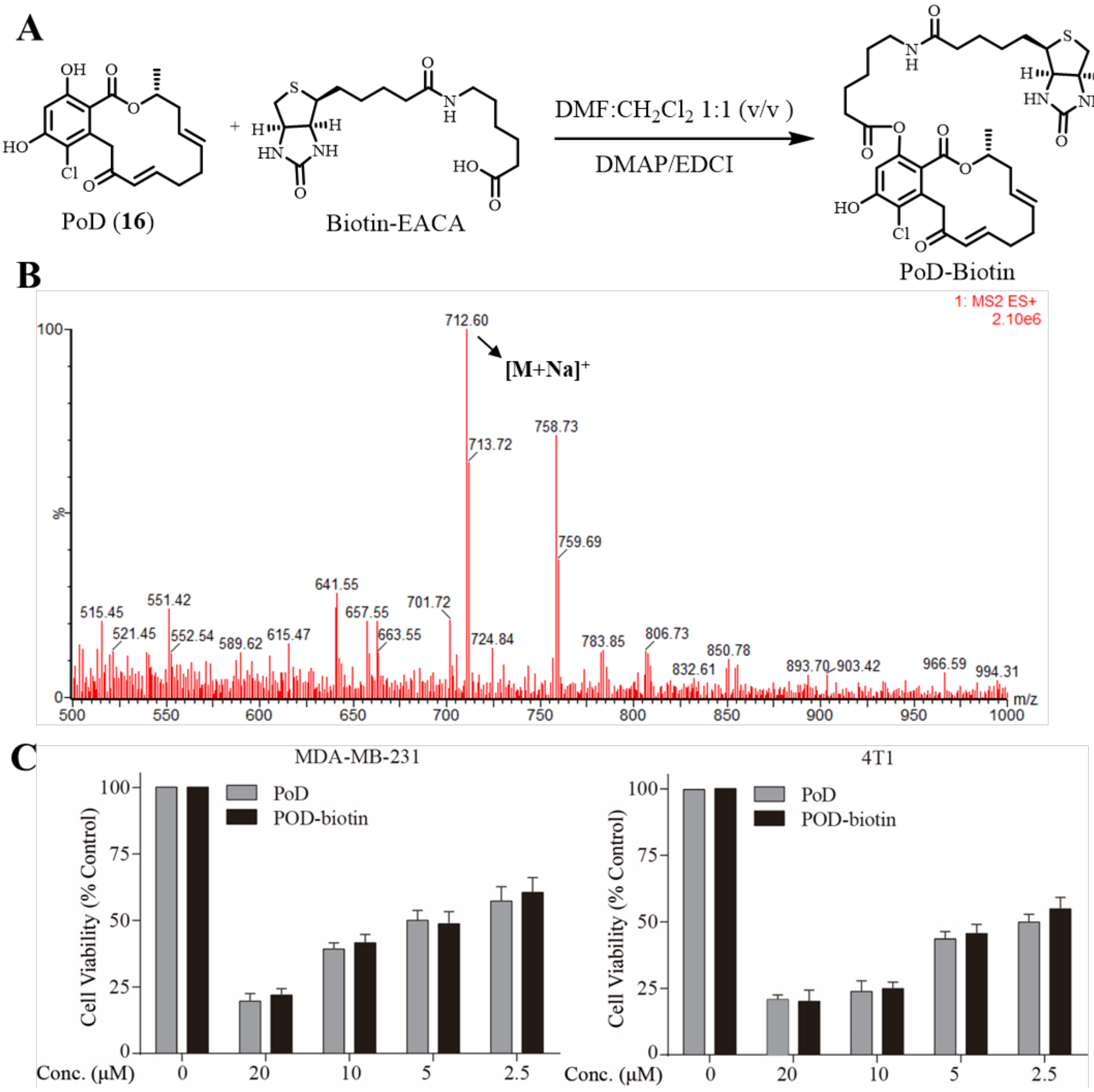

Figure S9 Synthesis and bioactivity evaluation of PoD-biotin. (A) Synthesis scheme of PoD-biotin. To synthesize PoD-Biotin, a solution of PoD (10 mg) in anhydrous CH_2Cl_2 : DMF (1:1, 4 mL) was added to a mixture containing biotin-EACA (20 mg), DMAP (7 mg), and EDCI (11 mg). The reaction mixture was stirred at room temperature for 12 hours and subsequently evaporated to dryness, yielding a residue. The residue was purified sequentially using silica gel column chromatography ($CHCl_3$/MeOH, 35:65) and preparative HPLC (45% acetonitrile in water), resulting in the isolation of compound PoD-biotin (5.38 mg). (B) The MS spectra of PoD-biotin (C) Cell viability of MDA-MB-231 and 4T1 cells exposed to indicated concentrations of PoD and PoD-biotin for 24 h.

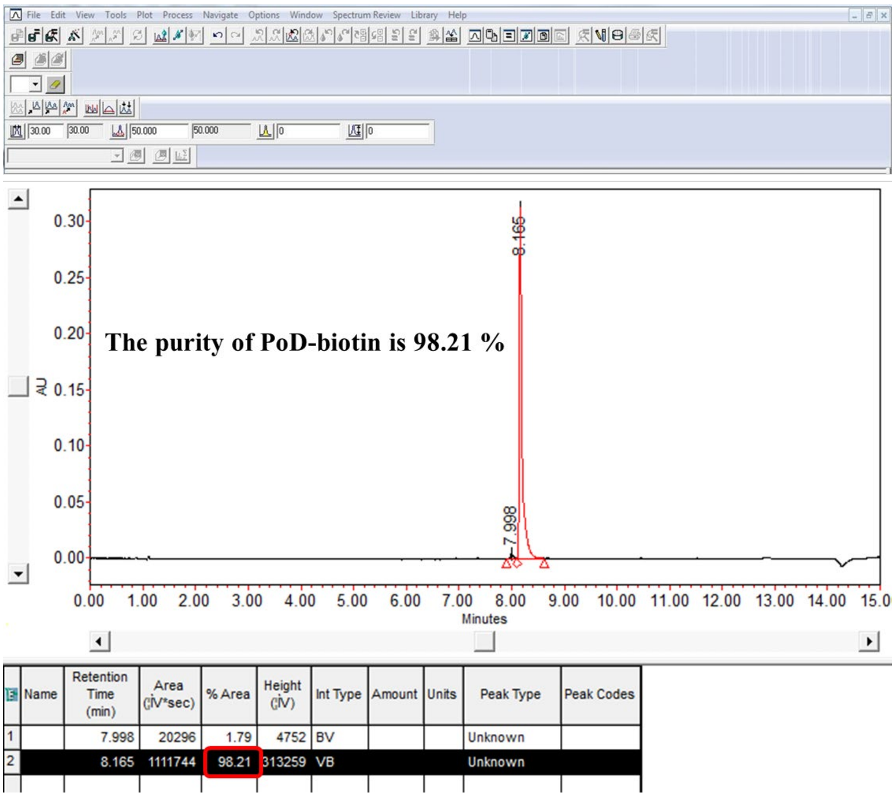

Figure S10 HPLC profiles of PoD-biotin. The HPLC analysis was performed using a Waters CORTECS ®C_{18} column (4.6 × 50 mm, 2.7 μm), the mobile phase consisted of a linear gradient of CH_3CN-H_2O (10%–100%) over 15 minutes, and the absorption wavelength was set at 210 nm.

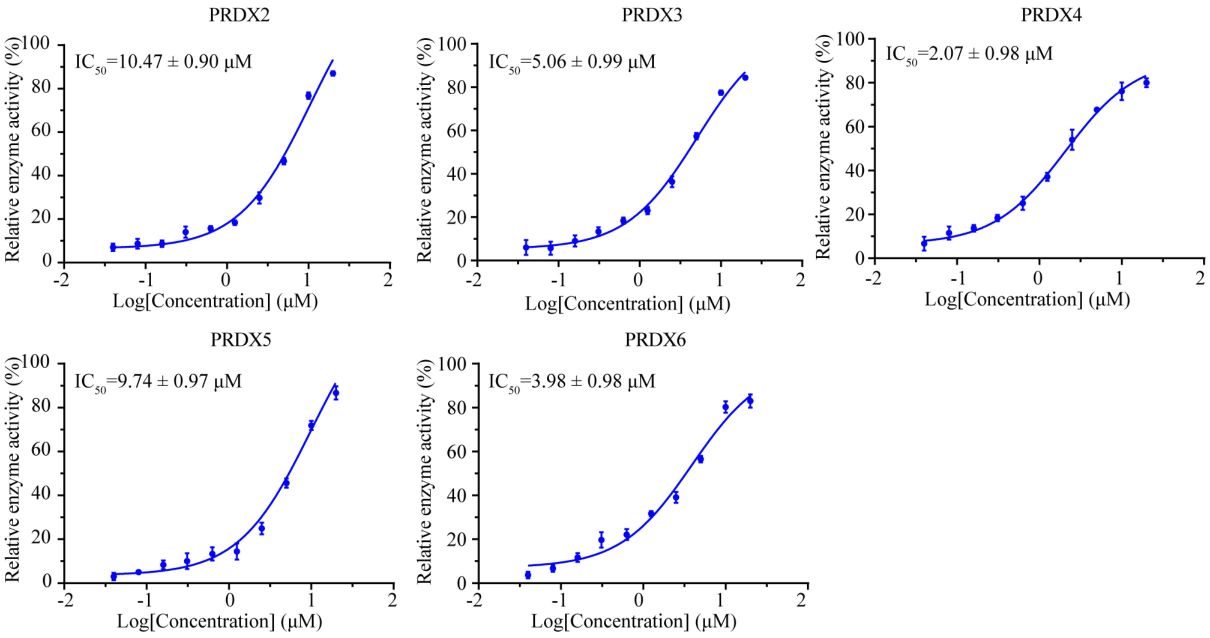

Figure S11 The inhibitory enzyme activity of PoD against PRDX2-6

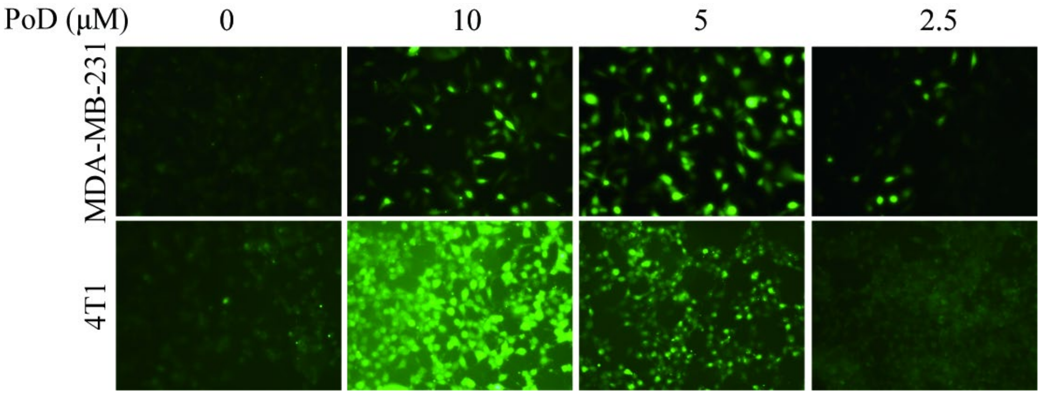

Figure S12 The effect of PoD on the ROS production in MDA-MB-231 and 4T1 cells

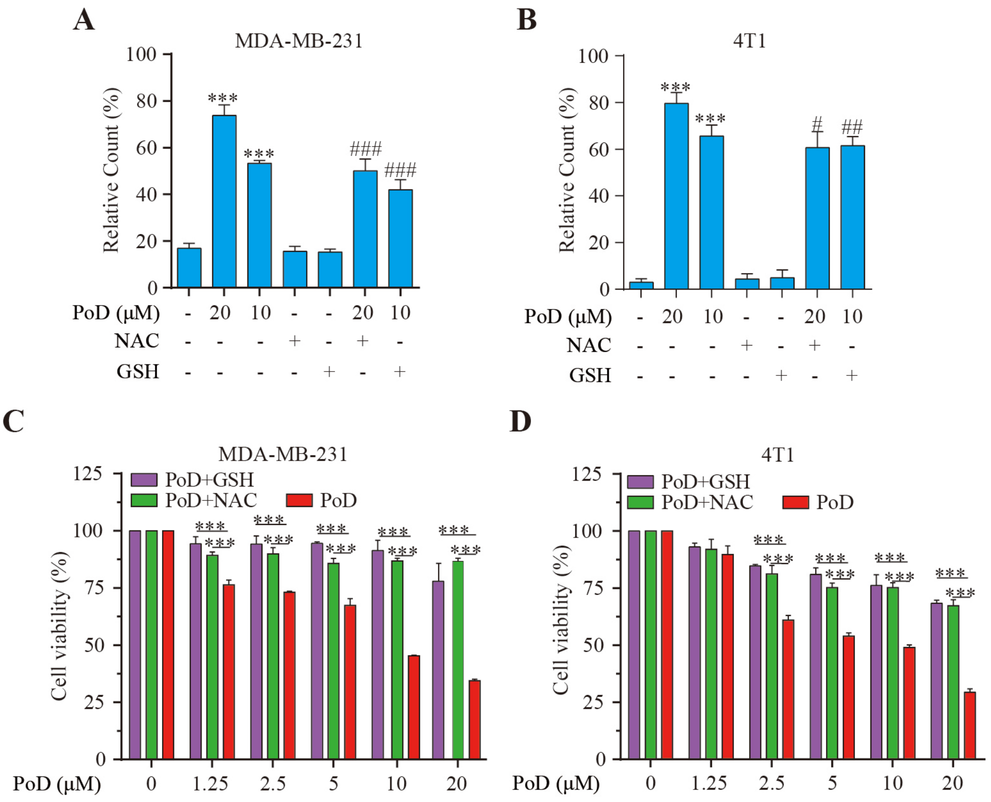

Figure S13 NAC and GSH on cell viability in MDA-MB-231 and 4T1 cells. (A and B) The ROS levels in MDA-MB-231 and 4T1 cells after treatment with various concentrations of PoD were measured by fluorescence microscope (DCFH-DA staining). (C and D) The antioxygen NAC and GSH eliminated the increase of PoD-induced cytotoxicity. The cell viability was evaluated by SRB assay. *** $p < 0.001$ vs. control.

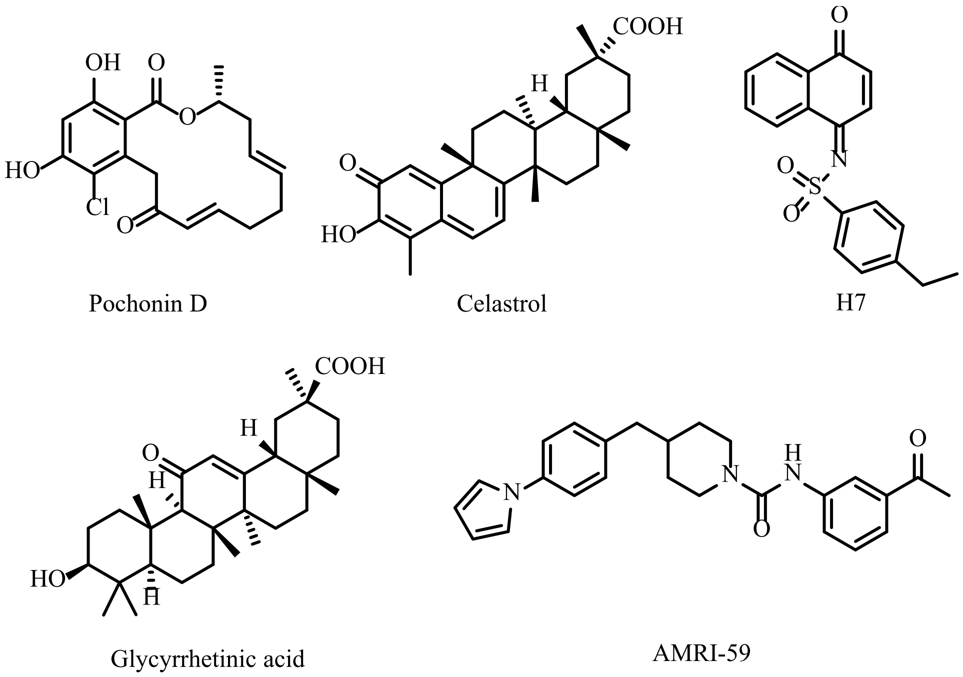

Figure S14 Chemical structure of PRDX1 inhibitors

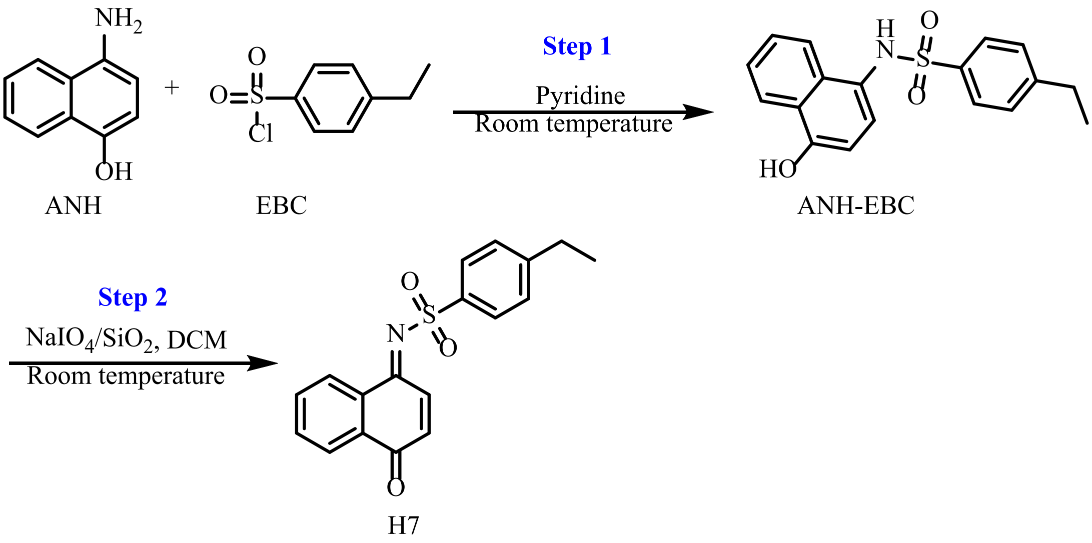

Figure S15 The synthesis scheme for the PRDX1 inhibitor H7. Step 1: Under ice-bath conditions, 4-amino-1-naphthol hydrochloride (ANH, 122.46 mg) was dissolved in pyridine (8 mL). 4-Ethylbenzenesulfonyl chloride (EBC, 120 μL) was then added dropwise, and the reaction mixture was stirred at room temperature for 20 minutes. The solvent was evaporated, and the residue was dissolved in ethyl acetate (EA). The organic layer was washed twice with saturated 1 N HCl, distilled water, and brine, respectively. The organic phase was dried and evaporated under vacuum. The crude product was purified by column chromatography, affording a white to yellow powder (ANH-EBC, 172 mg). Step 2: The ANH-EBC (40 mg) was

dissolved in dry CH_2Cl_2 (3 mL), followed by the addition of $NaIO_4/SiO_2$ (509 mg, 0.36 mmol $NaIO_4$ per gram of SiO_2). The reaction mixture was stirred at room temperature for 15 minutes under light-protected conditions. Afterward, the solid particles were filtered out, and the residue was washed with CH_2Cl_2. The filtrate was washed twice with saturated brine, dried, and concentrated. The crude product was purified by column chromatography using wet loading, yielding a white powder (H7, 21 mg).

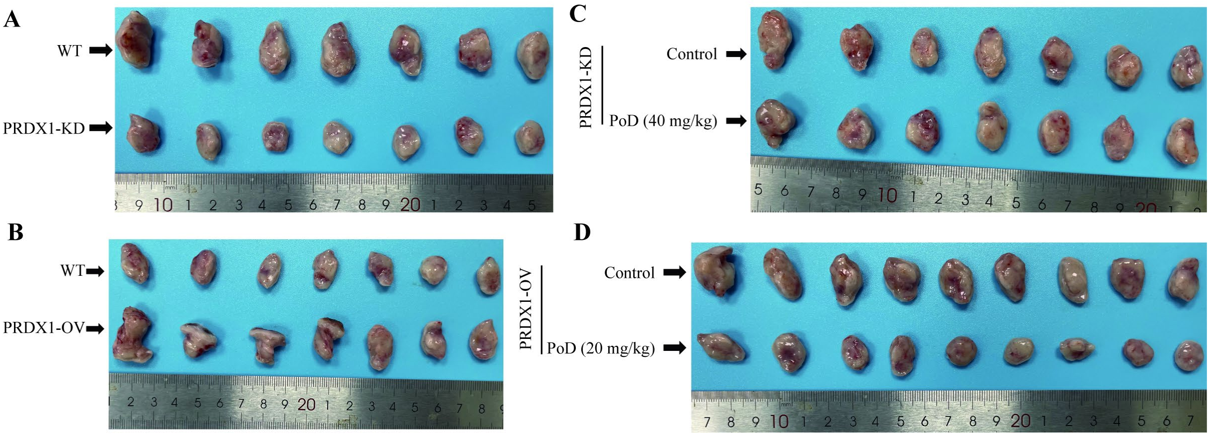

Figure S16 The images of 4T1 xenograft tumors

Experimental Sections

General Experimental Procedures

The optical rotations were measured at room temperature using a Perkin Elmer 241 polarimeter. Circular dichroism (CD) spectra were recorded on a Chirascan Circular Dichroism J-810 spectrometer. Ultraviolet (UV) spectra were obtained with a Shimadzu Biospec-2600i spectrophotometer. Infrared (IR) spectra were collected using a Bruker Tensor 27 FT-IR spectrometer. Nuclear magnetic resonance (NMR) experiments were conducted at 298 K on a Bruker AM-400 NMR spectrometer. Electrospray ionization mass spectrometry (ESIMS) data were acquired on a Waters Xevo-TQD MS spectrometer, and high-resolution electrospray ionization mass spectrometry (HRESIMS) was performed on an Agilent Q-6200-TOF spectrometer. Column chromatography (CC) was carried out using silica gel (200–300 mesh and 100–200 mesh, Qingdao Yu-Ming-Yuan Chemical Co., Ltd., Qingdao, China), LiChroprep RP-18 gel (40–63 μm, Merck, Darmstadt, Germany), and Sephadex LH-20 (40–63 μm,

Pharmacia Fine Chemical Co., Uppsala, Sweden). Thin-layer chromatography (TLC) was conducted on GF254 silica gel plates (Qingdao Yu-Ming-Yuan Chemical Co., Ltd.), with spots visualized under UV light and further treated with a 5 % sulfuric acid-ethanol reagent. High-performance liquid chromatography (HPLC) separations were performed using an LC-3000 HPLC system equipped with YMC-Pack ODS-A C_{18} columns (10 × 250 mm, 5 μm and 20 × 250 mm, 5 μm). HPLC analysis was carried out on a Waters ACQUITY Arc system with a Waters CORTECS® C_{18} column (4.6 × 50 mm, 2.7 μm). Finally, LC-MS were performed on a Waters Xevo-TQD MS spectrometer using a Waters ACQUITY UPLC BEH C_{18} column (2.1 × 50 mm, 1.7 μm).

Isolation and Identification of Fungus

The fungal strain *Ilyonectria* sp. FL-710 was isolated from the stems of fresh *Aster tataricus* L. f. (Asteraceae) harvested from the Botanical Garden of China Pharmaceutical University (118°55'15"E and 31°54'23"N) of Jiangsu Province, China, in July 2019. This strain was authenticated based on the morphology and the sequence analysis of the ITS region of rDNA (GenBank No. MN726651), and deposited in the School of Traditional Chinese Pharmacy, China Pharmaceutical University.

Extraction, Isolation and Purification

The fungal strain *Ilyonectria* sp. FL-710 was cultured on PDA medium at 28 °C for 7 days. Then the agar plugs were inoculated into autoclaved medium in 450 Erlenmeyer flasks (1 L) containing rice medium (220 mL deionized water and 180 g rice, sterilized in 121 °C for 30 min). After the strain was fermented at 28 °C for 35 days, the rice fermented materials were obtained and extracted with EtOAc (soaked in about 75 L EtOAc directly) four times. The extracted solutions were combined and evaporated under reduced pressure to yield the extracts (3.2 kg). Throughout the experiment, antitumor activity-guided isolation was conducted. The extracts were subjected to silica gel column chromatography (CC) (200–300 mesh) and eluted with a petroleum ether-acetone gradient system (20:1, 1:1, and 0:1, each eluted volume was 20 L) to yield three fractions (Fr. 1 to Fr. 3). Among these, Fr. 2 exhibited the most potent inhibitory activity against TNBC cells. Subsequently, Fr. 2 (169 g) was further

chromatographed by RP-18 gel CC and eluted with a methanol-water gradient system (30:1, 40:1, 50:1, 60:1, 70:1, 80:1 and 90:1, each eluted volume was 18 L) to yield seven fractions (Fr. 2-1–Fr. 2-7, the fraction combination is based on bioactivity test). Fr. 2-5 (45 g), which contained all components with higher inhibitory activity against TNBC cells, was further subjected to Sephadex LH-20 CC with an elution solvent of chloroform-methanol (1:1, total eluted volumes were 17 L) to yield four fractions (Fr. 2-5-1–Fr. 2-5-4). Based on cytotoxic activity evaluations, Fr. 2-5-2 (16 g) was subjected to silica gel column chromatography (CC) (200–300 mesh) and eluted with a chloroform-methanol gradient system (0:100–0:100, total eluted volumes were about 25 L) to yield six fractions (Fr. 2-5-2-1–Fr. 2-5-2-6). Fr. 2-5-2-1 (1.1 g) was further purified by semipreparative HPLC (2 mL/min, CH_3CN-H_2O, 66:34) to yield ilyolactone F (**6**) (39.5 mg, t_R = 14.34 min), ilyolactone H (**8**) (114.6 mg, t_R = 13.11 min), and monorden E (**10**) (26.1 mg, t_R = 12.26 min). Fr. 2-5-2-2 (2.2 g) was further purified by semipreparative HPLC (2 mL/min, CH_3CN-H_2O, 55:45) to yield ilyolactone A (**1**) (23.2 mg, t_R = 16.11 min), ilyolactone B (**2**) (17.2 mg, t_R = 15.44 min), ilyolactone C (**3**) (11.1 mg, t_R = 16.55 min), ilyolactone D (**4**) (12.5 mg, t_R = 13.21 min), and ilyolactone E (**5**) (7.4 mg, t_R = 18.25 min). Fr. 2-5-2-3 (1.3 g) was further purified by semipreparative HPLC (2 mL/min, CH_3CN-H_2O, 59:31) to yield Ilyolactone G (**7**) (36.1 mg, t_R = 12.24 min), monocillin III (**11**) (15.3 mg, t_R = 11.86 min), radicicol (**12**) (186.4 mg, t_R = 13.26 min), and monorden C (**13**) (22.4 mg, t_R = 10.78 min). Fr. 2-4-2-4 (643 mg) was further purified by semipreparative HPLC (2 mL/min, CH_3CN-H_2O, 58:32) to yield ilyolactone I (**9**) (7.9 mg, t_R = 18.22 min). Fr. 2-4-2-5 (1.3 g) was further purified by semipreparative HPLC (2 mL/min, CH_3CN-H_2O, 78:22) to yield pochonin A (**19**) (43.3 mg, t_R = 9.55 min), monocillin I (**20**) (127.5 mg, t_R = 11.89 min), and radicicol B (**21**) (9.9 mg, t_R = 13.21 min). Fr. 2-4-2-6 (3.1 g) was further purified by semipreparative HPLC (2 mL/min, CH_3CN-H_2O, 41:59) to yield *O*-desmethylgreensporone C (**14**) (23.4 mg, t_R = 21.55 min), monocillin II (**15**) (443.3 mg, t_R = 19.32 min), pochonin D (**16**) (17.5 mg, t_R = 18.58 min), radicicol C (**17**) (29.1 mg, t_R = 21.96 min), unnamed compound (**18**) (37.3 mg, t_R = 22.31 min), radisicol analogus I (**22**) (61.2 mg, t_R = 24.18 min), pochonin G (**23**) (6.1 mg, t_R = 17.03 min), and curvularin (**24**) (15.2 mg, t_R = 16.56 min).

Physical Constants, Spectrometric, and Spectroscopic Data

*Ilyolactone A (**1**)*: colourless crystal; $[\alpha]_D^{20.0}$ -30.0 (*c* 0.04, MeOH); UV (MeOH) λ_{max} (log ε): 216 (2.11), 250 (1.03), 261 (0.96), 313 (0.48) nm; IR (KBr) ν_{max} 2991, 2891, 1645, 1384, 1240, 1050, 654 cm^{-1}; ECD (0.38 mg/mL, MeOH) λ_{max} ($\Delta\varepsilon$) 218 (-18.14), 232 (2.63), 246 (-0.40), 264 (6.60), 299 (-1.81) nm; ^{1}H (400 MHz) and ^{13}C (100 MHz) NMR data, see **Table S2**; $[M+Na]^+$; HRESIMS (positive): *m/z* 437.09760 (calcd for $C_{19}H_{23}ClO_8$, 437.09737).

*Ilyolactone B (**2**)*: colourless crystal; $[\alpha]_D^{20.0}$ -15.0 (*c* 0.04, MeOH); UV (MeOH) λ_{max} (log ε): 217 (1.97), 250 (0.93), 262 (0.82), 315 (0.44) nm; IR (KBr) ν_{max} 2999, 2853, 1745, 1384, 1241, 1092, 663 cm^{-1}; ECD (0.34 mg/mL, MeOH) λ_{max} ($\Delta\varepsilon$) 212 (-25.75), 227 (-3.32), 238 (-8.72), 263 (2.67), 284 (-1.41), 316 (5.21) nm; ^{1}H (400 MHz) and ^{13}C (100 MHz) NMR data, see **Table S2**; HRESIMS (positive): *m/z* 414.10715 (calcd for $C_{19}H_{23}ClO_8$, 437.09737).

*Ilyolactone (**3**)*: white block; $[\alpha]_D^{20.0}$ -20.0 (*c* 0.04, MeOH); UV (MeOH) λ_{max} (log ε): 220 (2.50), 264 (0.88), 314 (0.56) nm; IR (KBr) ν_{max} 2896, 1384, 1241, 1022, 844 cm^{-1}; ECD (0.53 mg/mL, MeOH) λ_{max} ($\Delta\varepsilon$) 213 (-33.08), 228 (-5.19), 238 (-13.36), 263 (2.85), 284 (-1.95), 308 (8.43) nm; ^{1}H (400 MHz) and ^{13}C (100 MHz) NMR data, see **Table S2**; HRESIMS (positive): *m/z* 437.09730 (calcd for $C_{19}H_{23}ClO_8$, 437.09737).

*Ilyolactone D (**4**)*: white block; $[\alpha]_D^{20.0}$ -12.5 (*c* 0.04, MeOH); UV (MeOH) λ_{max} (log ε): 220 (2.60), 265 (0.88), 314 (0.60) nm; IR (KBr) ν_{max} 2923, 2853, 1736, 1653, 1384, 1241, 1093, 664 cm^{-1}; ECD (0.29 mg/mL, MeOH) λ_{max} ($\Delta\varepsilon$) 214 (-39.73), 230 (-4.08), 238 (-10.97), 262 (6.55), 311 (8.38) nm; ^{1}H (400 MHz) and ^{13}C (100 MHz) NMR data, see **Table S2**; HRESIMS (positive): *m/z* 437.09755 (calcd for $C_{19}H_{23}ClO_8$, 437.09737).

*Ilyolactone E (**5**)*: white block; $[\alpha]_D^{20.0}$ -17.5 (*c* 0.04, MeOH); UV (MeOH) λ_{max} (log ε): 220 (2.96), 250 (1.22), 262 (1.21), 315 (0.69) nm; IR (KBr) ν_{max} 2922, 1721, 1646, 1356, 1084, 1001, 773 cm^{-1}; ECD (0.52 mg/mL, MeOH) λ_{max} ($\Delta\varepsilon$) 213 (-23.38), 264 (4.38), 294 (-0.43), 313 (3.27) nm; ^{1}H (400 MHz) and ^{13}C (100 MHz) NMR data, see **Table S3**; HRESIMS (positive): *m/z* 428.12444 (calcd for $C_{20}H_{25}ClO_8$, 451.11302).

*Ilyolactone F (**6**)*: white block; $[\alpha]_D^{20.0}$ -22.5 (*c* 0.04, MeOH); UV (MeOH) λ_{max} (log ε): 250 (2.26), 316 (0.42) nm; IR (KBr) ν_{max} 2922, 2853, 1721, 1355, 1240, 1062, 772 cm^{-1}; ECD (0.45

mg/mL, MeOH) λ_{max} ($\Delta\varepsilon$) 217 (-5.69), 235 (1.92), 257 (-2.68), 274 (1.28) nm; ^{1}H (400 MHz) and ^{13}C (100 MHz) NMR data, see **Table S3**; HRESIMS (positive): *m/z* 405.07166 (calcd for $C_{18}H_{19}ClO_7$, 405.07115).

Ilyolactone G (7): white block; $[\alpha]_D^{20.0}$ -20.0 (*c* 0.04, MeOH); UV (MeOH) λ_{max} (log ε): 220 (2.50), 264 (0.88), 314 (0.66) nm; IR (KBr) ν_{max} 2914, 1681, 1384, 1241, 1050 cm^{-1}; ECD (0.47 mg/mL, MeOH) λ_{max} ($\Delta\varepsilon$) 269 (-1.81), 318 (0.87) nm; ^{1}H (400 MHz) and ^{13}C (100 MHz) NMR data, see **Table S3**; HRESIMS (positive): *m/z* 437.09738 (calcd for $C_{18}H_{19}ClO_7$, 437.09737).

*Ilyolactone H (**8**)*: colourless crystal; $[\alpha]_D^{20.0}$ -21.6 (*c* 0.04, MeOH); UV (MeOH) λ_{max} (log ε): 219 (1.83), 234 (1.46), 249 (1.56), 261 (1.04), 317 (0.47) nm; IR (KBr) ν_{max} 2925, 2853, 1719, 1571, 1356, 1083, 663 cm^{-1}; ECD (0.41 mg/mL, MeOH) λ_{max} ($\Delta\varepsilon$) 213 (-34.93), 257 (1.60), 383 (0.56), 310 (7.05) nm; ^{1}H (400 MHz) and ^{13}C (100 MHz) NMR data, see **Table S3**; HRESIMS (positive): *m/z* 405.10822 (calcd for $C_{19}H_{23}ClO_6$, 405.10754).

*Ilyolactone I (**9**)*: colourless crystal; $[\alpha]_D^{20.0}$ -12.5 (*c* 0.04, MeOH); UV (MeOH) λ_{max} (log ε): 241 (2.74), 249 (3.23) , 259 (1.44), 331 (0.50) nm; IR (KBr) ν_{max} 2914, 2860, 1681, 1566, 1355, 1050, 664 cm^{-1}; ^{1}H (400 MHz) and ^{13}C (100 MHz) NMR data, see **Table S4**; HRESIMS (positive): *m/z* 409.10239 (calcd for $C_{18}H_{23}ClO_7$, 409.10245).

Single Crystal X-ray Diffraction Analysis and Crystallographic Data for Ilyolactone A (1), Ilyolactone B (2), Ilyolactone H (8), and Ilyolactone I (9).

X-ray Crystallographic Analysis of 1: Colourless needle crystals of **1** was obtained from a solvent mixture of CH_3OH by slow evaporation method at room temperature. X-ray analysis was carried out on a Bruker APEX-II CCD diffractometer with *Cu* K*α* radiation (λ = 1.54). Using Olex2, the structure was solved with the ShelXT structure solution program using intrinsic phasing and refined with the ShelXL refinement package using least-squares technique. Crystallographic data for the structure of **1** have been deposited in the Cambridge Crystallographic Data Centre (deposition number: CCDC 2151623). Copies of these data can be obtained free of charge on application at the following address: www.ccdc.cam.ac.uk (or

from the Cambridge Crystallographic Data Centre, 12 Union Road, Cambridge CB21EZ, UK; fax (+44) 1223-336-033; or deposit@ccdc.cam.ac.uk).

Crystal data of **1**: $C_{19}H_{23}ClO_8$, M = 414.11, crystal size 0.13 × 0.12 × 0.1 mm^3, monoclinic, space group *P2₁* (no. 1) a = 7.7412(3) Å, b = 10.9475(4) Å, c = 12.0715(4) Å, V = 961.39(6) Å^3, Z = 1, T = 193.0 K, μ (Cu Kα) = 2.194 mm^{-1}, D_{calc} = 1.464 g/cm^3, 24887 reflections measured (7.7° ≤ 2θ ≤ 136.748°), 6582 unique (R_{int} = 0.0467, R_{sigma} = 0.0449) which were used in all calculations. The final R_1 was 0.0572 (I > 2σ(I)) and wR_2 was 0.1178 (all data). Flack parameter = 0.091(15).

X-ray Crystallographic Analysis of 2: Colourless needle crystals of **2** was obtained from a solvent mixture of CH_3OH by slow evaporation method at room temperature. X-ray analysis was carried out on a Bruker APEX-II CCD diffractometer with *Cu* Kα radiation (λ = 1.54). Using Olex2, the structure was solved with the ShelXT structure solution program using intrinsic phasing and refined with the ShelXL refinement package using least-squares technique. Crystallographic data for the structure of **2** have been deposited in the Cambridge Crystallographic Data Centre (deposition number: CCDC 2151624). Copies of these data can be obtained free of charge on application at the following address: www.ccdc.cam.ac.uk (or from the Cambridge Crystallographic Data Centre, 12 Union Road, Cambridge CB21EZ, UK; fax (+44) 1223-336-033; or deposit@ccdc.cam.ac.uk).

Crystal data of **2**: $C_{19}H_{23}ClO_8$, M = 414.11, monoclinic, space group $P2_1$ (no. 19) a = 8.8483(2) Å, b = 23.7325(4) Å, c = 28.0038(6) Å, V = 5880.6(2) Å^3, Z = 4, T = 193.0 K, μ (Cu Kα) = 2.093 mm^{-1}, D_{calc} = 1.464 g/cm^3, 9594 reflections measured (3.850° ≤ θ ≤ 68.397°), 6548 unique (R_{int} = 0.0572) which were used in all calculations. The final R_1 was 6548 (I > 2σ(I)) and $wR2$ was 0.1178 (all data).

X-ray Crystallographic Analysis of 8: Colourless needle crystals of **8** was obtained from a solvent mixture of CH_3OH by slow evaporation method at room temperature. X-ray analysis was carried out on a Bruker APEX-II CCD diffractometer with *Cu* Kα radiation (λ = 1.54). Using Olex2, the structure was solved with the ShelXT structure solution program using intrinsic phasing and refined with the ShelXL refinement package using least-squares

technique. Crystallographic data for the structure of **8** have been deposited in the Cambridge Crystallographic Data Centre (deposition number: CCDC 2151625). Copies of these data can be obtained free of charge on application at the following address: www.ccdc.cam.ac.uk (or from the Cambridge Crystallographic Data Centre, 12 Union Road, Cambridge CB21EZ, UK; fax (+44) 1223-336-033; or deposit@ccdc.cam.ac.uk).

Crystal data of **8**: $C_{18}H_{23}ClO_7$, M = 386.11, crystal size 0.13 × 0.12 × 0.08 mm^3, monoclinic, space group $P2_1$ (no. 19) a = 5.5813(2) Å, b = 17.3597(6) Å, c = 21.7300(8) Å, V = 2105.41(13) Å^3, Z = 4, T = 193.0 K, μ (Cu Kα) = 2.091 mm^{-1}, D_{calc} = 1.422 g/cm^3, 40392 reflections measured (6.516° ≤ 2θ ≤ 136.534°), 3844 unique (R_{int} = 0.0568, R_{sigma} = 0.0304) which were used in all calculations. The final R_1 was 0.0260 (I > 2σ(I)) and wR_2 was 0.0663 (all data). Flack parameter = 0.042(14).

X-ray Crystallographic Analysis of 9: Colourless needle crystals of **9** was obtained from a solvent mixture of CH_3OH by slow evaporation method at room temperature. X-ray analysis was carried out on a Bruker APEX-II CCD diffractometer with *Cu* Kα radiation (λ = 1.54). Using Olex2, the structure was solved with the ShelXT structure solution program using intrinsic phasing and refined with the ShelXL refinement package using least-squares technique. Crystallographic data for the structure of **9** have been deposited in the Cambridge Crystallographic Data Centre (deposition number: CCDC 2151626). Copies of these data can be obtained free of charge on application at the following address: www.ccdc.cam.ac.uk (or from the Cambridge Crystallographic Data Centre, 12 Union Road, Cambridge CB21EZ, UK; fax (+44) 1223-336-033; or deposit@ccdc.cam.ac.uk).

Crystal data of **9**: $C_{18}H_{23}ClO_7$, M = 386.11, crystal size 0.15 × 0.13 × 0.12 mm^3, monoclinic, space group $P2_1$ (no. 4) a = 13.8903(4) Å, b = 4.82280(10) Å, c = 30.6607(4) Å, V = 2041.54(9) Å^3, Z = 2, T = 193.0 K, μ (Cu Kα) = 2.049 mm^{-1}, D_{calc} = 1.346 g/cm^3, 32566 reflections measured (5.8° ≤ 2θ ≤ 136.558°), 7469 unique (R_{int} = 0.0392, R_{sigma} = 0.0385) which were used in all calculations. The final R_1 was 0.0264 (I > 2σ(I)) and wR_2 was 0.0695 (all data). Flack parameter = 0.023(10).

Experiment Methods for Calculational Chemistry

Conformational Analysis

Conformational analysis was initially performed using OpenBabel[13] with genetic algorithm at MMFF94 force field for undetermined configurations of **3**–**7**. Room-temperature equilibrium populations were calculated according to Boltzmann distribution law. The energies and populations of dominative conformers were provided in **eq. 1**.

$$\frac{N_i}{N} = \frac{g_i e^{-\frac{E_i}{k_B T}}}{\sum g_i e^{-\frac{E_i}{k_B T}}} \qquad \textbf{(1)}$$

where N_i is the number of conformers i with energy E_i and degeneracy g_i at temperature *T*, and k_B is Boltzmann constant.

ECD Calculation

The theoretical calculations were carried out using Gaussian 09[14]. At first, all conformers were optimized at PM6. Room-temperature equilibrium populations were calculated according to Boltzmann distribution law, based on which dominative conformers of population over 1% were kept. The chosen conformers were further optimized at B3LYP/6-31G(d) in gas phase. Vibrational frequency analysis confirmed the stable structures. ECD calculations were conducted at B3LYP/6-311G(d,p) level in methanol with IEFPCM model using Time-dependent Density functional theory (TD-DFT).[15] Rotatory strengths for a total of 10 excited states were calculated. The ECD spectrum was simulated in SpecDis by overlapping Gaussian functions for each transition according to eq. **2**.

$$\Delta\varepsilon(E) = \frac{1}{2.297\times10^{-39}} \times \frac{1}{\sqrt{2\pi\sigma}} \sum_{i}^{A} \Delta E_i R_i e^{-\left(\frac{E-E_i}{2\sigma}\right)^2} \qquad \textbf{(2)}$$

where σ represents the width of the band at 1/*e* height, while ΔE_i and R_i are the excitation energies and rotatory strengths for transition *i*, respectively.

The σ and UV-shift values were set 0.4 eV and 5 nm, respectively. Spectra of the enantiomers were produced directly by mirror inversion about the horizontal axis.

Conformational analysis was carried out using OpenBabel with the genetic algorithm at the MMFF94 force field for all possible configurations of **3**–**7**. The Boltzmann distribution law was used to calculate room temperature equilibrium populations. Only the conformers with a Boltzmann-population of more than 1 % were then subjected to ECD calculations. The theoretical ECD calculation was carried out using Gaussian 09.[14] The conformers were optimized at the PM6 with the semiempirical theory method and then at the B3LYP/6-311G (d, p) in methanol using the IEFPCM model. The theoretical calculation of ECD was conducted in methanol using TD-DFT at the same theory level.

NMR Calculation

NMR calculations were carried out by Gaussian 09 following the protocol adapted from Michael et al.[16] The theoretical calculation of NMR was conducted using the Gauge-Including Atomic Orbitals (GIAO) method at the mPW1PW91/6-311+G (2d, p) in methanol using the IEFPCM model. The NMR chemical shift value of tetramethylsilane was calculated at the same level and used as a reference. Finally, the calculated NMR data of these conformers were averaged according to the Boltzmann distribution theory and their relative Gibbs free energy. The linear R^2 and RMSD were calculated for the evaluation of the deviations between the experimental and calculated results (Tables S8 and S9).

Cell Lines and Culture Conditions

In this study, HCT116, A549, HepG2, MDA-MB-231, PANC-1, SGC-7901, 4T1, BT549, SUM159, MDA-MB-157, and MCF-10A cells were obtained from the cell bank of the Chinese Academy of Sciences (Shanghai, China). All cells were cultured according to the instructions. Cells were confirmed to have no mycoplasma contamination and were passaged no more than 25 to 30 times after thawing.

Cell Viability Assay

Cell viability was assessed using the Sulforhodamine B (SRB) assay (Sigma, St. Louis, MO, USA). Cells were seeded into 96-well plates at a density of 1×10^4 to 1×10^5 cells per well. After treated with PoD for 12, 24, 48, or 72 h (with or without pretreatment using Cu^{2+}, TM,

GSH, or NAC), the cells were fixed with 10 % cold trichloroacetic acid (TCA) for 1 hour. The fixed cells were washed with running water, and 100 μL of 0.4 % SRB solution was added to each well, followed by incubation for 30 min. After washing three times with 1% TCA and air-drying, the SRB-bound cells were solubilized in 100 μL/well of 10 mM unbuffered Tris base. Optical density (OD) values were measured at 540 nm using a microplate reader (Biotek, Winooski, VT, USA).

Colony Formation Assay

The clonogenic of TNBC cells were evaluated by colony formation assay. MDA-MB-231 and 4T1 cells were seeded into 6-well plates (approximately 2×10^3 cells per well) with the conditional medium and then treated with different concentrations of PoD for 24 h. After culturing for another 14 days in a 37 °C incubator with 5 % CO_2, the plates were washed with PBS and fixed with 4 % paraformaldehyde. After staining with 0.1 % crystal violet (Beyotime Biotechnology, Shanghai, China), the colonies were counted manually. The images were captured with a camera (Canon EOS-750D, Tokyo, Japan).

Cell Cycle Analysis

For cell cycle analysis, the PoD-treated cells were collected and fixed by 70 % ice-cold ethanol (v/v) overnight at 4 °C. After washed with cold PBS, the cells were further incubated with 0.05 mg/mL propidium iodide (PI, Sigma, St. Louis, USA) and 0.1 mg/mL RNase A (Yeasen, Shanghai, China) at 37 °C for 30 min in the dark. The cell cycle was analyzed by Invitrogen™ Attune™ NxT Flow Cytometer (Thermo Fisher Scientific, USA). Subsequently, the PI fluorescence signal were analyzed using the ModFit LT software (Verity Software House, Topsham, USA).

Annexin V-FITC/PI Double Staining Assay

Apoptosis was analysed by Annexin V-FITC/PI staining kit (Yeasen, Shanghai, China). The PoD-treated cells were collected and washed with cold PBS. Then, the cells were incubated with Annexin V-FITC and PI for 15 min at 4 °C in the dark. The stained cells were subjected to Invitrogen™ Attune™ NxT Flow Cytometer (Thermo Fisher Scientific, USA) and the data

were analyzed by FlowJo™ V10.

RNA-seq Analysis.

MDA-MB-231 cells were in 6-well plates treated with PoD (10 μM) for 24 h. The total RNA extraction of cells, cDNA library preparation, RNA sequencing, quality control and analysis were performed by Personalbio Technology Co., Ltd. (Shanghai, China). Briefly, total RNA was extracted by RNA extraction kit, following by cDNA library preparation with poly (A) selection. The libraries were sequenced by next-generation Sequencing (NGS) based on Illumina Sequencing platform. Differential statistical analysis of gene expression was performed by DESeq. GO enrichment analyses were performed with the database established by the Gene Ontology Consortium (http://geneontology.org/). KEGG enrichment analyses were performed with the database of Kyoto Encyclopedia of Genes and Genomes (http://www.kegg.jp/). Sequencing service were provided by Personal Biotechnology Co., Ltd. Shanghai, China. The data were analyzed using the free online platform Personalbio GenesCloud (https://www.genescloud.cn).

Connectivity Map Analysis

The gene expression profile of PoD in MDA-MB-231 cells was obtained from RNA-seq analysis. Differentially expressed genes associated with PoD were identified based on a fold-change threshold (FC $\geqslant 1.2$, $p < 0.05$). A total of 225 gene expression signatures for PoD were classified into two categories: "upregulated" and "downregulated" probe sets. These sets, consisting of significantly up- or downregulated probes, were saved as .grp files, which are required inputs for the Connectivity Map (CMap) analysis. The CMap query was conducted as a ‘quick query’ via the portal at http://portals.broadinstitute.org/cmap/.

Survival Curve Analysis and Expression Analysis

The RNA-sequencing data in the form of log 2 (FPKM + 0.1) and the corresponding clinical data of breast cancer patients from The Cancer Genome Atlas (TCGA) were acquired from the UCSC Xena browser (https://xenabrowser.net/), Furthermore, the RNA-seq data were transformed into log 2 (TPM + 1) for all subsequent analyses. One-way Cox regression analysis

and Kaplan-Meier survival analysis were used to explore the effect of PRDX1 or LIPT1 on the overall survival (OS) of breast cancer patients in TCGA. R packages were used to assess and visualize the predictive performance of PRDX1 or LIPT1, respectively. In addition, the GEPIA online website (http://gepia.cancer-pku.cn/) was used to investigate the differential expression of PRDX1 in tumor samples and corresponding normal tissues.

Evaluation of Intracellular Cu^{2+} Level

MDA-MB-231 and 4T1 cells were seeded in confocal dishes at a density of 1.0×10^5 cells per dish and cultured for 24 h. The cells were then treated with PoD with 10, 5, and 1 μM for 12 h. After that, the cells were then treated with RBH fluorescent probe for 12 h, and then observed by fluorescence microscope (Leica).

Spectrophotometric Titration of PoD with Cu^{2+}

PoD was titrated with $CuCl_2$ and the titration monitored by spectrophotometry in Tris buffer (10 mM, pH 7.4, 37 °C) that contained 25 % (v/v) DMSO in order to characterize complex formation under conditions that approximated those found biologically.

Streptavidin–Biotin Affinity Pull-Down Assay and LC-MS Analysis

MDA-MB-231 or 4T1 cell lysates were incubated overnight with biotin or PoD-biotin (80 μM) at 4°C with continuous rotation. Next, streptavidin agarose beads (Sigma-Aldrich, Missouri, USA) were added, and the mixture was incubated for 2 hours at 4°C with continuous shaking. After a series of washing steps, the beads with differentially bound proteins were analyzed by LC-MS/MS shotgun analysis, performed by Applied Protein Technology Co., Ltd.

Omics and Text based Target Enrichment and Ranking Analysis.

The OTTER workflow comprises three key steps. 1. Text mining for differentially expressed genes: OTTER begins by scanning PubMed abstracts for each differentially expressed gene, using user-defined keywords, such as “reactive oxygen species.” For every gene, a text score is calculated based on the presence and relevance of the keyword. This process is repeated for all genes in the user-provided list, enabling systematic text mining. 2. Protein-protein interaction (PPI) analysis. Following text mining and ratio quantification, OTTER evaluates

the protein-protein interactions among the top-ranking differentially expressed genes. Genes with a greater number of observed interactions receive higher PPI scores, reflecting their connectivity and potential significance in the network. 3. Final scoring and visualization. The final score for each gene is computed by summing its text and PPI scores. OTTER then generates a ranked table of the top 50 genes based on their final scores, accompanied by interactive plots for visualization. The RNA sequencing data of PoD in MDA-MB-231 cells were analyzed. The dataset was uploaded to the web server (http://otter-simm.com/otter.html) for processing.

Clinical Samples and IHC Analysis

A tissue microarray (TMA) consisting of paired breast cancer and corresponding adjacent nontumor colorectal tissues was purchased from Shanghai Outdo Biotech Co., Ltd. (Shanghai, China). IHC analysis was performed by Pathology Center of China Pharmaceutical University. After antigen retrieval and blocking, sections were incubated with primary antibodies against PRDX1 (Proteintech, Cat no : 15816-1-AP Wuhan, China) overnight at 4 °C. After washing with PBS, the sections were incubated with a secondary antibody for 2 h and developed with diaminobenzidine. Finally, the sections were stained with hematoxylin and sealed with neutral resin. All stained sections were scanned using microscopy.

Microscale Thermophoresis (MST) Analysis

The binding affinity of PoD with the PRDX1 protein was assessed using the microscale thermophoresis (MST) assay. The purified PRDX1 protein was labeled using a protein labeling kit (Monolith RED-NHS, Cat#MO-L018, Nano Temper). The diluted PoD was combined with labeled PRDX1 protein before being delivered into capillaries in an assay buffer (1 × PBS-P, 5% DMSO). A Monolith NT.115 instrument was used to conduct the MST measurement (Nano Temper Technologies). The results are analyzed using MO. Affinity Analysis software (v2.2.4).

Isothermal Titration Calorimetry (ITC) Analysis

Isothermal titration calorimetry analysis was performed with a PEAQ-ITC at 25 °C (Malvern). PRDX1 protein was solubilized using a PBS solution containing 1% (v/v) DMSO.

Ligand PoD was prepared by dilution with the same buffer used for proteins. Aliquots (2 μL) of the ligand solution at 20× the binding-site concentration were added by means of a 100 μL rotating stirrer-syringe to the reaction cell, containing 0.28 mL of the 0.02 mM protein solution. The heat of dilution was determined to be negligible in separate titrations of the ligand into the buffer solution. Calorimetric data analysis was carried out with MicroCal PEAQ-ITC Analysis Software (Malvern). Binding parameters such as the number of binding sites (n), the binding constant (Ka, M–1), and the binding enthalpy (ΔHa, kcal/mol of bound ligand) were determined by fitting the experimental binding isotherms. Ka was primarily determined by the slope of the isotherm in the equivalence point. The unknown molar concentration of binding sites of the PoD was estimated by altering the concentration of ligand (in the calculation), so as to fit one binding site.

Western Blot Analysis

For western blotting analysis, PoD-treated cells were lysed with lysis buffer (50 mM Tris, 150 mM NaCl, 1% Triton X-100 and 1 mM EDTA) containing protease inhibitor (Roche, Indianapolis, IN, USA) in ice. Then, the cell lysates were quantified by Detergent Compatible Bradford Protein Assay Kit (Beyotime, Hangzhou, China). The samples were subjected to SDS-PAGE and transferred to PVDF membranes (Millipore, Bedford, MA, USA). The membranes were blocked with 5 % nonfat milk dissolved in TBST (Buffered Saline with 0.1 % Tween 20) for 2 h, followed by incubation at 4 °C with primary antibodies overnight. The membranes were washed with TBST for three times and incubated with indicated secondary antibodies for 2 h at room temperature. After with TBST for three times, the bands on the membranes were detected by the ECL-Plus Western Blotting Detection System (Tanon, Shanghai, China).

Drug Affinity Responsive Target Stability (DARTS) Analysis

Total cellular protein of MDA-MB-231 or 4T1 was extracted using RIPA lysis buffer. Next, the protein concentration was determined using a gold bicinchoninic acid (BCA) protein concentration assay kit. Aliquots of 50 μg of protein were taken from the lysate to each 1.5 mL tube, and then PoD with various concentrations (20, 10, 5, and 1 μM) were added to each

sample, followed by incubation at 4 °C for 8 h. After incubation, the diluted different concentrations of pronase solution (roche) were added to the mixture of cellular protein for digestion of 20 min at 37 °C, and then these samples were used for western blot analysis.

Cellular Thermal Shift Assay (CETSA) Analysis

Total cellular protein of MDA-MB-231 and 4T1 were extracted using RIPA lysis buffer. Next, the protein concentration was determined using a gold bicinchoninic acid (BCA) protein concentration assay kit. Aliquots of 50 μg of protein were taken from the lysate to each 1.5 mL tube, Then, the supernatants were treated with DMSO or PoD (20 μM), and heated for 5 min at different temperatures (50–75 °C) by a thermal cycler. After that, the tubes were kept at room temperature for 3 min and then kept on ice, and then these samples were used for western blot analysis.

Pochonin D and PRDX1 Competitive Ligand Binding Assay

The MDA-MB-231 or 4T1 cell lysates were divided into 3 equal parts, and the DMSO, PoD-biotin, and PoD with PoD-biotin group. The pull-down experiments were performed and detected using western blot.

Enzyme Activity Assay

The inhibitory enzyme activity of PoD on PRDX1-6 protein was performed according to PRDXs ELISA protocol (Hengyuan Biotechnology CO., Ltd, Shanghai, China).

Measurement of ROS

Cells were seeded in 6-well plates and cultured overnight, followed by treatment with PoD for 24 h, with or without NAC and GSH. After treatment, the cells were harvested, washed with serum-free medium, and incubated with DCFH-DA (Molecular Probes, Thermo Fisher Scientific, USA) for 30 minutes at 37 °C in the dark. DCFH-DA was deacetylated by intracellular esterases to a non-fluorescent intermediate, which was oxidized by intracellular ROS subsequently to form the fluorescent DCF. Fluorescence microscopy (Leica) or flow cytometry was then used to detect and quantify ROS levels.

Knockdown and Overexpression of PRDX1

A short interfering RNA (siRNA) targeting the PRDX1 gene in humans and mice was designed and synthesized by GenePharma (Shanghai, China). The sequences of the siRNAs used were as follows: for mouse cells, siRNA-*prdx1*: S-GUAUAUGUGAGGCUAGUAA and AS-UUACUAGCCUCACAUCUCC; for human cells, siRNA-PRDX1: S-AATGCAAAAATT GGGTATCCTGC and AS-CGTGGGACACACAAAAGTAAAGT. MDA-MB-231 or 4T1 cells were plated in six-well plates and cultured to approximately 70 % confluence. The cells were then transfected with 50 nM indicated siRNA or a negative control using Lipofectamine RNAiMAX, and incubated for 48 hours (Thermo Fisher, Logan, UT, USA), following the manufacturer's instructions. The plasmids expressing PRDX1 were transfected using Lipofectamine 2000 in accordance with the manufacturer's guidelines. The PRDX1 KD or OV MDA-MB-231 and 4T1 cells were further used for cell viability, cloning colony, ROS production, cuproptosis related assays.

Animals and Ethical Statement

This study was approved by the Institutional Animal Care and Use Committee of China Pharmaceutical University and Ethics Committee of Shanghai Outdo Biotech Co., Ltd. (SHYJS-CP-2210008). All animal care and experimental procedures were conducted in strict accordance with the principles and procedures approved by the Institutional Animal Care and Use Committee of China Pharmaceutical University. This study was also in accordance with the 3*R*s principles (replacement, refinement and reduction). Animal studies are reported in compliance with the ARRIVE guidelines.

Female BALB/c mice (6-8 weeks old) were obtained from Qinglongshan Animal Breeding Farm (Nanjing, China). Animals were housed in the specific pathogen-free conditions (12 h light/dark cycle at 22 °C) at China Pharmaceutical University and allowed free access to a standard diet and water until initiation of the experiment. Animals were randomized for treatment. Data collection and evaluation of all experiments were performed blindly of the group identity. Cultured 4T1 cells were subcutaneously injected into the right armpit of the mice. After tumor volumes reached 50-100 mm^3, the tumor-bearing animals were randomly

divided into several groups. Vehicle or Pod (40, 20, and 10 mg/kg) was administered to the mice by intraperitoneal injection every other day, CTX (20 mg/kg) group as positive control. Tumor size and body weight were monitored every other day. Tumor size was calculated the following formula: calculated volume (mm^3) = shortest diameter^2 × longest diameter/2. After 18 days, mice were sacrificed by cervical dislocation. Tumor xenografts of each mouse were removed and weighed. Organs (heart, liver, spleen, lung, and kidney) were removed for microscopic examination by haematoxylin-eosin staining. Serum aspartate transaminase (AST), creatine kinase, alkaline phosphate (ALP) and alanine transaminase (ALT) were determined using relevant kits (Jiancheng, Nanjing, China) based on the manufacturer instructions.

PRDX1 KD, PRDX1 OV 4T1 cells or nonmoral 4T1 cells were subcutaneously injected into the both right and left armpit of the mice. Tumor size and body weight were monitored every other day. Tumor size was calculated the following formula: calculated volume (mm^3) = shortest diameter^2 × longest diameter/2. After 20 and 28 days respectively, mice were sacrificed by cervical dislocation. Tumor xenografts of each mouse were removed and weighed.

PRDX1 KD and PRDX1 OV 4T1 cells were subcutaneously injected into the both right armpit of the mice. After tumor volumes reached 50-100 mm^3, the tumor-bearing animals were randomly divided into several groups. Vehicle or Pod (40 or 20 mg/kg) was administered to the mice by intraperitoneal injection every other day. Tumor size and body weight were monitored every other day. Tumor size was calculated the following formula: calculated volume (mm^3) = shortest diameter^2 × longest diameter/2. After 22 days, mice were sacrificed by cervical dislocation. Tumor xenografts of each mouse were removed and weighed.

Safety Statement

No unexpected or unusually high safety hazards were encountered.

Statistical Analysis

All the data were shown as mean ± SD. The student's *t*-test was employed for statistical analysis using GraphPad Prism software. The $p < 0.05$ was considered statistically significant.

References

(1) Masayoshi, A., Kazunobu, Y., Ichiji, N., Hiroshi, T., Satoshi, O. New monordens produced by amidepsine-producing fungus *Humicola* sp. FO-2942. *J. Antibiot.* **2003**, 56, 526–532.

(2) Wicklow, D.T., Joshi, B.K., Gamble, W.R., Gloer, J.B., Dowd, P.F., Antifungal metabolites (monorden, monocillin IV, and cerebrosides) from *Humicola fuscoatra* NRRL 22980, a mycoparasite of *Aspergillus flavus*. *App. Environ. Microb.* 1998, 64, 4482-4484.

(3) Evans, G., White, N.H., Radicicolin and radicicol, two new antibiotics produced by *Cylindrocarpon radicicola*. *Trans. Brit. Myco. Soc.* 1966, 49, 563-567.

(4) Cutler, H.G., Arrendale, R.F., Springer, J.P., Cole, P.D., Roberts, R.G., Hanlin, R.T. Monorden from a novel source, *Neocosmospora tenuicristata*: Stereochemistry and plant growth regulatory properties. *Agric. Biol. Chem.* **1987**, 51. 3331-3338.

(5) El-Elimat, T., Raja, H.A., Day, C.S., Chen, W.L., Swanson, S.M., Oberlies, N.H. Greensporones: resorcylic acid lactones from an aquatic *Halenospora* sp. *J. Nat. Prod.* **2014**, 77, 2088-2098.

(6) Choe, H., Cho, H., Ko, H.J., Lee, J. Total synthesis of (+)-pochonin D and (+)-monocillin ii via chemo-and regioselective intramolecular nitrile oxide cycloaddition. *Org. lett.* **2017**, 19, 6004-6007.

(7) Garbaccio, R.M., Stachel, S.J., Baeschlin, D.K., Danishefsky, S.J. Concise asymmetric syntheses of radicicol and monocillin I. *J. Am. Chem. Soc.* **2011**, 123.10903-10908.

(8) Mejia, E.J., Loveridge, S.T., Stepan, G., Tsai, A., Jones, G.S., Barnes, T., White, K.N., Drašković, M., Tenney, K., Tsiang, M., Geleziunas, R. Study of marine natural products including resorcyclic acid lactones from *Humicola fuscoatra* that reactivate latent HIV-1 expression in an *in vitro* model of central memory $CD4^+$ T cells. *J. Nat. Prod.* **2014**, 77, 618-624.

(9) Hellwig, V., Mayer-Bartschmid, A., Müller, H., Greif, G., Kleymann, G., Zitzmann, W., Tichy, H.V., Stadler, M. Pochonins A− F, new antiviral and antiparasitic resorcylic acid lactones from *Pochonia chlamydosporia. J. Nat. Prod.* **2003**, 66, 829-837.

(10) Qin, F., Li, Y., Lin, R., Zhang, X., Mao, Z., Ling, J., Yang, Y., Zhuang, X., Du, S., Cheng, X., Xie, B. Antibacterial radicicol analogues from *Pochonia chlamydosporia* and their

biosynthetic gene cluster. *J. Agric. Food Chem.* **2019**, 67, 7266-7273.

(11) Shinonaga, H., Kawamura, Y., Ikeda, A., Aoki, M., Sakai, N., Fujimoto, N., Kawashima, A. Pochonins K–P: new radicicol analogues from *Pochonia chlamydosporia* var. *chlamydosporia* and their WNT-5A expression inhibitory activities. *Tetrahedron* **2009**, 65, 3446-3453.

(12) Dai, J., Krohn, K., Flörke, U., Pescitelli, G., Kerti, G., Papp, T., Kövér, K.E., Bényei, A.C., Draeger, S., Schulz, B., Kurtán, T. Curvularin-type metabolites from the fungus *Curvularia* sp. isolated from a marine alga. *Eur. J. Org. Chem.* **2010**, 5, 1175-1180.

(13) O'Boyle, N., Banck, M. M., James, C., Morley, A. C., Vandermeersch, T., Hutchison, G. R. Open babel: an open chemical toolbox. *J. Cheminformatics* **2011**, 3, 1758-1762.

(14) Frisch, M. J., Trucks, G. W., Schlegel, H. B., Scuseria, G. E., Robb, M. A., Cheeseman, J. R., Scalmani, G., Barone, V., Mennucci, B., Petersson, G. A., Nakatsuji, H., Caricato, M., Li, X., Hratchian, H. P., Izmaylov, A. F., Bloino, J., Zheng, G., Sonnenberg, J. L., Hada, M., Fukuda, R., Hasegawa, J., Ishida, M., Nakajima, T., Honda, Y., Kitao, O., Nakai, H., Vreven, T., Montgomery, J. A., Ogliaro, F., Bearpark, M., Heyd, J. J., Brothers, E., Kudin, K. N., Staroverov, V. N., Keith, T., Kobayashi, R., Normand, J., Raghavachari, K., Rendell, A., Burant, J. C., Iyengar, S. S., Tomasi, J., Cossi, M., Rega, N., Millam, J. M., Klene, M., Knox, J. E. J., Cross, B., Bakken, V., Adamo, C., Jaramillo, J., Gomperts, R., Stratmann, R. E., Yazyev, O., Austin, A. J., Cammi, R., Pomelli, C., Ochterski, J. W., Martin, R. L., Morokuma, K., Zakrzewski, V. G., Voth, G. A., Salvador, P., Dannenberg, J. J., Dapprich, S., Daniels, A. D., Farkas, O., Foresman, J. B., Ortiz, J. V., Cioslowski, J., Fox, D. J. Gaussian 09, revision C.01, Gaussian, Inc. Wallingford CT, 2010.

(15) Bruhn, T., Schaumlffl, A., Hemberger, Y., Bringmann, G. SpecDis: quantifying the comparison of calculated and experimental electronic circular dichroism spectra. *Chirality* **2013**, 25, 243-249.

(16) Lodewyk, M. W., Siebert, M. R., Tantillo, D. J. Computational prediction of ^{1}H and ^{13}C chemical shifts: a useful tool for natural product, mechanistic, and synthetic organic chemistry. *Chem. Rev.* **2012**, 112, 1839-1862.

Figure S17 ^{1}H NMR spectrum (400 MHz, CD3OD) of ilyolactone A (**1**)

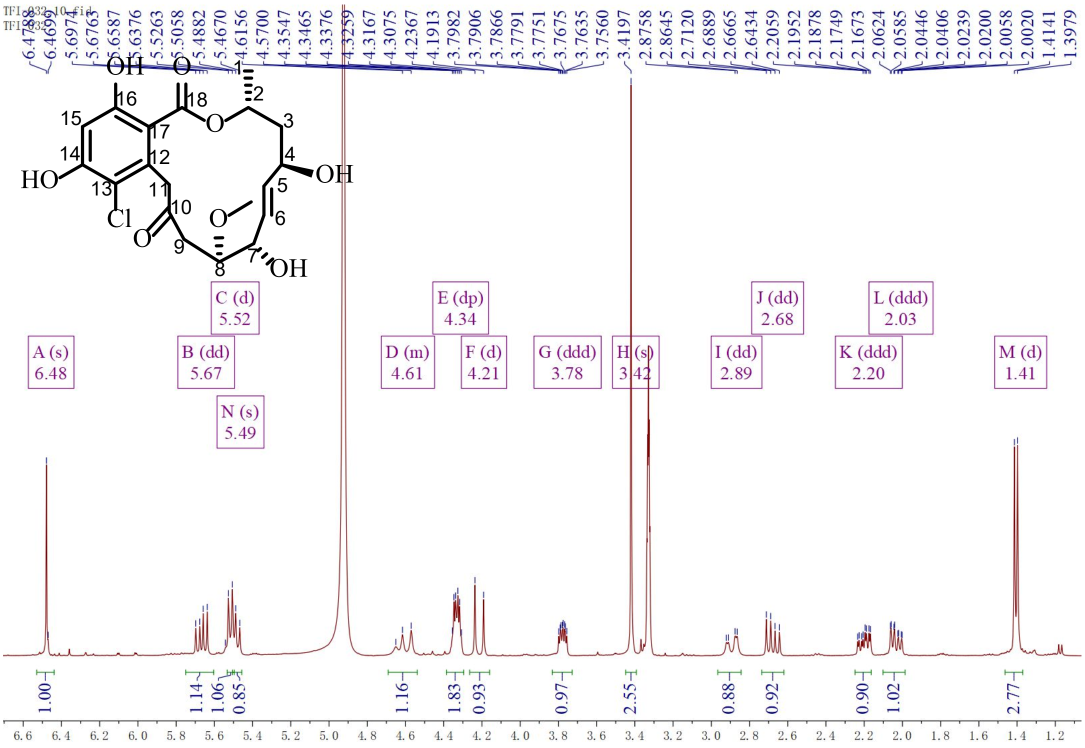

Figure S18 ^{13}C NMR spectrum (400 MHz, CD3OD) of ilyolactone A (**1**)

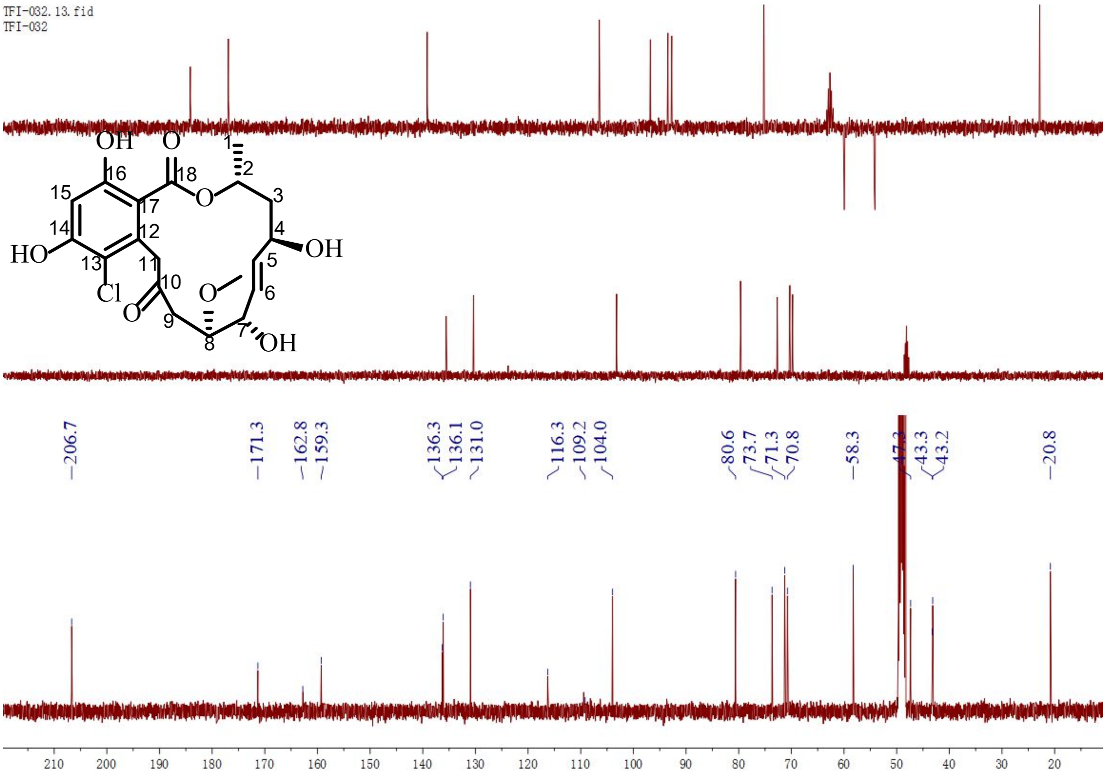

Figure S19 HSQC spectrum (400 MHz, CD_3OD) of ilyolactone A (**1**)

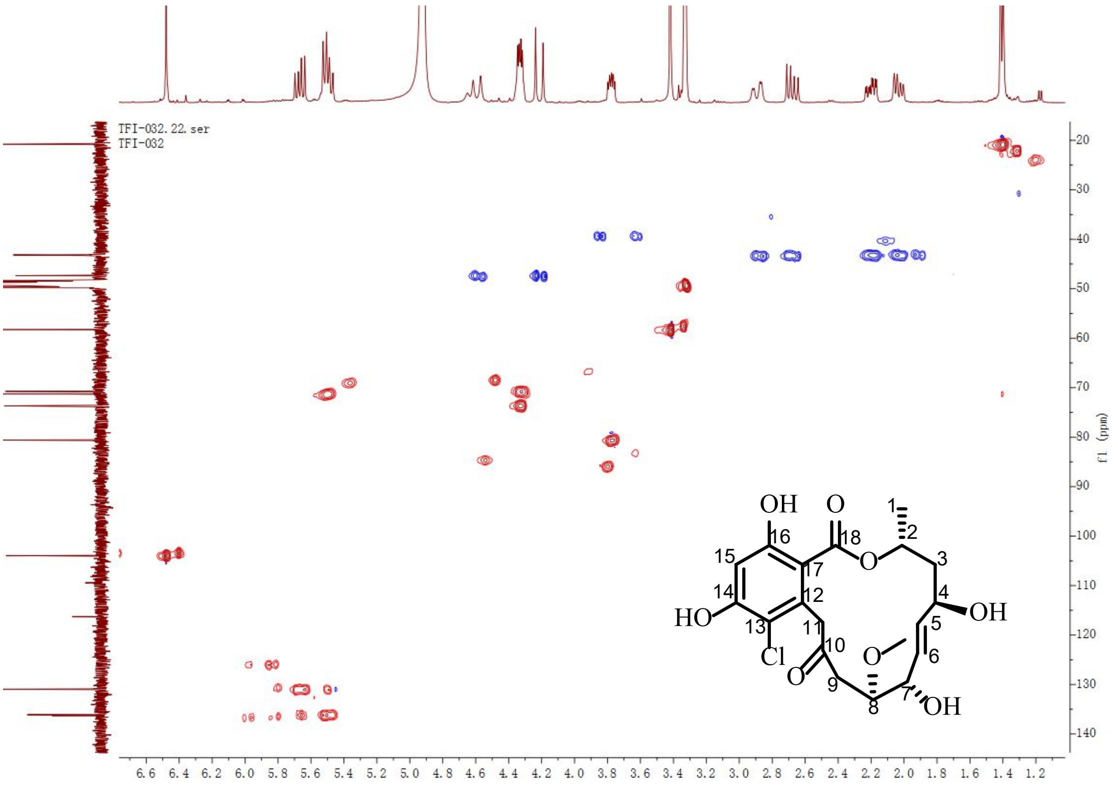

Figure S20 ^1H-^1H COSY spectrum (400 MHz, CD_3OD) of ilyolactone A (**1**)

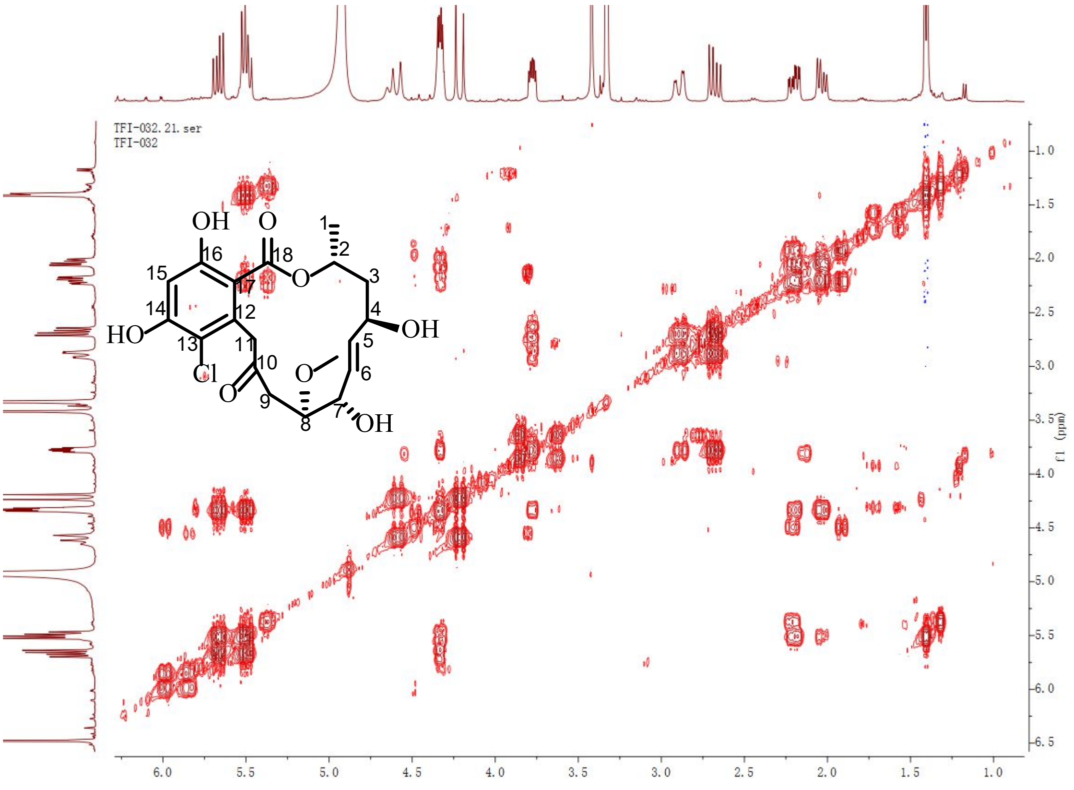

Figure S21 HMBC spectrum (400 MHz, CD_3OD) of ilyolactone A (**1**)

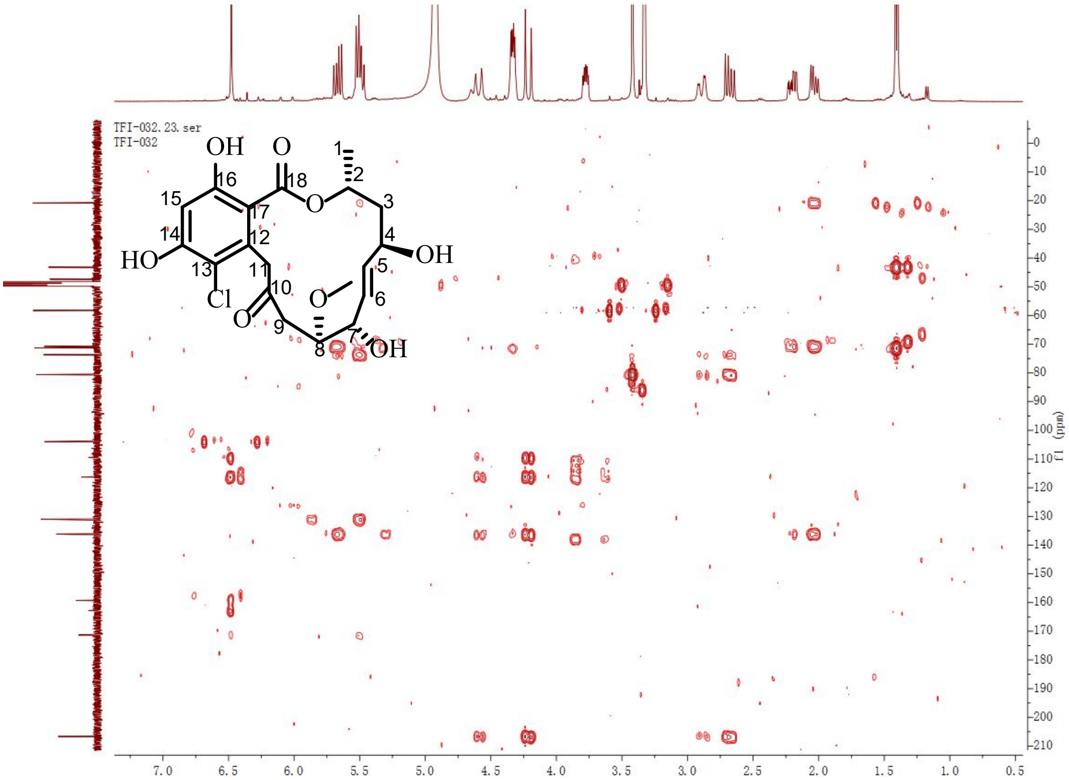

Figure S22 ROESY spectrum (400 MHz, CD_3OD) of ilyolactone A (**1**)

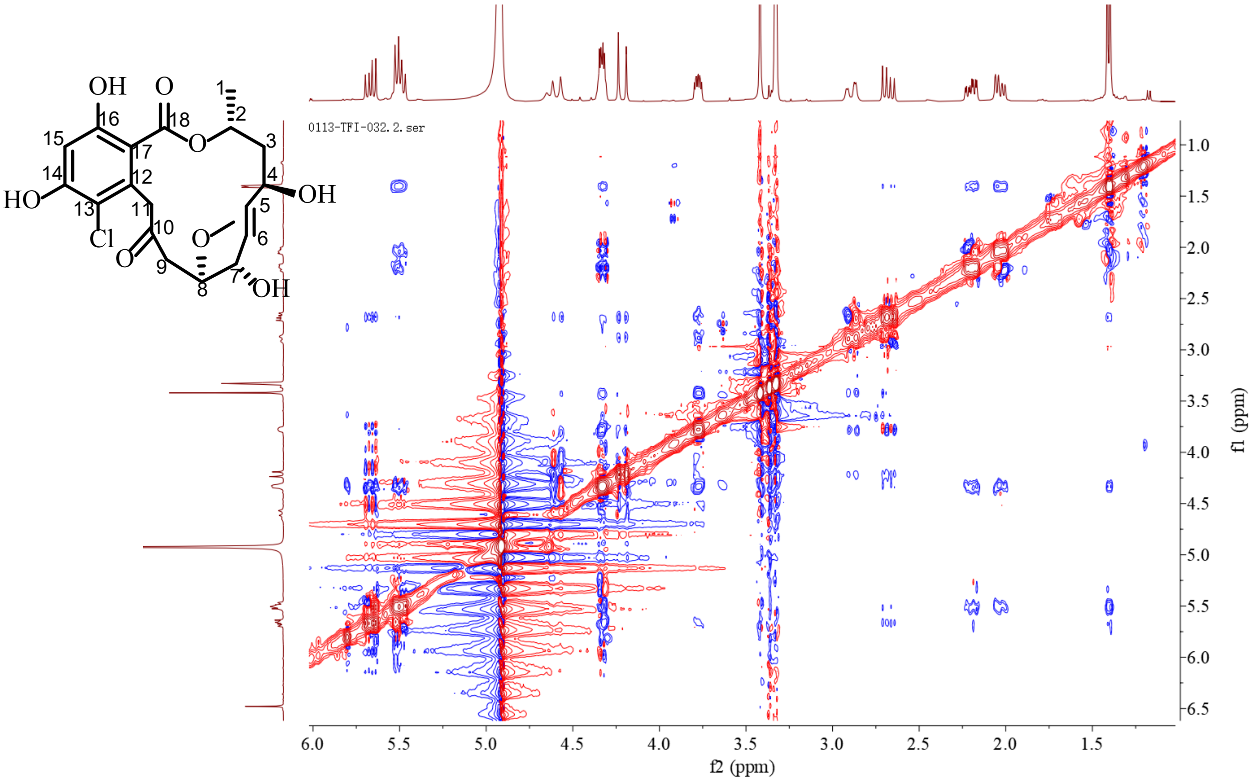

Figure S23 ESIMS spectrum of ilyolactone A (**1**)

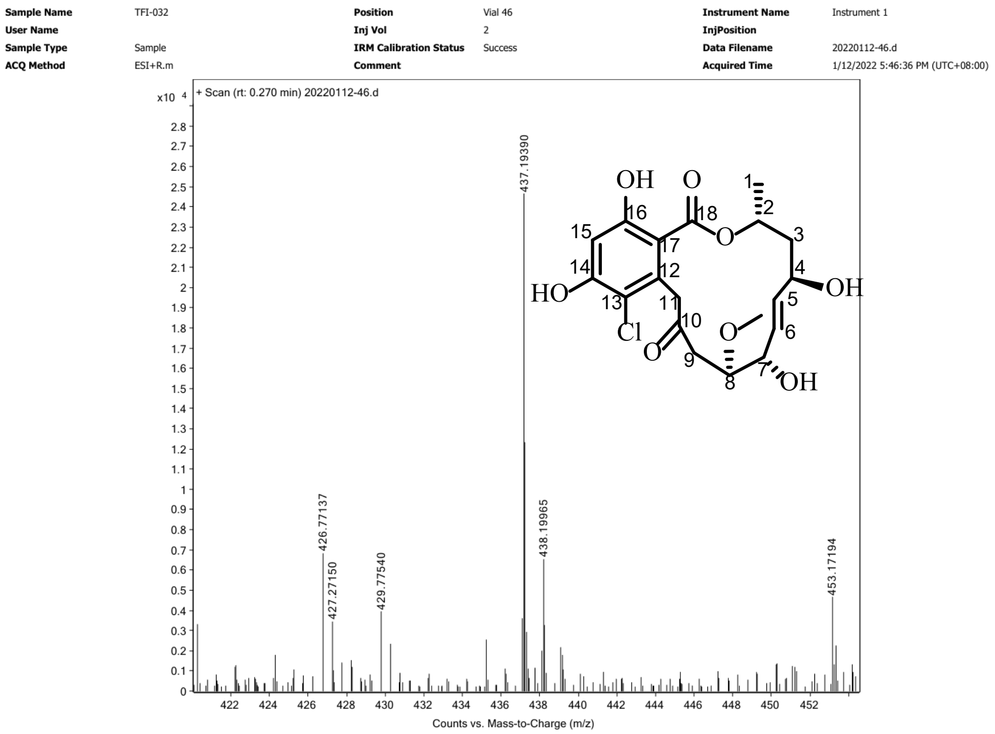

Figure S24 IR spectrum of ilyolactone A (**1**)

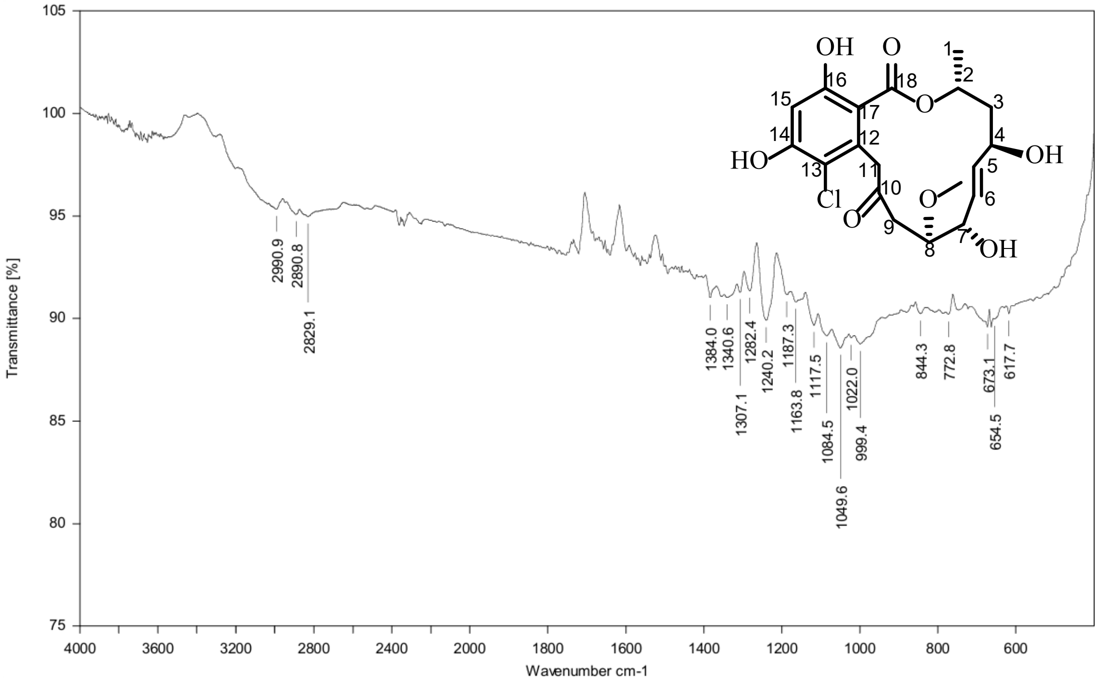

Sample Name: TFI-032

Experiment: MIR_TR.xpm

Operator Name: CPU

Instrument Type: Bruker Tensor 27

Path of File: E:\OPUS\MEAS

Date of Measurement: 13/01/2022

Laboratory: China Pharmaceutical University

Figure S25 HRESIMS spectrum of ilyolactone A (**1**)

Qualitative Compound Identification Report

**Data File**	20220112-46.d	**Sample Name**	TFI-032
**Sample Type**	Sample	**Position**	Vial 46
**Instrument Name**	Instrument 1	**User Name**	
**Acq Method**	ESI+R.m	**Acquired Time**	1/12/2022 5:46:36 PM (UTC+08:00)
**IRM Calibration Status**	Success	**DA Method**	1.m
**Comment**			

**Sample Group**		**Info.**	
**Stream Name**	LC 1	**Acquisition Time (Local)**	1/12/2022 5:46:36 PM (UTC+08:00)
**Acquisition SW Version**	6200 series TOF/6500 series Q-TOF B.06.01 (B6157)	**TOF Driver Version**	6.00.01
**TOF Firmware Version**	17.643		

MS Spectrum

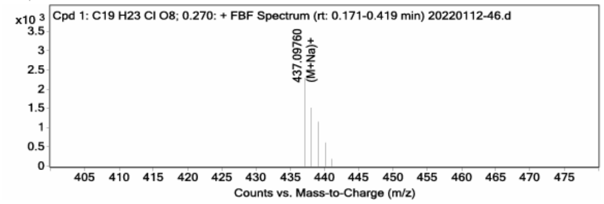

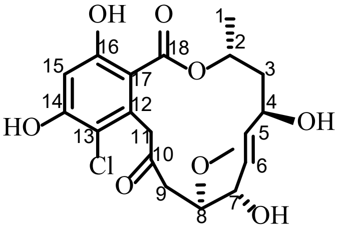

MS Spectrum Peak List

m/z	z	Abund	Ion
437.0976	1	2292.61	(M+Na)+
438.10331	1	1518.36	(M+Na)+
439.09982	1	1156.68	(M+Na)+
440.10259	1	609.43	(M+Na)+
441.10736	1	187.26	(M+Na)+

MS Spectrum

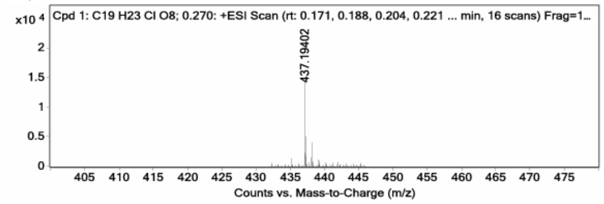

MS Spectrum Peak List

Qualitative Compound Identification Report

m/z	*Calc m/z*	Diff(ppm)	z	Abund	Ion
437.0976	437.09737	-0.54	1	2292.61	(M+Na)+
438.10331	438.10077	-5.8	1	1518.36	(M+Na)+
439.09982	439.09533	-10.24	1	1156.68	(M+Na)+
440.10259	440.09835	-9.64	1	609.43	(M+Na)+
441.10736	441.10044	-15.67	1	187.26	(M+Na)+

--- End Of Report ---

Figure S26 UV spectrum of ilyolactone A (**1**)

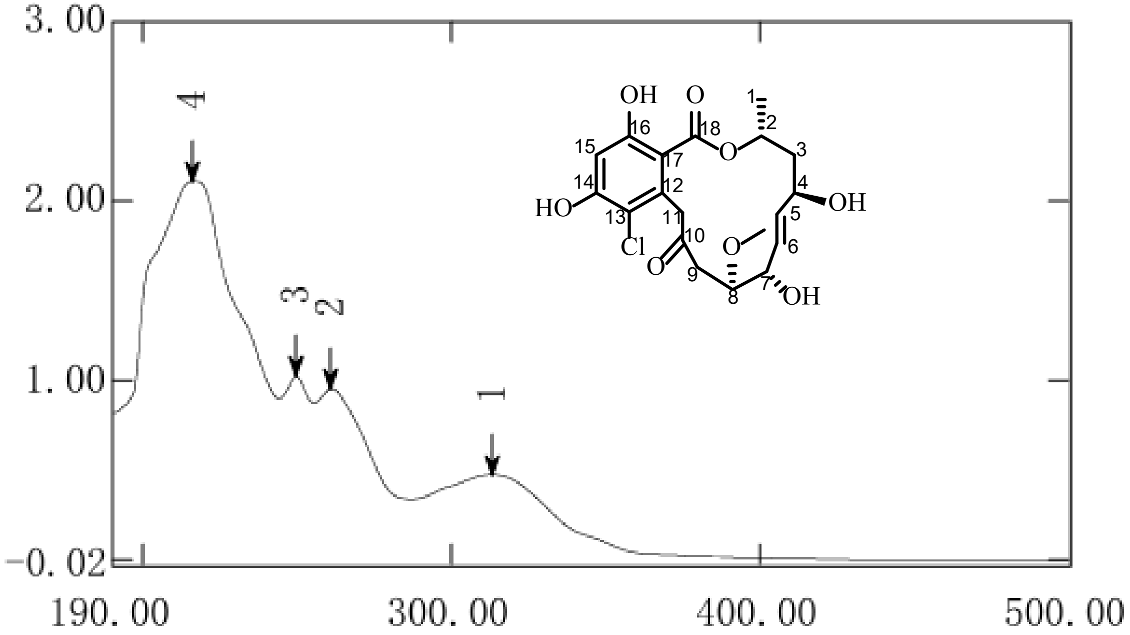

Figure S27 Optical rotation data of ilyolactone A (**1**)

Optical rotation test report

**Sample number**	TFI-032	**weight**	
**Inspection department**	School of Traditional Chinese Pharmacy		
**Inspection item**	Optical rotation		
**Date received**	2022-01-11	**Report data**	2022-01-14

According to the Chinese Pharmacopoeia (2020) of the fourth general rule 0621 spectrophotometric method:

Instrument: AUTOPOL-IV

Light source: halogen tungsten lamp

Wave length: 589 nm

Solvent: Methanol

Temperature: 20°C

Result: $[\alpha]_D^{20}$-30.0 (c=0.04)

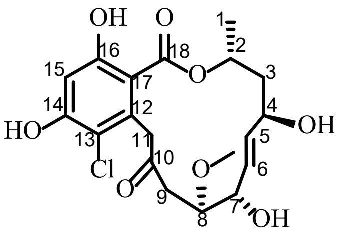

Notes: 1. Inspection report is only responsible for incoming samples

2. Additions, deletions and corrections to this report are invalid

Figure S28 ^{1}H NMR spectrum (400 MHz, DMSO-d_6) of ilyolactone B (**2**)

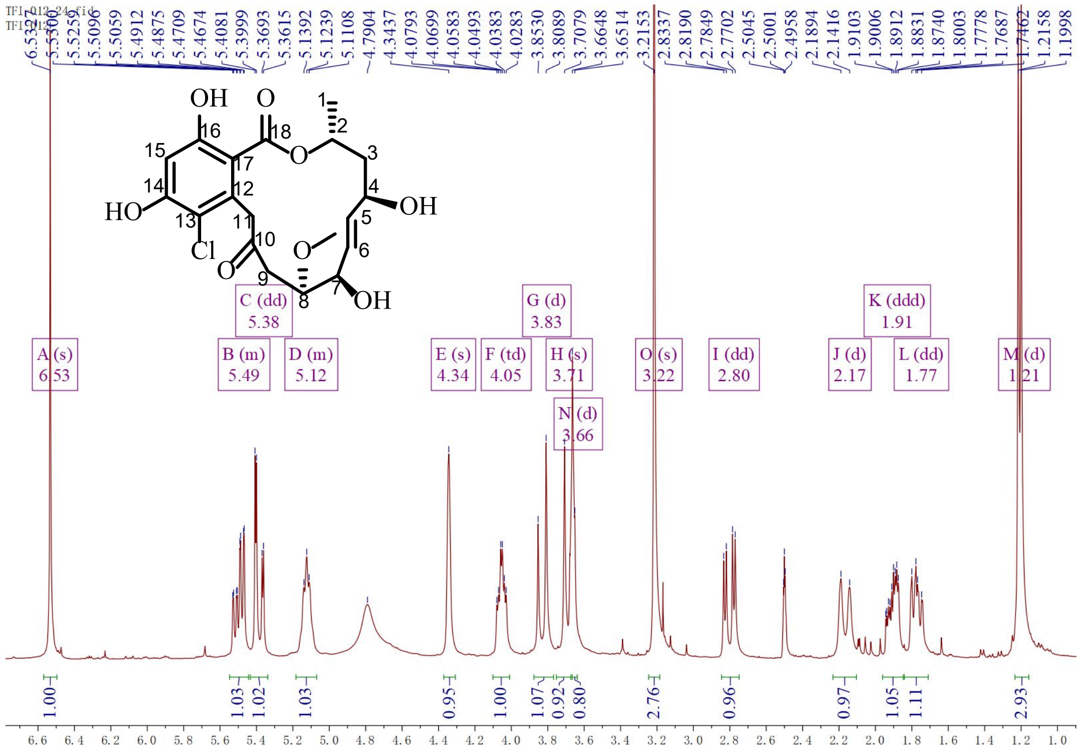

Figure S29 ^{13}C NMR spectrum (400 MHz, DMSO-d_6) of ilyolactone B (**2**)

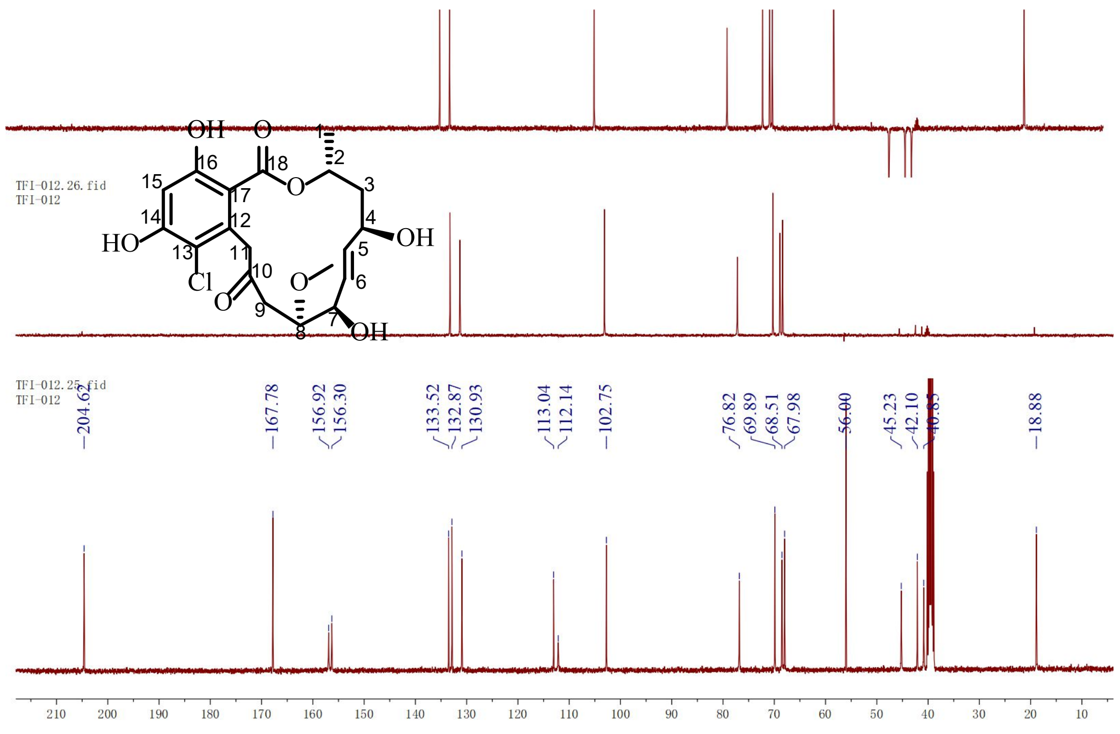

Figure S30 HSQC spectrum (400 MHz, DMSO-d_6) of ilyolactone B (**2**)

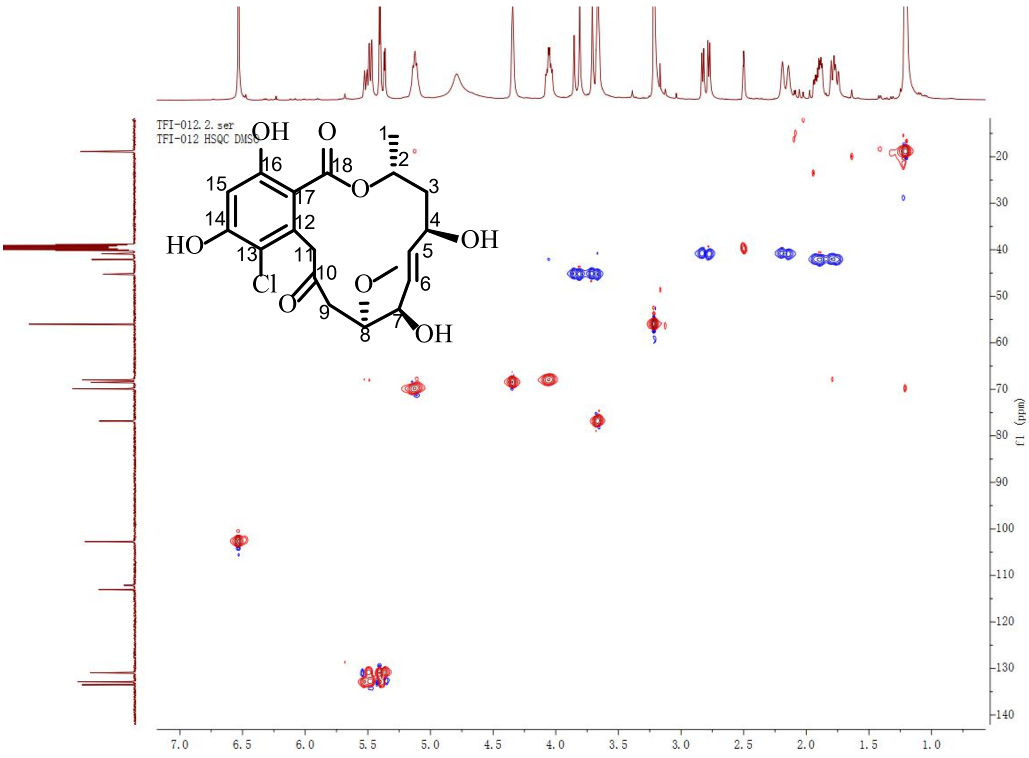

Figure S31 ^{1}H-^{1}H COSY spectrum (400 MHz, DMSO-d_6) of ilyolactone B (**2**)

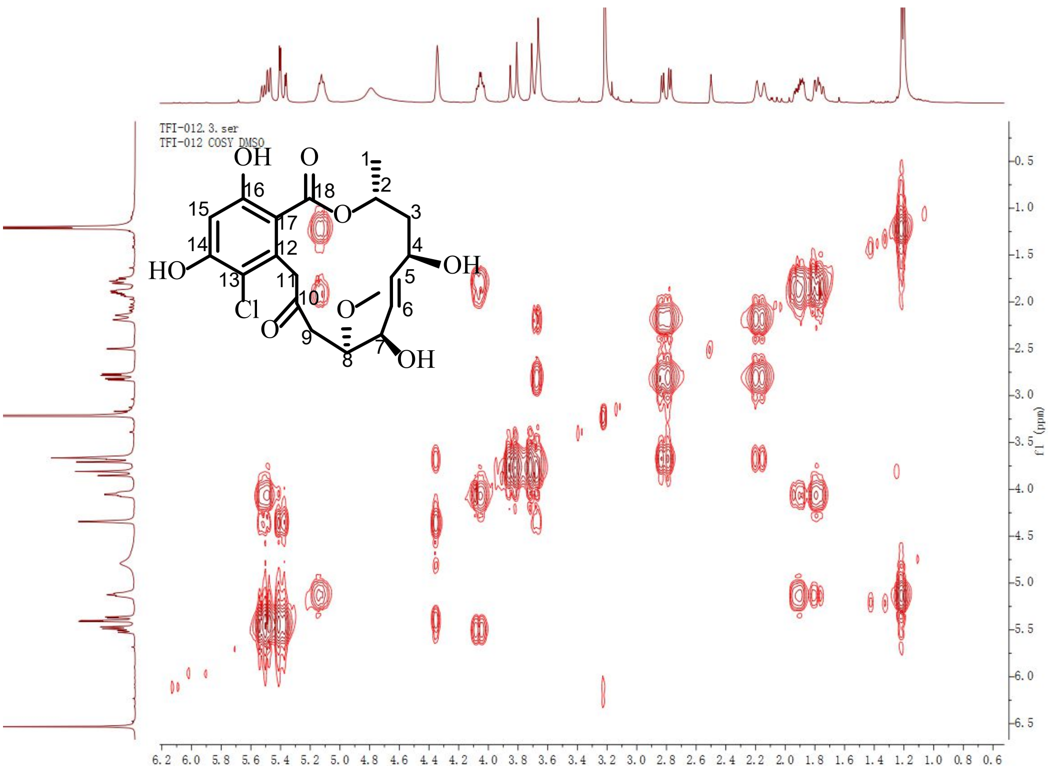

Figure S32 HMBC spectrum (400 MHz, DMSO-d_6) of ilyolactone B (**2**)

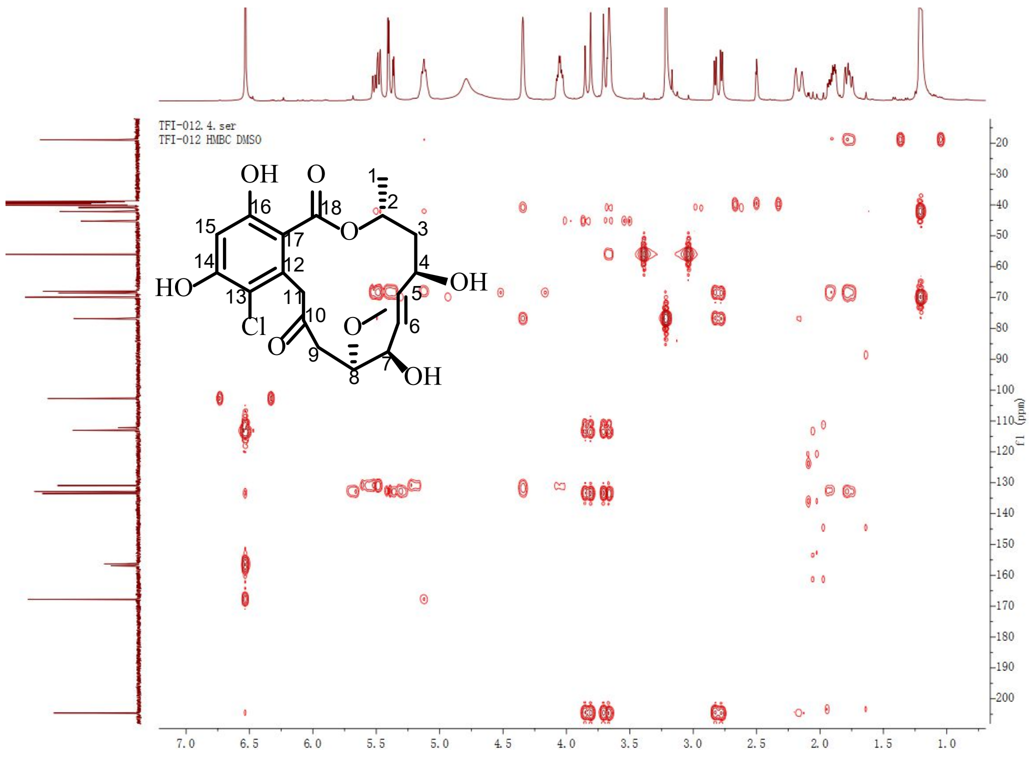

Figure S33 ROESY spectrum (400 MHz, DMSO-d_6) of ilyolactone B (**2**)

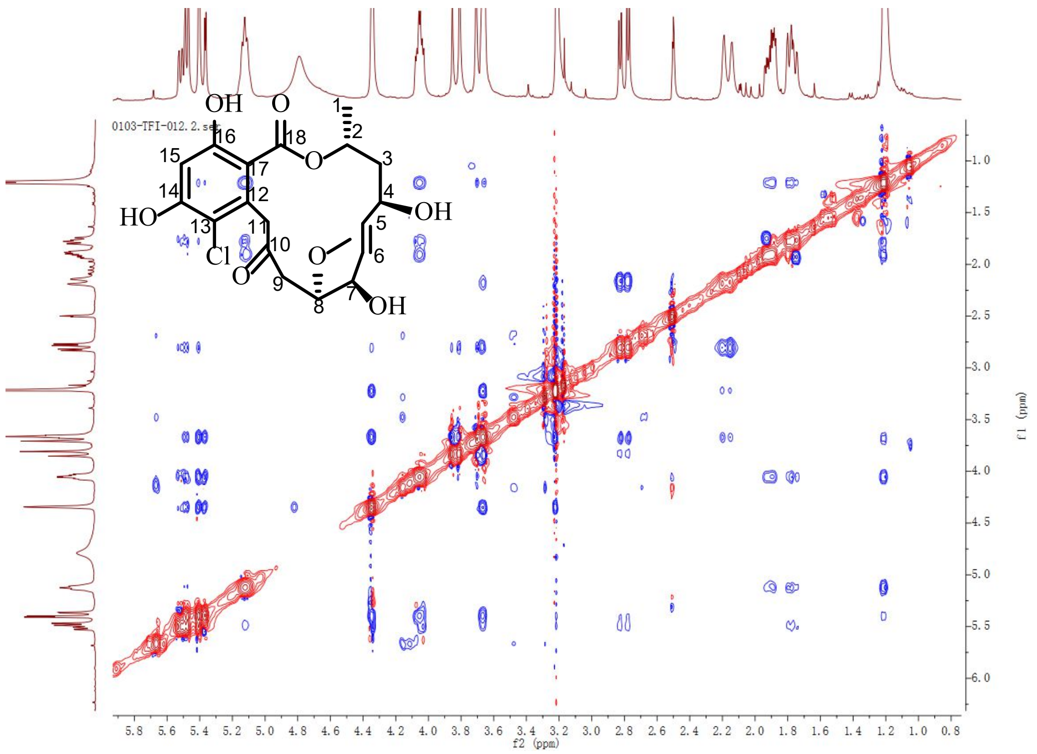

Figure S34 ESIMS spectrum of ilyolactone B (**2**)

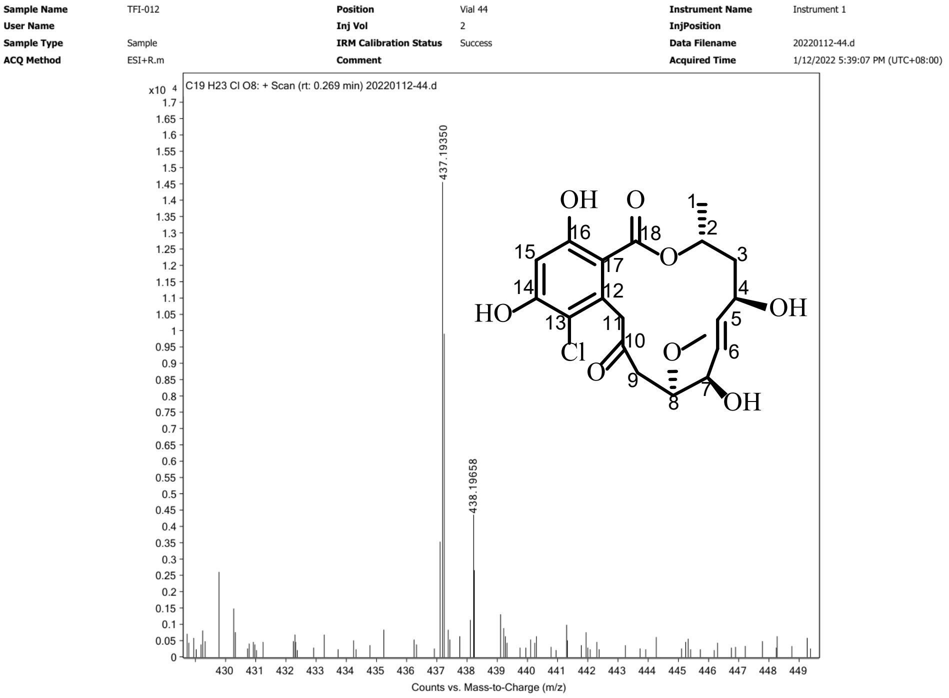

Figure S35 IR spectrum of ilyolactone B (**2**)

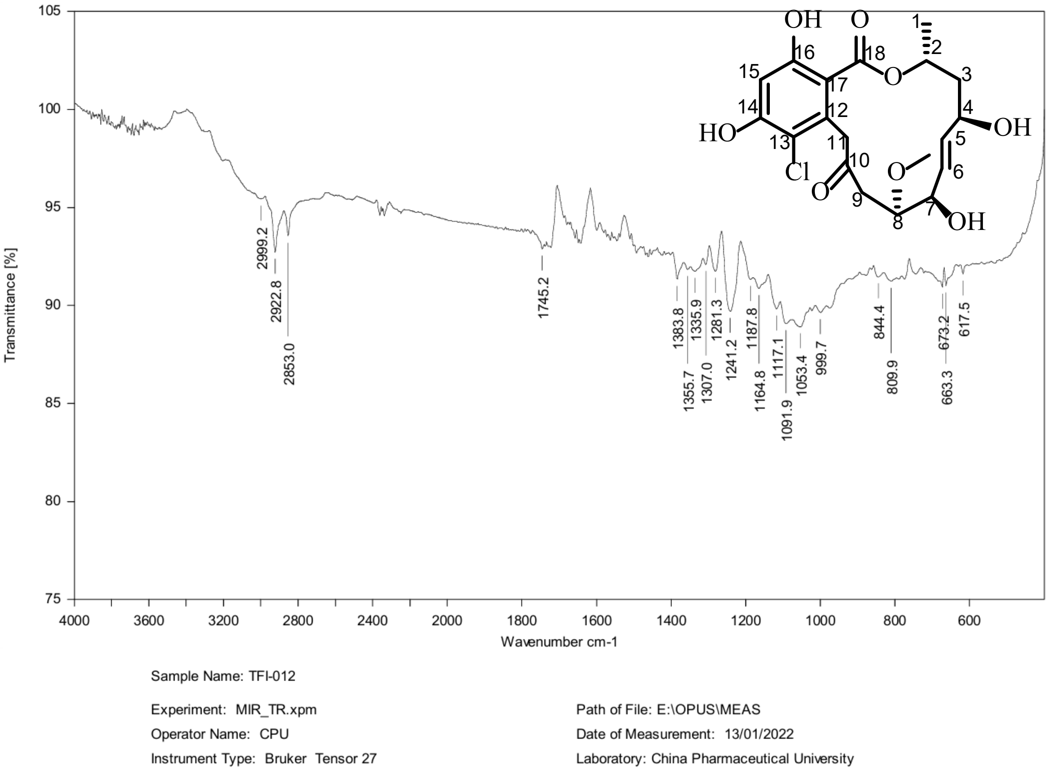

Figure S36 HRESIMS spectrum of ilyolactone B (**2**)

Qualitative Analysis Report

**Data Filename**	20220112-44.d	**Sample Name**	TFI-012
**Sample Type**	Sample	**Position**	Vial 44
**Instrument Name**	Instrument 1	**User Name**	
**Acq Method**	ESI+R.m	**Acquired Time**	1/12/2022 5:39:07 PM (UTC+08:00)
**IRM Calibration Status**	Success	**DA Method**	1.m
**Comment**			

**Sample Group**		**Info.**	
**Stream Name**	LC 1	**Acquisition Time (Local)**	1/12/2022 5:39:07 PM (UTC+08:00)
**Acquisition SW Version**	6200 series TOF/6500 series Q-TOF B.06.01 (B6157)	**TOF Driver Version**	6.00.01
**TOF Firmware Version**	17.643		

Spectra

Collision Energy	Ionization Mode
0	ESI

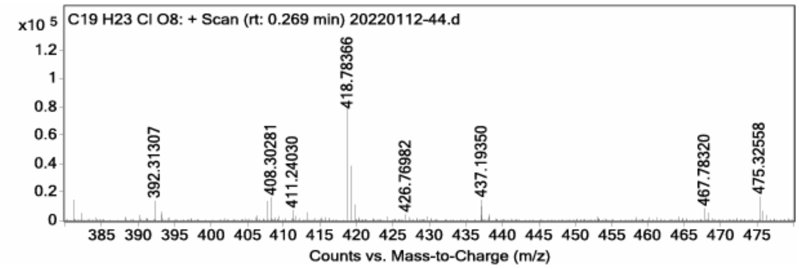

Peak List

m/z	z	Abund
107.04131		54940.8
360.32346	1	348528.38
361.32706	1	70336.31
418.78366	2	80693.74
701.49265	1	51019.79

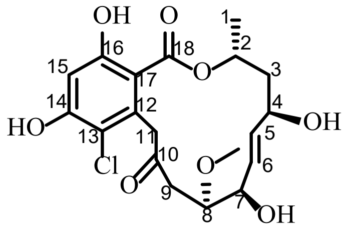

Formula Calculator Element Limits

Element	Min	Max
C	15	25
H	20	26
O	6	10
Cl	0	2

Formula Calculator Results

Formula	Best	Mass	Tgt Mass	Diff (ppm)	Ion Species	CalculatedMz
C19 H23 Cl O8	TRUE	414.10715	414.10815	2.39	C19 H23 Cl Na O8	437.09737

--- End Of Report ---

Figure S37 UV spectrum of ilyolactone B (**2**)

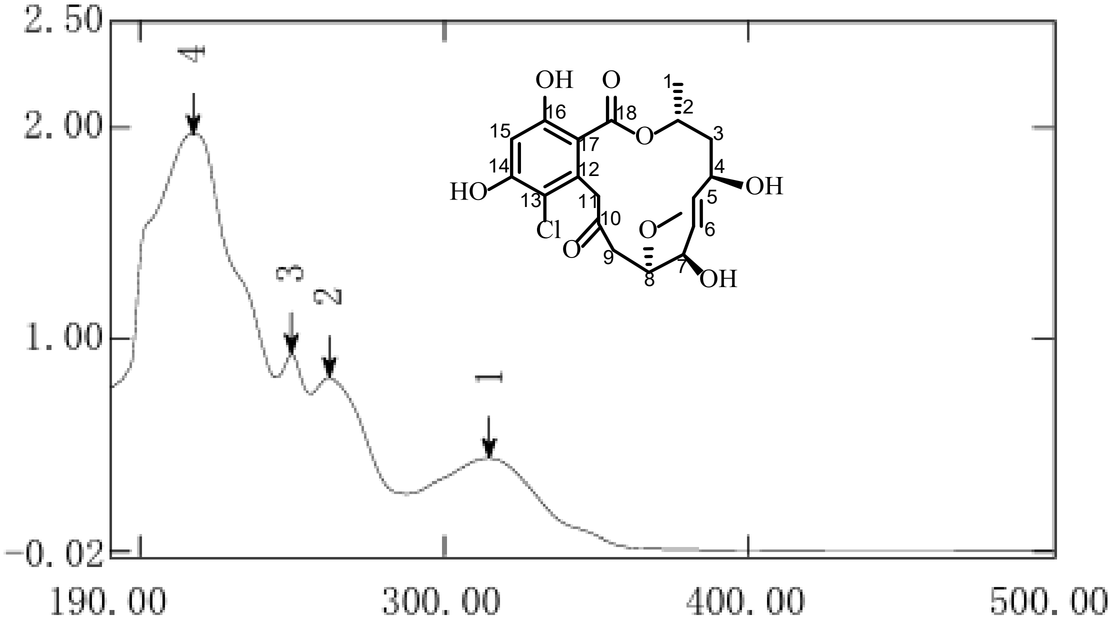

Figure S38 Optical rotation data of ilyolactone B (**2**)

Optical rotation test report

**Sample number**	TFI-012	**weight**	
**Inspection department**	School of Traditional Chinese Pharmacy		
**Inspection item**	Optical rotation		
**Date received**	2022-01-11	**Report data**	2022-01-14

According to the Chinese Pharmacopoeia (2020) of the fourth general rule 0621 spectrophotometric method:

Instrument: AUTOPOL-IV

Light source: halogen tungsten lamp

Wave length: 589 nm

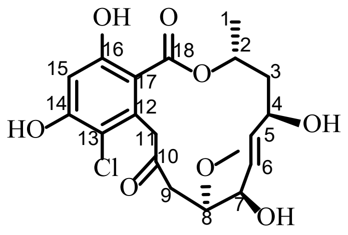

Solvent: Methanol

Temperature: 20°C

Result: $[\alpha]_D^{20}$-15.0 (c=0.04)

Notes: 1. Inspection report is only responsible for incoming samples

2. Additions, deletions and corrections to this report are invalid

Figure S39 ^{1}H NMR spectrum (400 MHz, CD_3OD) of ilyolactone C (**3**)

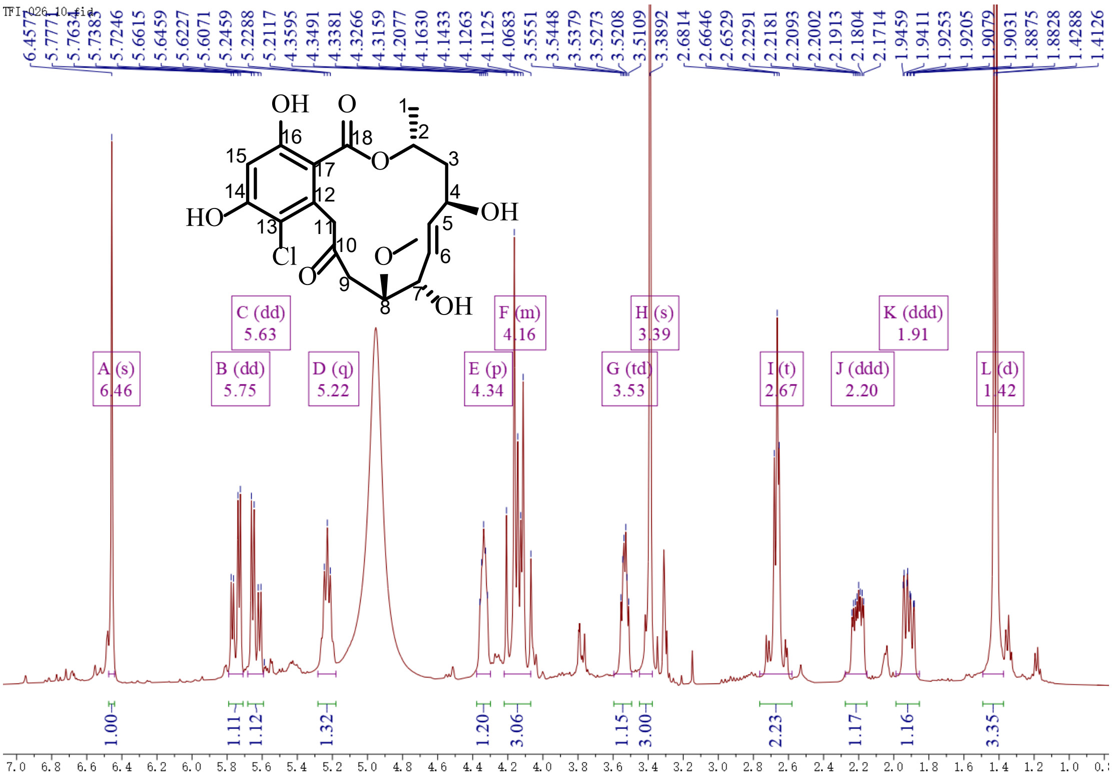

Figure S40 ^{13}C NMR spectrum (400 MHz, CD_3OD) of ilyolactone C (**3**)

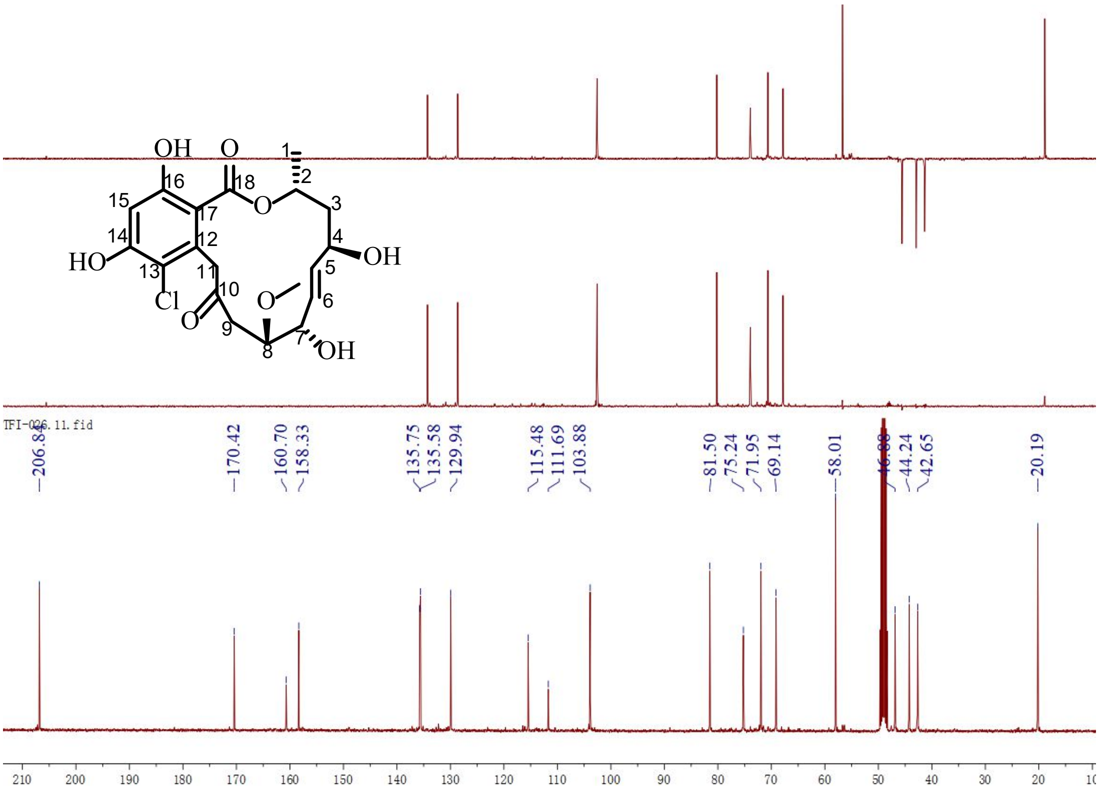

Figure S41 HSQC spectrum (400 MHz, CD_3OD) of ilyolactone C (**3**)

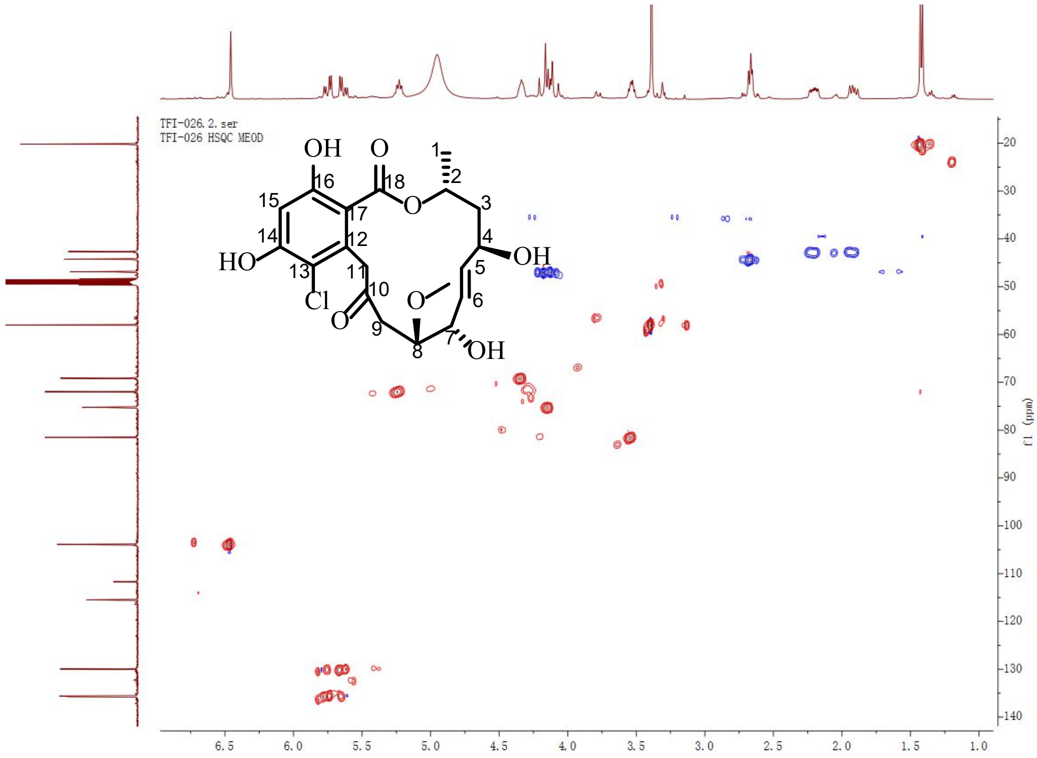

Figure S42 ^1H-^1H COSY spectrum (400 MHz, CD_3OD) of ilyolactone C (**3**)

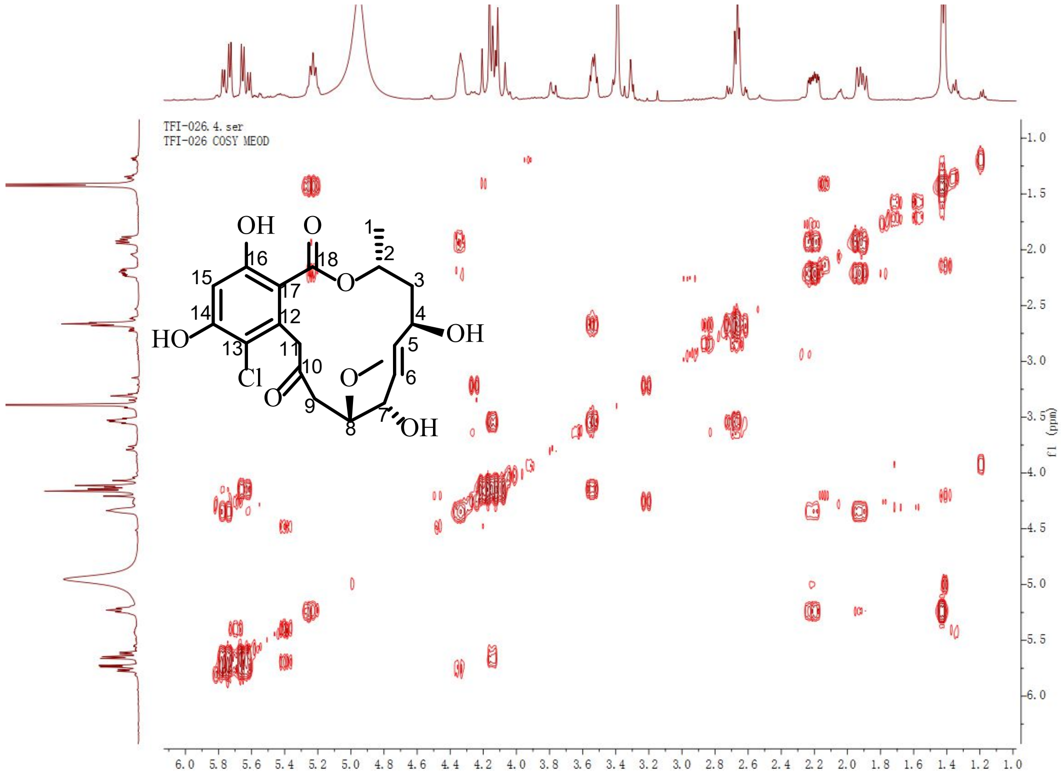

Figure S43 HMBC spectrum (400 MHz, CD_3OD) of ilyolactone C (**3**)

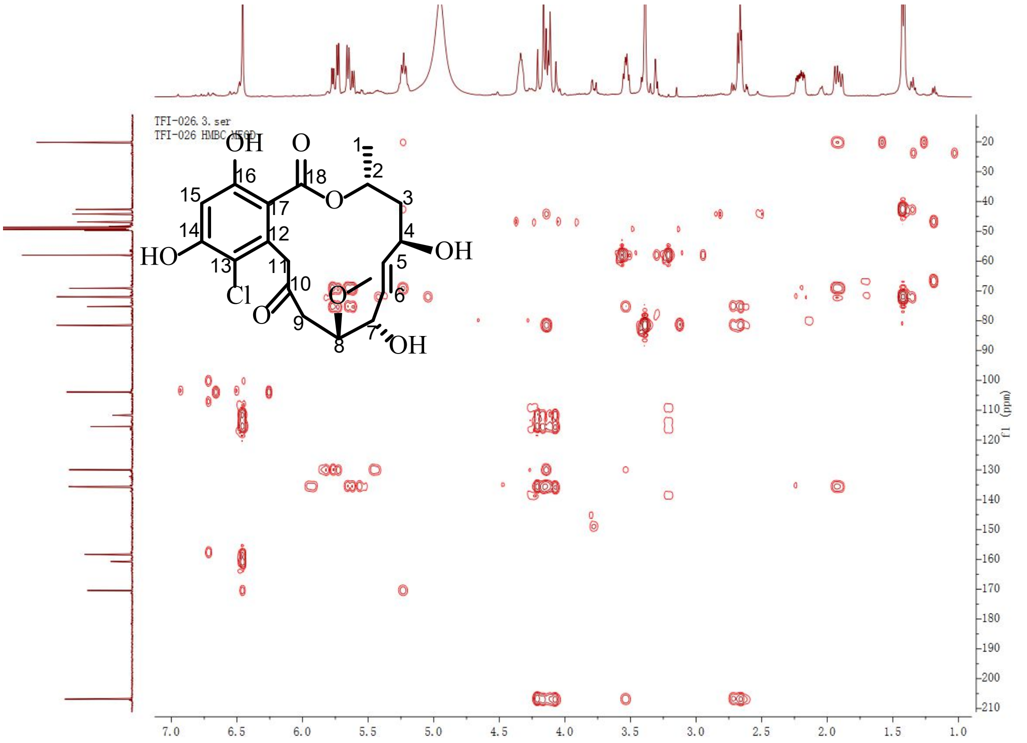

Figure S44 ROESY spectrum (400 MHz, CD_3OD) of ilyolactone C (**3**)

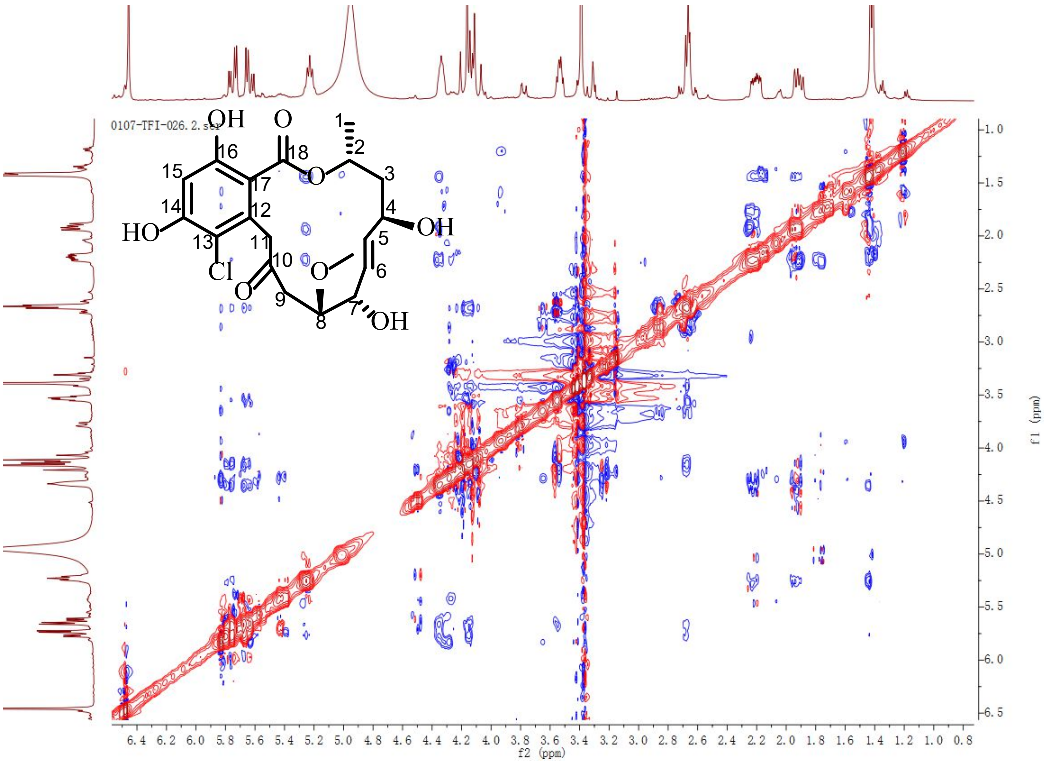

Figure S45 ESIMS spectrum of ilyolactone C (**3**)

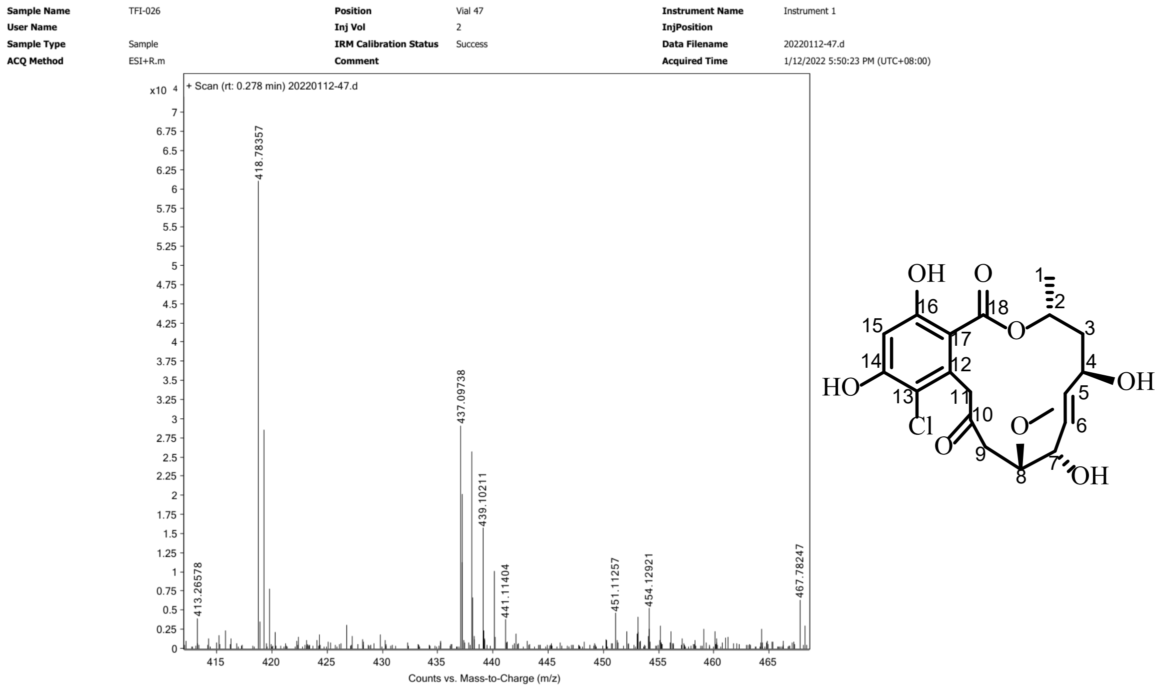

Figure S46 IR spectrum of ilyolactone C (**3**)

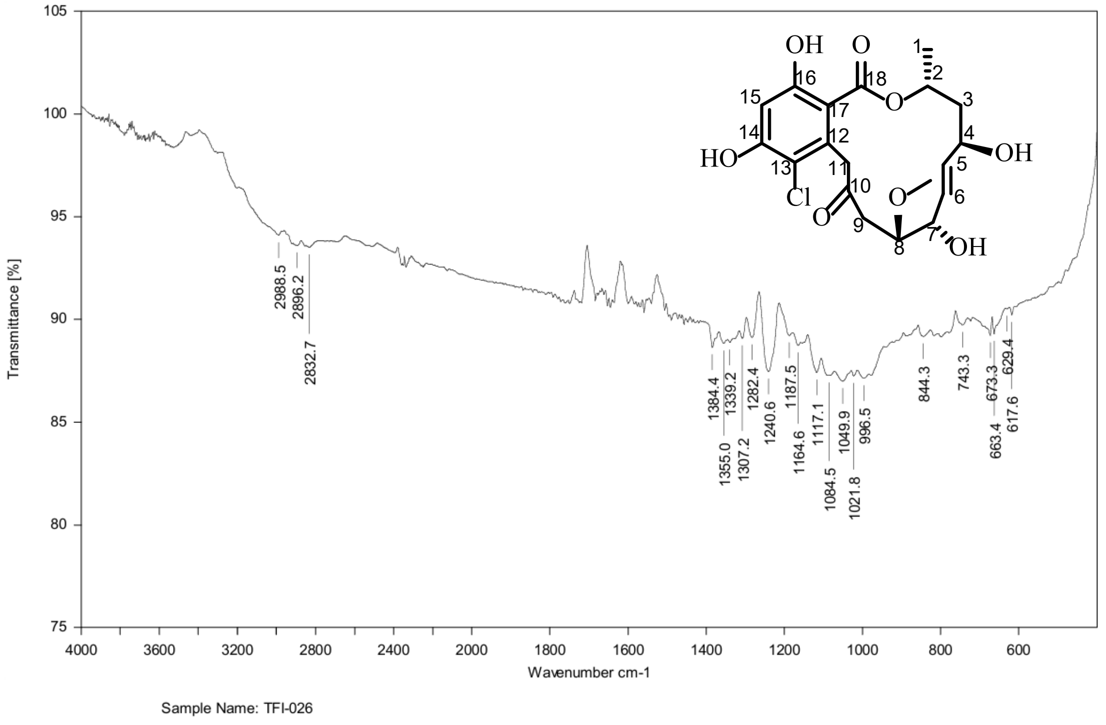

Sample Name: TFI-026

Experiment: MIR_TR.xpm
Operator Name: CPU
Instrument Type: Bruker Tensor 27

Path of File: E:\OPUS\MEAS
Date of Measurement: 13/01/2022
Laboratory: China Pharmaceutical University

Figure S47 HRESIMS spectrum of ilyolactone C (**3**)

Qualitative Compound Identification Report

**Data File**	20220112-45.d	**Sample Name**	TFI-026
**Sample Type**	Sample	**Position**	Vial 45
**Instrument Name**	Instrument 1	**User Name**	
**Acq Method**	ESI+R.m	**Acquired Time**	1/12/2022 5:42:53 PM (UTC+08:00)
**IRM Calibration Status**	Success	**DA Method**	1.m
**Comment**			

**Sample Group**		**Info.**	
**Stream Name**	LC 1	**Acquisition Time (Local)**	1/12/2022 5:42:53 PM (UTC+08:00)
**Acquisition SW Version**	6200 series TOF/6500 series Q-TOF B.06.01 (B6157)	**TOF Driver Version**	6.00.01
**TOF Firmware Version**	17.643		

MS Spectrum

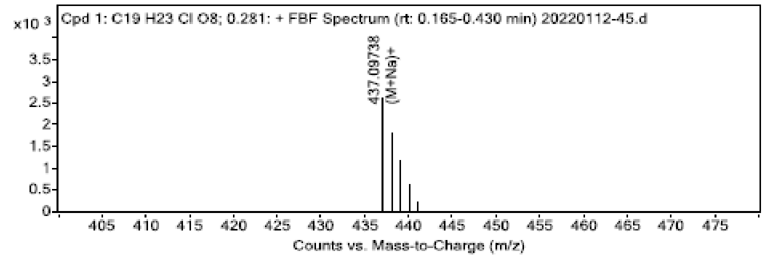

MS Spectrum Peak List

m/z	z	Abund	Ion
437.09738	1	2601.24	(M+Na)+
438.10243	1	1781.72	(M+Na)+
439.09947	1	1187.05	(M+Na)+
440.10211	1	621.14	(M+Na)+
441.10295	1	194.28	(M+Na)+

MS Spectrum

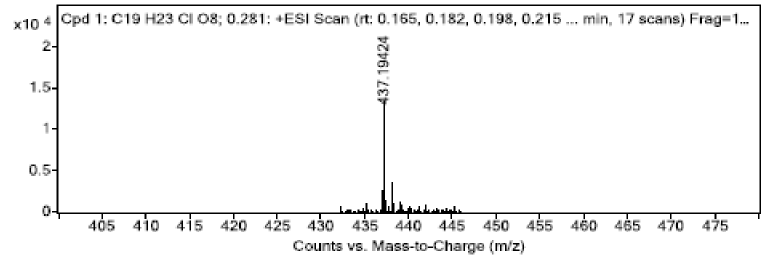

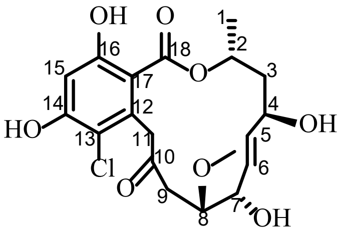

MS Spectrum Peak List

Qualitative Compound Identification Report

m/z	*Calc m/z*	Diff(ppm)	z	Abund	Ion
437.09738	437.09737	-0.04	1	2601.24	(M+Na)+
438.10243	438.10077	-3.79	1	1781.72	(M+Na)+
439.09947	439.09533	-9.43	1	1187.05	(M+Na)+
440.10211	440.09835	-8.55	1	621.14	(M+Na)+
441.10295	441.10044	-5.67	1	194.28	(M+Na)+

--- End Of Report ---

Figure S48 UV spectrum of ilyolactone C (**3**)

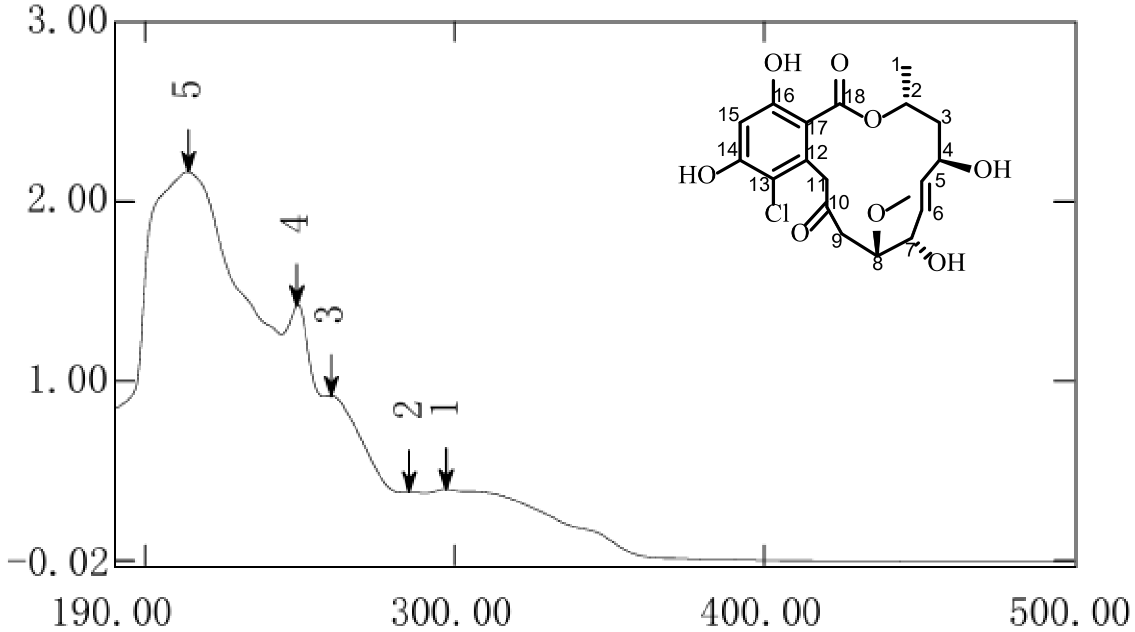

Figure S49 Optical rotation data of ilyolactone C (**3**)

Optical rotation test report

**Sample number**	TFI-026	**weight**	
**Inspection department**	School of Traditional Chinese Pharmacy		
**Inspection item**	Optical rotation		
**Date received**	2022-01-11	**Report data**	2022-01-14

According to the Chinese Pharmacopoeia (2020) of the fourth general rule 0621 spectrophotometric method:

Instrument: AUTOPOL-IV

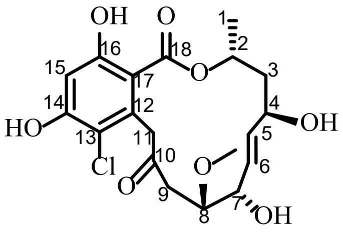

Light source: halogen tungsten lamp

Wave length: 589 nm

Solvent: Methanol

Temperature: 20°C

Result: $[\alpha]_D^{20}$-20.0 (c=0.04)

Notes: 1. Inspection report is only responsible for incoming samples

2. Additions, deletions and corrections to this report are invalid

Figure S50 ^{1}H NMR spectrum (400 MHz, DMSO-d_6) of ilyolactone D (**4**)

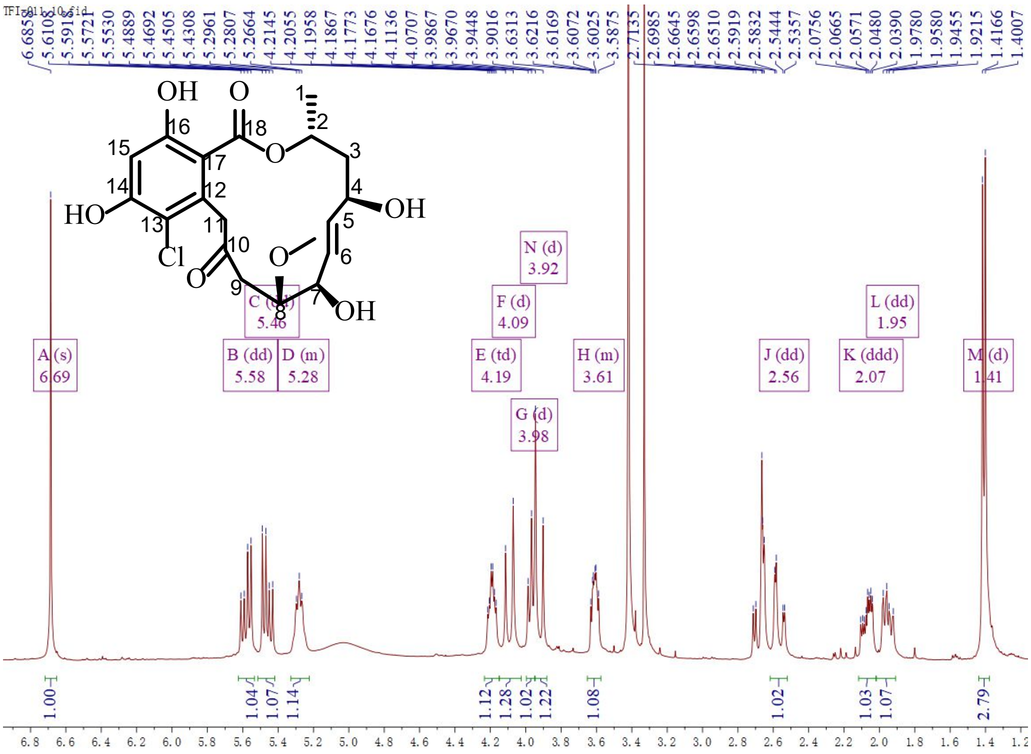

Figure S51 ^{13}C NMR spectrum (400 MHz, DMSO-d_6) of ilyolactone D (**4**)

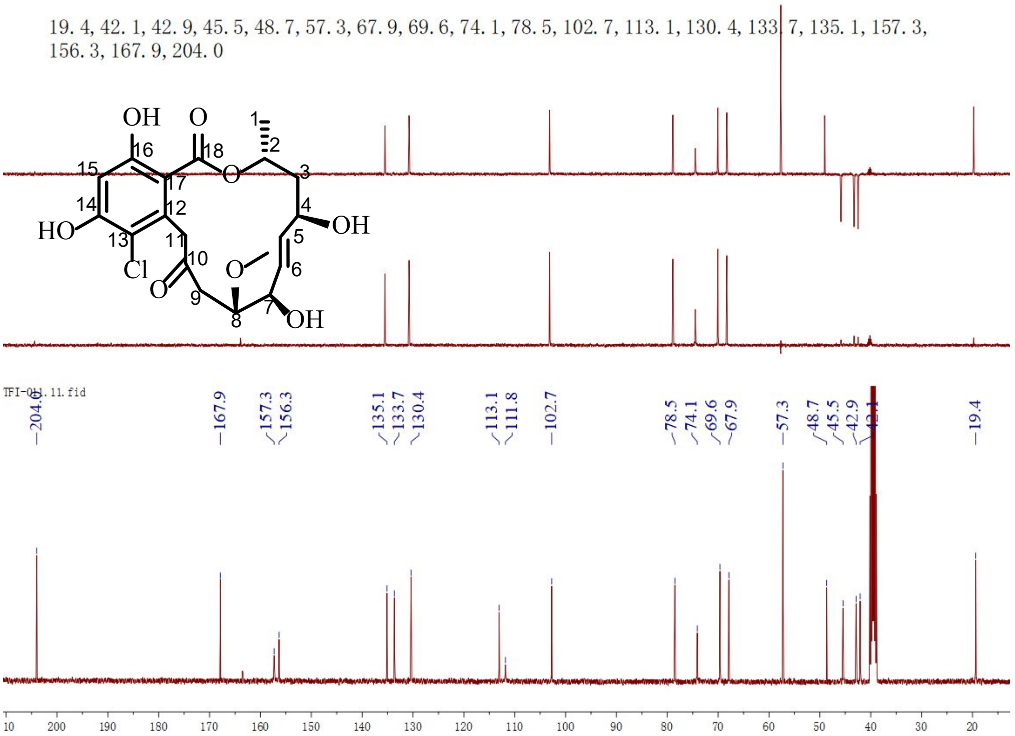

Figure S52 HSQC spectrum (400 MHz, DMSO-d_6) of ilyolactone D (**4**)

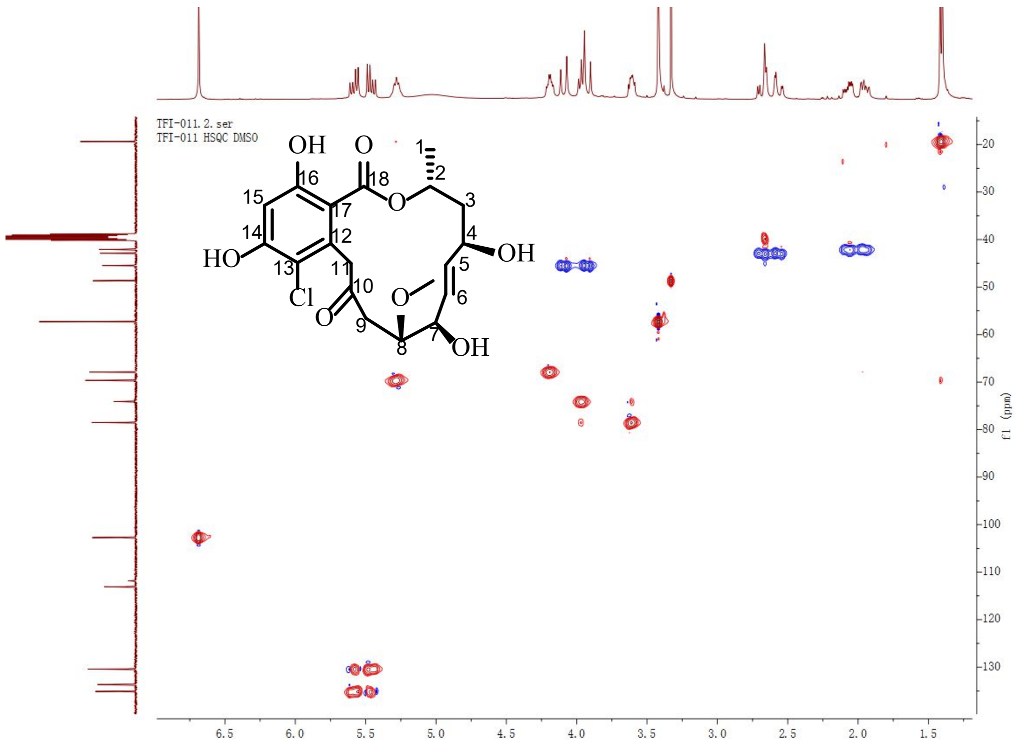

Figure S53 ^{1}H-^{1}H COSY spectrum (400 MHz, DMSO-d_6) of ilyolactone D (**4**)

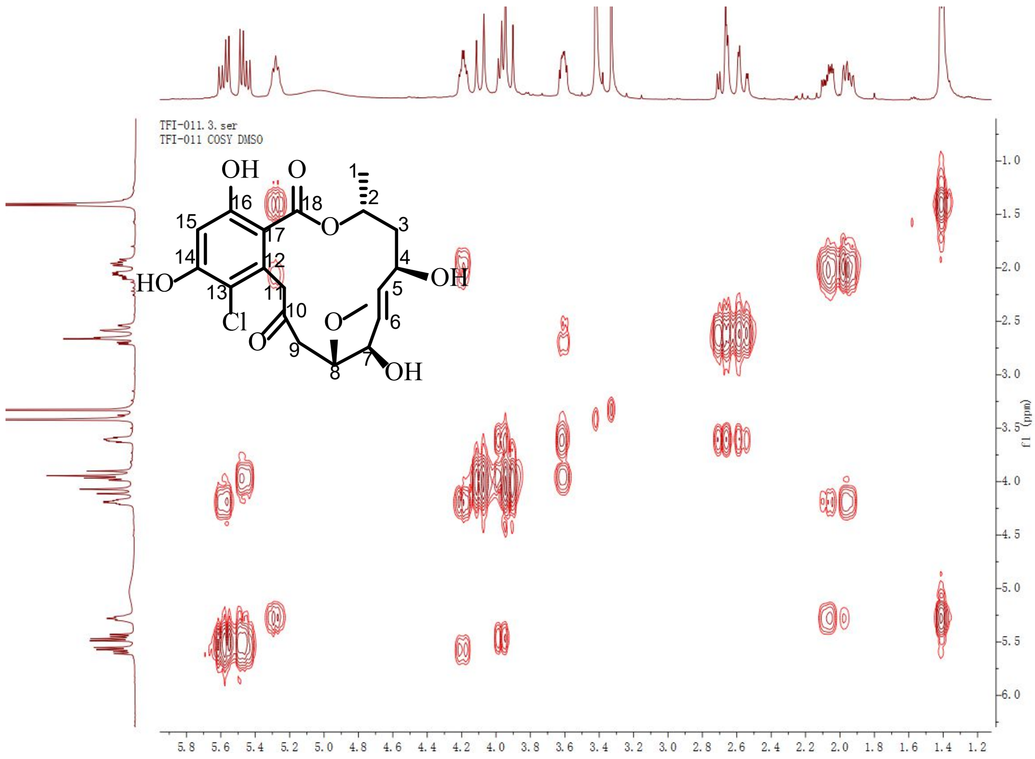

Figure S54 HMBC spectrum (400 MHz, DMSO-d_6) of ilyolactone D (**4**)

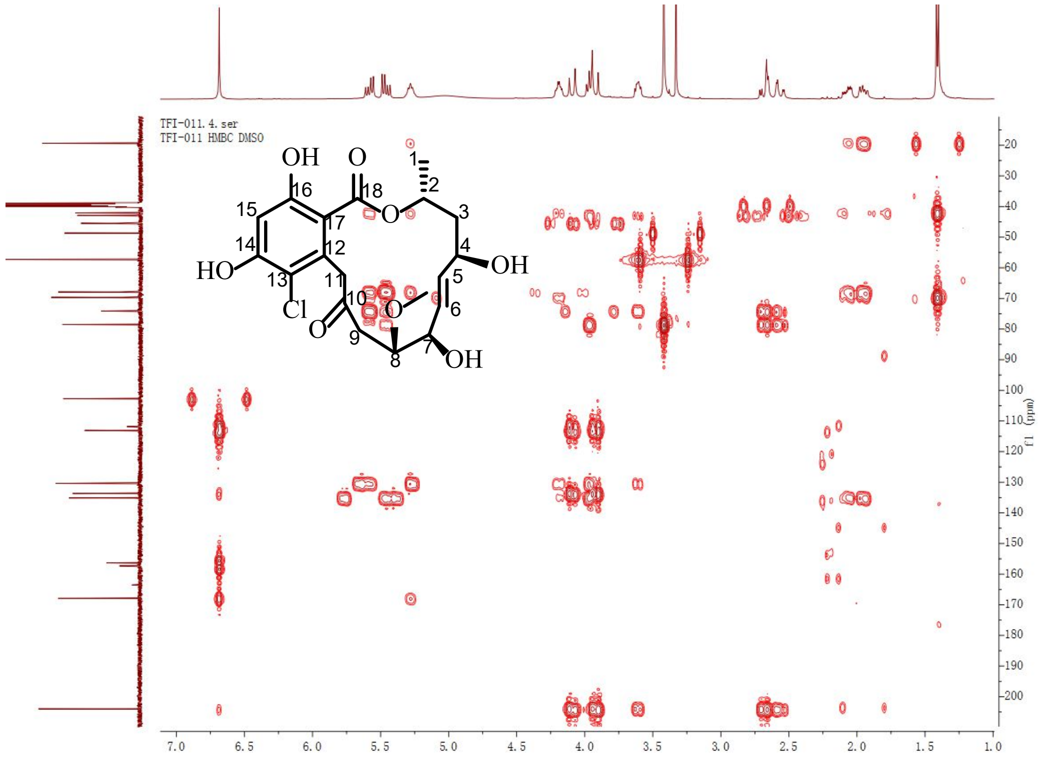

Figure S55 ROESY spectrum (400 MHz, DMSO-d_6) of ilyolactone D (**4**)

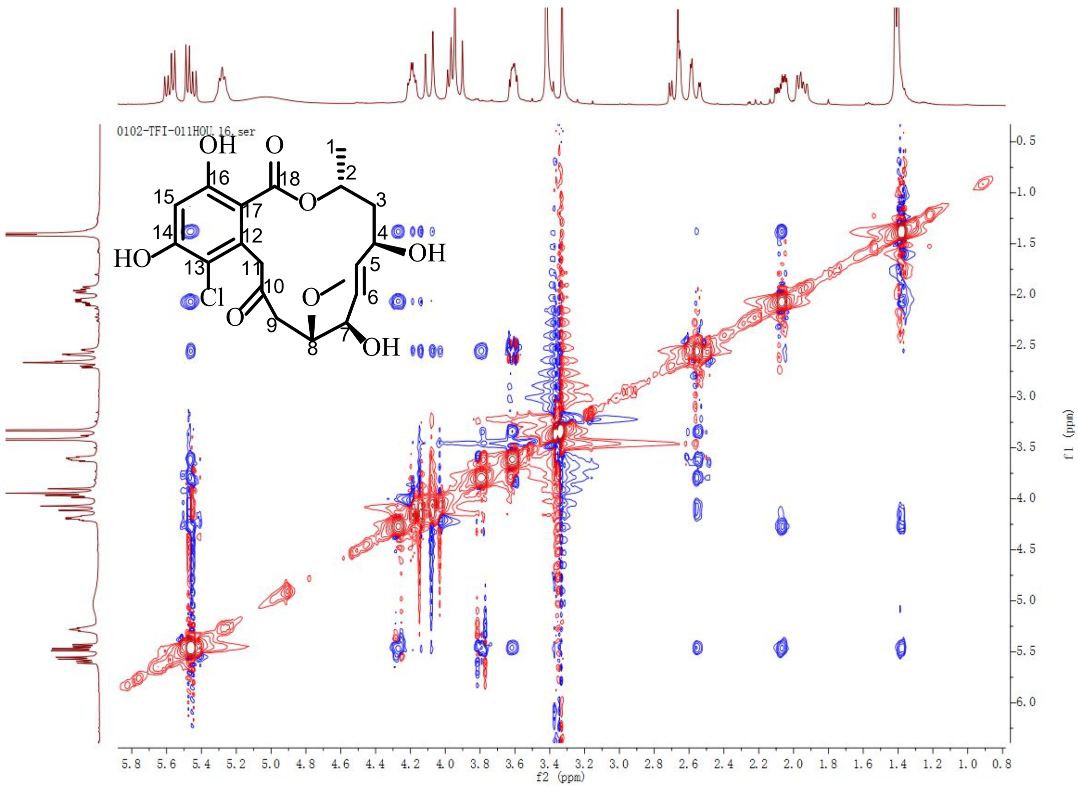

Figure S56 ESIMS spectrum of ilyolactone D (**4**)

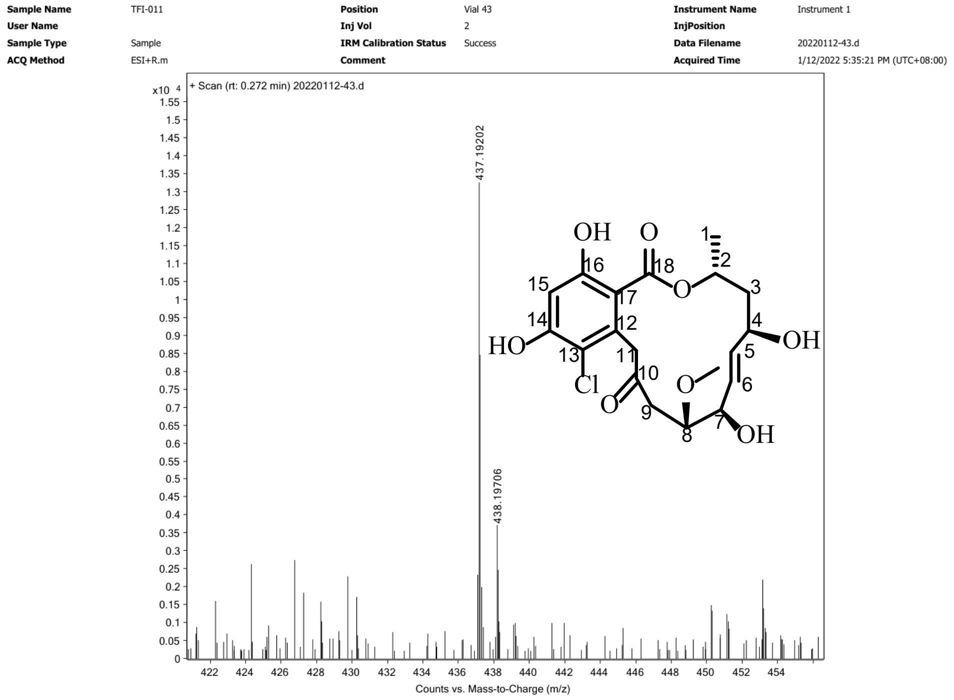

Figure S57 IR spectrum of ilyolactone D (**4**)

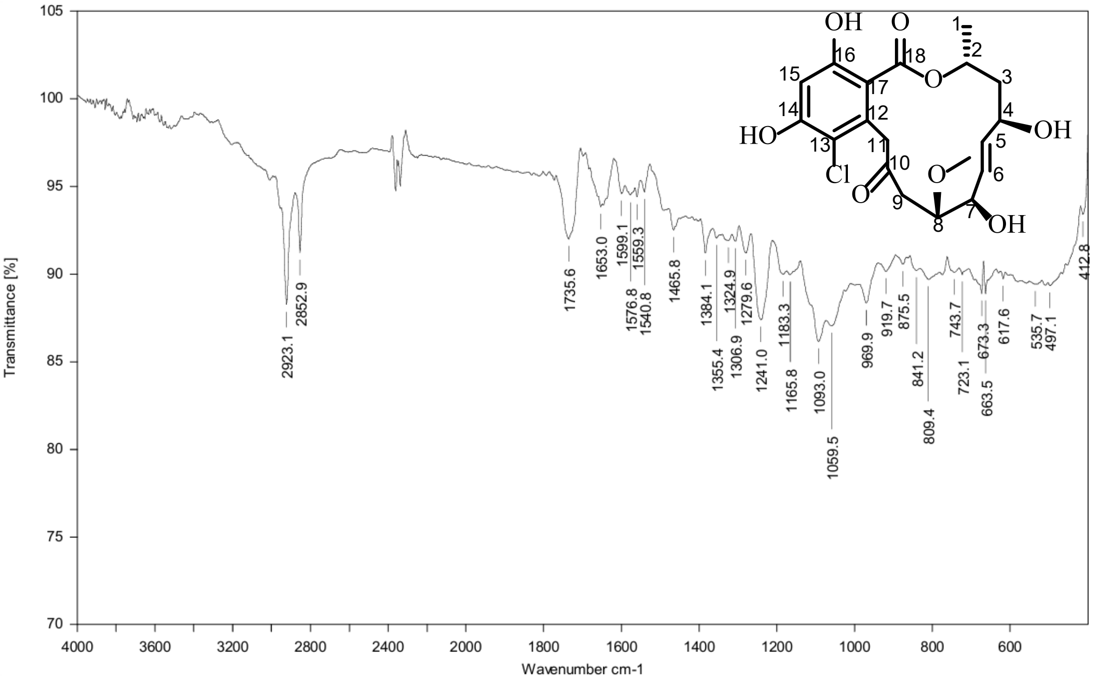

Sample Name: TFI-011

Experiment: MIR_TR.xpm

Operator Name: CPU

Instrument Type: Bruker Tensor 27

Path of File: E:\OPUS\MEAS

Date of Measurement: 13/01/2022

Laboratory: China Pharmaceutical University

Figure S58 HRESIMS spectrum of ilyolactone D (**4**)

Qualitative Compound Identification Report

**Data File**	20220112-43.d	**Sample Name**	TFI-011
**Sample Type**	Sample	**Position**	Vial 43
**Instrument Name**	Instrument 1	**User Name**	
**Acq Method**	ESI+R.m	**Acquired Time**	1/12/2022 5:35:21 PM (UTC+08:00)
**IRM Calibration Status**	Success	**DA Method**	1.m
**Comment**			

**Sample Group**		**Info.**	
**Stream Name**	LC 1	**Acquisition Time (Local)**	1/12/2022 5:35:21 PM (UTC+08:00)
**Acquisition SW Version**	6200 series TOF/6500 series Q-TOF B.06.01 (B6157)	**TOF Driver Version**	6.00.01
**TOF Firmware Version**	17.643		

MS Spectrum

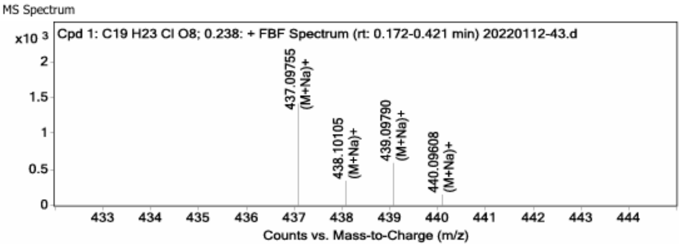

MS Spectrum Peak List

m/z	z	Abund	Ion
437.09755	1	1411.47	(M+Na)+
438.10105	1	334.91	(M+Na)+
439.0979	1	583.99	(M+Na)+
440.09608	1	144.92	(M+Na)+

MS Spectrum

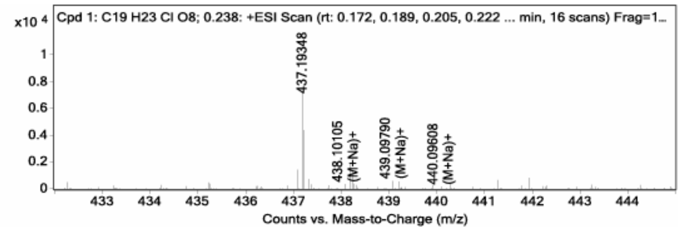

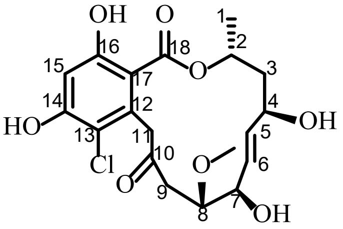

MS Spectrum Peak List

m/z	Calc m/z	Diff(ppm)	z	Abund	Ion
437.09755	437.09737	-0.42	1	1411.47	(M+Na)+
437.19348				7302.65	
438.10105	438.10077	-0.65	1	334.91	(M+Na)+
439.0979	439.09533	-5.87	1	583.99	(M+Na)+
440.09608	440.09835	5.14	1	144.92	(M+Na)+

Qualitative Compound Identification Report

--- End Of Report ---

Figure S59 UV spectrum of ilyolactone D (**4**)

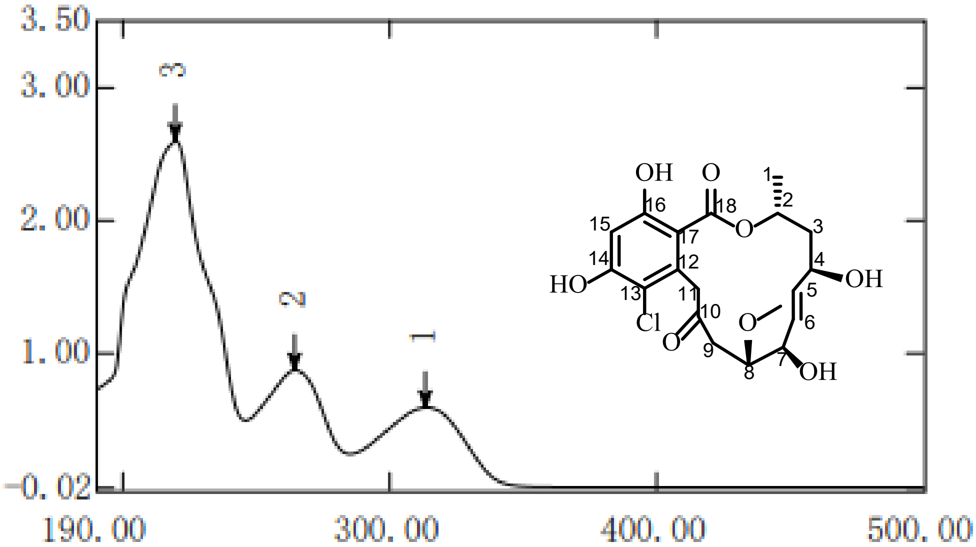

Figure S60 Optical rotation data of ilyolactone D (**4**)

Optical rotation test report

**Sample number**	TFI-011	**weight**	
**Inspection department**	School of Traditional Chinese Pharmacy		
**Inspection item**	Optical rotation		
**Date received**	2022-01-11	**Report data**	2022-01-14

According to the Chinese Pharmacopoeia (2020) of the fourth general rule 0621 spectrophotometric method:

Instrument: AUTOPOL-IV

Light source: halogen tungsten lamp

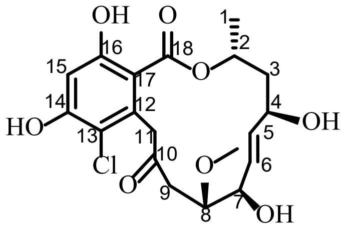

Wave length: 589 nm

Solvent: Methanol

Temperature: 20°C

Result: $[\alpha]_D^{20}$-12.5 (c=0.04)

Notes: 1. Inspection report is only responsible for incoming samples

2. Additions, deletions and corrections to this report are invalid

Figure S61 ^{1}H NMR spectrum (400 MHz, DMSO-d_6) of ilyolactone E (**5**)

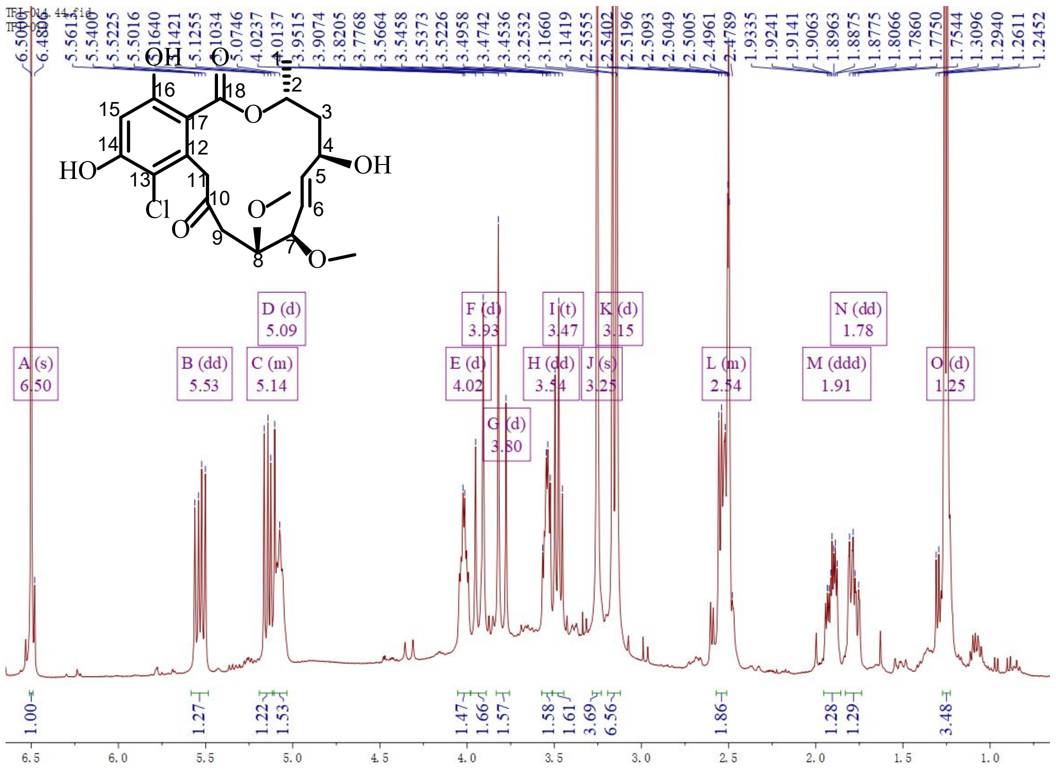

Figure S62 ^{13}C NMR spectrum (400 MHz, DMSO-d_6) of ilyolactone E (**5**)

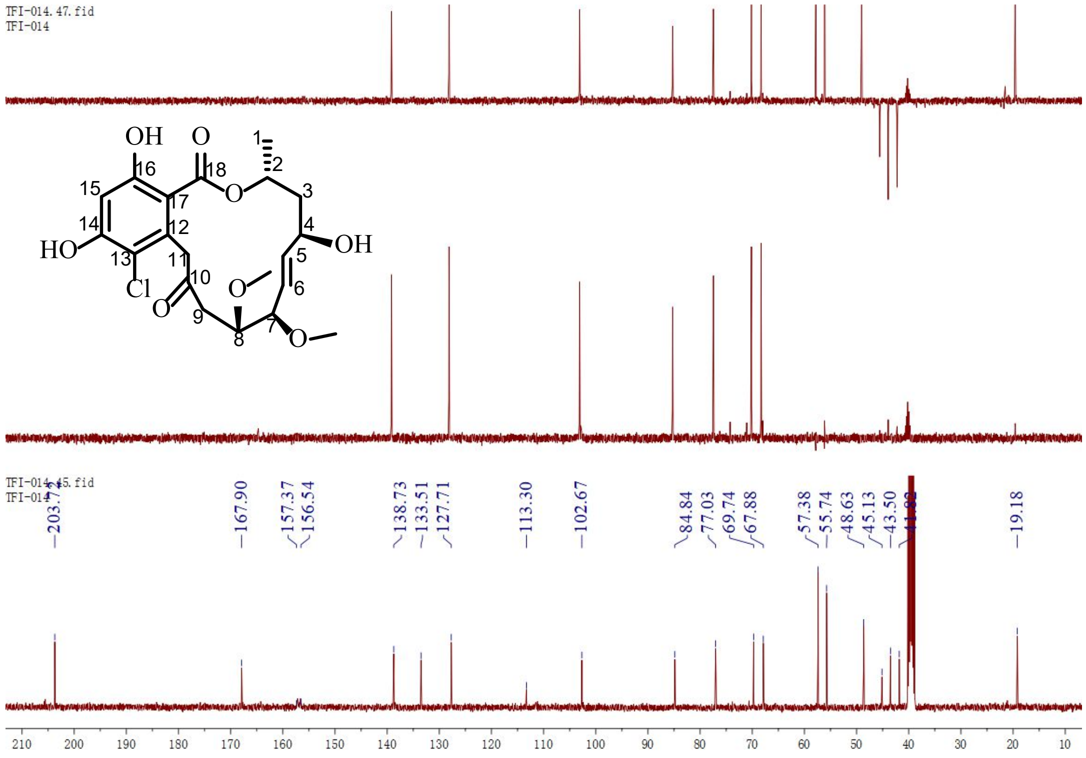

Figure S63 HSQC spectrum (400 MHz, DMSO-d_6) of ilyolactone E (**5**)

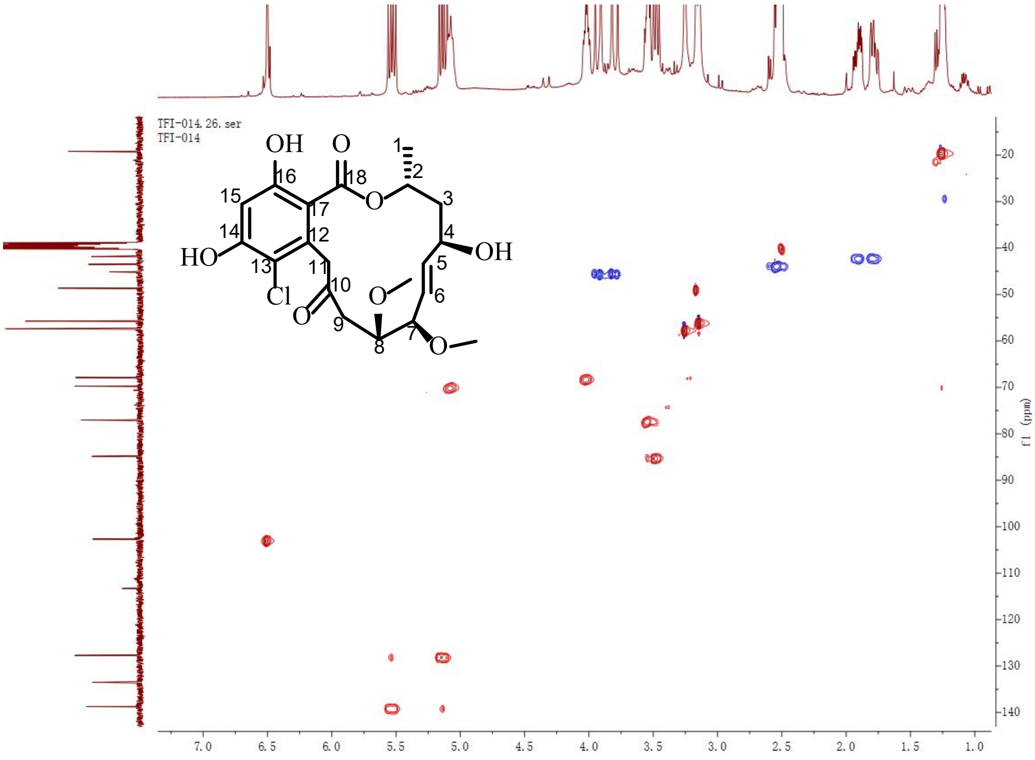

Figure S64 ^{1}H-^{1}H COSY spectrum (400 MHz, DMSO-d_6) of ilyolactone E (**5**)

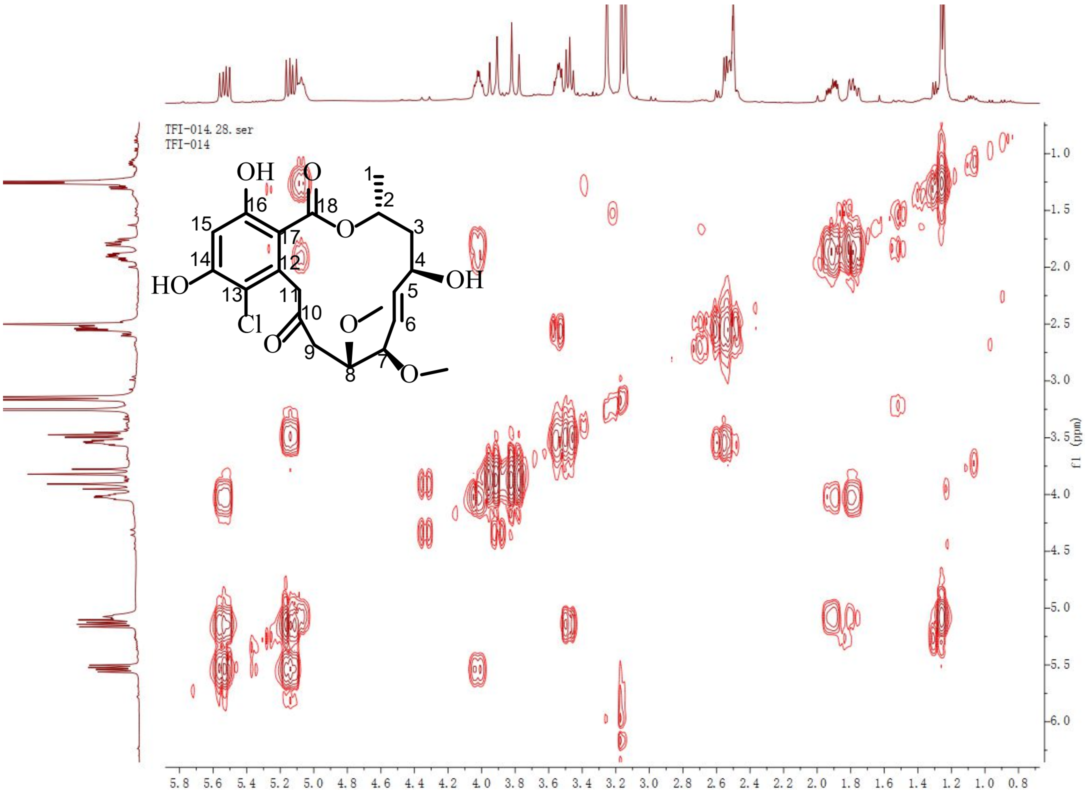

Figure S65 HMBC spectrum (400 MHz, DMSO-d_6) of ilyolactone E (**5**)

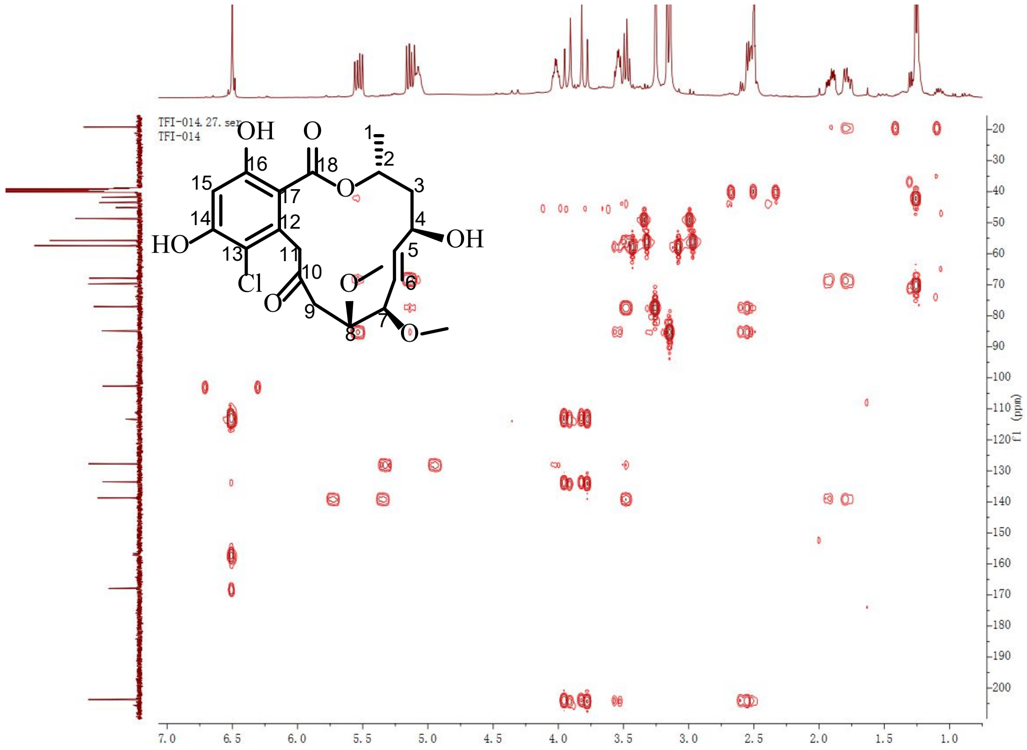

Figure S66 ROESY spectrum (400 MHz, DMSO-d_6) of ilyolactone E (**5**)

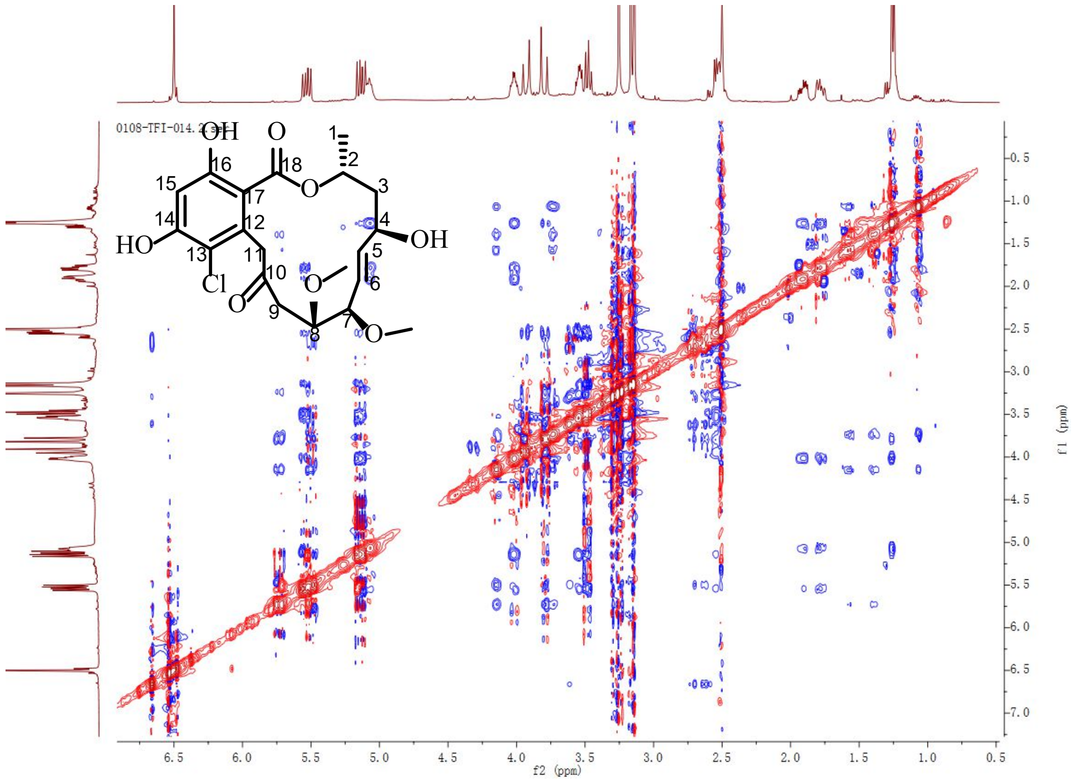

Figure S67 ESIMS spectrum of ilyolactone E (**5**)

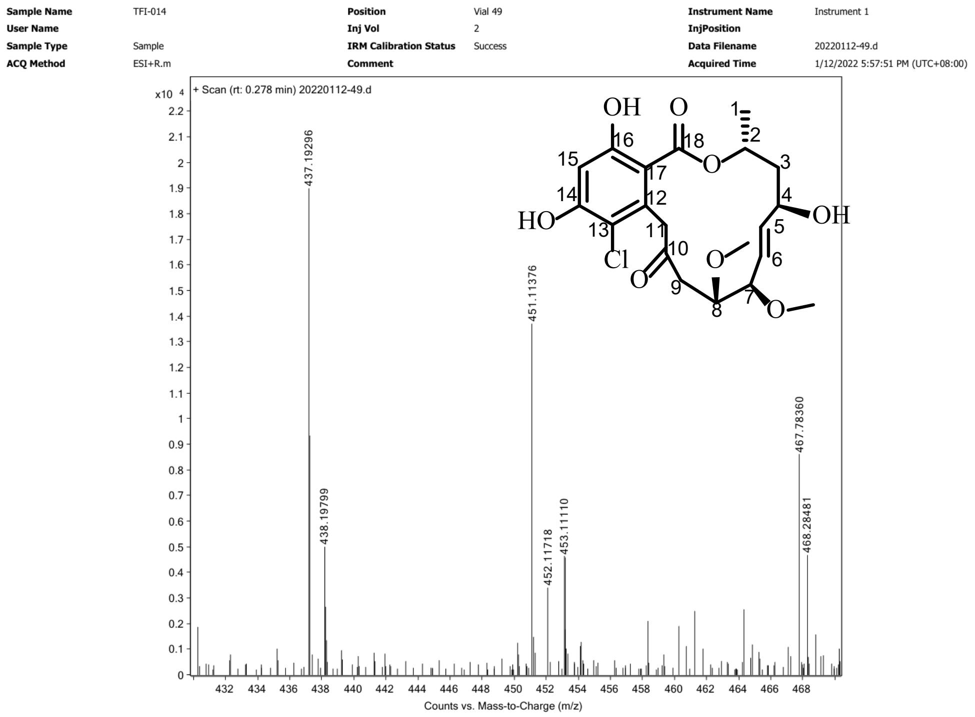

Figure S68 IR spectrum of ilyolactone E (**5**)

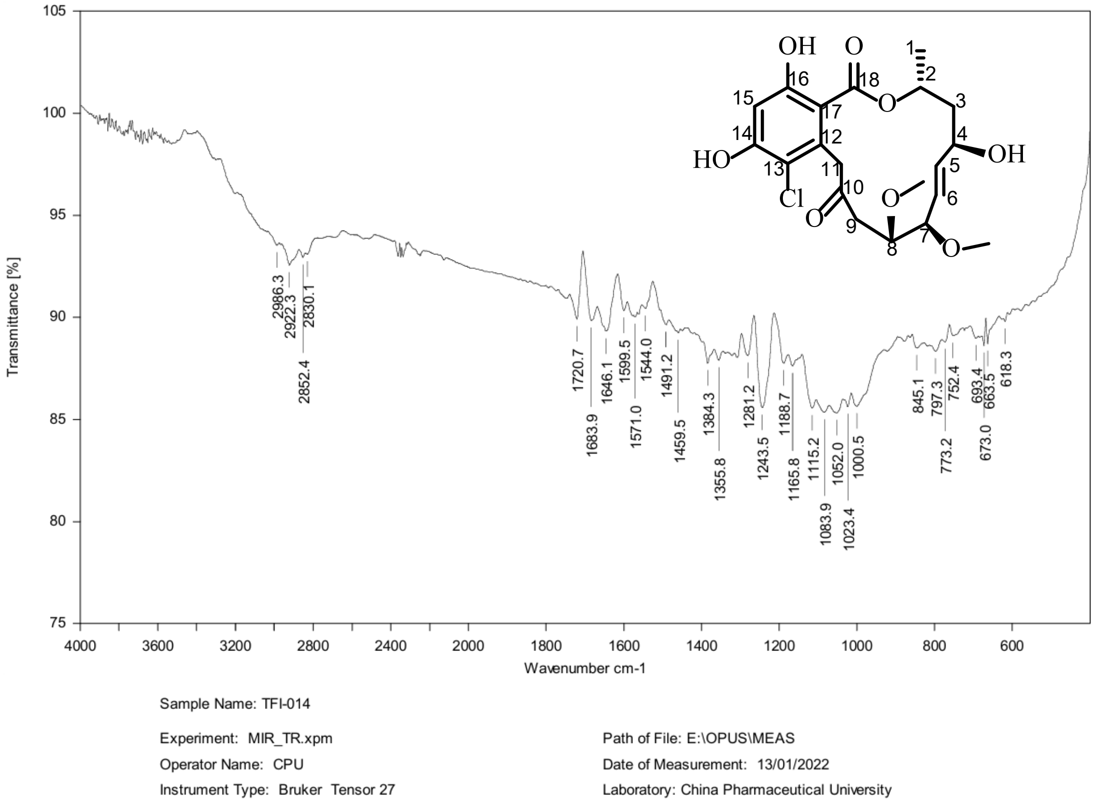

Figure S69 HRESIMS spectrum of ilyolactone E (**5**)

Qualitative Analysis Report

**Data Filename**	20220112-49.d	**Sample Name**	TFI-014
**Sample Type**	Sample	**Position**	Vial 49
**Instrument Name**	Instrument 1	**User Name**	
**Acq Method**	ESI+R.m	**Acquired Time**	1/12/2022 5:57:51 PM (UTC+08:00)
**IRM Calibration Status**	Success	**DA Method**	1.m
**Comment**			

**Sample Group**		**Info.**	
**Stream Name**	LC 1	**Acquisition Time (Local)**	1/12/2022 5:57:51 PM (UTC+08:00)
**Acquisition SW Version**	6200 series TOF/6500 series Q-TOF B.06.01 (B6157)	**TOF Driver Version**	6.00.01
**TOF Firmware Version**	17.643		

Spectra

Collision Energy	Ionization Mode
0	ESI

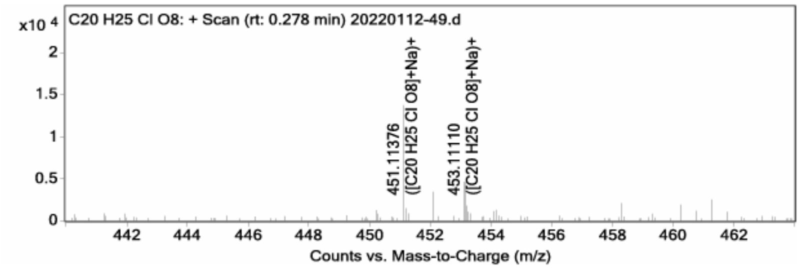

Peak List

m/z	z	Abund
107.04125		72689.42
360.32373	1	290011.13
361.32761	1	58802.88
418.78414	2	77706.76
701.49326	1	47366.57

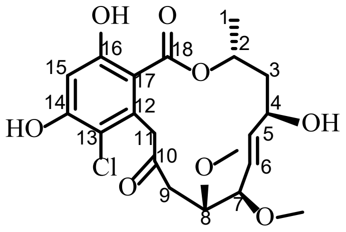

Formula Calculator Element Limits

Element	Min	Max
C	10	30
H	10	30
O	7	10
Cl	0	1

Formula Calculator Results

Formula	Best	Mass	Tgt Mass	Diff (ppm)	Ion Species	CalculatedMz
C20 H25 Cl O8	TRUE	428.12444	428.1238	-1.51	C20 H25 Cl Na O8	451.11302

--- End Of Report ---

Agilent Technologies   Page 1 of 1   Printed at: 5:27 PM on: 1/13/2022

Figure S70 UV spectrum of ilyolactone E **(5)**

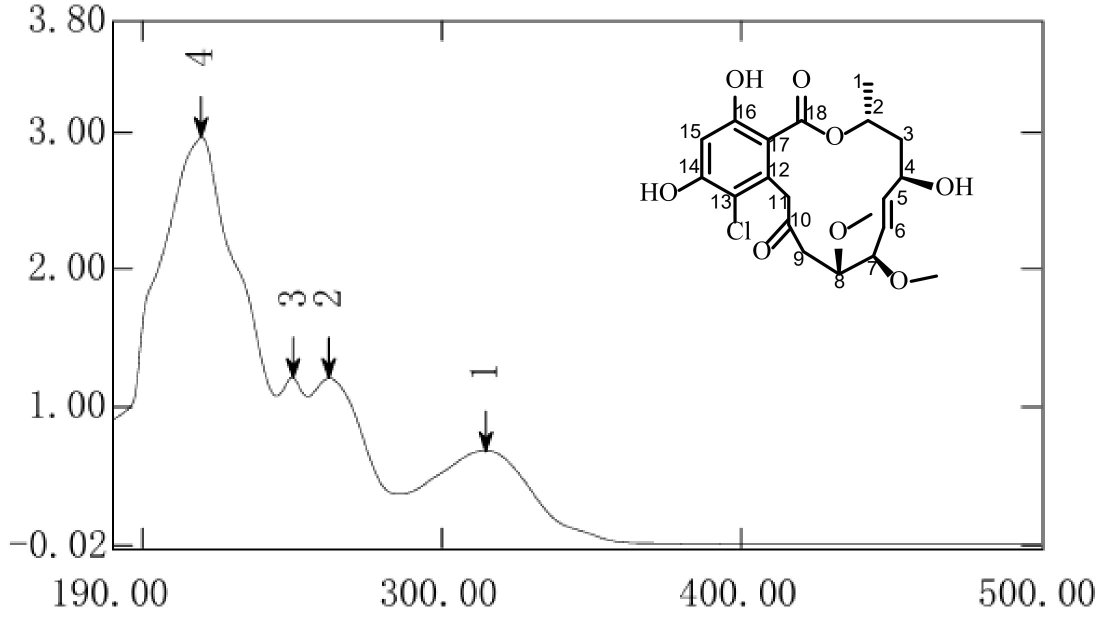

Figure S71 Optical rotation data of ilyolactone E **(5)**

Optical rotation test report

**Sample number**	TFI-014	**weight**	
**Inspection department**	School of Traditional Chinese Pharmacy		
**Inspection item**	Optical rotation		
**Date received**	2022-01-11	**Report data**	2022-01-14

According to the Chinese Pharmacopoeia (2020) of the fourth general rule 0621 spectrophotometric method:

Instrument: AUTOPOL-IV

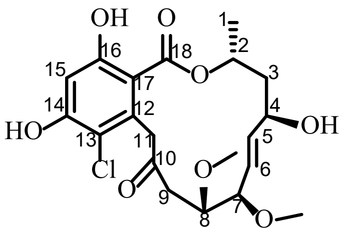

Light source: halogen tungsten lamp

Wave length: 589 nm

Solvent: Methanol

Temperature: 20°C

Result: $[\alpha]_D^{20}$-17.5 (c=0.04)

Notes: 1. Inspection report is only responsible for incoming samples

2. Additions, deletions and corrections to this report are invalid

Figure S72 ^{1}H NMR spectrum (400 MHz, DMSO-d_6) of ilyolactone F (**6**)

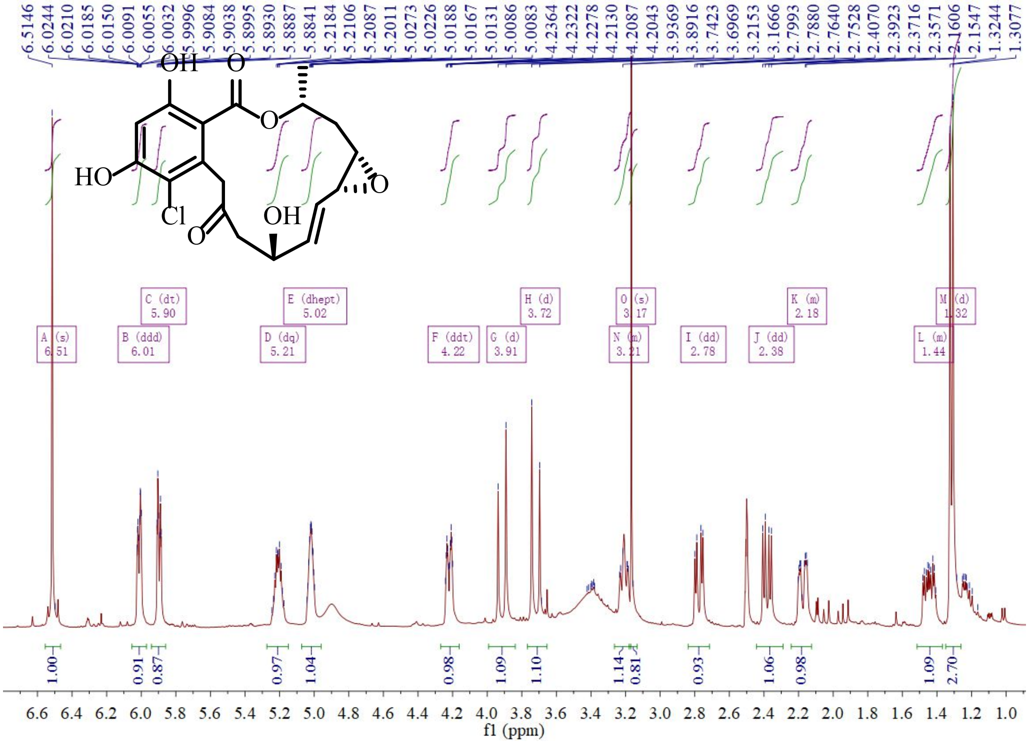

Figure S73 ^{13}C NMR spectrum (400 MHz, DMSO-d_6) of ilyolactone F (**6**)

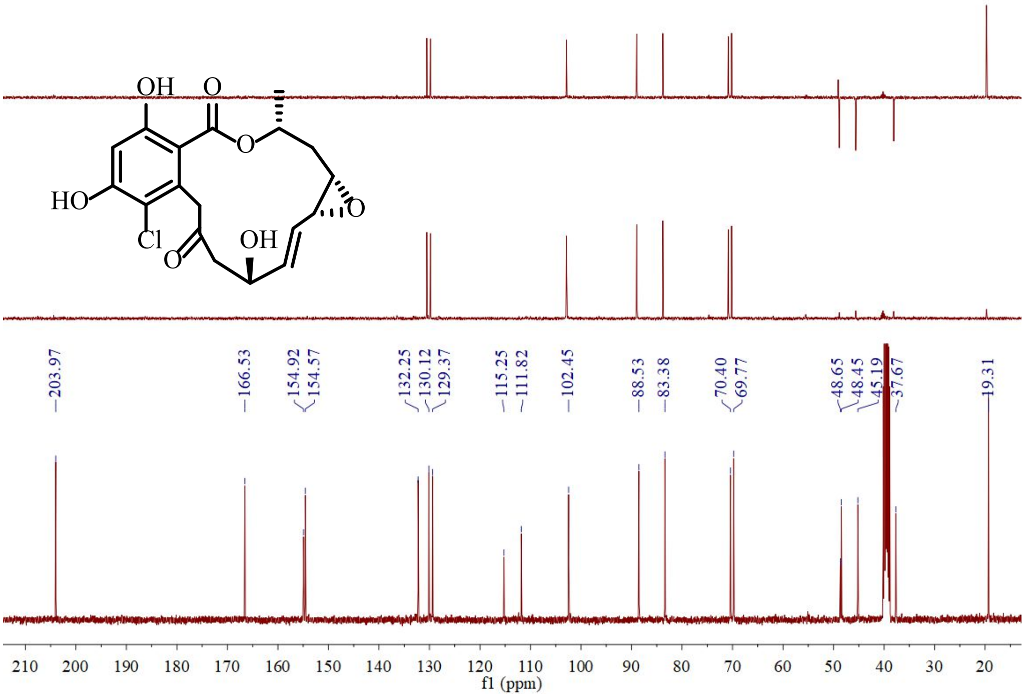

Figure S74 HSQC spectrum (400 MHz, DMSO-d_6) of ilyolactone F (**6**)

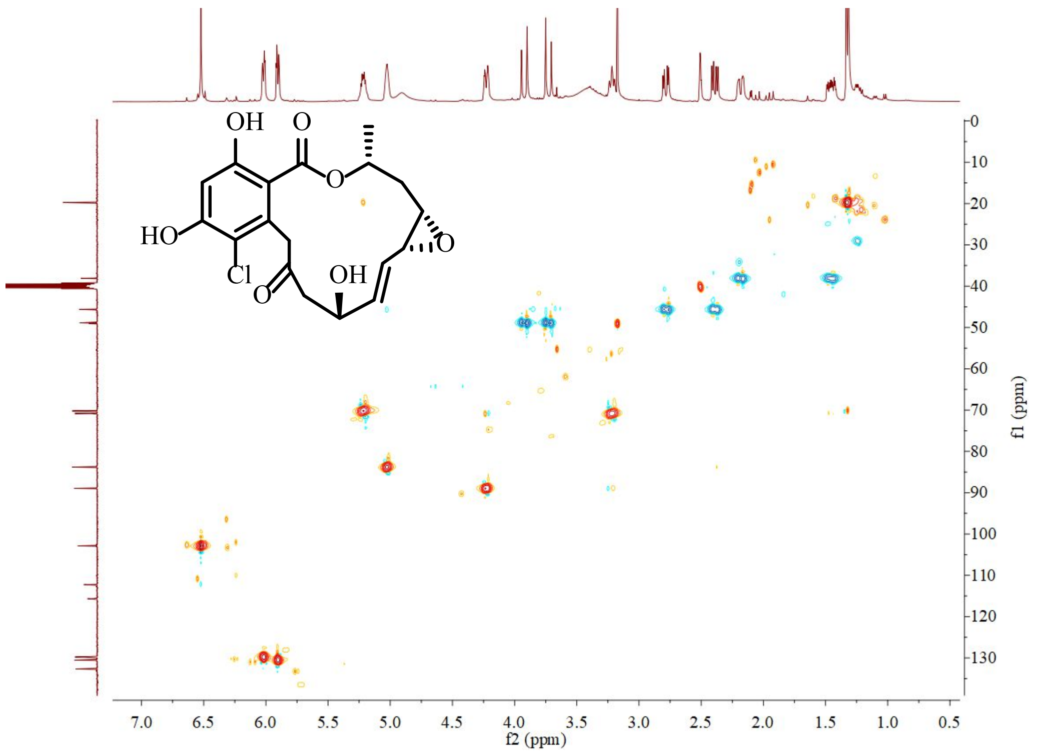

Figure S75 ^{1}H-^{1}H COSY spectrum (400 MHz, DMSO-d_6) of ilyolactone F (**6**)

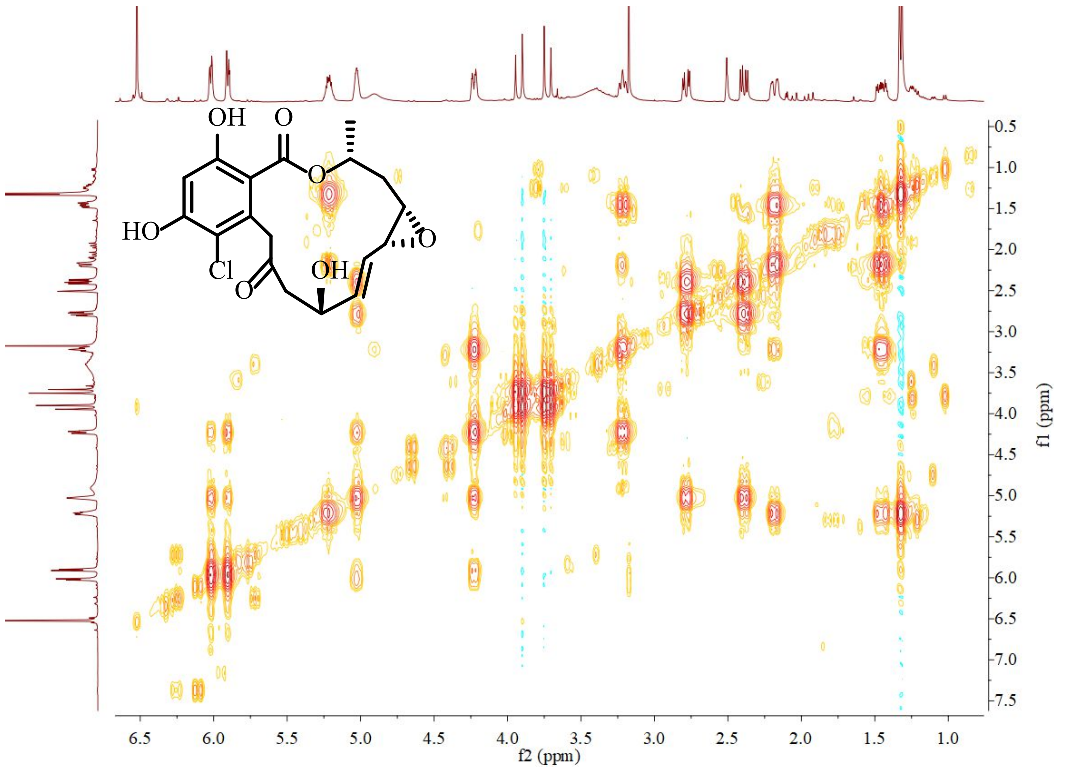

Figure S76 HMBC spectrum (400 MHz, DMSO-d_6) of ilyolactone F (**6**)

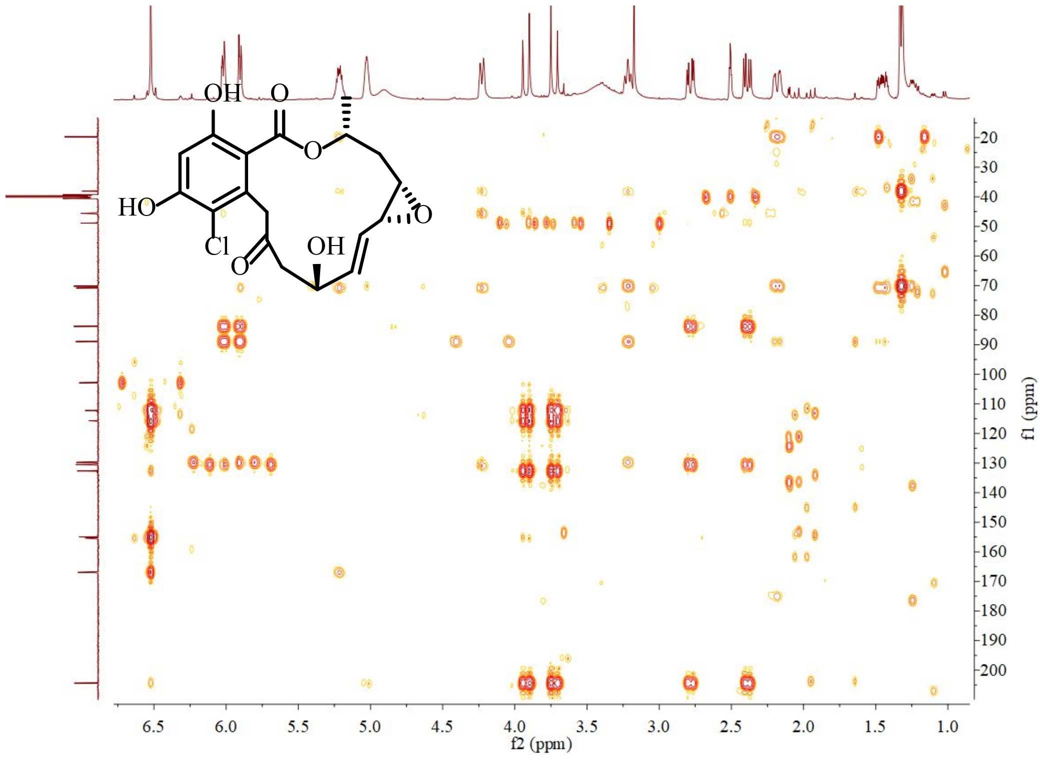

Figure S77 ROESY spectrum (400 MHz, DMSO-d_6) of ilyolactone F (**6**)

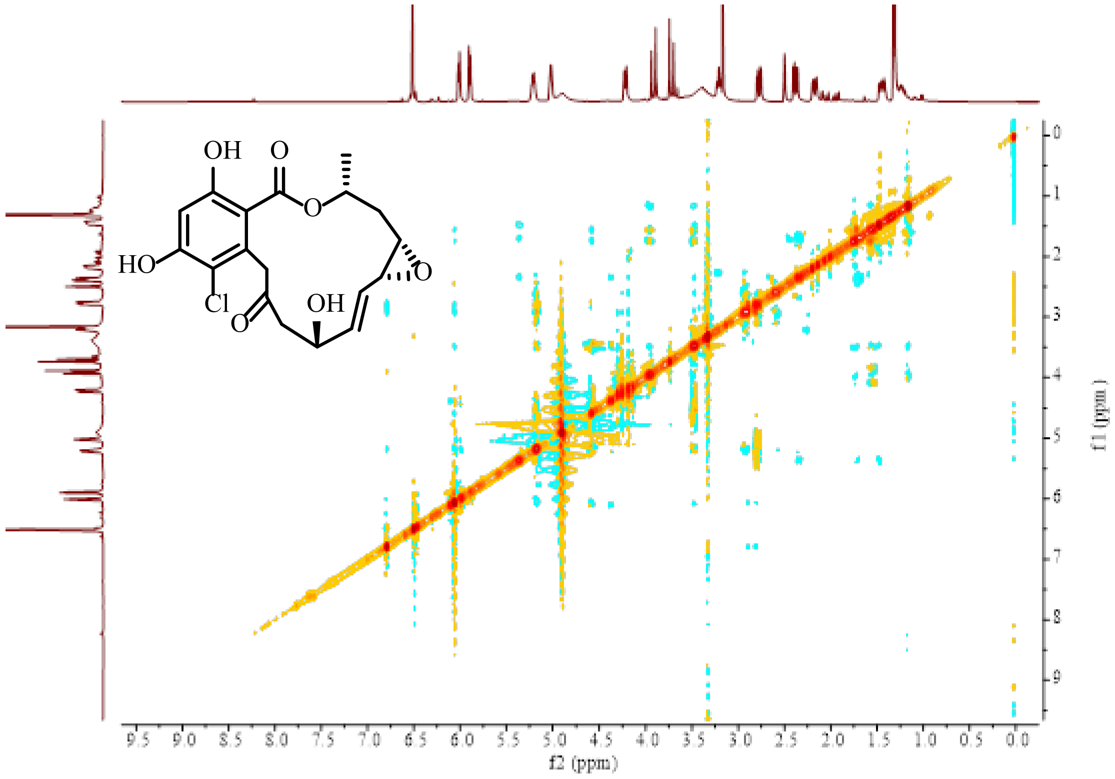

Figure S78 ESIMS spectrum of ilyolactone F (**6**)

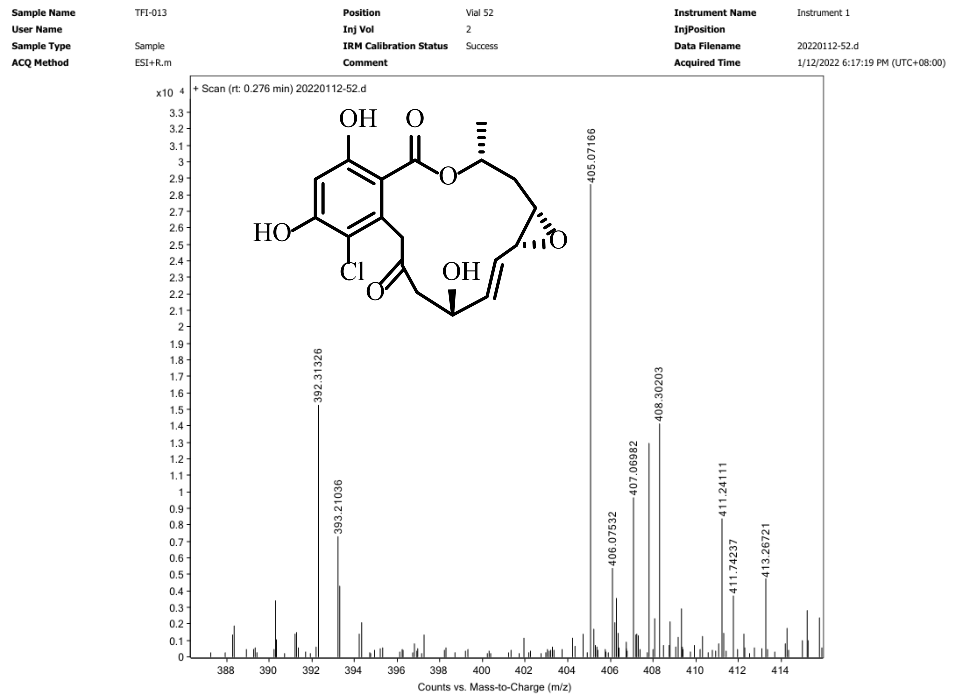

Figure S79 IR spectrum of ilyolactone F (**6**)

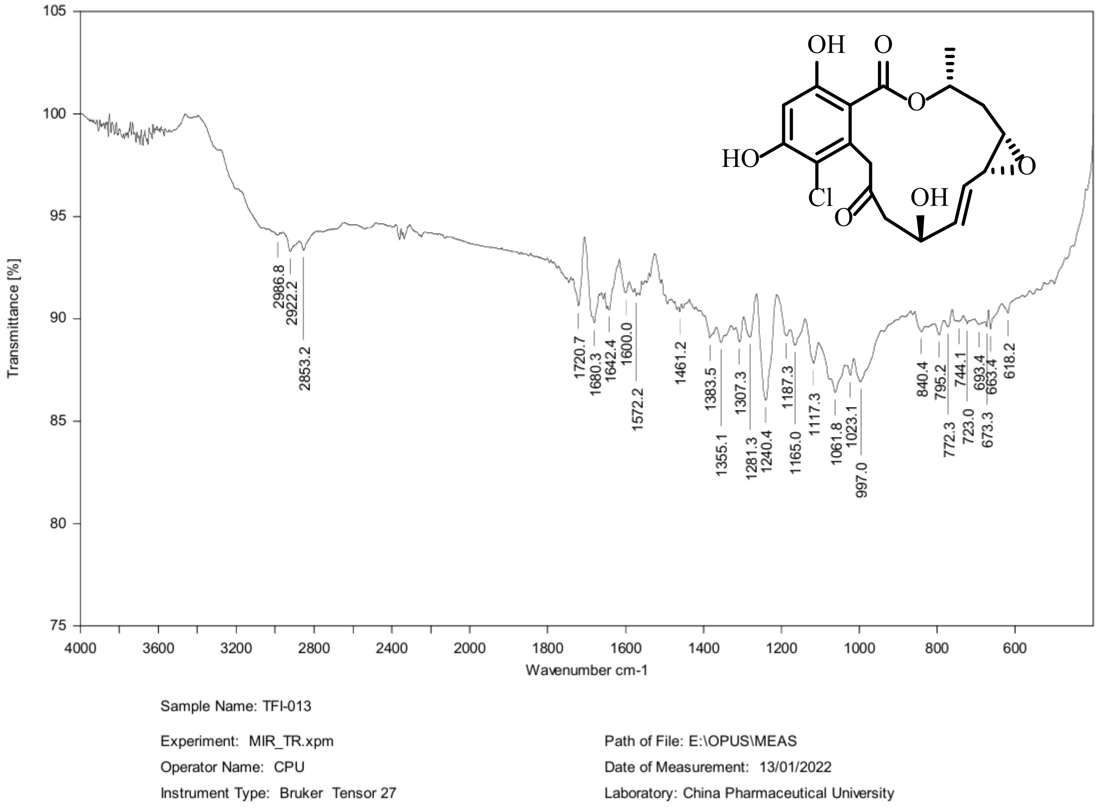

Figure S80 HRESIMS spectrum of ilyolactone F (**6**)

Qualitative Analysis Report

**Data Filename**	20220112-52.d	**Sample Name**	TFI-013
**Sample Type**	Sample	**Position**	Vial 52
**Instrument Name**	Instrument 1	**User Name**	
**Acq Method**	ESI+R.m	**Acquired Time**	1/12/2022 6:17:19 PM (UTC+08:00)
**IRM Calibration Status**	Success	**DA Method**	1.m
**Comment**			

**Sample Group**		**Info.**	
**Stream Name**	LC 1	**Acquisition Time (Local)**	1/12/2022 6:17:19 PM (UTC+08:00)
**Acquisition SW Version**	6200 series TOF/6500 series Q-TOF B.06.01 (B6157)	**TOF Driver Version**	6.00.01
**TOF Firmware Version**	17.643		

Spectra

Collision Energy	Ionization Mode
0	ESI

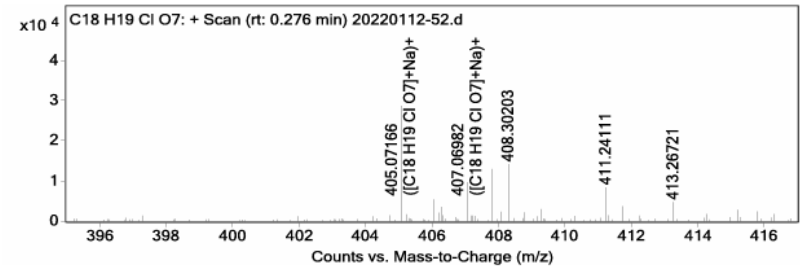

Peak List

m/z	z	Abund
107.041		125026.13
360.32357	1	346931.41
361.32708	1	69423.38
418.7836	2	82519.7
701.4929	1	71342.8

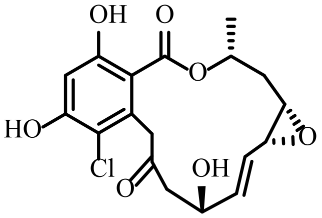

Formula Calculator Element Limits

Element	Min	Max
C	10	30
H	10	30
O	6	10
Cl	0	1

Formula Calculator Results

Formula	Best	Mass	Tgt Mass	Diff (ppm)	Ion Species	CalculatedMz
C18 H19 Cl O7	TRUE	382.08238	382.08193	-1.18	C18 H19 Cl Na O7	405.07115

--- End Of Report ---

Figure S81 UV spectrum of ilyolactone F (**6**)

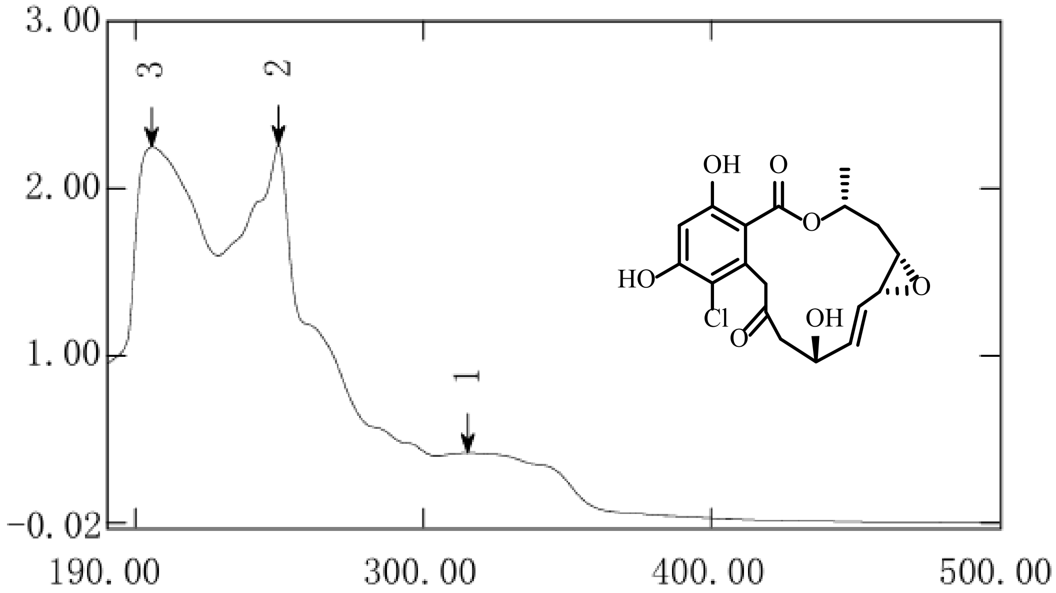

Figure S82 Optical rotation data of ilyolactone F (**6**)

Optical rotation test report

**Sample number**	TFI-013	**weight**	
**Inspection department**	School of Traditional Chinese Pharmacy		
**Inspection item**	Optical rotation		
**Date received**	2022-01-11	**Report data**	2022-01-14

According to the Chinese Pharmacopoeia (2020) of the fourth general rule 0621 spectrophotometric method:

Instrument: AUTOPOL-IV

Light source: halogen tungsten lamp

Wave length: 589 nm

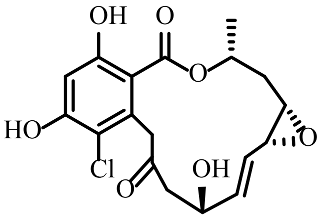

Solvent: Methanol

Temperature: 20°C

Result: $[\alpha]_D^{20}$-22.5 (c=0.04)

Notes: 1. Inspection report is only responsible for incoming samples

2. Additions, deletions and corrections to this report are invalid

Figure S83 ^{1}H NMR spectrum (400 MHz, CD_3OD) of ilyolactone G (**7**)

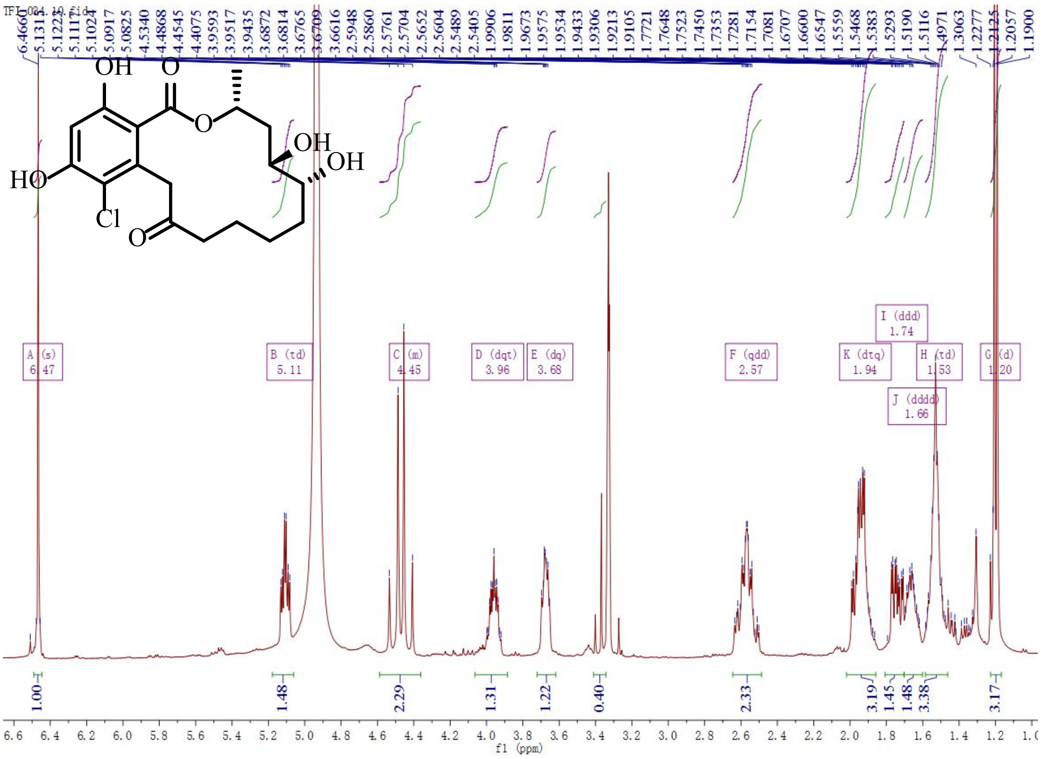

Figure S84 ^{13}C NMR spectrum (400 MHz, CD_3OD) of ilyolactone G (**7**)

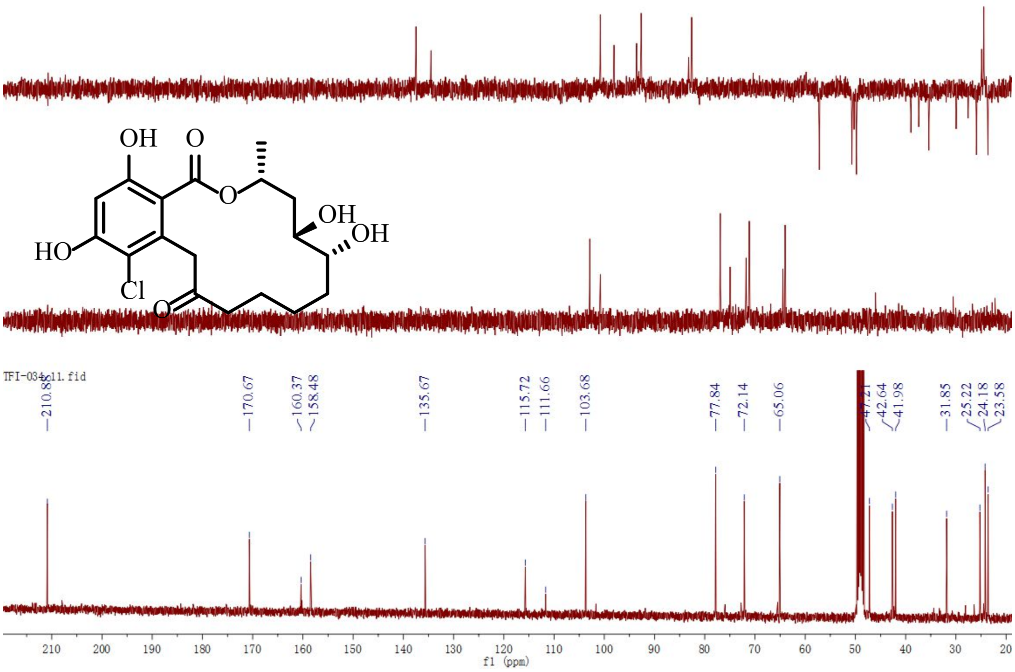

Figure S85 HSQC spectrum (400 MHz, CD_3OD) of ilyolactone G (**7**)

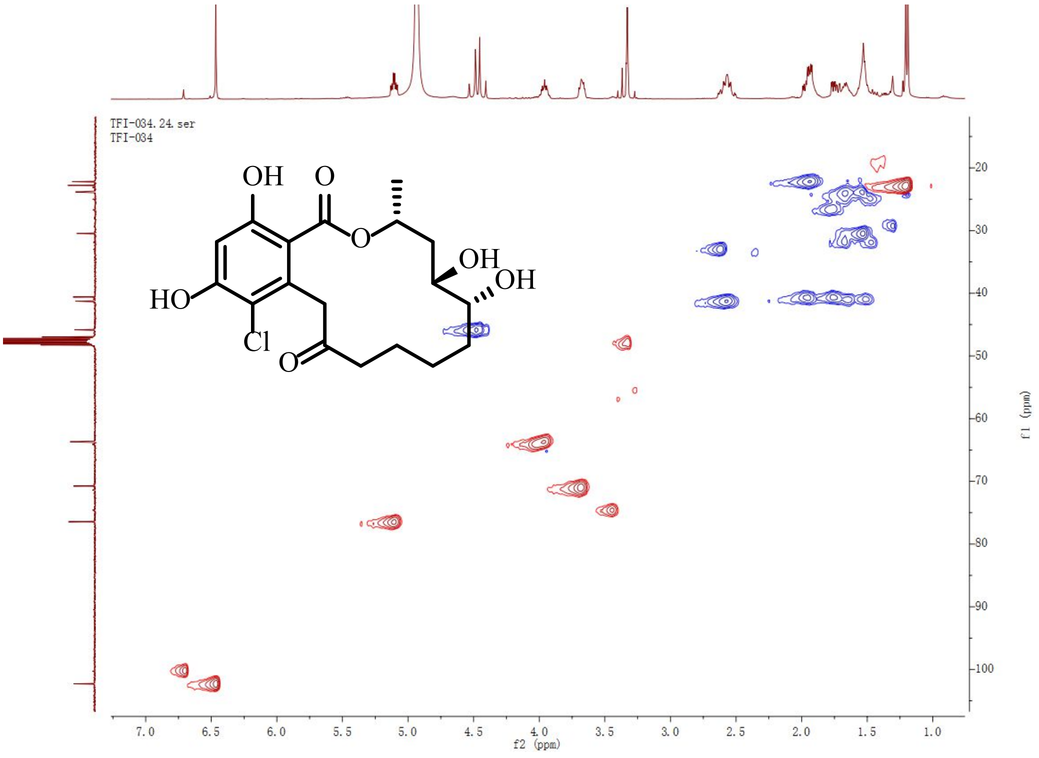

Figure S86 ^{1}H-^{1}H COSY spectrum (400 MHz, CD_3OD) of ilyolactone G (**7**)

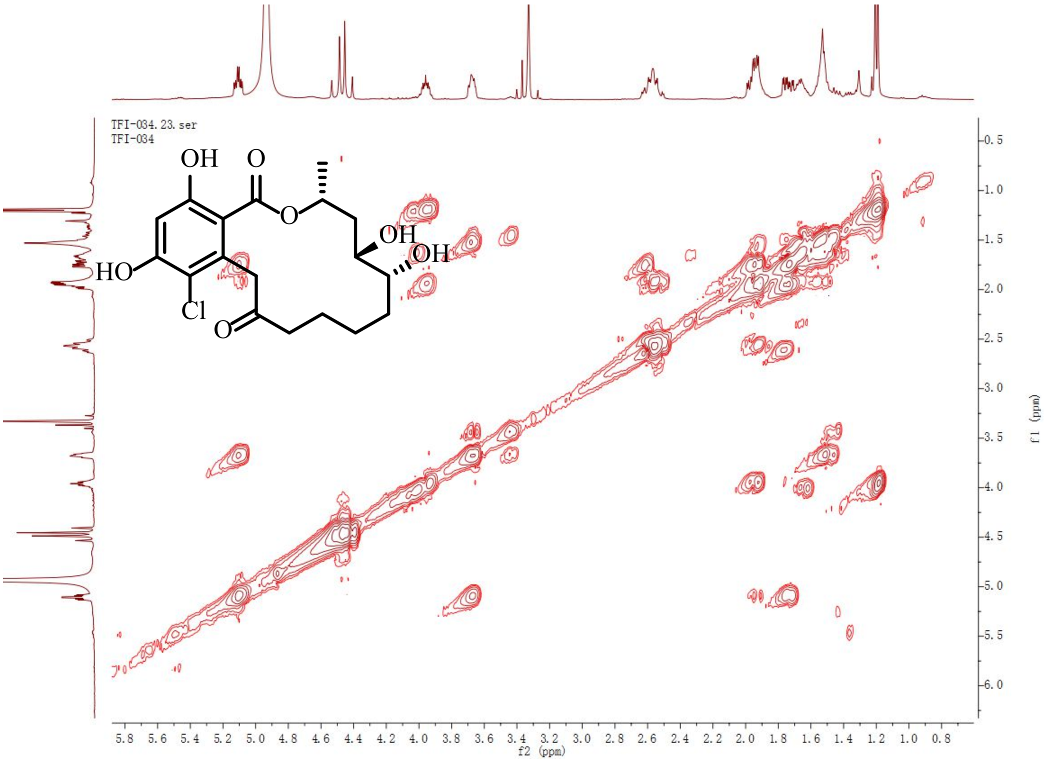

Figure S87 HMBC spectrum (400 MHz, CD_3OD) of ilyolactone G (**7**)

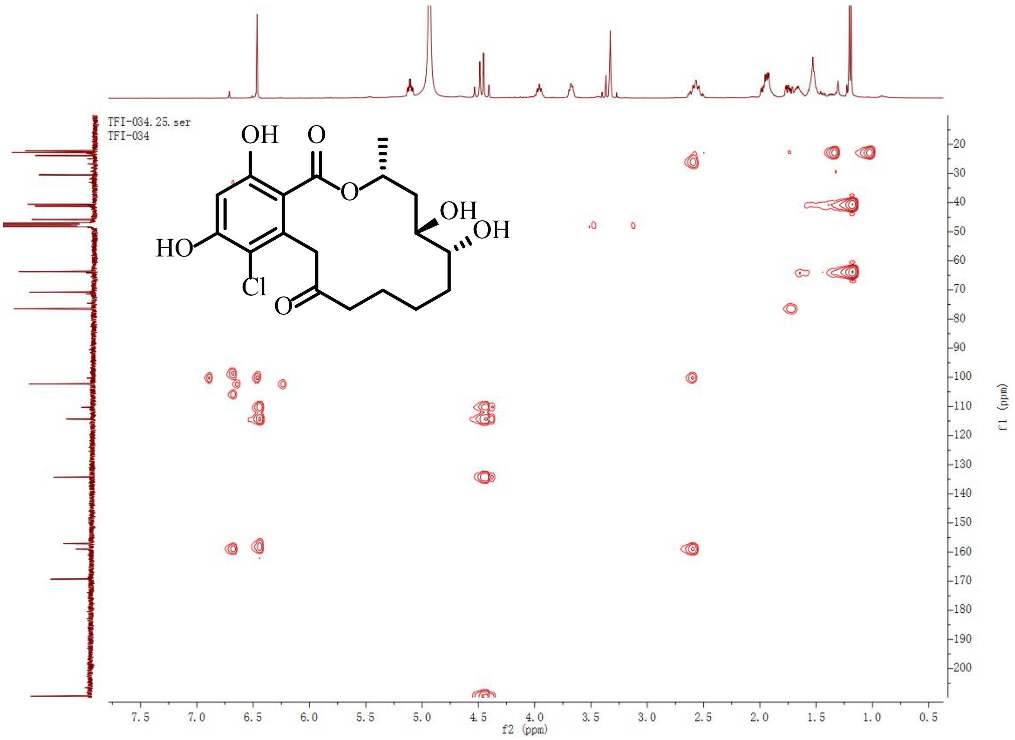

Figure S88 ESIMS spectrum of ilyolactone G (**7**)

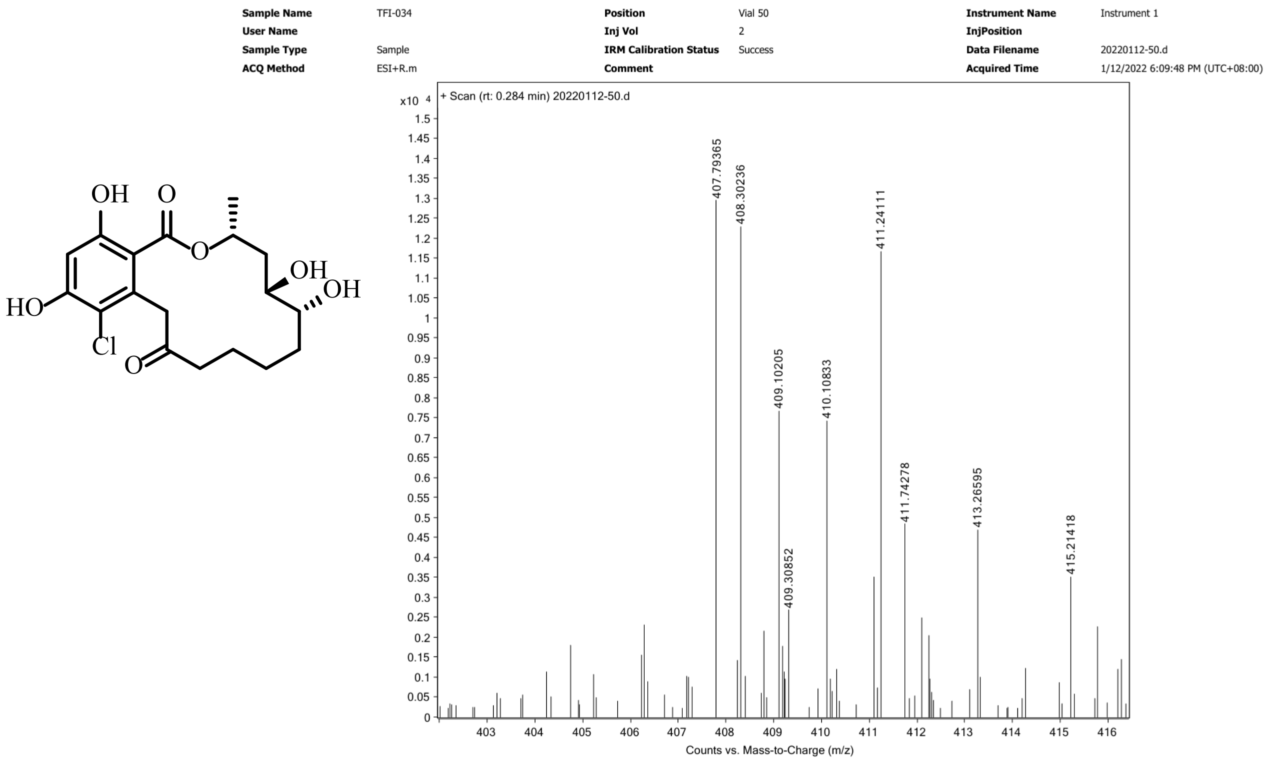

Figure S89 IR spectrum of ilyolactone G (**7**)

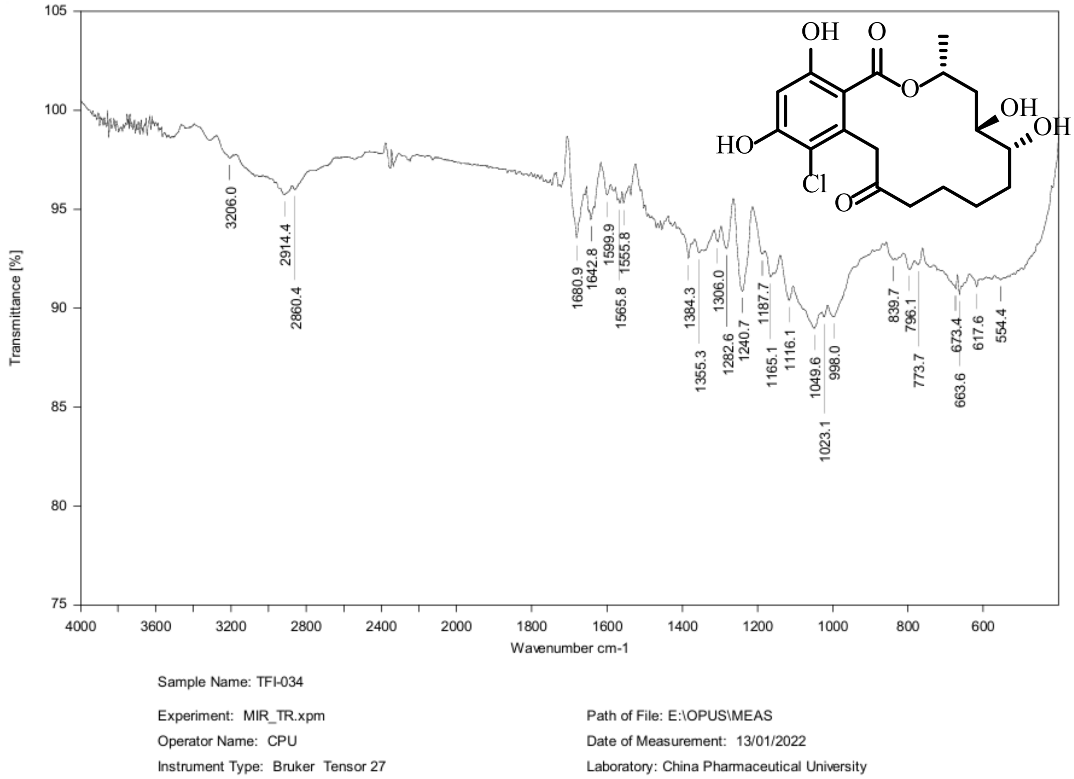

Figure S90 HRESIMS spectrum of ilyolactone G (**7**)

Qualitative Compound Identification Report

**Data File**	20220112-50.d	**Sample Name**	TFI-034
**Sample Type**	Sample	**Position**	Vial 50
**Instrument Name**	Instrument 1	**User Name**	
**Acq Method**	ESI+R.m	**Acquired Time**	1/12/2022 6:09:48 PM (UTC+08:00)
**IRM Calibration Status**	Success	**DA Method**	1.m
**Comment**			

**Sample Group**		**Info.**	
**Stream Name**	LC 1	**Acquisition Time (Local)**	1/12/2022 6:09:48 PM (UTC+08:00)
**Acquisition SW Version**	6200 series TOF/6500 series Q-TOF B.06.01 (B6157)	**TOF Driver Version**	6.00.01
**TOF Firmware Version**	17.643		

Compound Table

Compound Label	RT	Mass	Abund	Formula	Tgt Mass	Diff (ppm)	Hits (DB)
Cpd 1: C18 H23 Cl O7; 0.250	0.25	386.11507	5128	C18 H23 Cl O7	386.11323	4.75	1

Compound Label	*m/z*	RT	Algorithm	Mass
Cpd 1: C18 H23 Cl O7; 0.250	409.10239	0.25	Find By Formula	386.11507

MS Spectrum

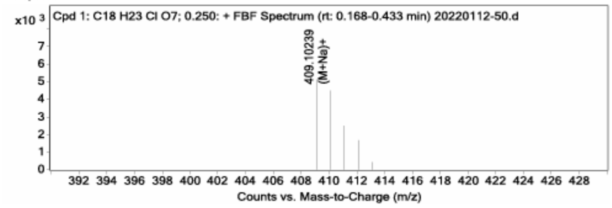

MS Spectrum Peak List

m/z	z	Abund	Ion
409.10239	1	5128.41	(M+Na)+
410.10827	1	4504.81	(M+Na)+
411.10408	1	2491.96	(M+Na)+
412.10627	1	1665.45	(M+Na)+
413.10871	1	419.3	(M+Na)+

MS Spectrum

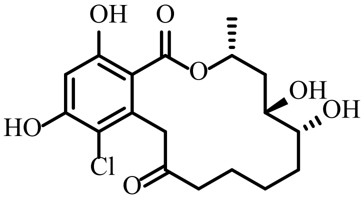

Qualitative Compound Identification Report

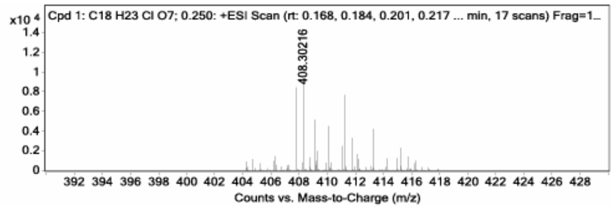

MS Spectrum Peak List

m/z	*Calc m/z*	Diff(ppm)	z	Abund	Ion
409.10239	409.10245	0.15	1	5128.41	(M+Na)+
410.10827	410.10586	-5.88	1	4504.81	(M+Na)+
411.10408	411.10032	-9.15	1	2491.96	(M+Na)+
412.10627	412.10337	-7.02	1	1665.45	(M+Na)+
413.10871	413.1055	-7.76	1	419.3	(M+Na)+

--- End Of Report ---

Figure S91 UV spectrum of ilyolactone G (**7**)

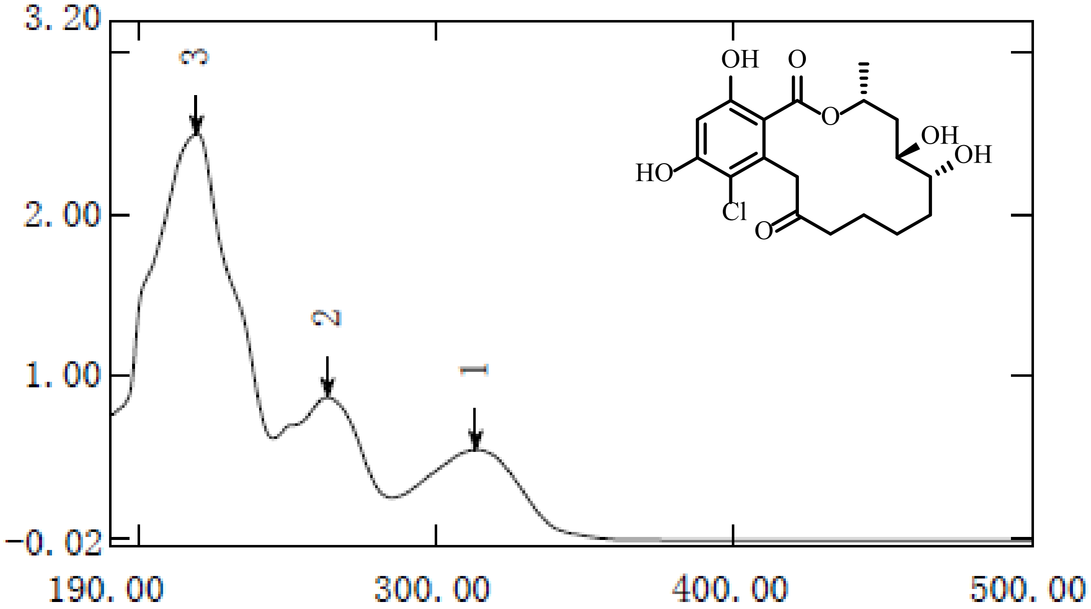

Figure S92 Optical rotation data of ilyolactone G (**7**)

Optical rotation test report

**Sample number**	TFI-034	**weight**	
**Inspection department**	School of Traditional Chinese Pharmacy		
**Inspection item**	Optical rotation		
**Date received**	2022-01-11	**Report data**	2022-01-14

According to the Chinese Pharmacopoeia (2020) of the fourth general rule 0621 spectrophotometric method:

Instrument: AUTOPOL-IV

Light source: halogen tungsten lamp

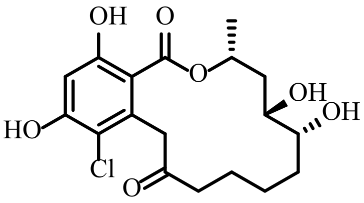

Wave length: 589 nm

Solvent: Methanol

Temperature: 20°C

Result: $[\alpha]_D^{20}$ -20.0 (c=0.04)

Notes: 1. Inspection report is only responsible for incoming samples

2. Additions, deletions and corrections to this report are invalid

Figure S93 ^{1}H NMR spectrum (400 MHz, CD_3OD) of ilyolactone H (**8**)

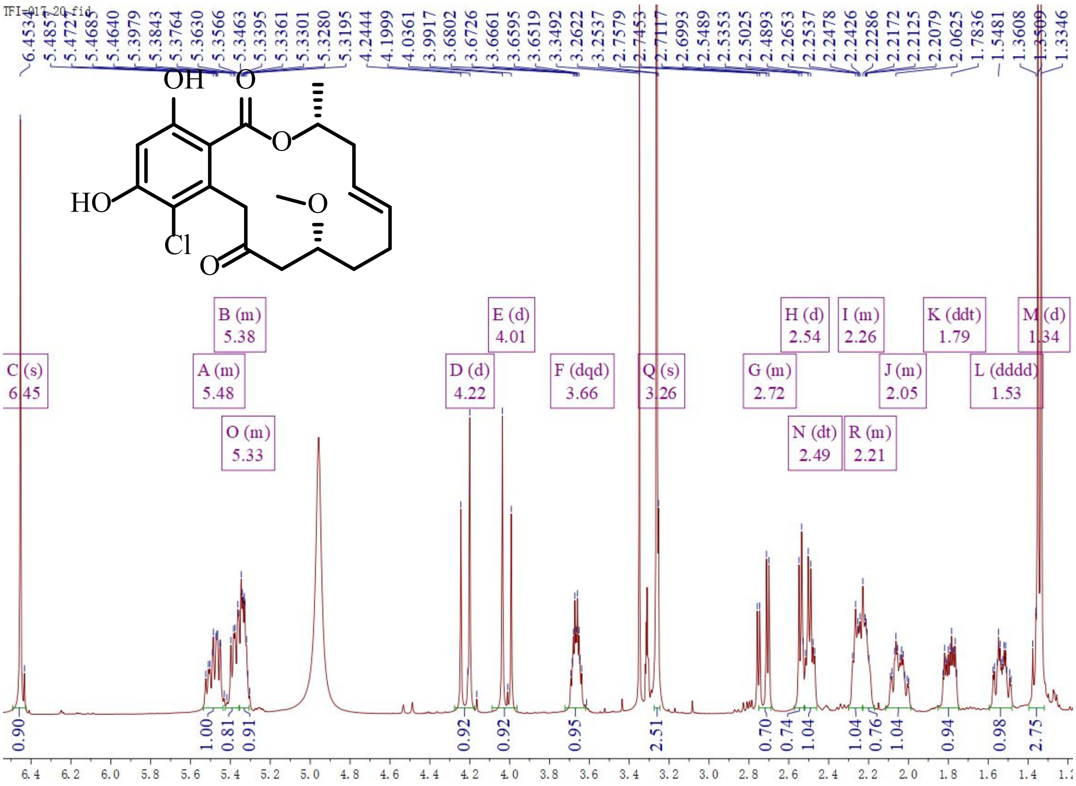

Figure S94 ^{13}C NMR spectrum (400 MHz, CD_3OD) of ilyolactone H (**8**)

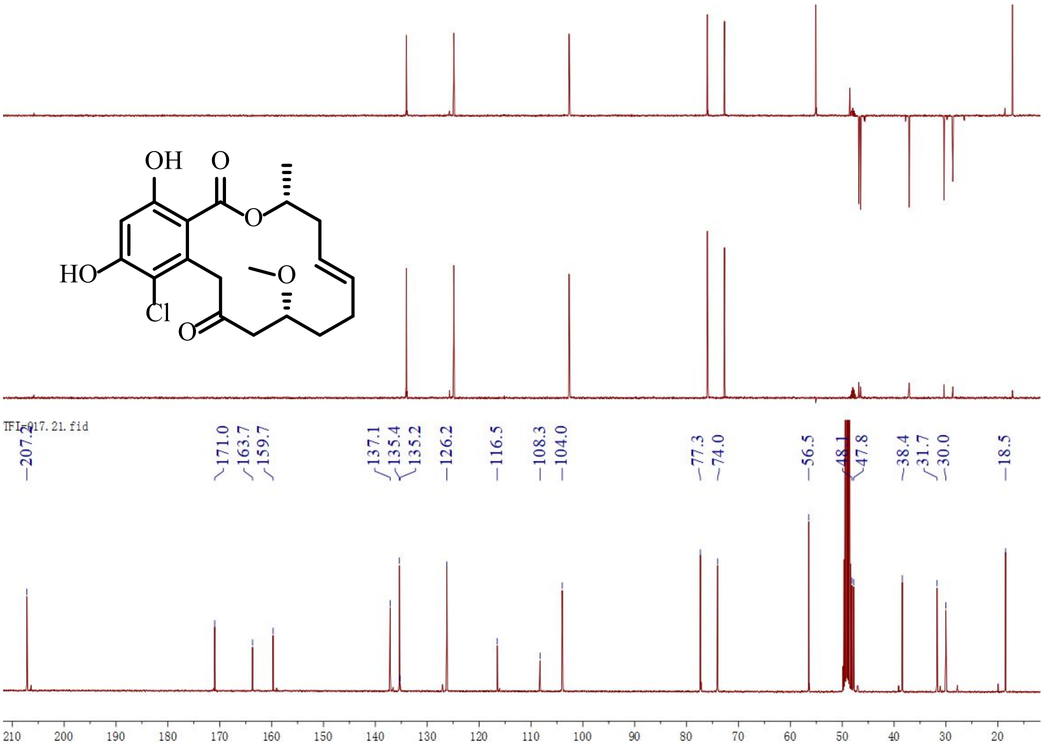

Figure S95 HSQC spectrum (400 MHz, CD_3OD) of ilyolactone H (**8**)

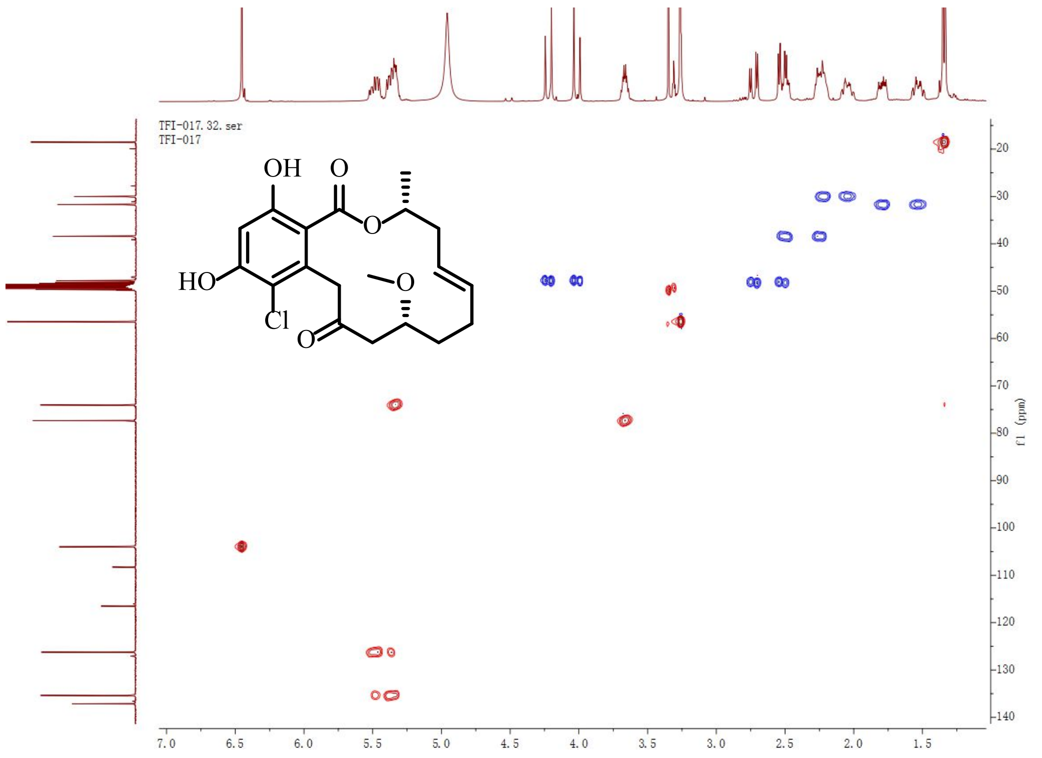

Figure S96 ^1H-^1H COSY spectrum (400 MHz, CD_3OD) of ilyolactone H (**8**)

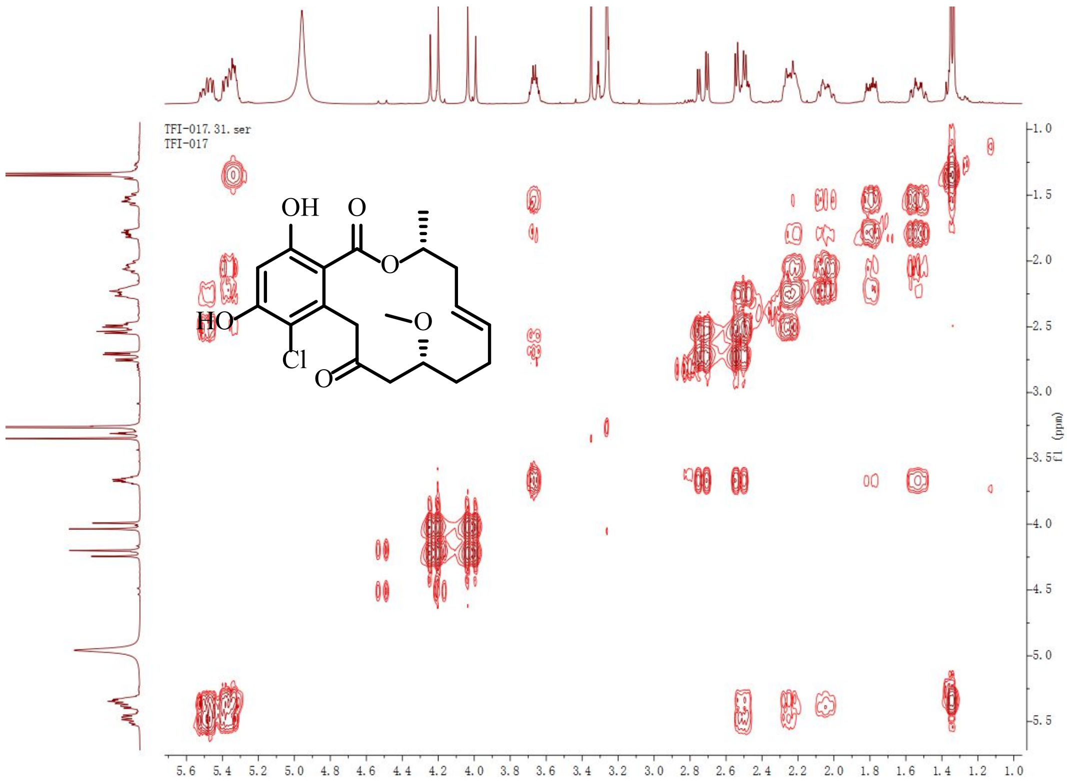

Figure S97 HMBC spectrum (400 MHz, CD_3OD) of ilyolactone H (**8**)

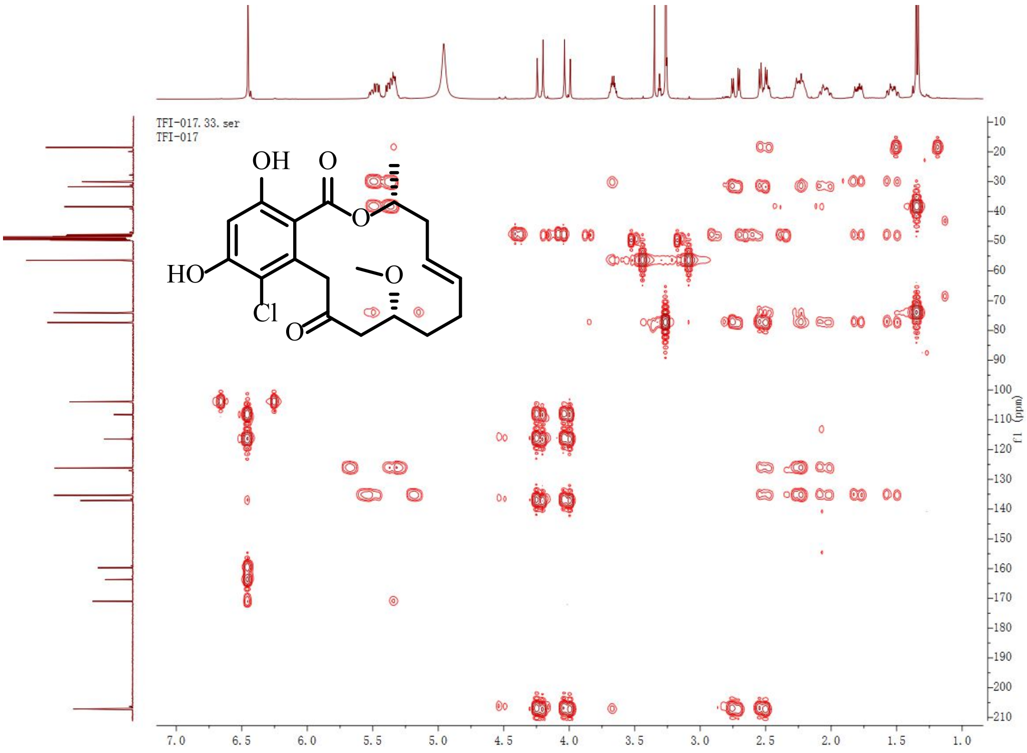

Figure S98 ROESY spectrum (400 MHz, CD_3OD) of ilyolactone H (**8**)

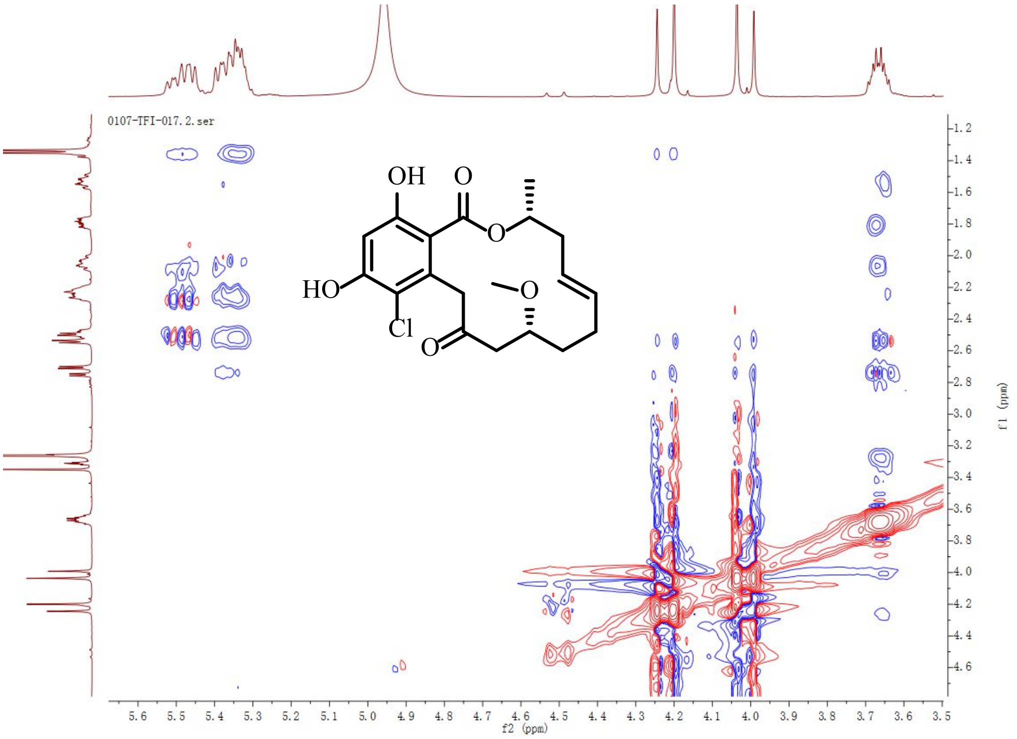

Figure S99 ESIMS spectrum of ilyolactone H (**8**)

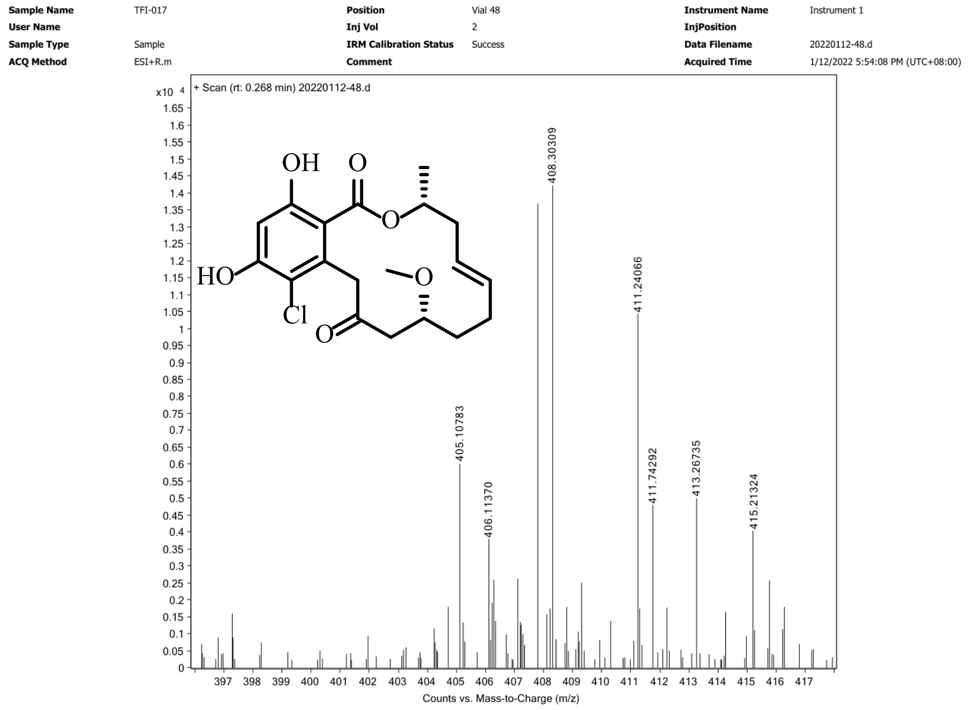

Figure S100 IR spectrum of ilyolactone H (**8**)

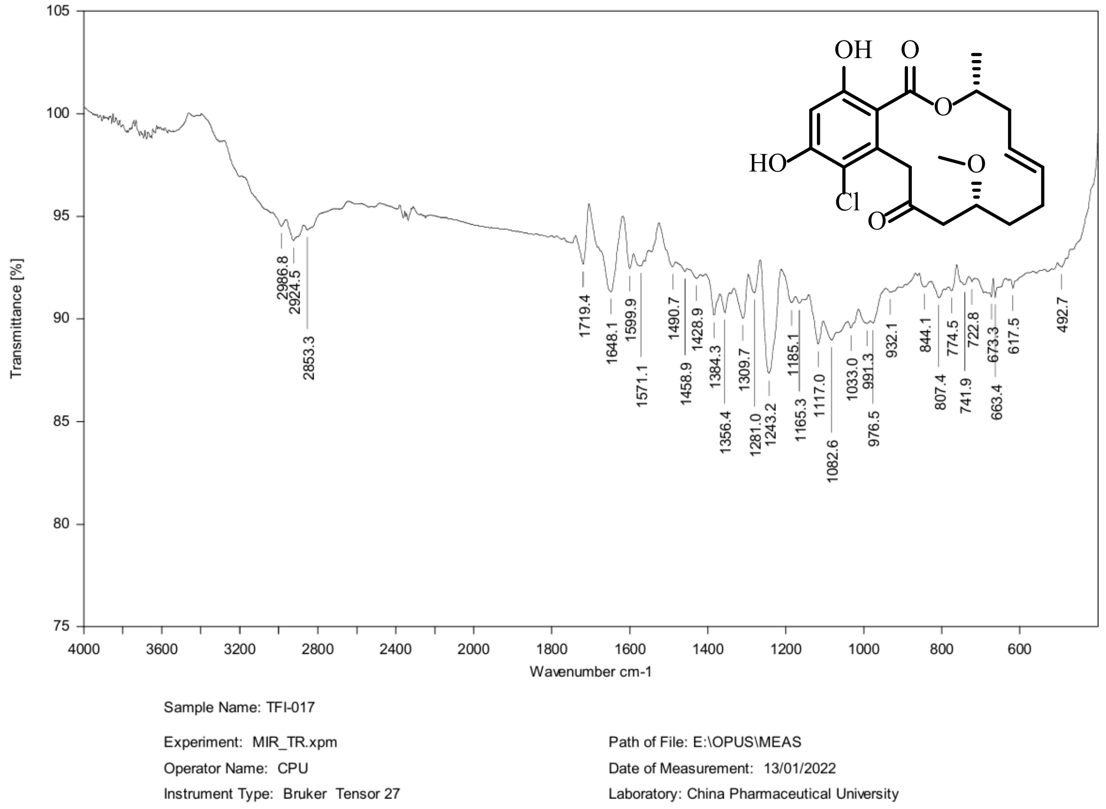

Figure S101 HRESIMS spectrum of ilyolactone H (**8**)

Qualitative Compound Identification Report

**Data File**	20220112-48.d	**Sample Name**	TFI-017
**Sample Type**	Sample	**Position**	Vial 48
**Instrument Name**	Instrument 1	**User Name**	
**Acq Method**	ESI+R.m	**Acquired Time**	1/12/2022 5:54:08 PM (UTC+08:00)
**IRM Calibration Status**	Success	**DA Method**	1.m
**Comment**			

**Sample Group**		**Info.**	
**Stream Name**	LC 1	**Acquisition Time (Local)**	1/12/2022 5:54:08 PM (UTC+08:00)
**Acquisition SW Version**	6200 series TOF/6500 series Q-TOF B.06.01 (B6157)	**TOF Driver Version**	6.00.01
**TOF Firmware Version**	17.643		

Compound Table

Compound Label	RT	Mass	Abund	Formula	Tgt Mass	Diff (ppm)	Hits (DB)
Cpd 1: C19 H23 Cl O6; 0.251	0.251	382.12024	3395	C19 H23 Cl O6	382.11832	5.04	1

Compound Label	*m/z*	RT	Algorithm	Mass
Cpd 1: C19 H23 Cl O6; 0.251	405.10822	0.251	Find By Formula	382.12024

MS Spectrum

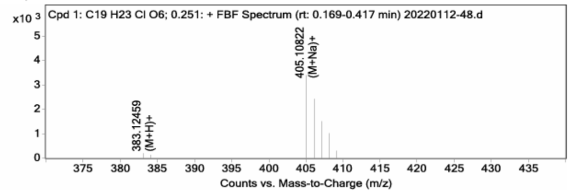

MS Spectrum Peak List

m/z	z	Abund	Ion
405.10822	1	3394.66	(M+Na)+
406.11294	1	2421.36	(M+Na)+
407.10954	1	1507.83	(M+Na)+
408.11072	1	1010.87	(M+Na)+
409.11566	1	299.81	(M+Na)+

MS Spectrum

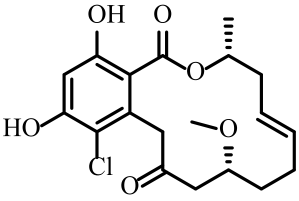

Qualitative Compound Identification Report

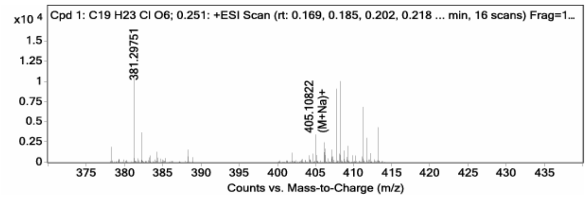

MS Spectrum Peak List

m/z	*Calc m/z*	Diff(ppm)	z	Abund	Ion
405.10822	405.10754	-1.69	1	3394.66	(M+Na)+
406.11294	406.11094	-4.94	1	2421.36	(M+Na)+
407.10954	407.10542	-10.13	1	1507.83	(M+Na)+
408.11072	408.10843	-5.6	1	1010.87	(M+Na)+
409.11566	409.11073	-12.05	1	299.81	(M+Na)+

--- End Of Report ---

Figure S102 UV spectrum of ilyolactone H (**8**)

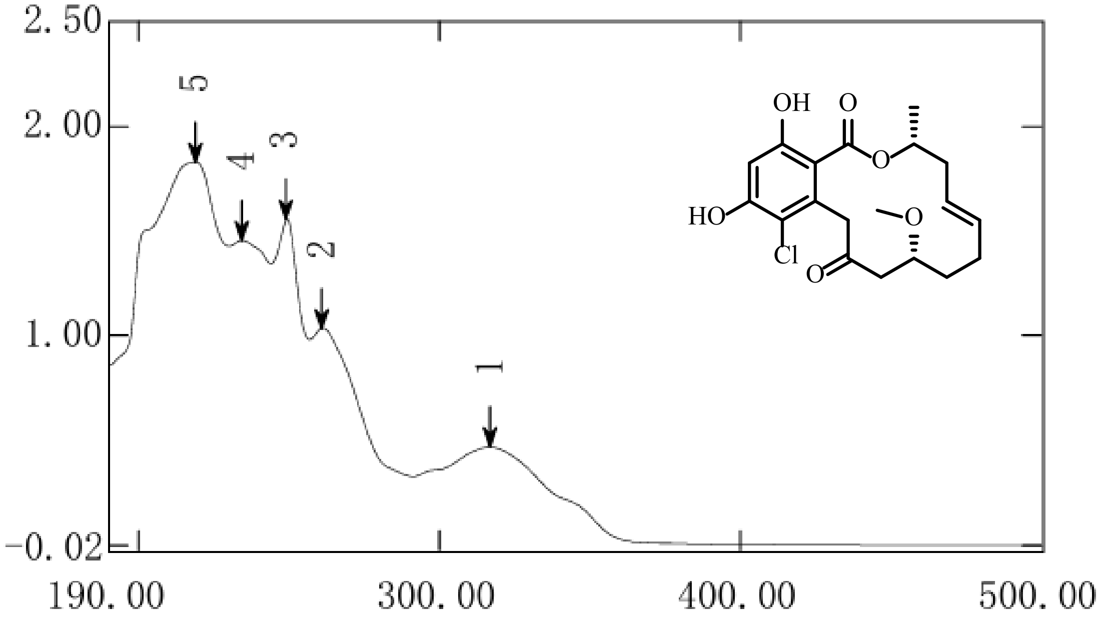

Figure S103 Optical rotation data of ilyolactone H (**8**)

Optical rotation test report

**Sample number**	TFI-017	**weight**	
**Inspection department**	School of Traditional Chinese Pharmacy		
**Inspection item**	Optical rotation		
**Date received**	2022-01-11	**Report data**	2022-01-14

According to the Chinese Pharmacopoeia (2020) of the fourth general rule 0621 spectrophotometric method:

Instrument: AUTOPOL-IV

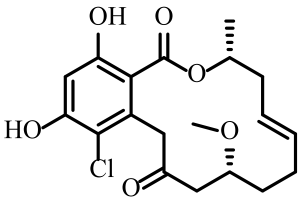

Light source: halogen tungsten lamp

Wave length: 589 nm

Solvent: Methanol

Temperature: 20°C

Result: $[\alpha]_D^{20}$-21.6 (c=0.04)

Notes: 1. Inspection report is only responsible for incoming samples

2. Additions, deletions and corrections to this report are invalid

Figure S104 ^{1}H NMR spectrum (400 MHz, CD_3OD) of ilyolactone I (**9**)

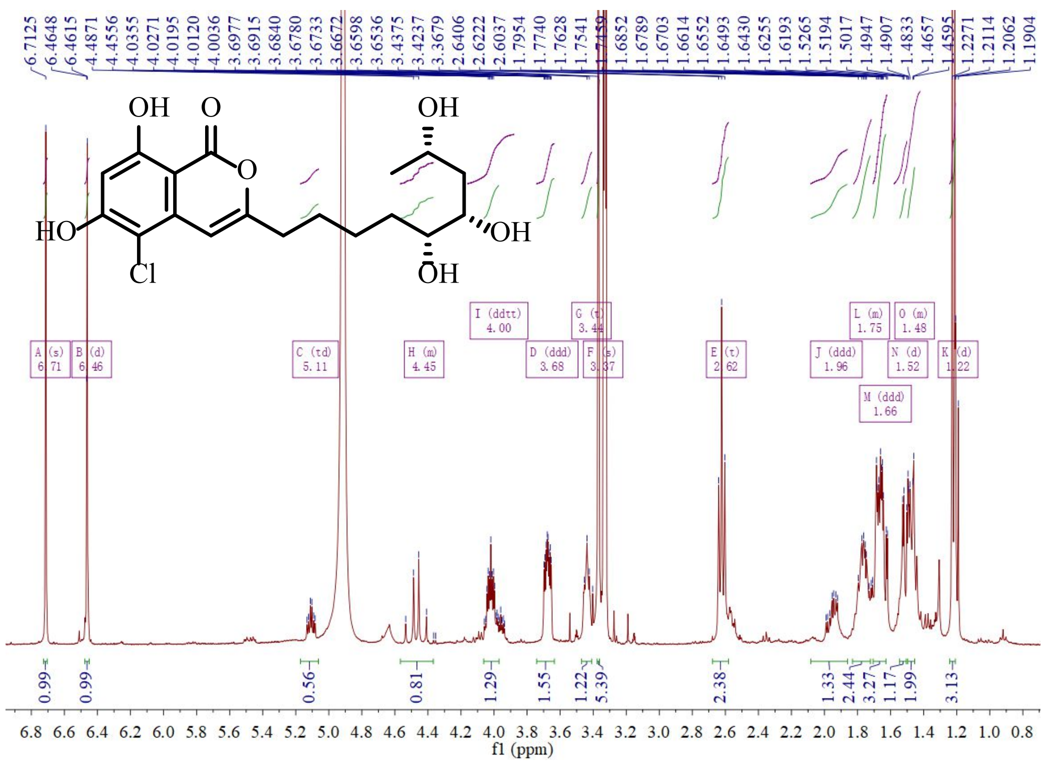

Figure S105 ^{13}C NMR spectrum (400 MHz, CD_3OD) of ilyolactone I (**9**)

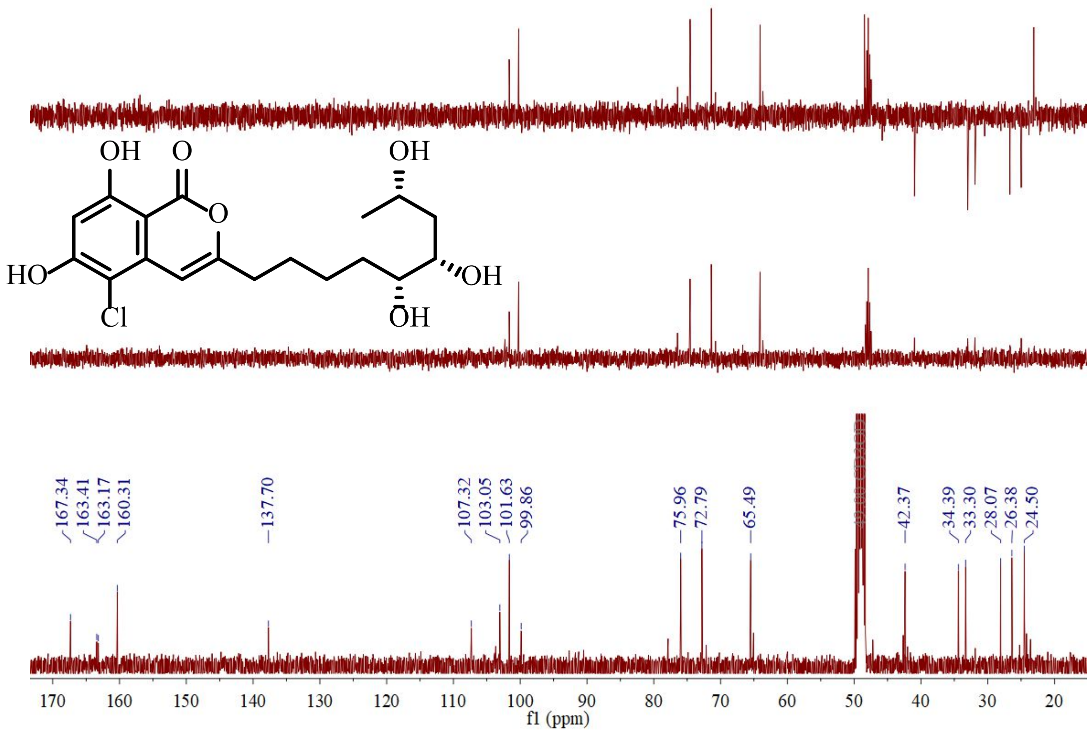

Figure S106 HSQC spectrum (400 MHz, CD_3OD) of ilyolactone I (**9**)

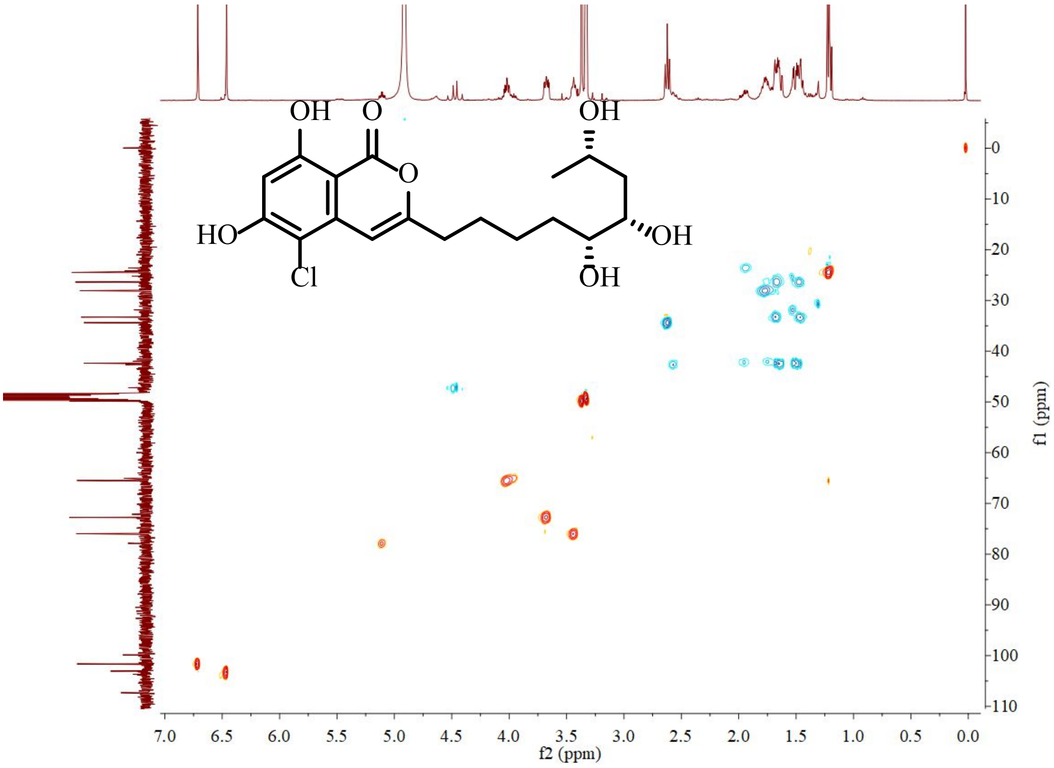

Figure S107 ^{1}H-^{1}H COSY spectrum (400 MHz, CD_3OD) of ilyolactone I (**9**)

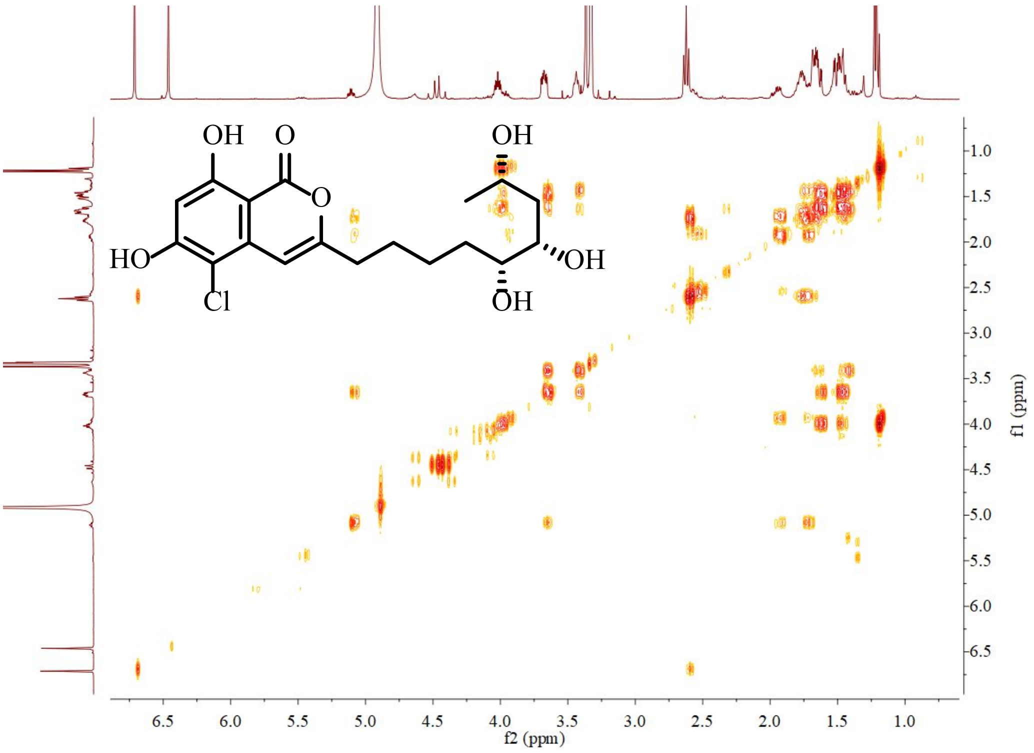

Figure S108 HMBC spectrum (400 MHz, CD_3OD) of ilyolactone I (**9**)

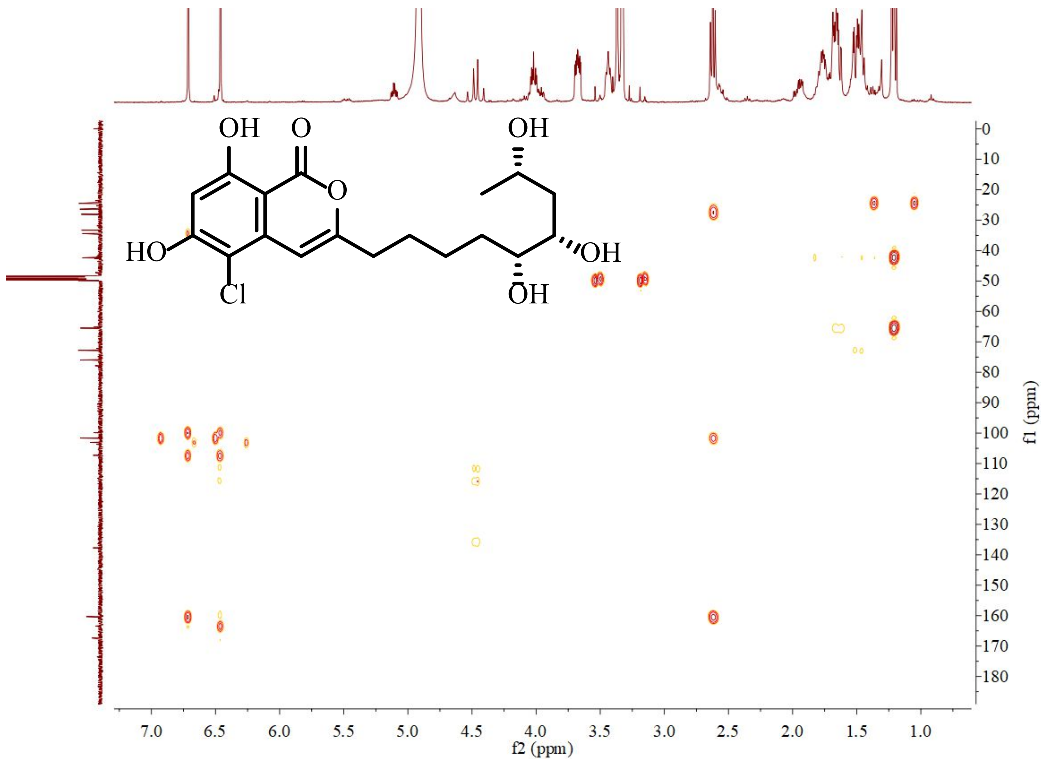

Figure S109 ROESY spectrum (400 MHz, CD_3OD) of ilyolactone I (**9**)

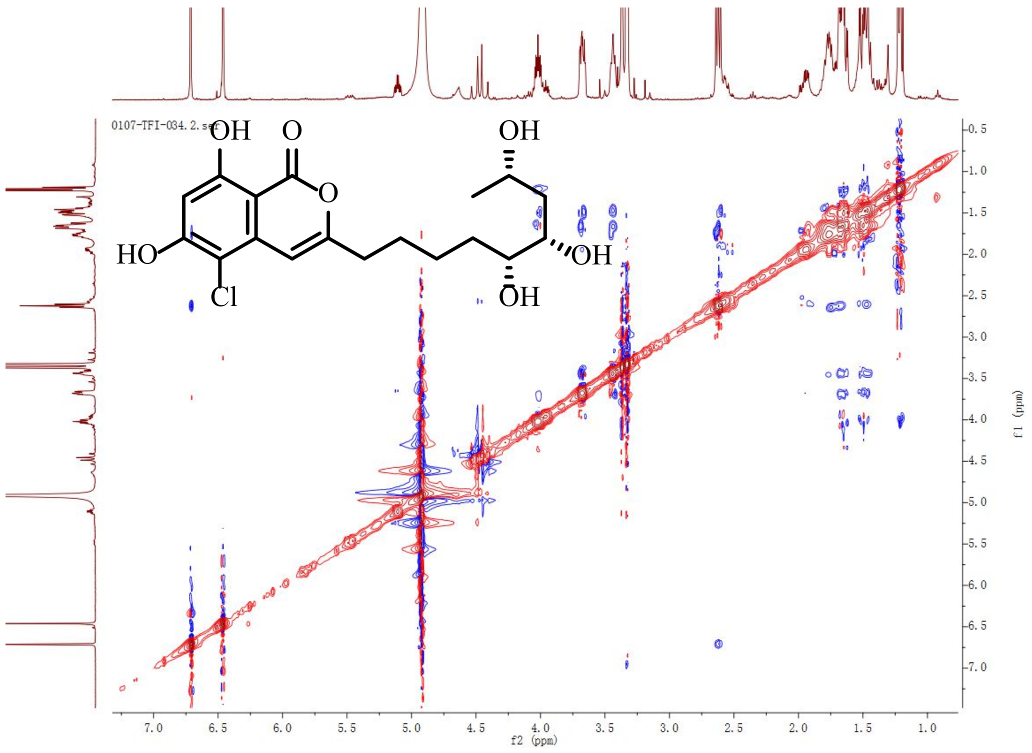

Figure S110 ESIMS spectrum of ilyolactone I (**9**)

**Sample Name**	TFI-034	**Position**	Vial 50	**Instrument Name**	Instrument 1
**User Name**		**Inj Vol**	2	**InjPosition**	
**Sample Type**	Sample	**IRM Calibration Status**	Success	**Data Filename**	20220112-50.d
**ACQ Method**	ESI+R.m	**Comment**		**Acquired Time**	1/12/2022 6:09:48 PM (UTC+08:00)

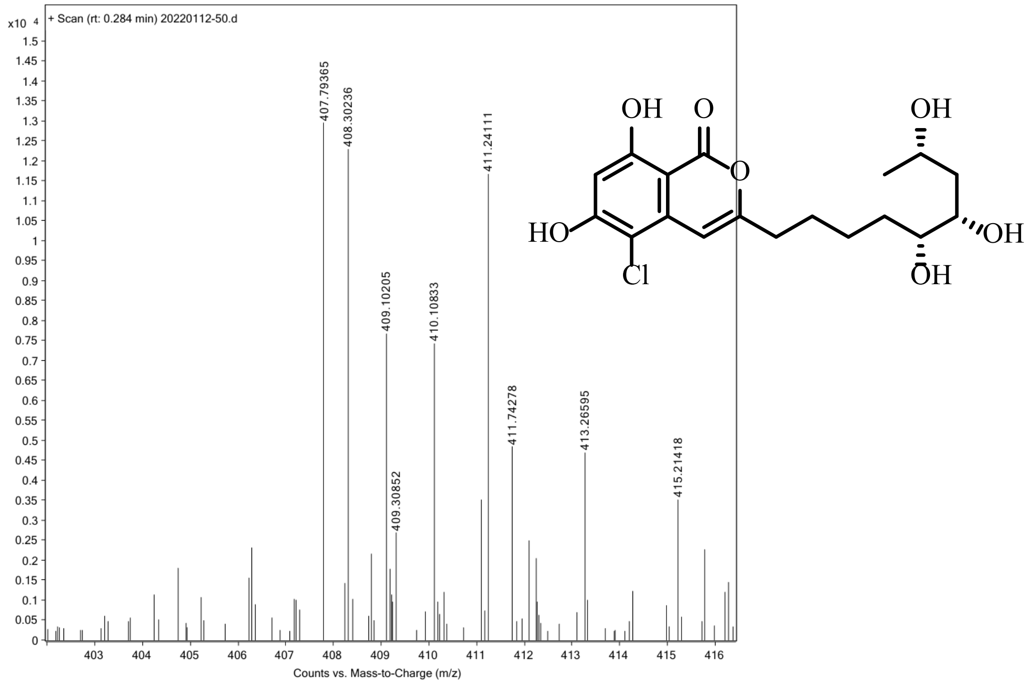

Figure S111 IR spectrum of ilyolactone I (**9**)

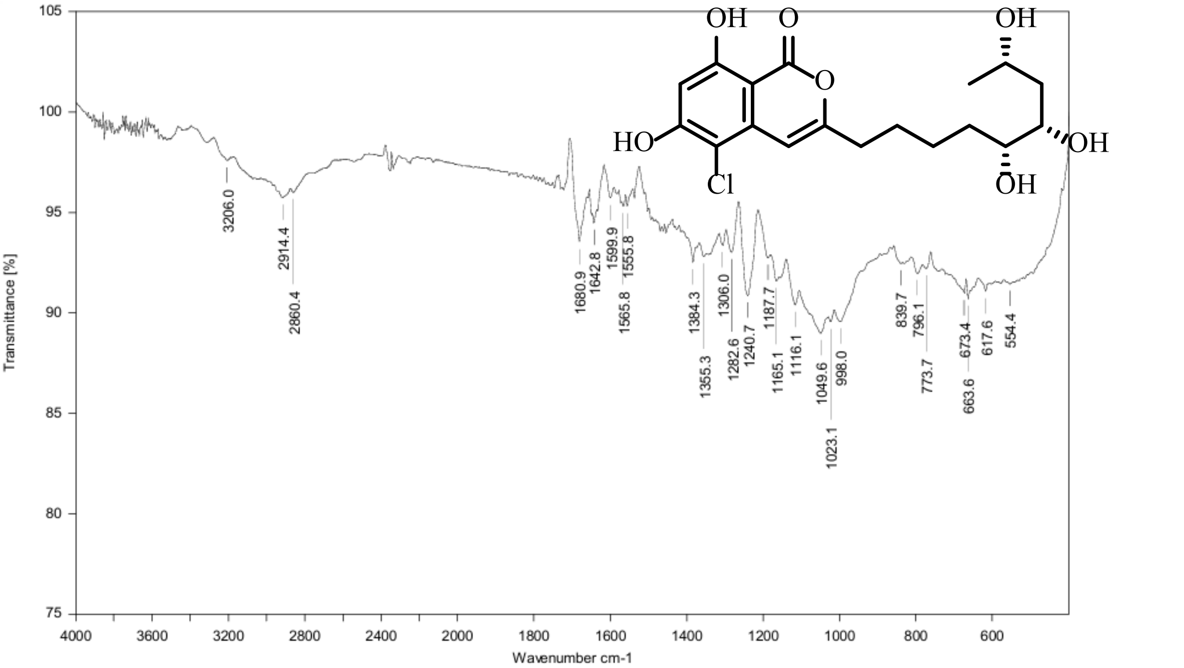

Sample Name: TFI-034

Experiment: MIR_TR.xpm | Path of File: E:\OPUS\MEAS

Operator Name: CPU | Date of Measurement: 13/01/2022

Instrument Type: Bruker Tensor 27 | Laboratory: China Pharmaceutical University

Figure S112 HRESIMS spectrum of ilyolactone I (**9**)

Qualitative Compound Identification Report

**Data File**	20220112-50.d	**Sample Name**	TFI-034
**Sample Type**	Sample	**Position**	Vial 50
**Instrument Name**	Instrument 1	**User Name**	
**Acq Method**	ESI+R.m	**Acquired Time**	1/12/2022 6:09:48 PM (UTC+08:00)
**IRM Calibration Status**	Success	**DA Method**	1.m
**Comment**			

**Sample Group**		**Info.**	
**Stream Name**	LC 1	**Acquisition Time (Local)**	1/12/2022 6:09:48 PM (UTC+08:00)
**Acquisition SW Version**	6200 series TOF/6500 series Q-TOF B.06.01 (B6157)	**TOF Driver Version**	6.00.01
**TOF Firmware Version**	17.643		

Compound Table

Compound Label	RT	Mass	Abund	Formula	Tgt Mass	Diff (ppm)	Hits (DB)
Cpd 1: C18 H23 Cl O7; 0.250	0.25	386.11507	5128	C18 H23 Cl O7	386.11323	4.75	1

Compound Label	*m/z*	RT	Algorithm	Mass
Cpd 1: C18 H23 Cl O7; 0.250	409.10239	0.25	Find By Formula	386.11507

MS Spectrum

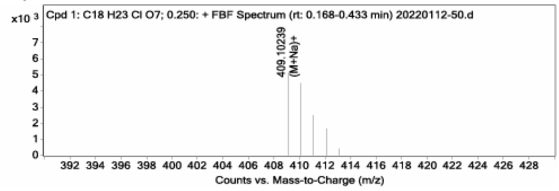

MS Spectrum Peak List

m/z	z	Abund	Ion
409.10239	1	5128.41	(M+Na)+
410.10827	1	4504.81	(M+Na)+
411.10408	1	2491.96	(M+Na)+
412.10627	1	1665.45	(M+Na)+
413.10871	1	419.3	(M+Na)+

MS Spectrum

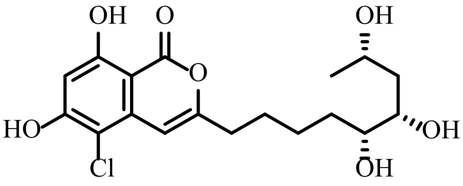

Qualitative Compound Identification Report

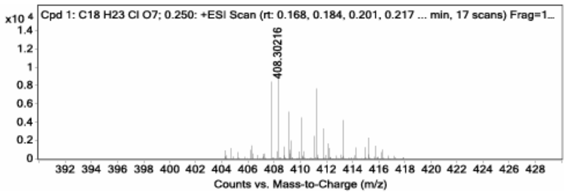

MS Spectrum Peak List

m/z	*Calc m/z*	Diff(ppm)	z	Abund	Ion
409.10239	409.10245	0.15	1	5128.41	(M+Na)+
410.10827	410.10586	-5.88	1	4504.81	(M+Na)+
411.10408	411.10032	-9.15	1	2491.96	(M+Na)+
412.10627	412.10337	-7.02	1	1665.45	(M+Na)+
413.10871	413.1055	-7.76	1	419.3	(M+Na)+

--- End Of Report ---

Figure S113 UV spectrum of ilyolactone I (**9**)

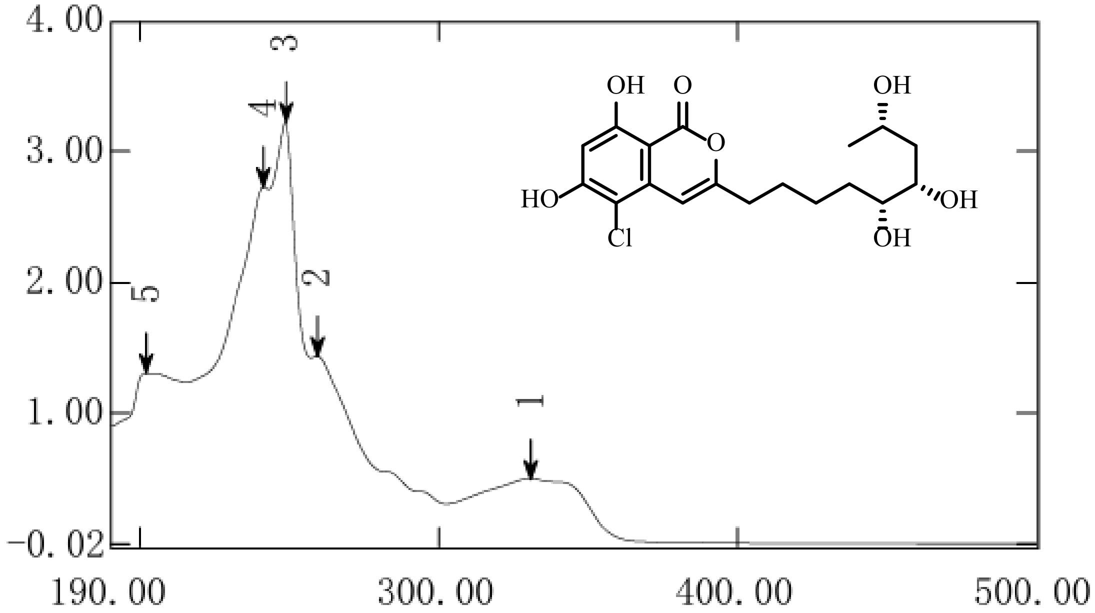

Figure S114 Optical rotation data of ilyolactone I (**9**)

Optical rotation test report

**Sample number**	TFI-034	**weight**	
**Inspection department**	School of Traditional Chinese Pharmacy		
**Inspection item**	Optical rotation		
**Date received**	2022-01-11	**Report data**	2022-01-14

According to the Chinese Pharmacopoeia (2020) of the fourth general rule 0621 spectrophotometric method:

Instrument: AUTOPOL-IV

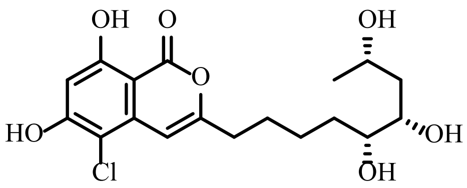

Light source: halogen tungsten lamp

Wave length: 589 nm

Solvent: Methanol

Temperature: 20°C

Result: $[\alpha]_D^{20}$-12.5 (c=0.04)

Notes: 1. Inspection report is only responsible for incoming samples

2. Additions, deletions and corrections to this report are invalid

Table S8 Energies of configurations **3**–**7** at B3LYP/6-311G (d, p) in methanol

Conformer	Structure	Energy (Hartree)	Energy (kcal/mol)	Population (%)
**3b**-1	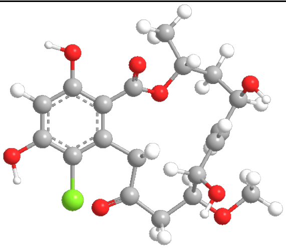	-1800.256402	-1129677.939	100
**3c**-1	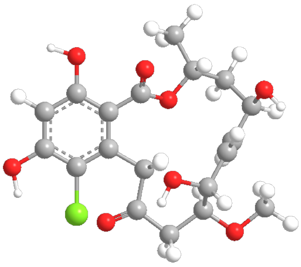	-1800.263168	-1129682.184	100
**4b**-1	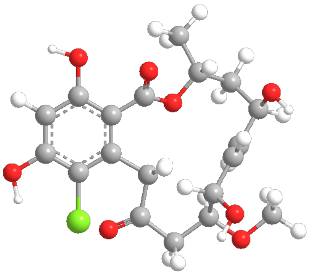	-1800.256402	-1129677.939	100
**4c**-1	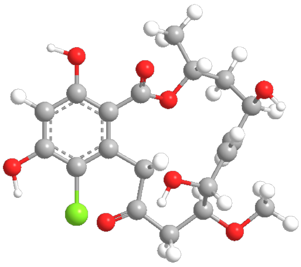	-1800.263168	-1129682.184	100
**5a**-1	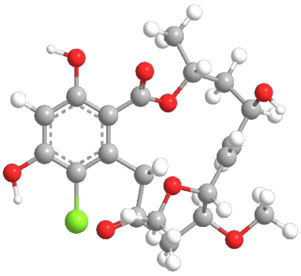	-1839.57	-1154346	100
**5b**-1	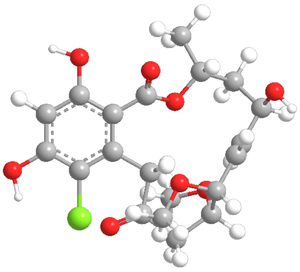	-1839.560312	-1154341.515	100

**5c-**1	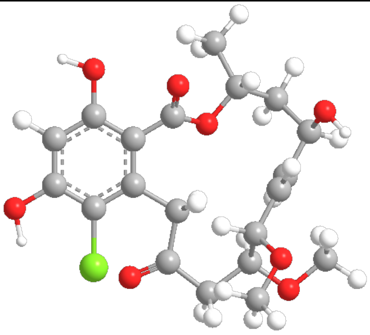	-1839.567286	-1154345.891	100
**5d-**1	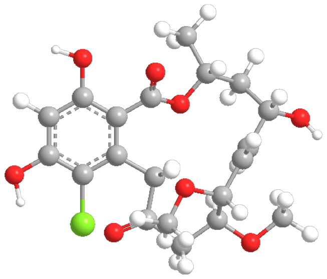	-1839.563169	-1154343.307	100
**5e-**1	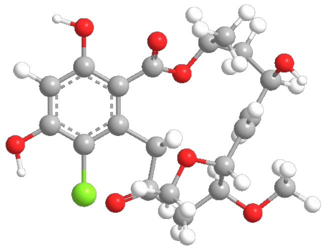	-1839.565384	-1154344.697	100
**5f-**1	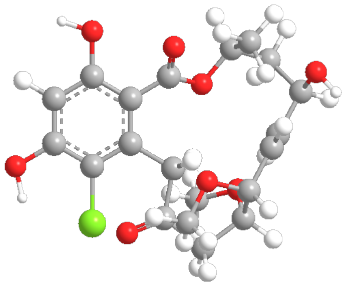	-1839.565384	-1154344.697	100
**5g-**1	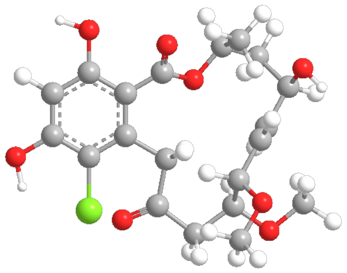	-1839.565502	-1154344.771	100
**5h-**1	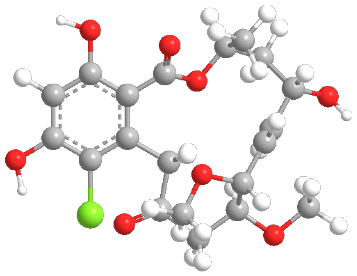	-1839.565384	-1154344.697	100

**6a-**1	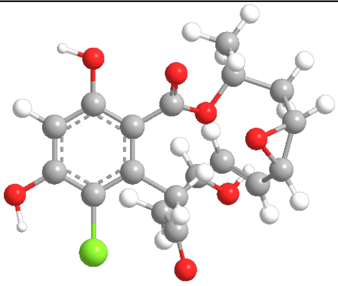	-1684.446243	-1057005.967	100
**6b-**1	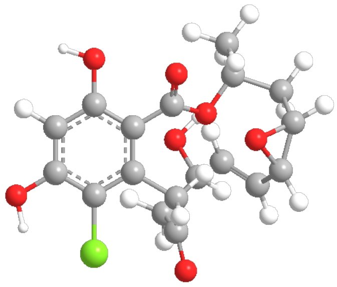	-1684.457394	-1057012.965	100
**6c-**1	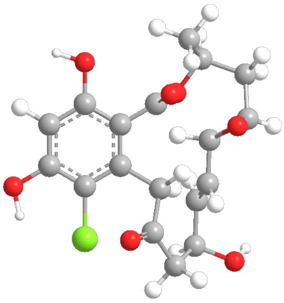	-1684.459984	-1057014.59	100
**6d-**1	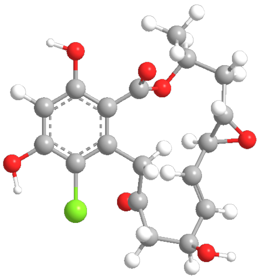	-1684.443593	-1057004.305	100
**6e-**1	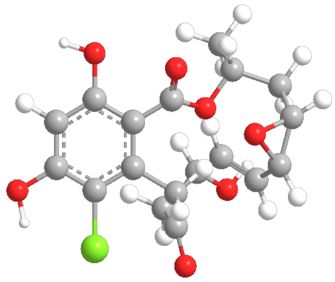	-1684.457339	-1057012.93	100
**6f-**1	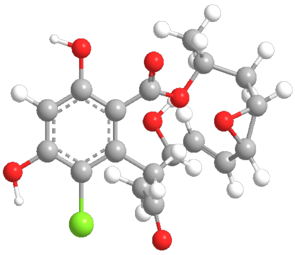	-1684.445339	-1057005.4	100

**6g-**1	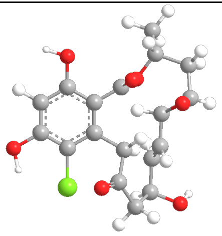	-1684.450527	-1057008.656	100
**6h-**1	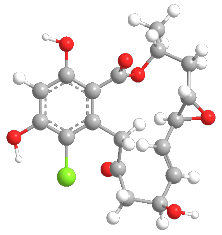	-1684.446484	-1057006.119	100
**7a-1**	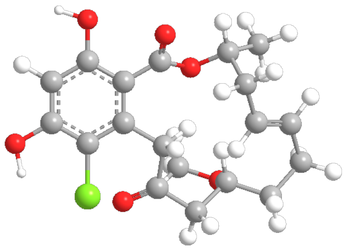	-1649.766518	-1035244.111	4.03
**7a-2**		-1649.769512	-1035245.99	95.97

Table S9 Standard orientations of the conformers of **3**–**7** at mPW1PW91/6-311G (2d, p) in gas phase

Standard orientation of **3b**-1

Center Number	Atomic Number	Atomic Type	Coordinates (Angstroms) X	Y	Z
1	6	C	-4.549786	0.155578	0.206846
2	6	C	-4.153338	-1.076264	-0.283799
3	6	C	-2.790178	-1.304821	-0.560795
4	6	C	-1.825671	-0.309994	-0.3847
5	6	C	-2.230928	0.96709	0.117268
6	6	C	-3.612725	1.168038	0.430515
7	6	C	-0.386382	-0.664775	-0.700094
8	8	O	-0.097507	2.065434	-0.117465
9	6	C	-1.344817	2.130055	0.361771
10	6	C	2.8353	1.924801	-0.868694
11	6	C	2.191786	2.494735	0.414205
12	6	C	0.828039	3.156512	0.226706
13	6	C	0.755749	4.240084	-0.841269
14	8	O	-4.105685	2.305545	0.9367
15	8	O	-5.027207	-2.088306	-0.49957
16	8	O	-1.733238	3.14371	0.957376
17	17	Cl	-2.36634	-2.922191	-1.115839
18	8	O	3.745499	2.922558	-1.349565
19	6	C	3.512298	0.614821	-0.573409
20	6	C	3.067822	-0.554056	-1.042742
21	6	C	0.383976	-1.177685	0.537763
22	8	O	0.316909	-0.574772	1.592533
23	6	C	1.178813	-2.47861	0.416921
24	6	C	3.522525	-1.904708	-0.567528
25	6	C	2.715708	-2.408385	0.654022
26	8	O	3.36721	-2.905135	-1.575421
27	8	O	3.118488	-1.602189	1.736996
28	6	C	2.730011	-2.075792	3.016635

29	1	H	-5.589119	0.35937	0.448389
30	1	H	-0.355866	-1.409388	-1.495262
31	1	H	0.142397	0.22078	-1.046348
32	1	H	2.050919	1.743206	-1.620441
33	1	H	2.887708	3.231901	0.831631
34	1	H	2.070664	1.697742	1.155017
35	1	H	0.492764	3.562048	1.183101
36	1	H	-0.25145	4.663965	-0.881023
37	1	H	1.462873	5.040833	-0.600689
38	1	H	1.017738	3.849852	-1.829217
39	1	H	-3.328961	2.904099	1.104303
40	1	H	-5.917686	-1.787988	-0.256826
41	1	H	4.158293	2.573721	-2.155649
42	1	H	4.331187	0.645752	0.145695
43	1	H	2.242205	-0.570628	-1.755749
44	1	H	1.021523	-2.957897	-0.55301
45	1	H	0.751954	-3.147697	1.174393
46	1	H	4.566777	-1.851054	-0.225414
47	1	H	3.045755	-3.448652	0.815509
48	1	H	3.848977	-2.604908	-2.361387
49	1	H	3.252126	-1.453242	3.747896
50	1	H	1.650229	-1.974065	3.171294
51	1	H	3.031814	-3.125006	3.16264

Standard orientation of **3c**-1

Center	Atomic	Atomic	Coordinates (Angstroms)		
Number	Number	Type	X	Y	Z
1	6	C	-4.064159	1.029361	-0.676229
2	6	C	-4.333817	-0.325898	-0.574015
3	6	C	-3.357865	-1.182276	-0.015333
4	6	C	-2.14183	-0.705439	0.462826
5	6	C	-1.876232	0.694158	0.369552
6	6	C	-2.846296	1.544279	-0.230827
7	6	C	-1.103316	-1.661701	1.013356

8	8	O	-0.262631	2.44666	0.425263
9	6	C	-0.624054	1.231378	0.95242
10	6	C	2.193634	2.457446	-1.250265
11	6	C	1.781476	3.552851	-0.247559
12	6	C	0.956119	3.081127	0.946413
13	6	C	0.542394	4.202357	1.891858
14	8	O	-2.692425	2.874706	-0.390348
15	8	O	-5.524736	-0.768128	-1.030086
16	8	O	0.05065	0.691654	1.808977
17	17	Cl	-3.775446	-2.905746	0.02584
18	8	O	2.978048	3.056804	-2.295238
19	6	C	2.911056	1.297845	-0.600861
20	6	C	2.766538	0.030742	-0.997635
21	6	C	0.149816	-1.744226	0.12906
22	8	O	0.08009	-1.573859	-1.074278
23	6	C	1.449306	-2.0911	0.834661
24	6	C	3.508798	-1.137504	-0.413258
25	6	C	2.616351	-2.372674	-0.117142
26	8	O	4.185332	-0.748199	0.770958
27	8	O	3.446705	-3.356942	0.523395
28	6	C	4.143848	-4.21264	-0.368961
29	1	H	-4.795986	1.694446	-1.118941
30	1	H	-1.512826	-2.6766	1.049058
31	1	H	-0.820812	-1.388958	2.030238
32	1	H	1.300415	2.087175	-1.76241
33	1	H	2.694217	4.014105	0.157751
34	1	H	1.25031	4.340223	-0.79613
35	1	H	1.490572	2.305469	1.495254
36	1	H	1.43137	4.687976	2.309182
37	1	H	-0.047921	4.964241	1.370046
38	1	H	-0.050332	3.806902	2.72272
39	1	H	-1.770144	3.106955	-0.160904
40	1	H	-5.568406	-1.734309	-0.917461

41	1	H	3.871362	3.195644	-1.940835
42	1	H	3.601823	1.522239	0.21221
43	1	H	2.084875	-0.212526	-1.810161
44	1	H	1.284148	-2.975762	1.463862
45	1	H	1.687954	-1.268385	1.520136
46	1	H	4.246423	-1.467366	-1.17056
47	1	H	2.225899	-2.771543	-1.062416
48	1	H	4.460262	-1.584679	1.187074
49	1	H	4.711078	-4.914671	0.24765
50	1	H	3.444948	-4.775494	-1.004591
51	1	H	4.84449	-3.667485	-1.017555

Standard orientation of **4c**-1

Center	Atomic	Atomic	Coordinates (Angstroms)		
Number	Number	Type	X	Y	Z
1	6	C	-4.064159	1.029361	-0.676229
2	6	C	-4.333817	-0.325898	-0.574015
3	6	C	-3.357865	-1.182276	-0.015333
4	6	C	-2.14183	-0.705439	0.462826
5	6	C	-1.876232	0.694158	0.369552
6	6	C	-2.846296	1.544279	-0.230827
7	6	C	-1.103316	-1.661701	1.013356
8	8	O	-0.262631	2.44666	0.425263
9	6	C	-0.624054	1.231378	0.95242
10	6	C	2.193634	2.457446	-1.250265
11	6	C	1.781476	3.552851	-0.247559
12	6	C	0.956119	3.081127	0.946413
13	6	C	0.542394	4.202357	1.891858
14	8	O	-2.692425	2.874706	-0.390348
15	8	O	-5.524736	-0.768128	-1.030086
16	8	O	0.05065	0.691654	1.808977
17	17	Cl	-3.775446	-2.905746	0.02584
18	8	O	2.978048	3.056804	-2.295238
19	6	C	2.911056	1.297845	-0.600861

20	6	C	2.766538	0.030742	-0.997635
21	6	C	0.149816	-1.744226	0.12906
22	8	O	0.08009	-1.573859	-1.074278
23	6	C	1.449306	-2.0911	0.834661
24	6	C	3.508798	-1.137504	-0.413258
25	6	C	2.616351	-2.372674	-0.117142
26	8	O	4.185332	-0.748199	0.770958
27	8	O	3.446705	-3.356942	0.523395
28	6	C	4.143848	-4.21264	-0.368961
29	1	H	-4.795986	1.694446	-1.118941
30	1	H	-1.512826	-2.6766	1.049058
31	1	H	-0.820812	-1.388958	2.030238
32	1	H	1.300415	2.087175	-1.76241
33	1	H	2.694217	4.014105	0.157751
34	1	H	1.25031	4.340223	-0.79613
35	1	H	1.490572	2.305469	1.495254
36	1	H	1.43137	4.687976	2.309182
37	1	H	-0.047921	4.964241	1.370046
38	1	H	-0.050332	3.806902	2.72272
39	1	H	-1.770144	3.106955	-0.160904
40	1	H	-5.568406	-1.734309	-0.917461
41	1	H	3.871362	3.195644	-1.940835
42	1	H	3.601823	1.522239	0.21221
43	1	H	2.084875	-0.212526	-1.810161
44	1	H	1.284148	-2.975762	1.463862
45	1	H	1.687954	-1.268385	1.520136
46	1	H	4.246423	-1.467366	-1.17056
47	1	H	2.225899	-2.771543	-1.062416
48	1	H	4.460262	-1.584679	1.187074
49	1	H	4.711078	-4.914671	0.24765
50	1	H	3.444948	-4.775494	-1.004591
51	1	H	4.84449	-3.667485	-1.017555

Standard orientation of **5a**-1

Center	Atomic	Atomic	Coordinates (Angstroms)		
Number	Number	Type	X	Y	Z
1	6	C	-4.68029	-1.00421	-0.721388
2	6	C	-3.896986	-2.128708	-0.513488
3	6	C	-2.619687	-1.973503	0.064912
4	6	C	-2.086191	-0.727501	0.387212
5	6	C	-2.876895	0.430296	0.135112
6	6	C	-4.204894	0.259991	-0.373488
7	6	C	-0.745091	-0.696397	1.089112
8	8	O	-1.1241	2.057702	0.332512
9	6	C	-2.4465	1.829897	0.348812
10	6	C	1.777397	2.923312	-0.097988
11	6	C	0.790895	3.300108	1.028712
12	6	C	-0.668905	3.433903	0.595812
13	6	C	-0.892908	4.329303	-0.618688
14	8	O	-5.055398	1.279788	-0.564988
15	8	O	-4.411782	-3.330509	-0.848188
16	8	O	-3.250603	2.757395	0.489512
17	17	Cl	-1.724082	-3.4657	0.402112
18	8	O	2.456292	4.140414	-0.446888
19	6	C	2.7424	1.843115	0.314412
20	6	C	2.932404	0.731816	-0.404088
21	6	C	0.45401	-1.082793	0.212212
22	8	O	0.41171	-1.030693	-1.000388
23	6	C	1.682812	-1.550288	0.985112
24	6	C	3.783408	-0.436881	0.013412
25	6	C	2.958713	-1.737584	0.152512
26	8	O	4.801909	-0.708378	-0.950988
27	8	O	3.732716	-2.736981	0.808512
28	6	C	4.440919	-3.621379	-0.048288
29	6	C	5.860806	0.231126	-0.946788
30	1	H	-5.67789	-1.105814	-1.131588
31	1	H	-0.531494	0.299604	1.480512

32	1	H	-0.772589	-1.371397	1.953312
33	1	H	1.215398	2.54801	-0.965188
34	1	H	1.118892	4.26091	1.441512
35	1	H	0.839898	2.568909	1.843712
36	1	H	-1.265706	3.804201	1.434412
37	1	H	-0.411107	3.920704	-1.511488
38	1	H	-0.456312	5.314504	-0.424688
39	1	H	-1.959609	4.447599	-0.814988
40	1	H	-4.637801	2.08739	-0.174488
41	1	H	-3.75928	-4.023707	-0.644188
42	1	H	3.076893	3.926316	-1.161888
43	1	H	3.2767	1.995617	1.254312
44	1	H	2.398305	0.602514	-1.344788
45	1	H	1.430115	-2.528589	1.415912
46	1	H	1.85141	-0.885288	1.843012
47	1	H	4.245707	-0.23668	0.994712
48	1	H	2.675414	-2.069185	-0.855388
49	1	H	3.759321	-4.130481	-0.747188
50	1	H	5.214417	-3.102776	-0.623588
51	1	H	4.904122	-4.371377	0.599512
52	1	H	5.511902	1.248125	-1.176588
53	1	H	6.567207	-0.086172	-1.718088
54	1	H	6.377006	0.252328	0.025812

Standard orientation of **5b**-1

Center	Atomic	Atomic	Coordinates (Angstroms)		
Number	Number	Type	X	Y	Z
1	6	C	-4.600009	0.536075	0.0075
2	6	C	-4.501704	-0.844925	-0.0849
3	6	C	-3.232903	-1.451621	0.0245
4	6	C	-2.073705	-0.714617	0.267
5	6	C	-2.188509	0.697883	0.3766
6	6	C	-3.458411	1.310779	0.2082
7	6	C	-0.732103	-1.411513	0.3339

8	8	O	-1.036916	2.760086	0.1464
9	6	C	-0.985312	1.508986	0.6948
10	6	C	2.496185	2.414797	0.129
11	6	C	1.282484	2.967093	-0.6461
12	6	C	0.176482	3.58569	0.2364
13	6	C	-0.249823	4.979189	-0.1994
14	8	O	-3.660215	2.646678	0.2615
15	8	O	-5.638502	-1.545628	-0.2953
16	8	O	-0.038111	1.150589	1.3677
17	17	Cl	-3.192897	-3.214421	-0.187
18	8	O	3.477982	3.4657	0.1367
19	6	C	3.024489	1.156699	-0.5083
20	6	C	3.005193	-0.035701	0.0952
21	6	C	0.130597	-1.20171	-0.9198
22	8	O	0.017894	-0.215111	-1.623
23	6	C	1.1301	-2.305407	-1.2799
24	6	C	3.420597	-1.3285	-0.5513
25	6	C	2.378601	-2.462203	-0.3824
26	8	O	4.634899	-1.844096	0.016
27	8	O	1.926901	-2.613705	0.9549
28	6	C	2.764703	-3.376602	1.8157
29	6	C	5.789996	-1.097293	-0.3166
30	1	H	-5.56491	1.017572	-0.0984
31	1	H	-0.140304	-1.020311	1.1655
32	1	H	-0.850399	-2.478513	0.5174
33	1	H	2.190086	2.178896	1.1552
34	1	H	0.829586	2.174392	-1.2487
35	1	H	1.665081	3.719995	-1.3449
36	1	H	0.481382	3.592191	1.2868
37	1	H	-0.596923	4.975088	-1.2384
38	1	H	0.607075	5.656591	-0.1258
39	1	H	-1.052124	5.367686	0.4372
40	1	H	-2.789417	3.089381	0.2253

41	1	H	-5.418399	-2.491928	-0.357
42	1	H	4.231983	3.147203	0.6578
43	1	H	3.371289	1.252	-1.5392
44	1	H	2.645693	-0.117102	1.1194
45	1	H	1.4504	-2.119706	-2.3097
46	1	H	0.597303	-3.263709	-1.2534
47	1	H	3.575997	-1.169299	-1.6319
48	1	H	2.892303	-3.382702	-0.7019
49	1	H	2.200204	-3.522004	2.7407
50	1	H	3.706902	-2.863699	2.03
51	1	H	2.990407	-4.359201	1.3749
52	1	H	5.948596	-1.069192	-1.4067
53	1	H	6.637598	-1.60339	0.1528
54	1	H	5.736693	-0.063793	0.0521

Standard orientation of **5c**-1

Center	Atomic	Atomic	Coordinates (Angstroms)		
Number	Number	Type	X	Y	Z
1	6	C	-4.613993	-0.801625	-0.606904
2	6	C	-4.006184	-1.91922	-0.064204
3	6	C	-2.692485	-1.800909	0.438696
4	6	C	-1.981995	-0.602604	0.427796
5	6	C	-2.611804	0.555591	-0.124804
6	6	C	-3.939403	0.419681	-0.655904
7	6	C	-0.562794	-0.636992	0.952296
8	8	O	-0.861617	2.122105	0.438796
9	6	C	-2.035215	1.918196	-0.182204
10	6	C	1.943176	2.977028	-0.478204
11	6	C	1.127473	3.418021	0.758396
12	6	C	-0.376928	3.508609	0.534796
13	6	C	-1.109234	4.211703	1.672696
14	8	O	-4.621011	1.426775	-1.214704
15	8	O	-4.700975	-3.075926	-0.046804
16	8	O	-2.608423	2.860191	-0.743704

17	17	Cl	-1.968573	-3.298304	1.065396
18	8	O	2.668167	4.144034	-0.901204
19	6	C	2.874386	1.836236	-0.167804
20	6	C	2.934195	0.718236	-0.895004
21	6	C	0.447109	-1.101384	-0.105204
22	8	O	0.215709	-1.004186	-1.295004
23	6	C	1.742914	-1.698174	0.434696
24	6	C	3.770204	-0.489957	-0.551504
25	6	C	2.884115	-1.759564	-0.595504
26	8	O	4.364003	-0.287652	0.720196
27	8	O	3.740724	-2.878657	-0.380004
28	6	C	3.251234	-4.094261	-0.911804
29	6	C	5.616108	-0.925642	0.932496
30	1	H	-5.618093	-0.869033	-1.008204
31	1	H	-0.492089	-1.293492	1.824396
32	1	H	-0.235202	0.35051	1.276596
33	1	H	1.253879	2.660223	-1.277204
34	1	H	1.491265	4.411024	1.044296
35	1	H	1.315478	2.752323	1.609396
36	1	H	-0.599032	3.998308	-0.416004
37	1	H	-0.91393	3.712705	2.628596
38	1	H	-0.764242	5.248506	1.748996
39	1	H	-2.186934	4.224395	1.491796
40	1	H	-4.025117	2.22168	-1.206704
41	1	H	-4.145869	-3.774021	0.343596
42	1	H	3.229569	3.874138	-1.645504
43	1	H	3.510485	1.943641	0.708296
44	1	H	2.295896	0.608031	-1.770704
45	1	H	1.490122	-2.715376	0.766996
46	1	H	2.06851	-1.161171	1.333096
47	1	H	4.558606	-0.632251	-1.310404
48	1	H	2.443615	-1.826068	-1.600904
49	1	H	2.310736	-4.413569	-0.437004

50	1	H	3.081333	-4.020463	-1.997004
51	1	H	4.01294	-4.854455	-0.719604
52	1	H	6.000605	-0.536139	1.879496
53	1	H	5.516117	-2.013343	0.992596
54	1	H	6.329206	-0.676237	0.131996

Standard orientation of **5d**-1

Center	Atomic	Atomic	Coordinates (Angstroms)		
Number	Number	Type	X	Y	Z
1	6	C	-3.832496	0.112919	-1.738099
2	6	C	-3.861502	-1.028181	-0.955299
3	6	C	-3.012303	-1.110586	0.173001
4	6	C	-2.124397	-0.092891	0.523901
5	6	C	-2.08319	1.076909	-0.300599
6	6	C	-2.97559	1.167314	-1.418499
7	6	C	-1.280298	-0.247295	1.775001
8	8	O	-0.206385	2.054799	0.805401
9	6	C	-1.186184	2.233904	-0.088299
10	6	C	2.458617	2.389683	-0.524999
11	6	C	2.107919	2.778685	0.944001
12	6	C	0.654122	3.191394	1.163001
13	6	C	0.345324	3.543695	2.613201
14	8	O	-3.042884	2.235715	-2.221299
15	8	O	-4.716608	-2.010576	-1.304299
16	8	O	-1.303578	3.294705	-0.715099
17	17	Cl	-3.160611	-2.597885	1.131301
18	8	O	3.586721	3.128177	-1.009399
19	6	C	2.832509	0.937681	-0.615399
20	6	C	2.028803	-0.032414	-1.050699
21	6	C	0.169199	-0.750304	1.603101
22	8	O	1.036602	-0.326309	2.340901
23	6	C	0.413193	-1.870905	0.599701
24	6	C	2.382095	-1.505016	-1.000399
25	6	C	1.889091	-2.181013	0.301401

26	8	O	3.785594	-1.728024	-1.064099
27	8	O	1.999383	-3.598014	0.201901
28	6	C	3.21068	-4.153321	0.692501
29	6	C	4.336395	-1.556427	-2.359699
30	1	H	-4.487595	0.199723	-2.596599
31	1	H	-1.762702	-0.977093	2.434701
32	1	H	-1.217893	0.688204	2.325801
33	1	H	1.584918	2.583488	-1.166399
34	1	H	2.351515	1.957684	1.627101
35	1	H	2.738724	3.629082	1.233401
36	1	H	0.384726	4.014895	0.498801
37	1	H	-0.704275	3.833701	2.729101
38	1	H	0.968028	4.385092	2.936501
39	1	H	0.550819	2.687794	3.264401
40	1	H	-2.42738	2.920611	-1.849099
41	1	H	-4.636512	-2.745077	-0.670099
42	1	H	3.335026	4.064679	-1.037499
43	1	H	3.824107	0.690276	-0.238299
44	1	H	1.037505	0.215491	-1.430199
45	1	H	-0.166706	-1.689302	-0.311899
46	1	H	-0.023212	-2.777103	1.042001
47	1	H	1.888792	-2.029613	-1.836699
48	1	H	2.504393	-1.805117	1.129301
49	1	H	3.093974	-5.23992	0.640601
50	1	H	3.384982	-3.866322	1.741301
51	1	H	4.074682	-3.846426	0.094901
52	1	H	4.203201	-0.530726	-2.727599
53	1	H	3.883591	-2.254925	-3.080299
54	1	H	5.404994	-1.771833	-2.278299

Standard orientation of **5e**-1

Center	Atomic	Atomic	Coordinates (Angstroms)		
Number	Number	Type	X	Y	Z
1	6	C	4.2487	1.057304	-0.430601

2	6	C	4.502502	-0.304996	-0.438901
3	6	C	3.478403	-1.197397	-0.050001
4	6	C	2.230302	-0.753698	0.375199
5	6	C	1.980001	0.651201	0.395899
6	6	C	2.9996	1.541803	-0.042201
7	6	C	1.145703	-1.7492	0.722599
8	8	O	0.362599	2.3973	0.440799
9	6	C	0.6929	1.1566	0.929499
10	6	C	-2.063002	2.631397	-1.294701
11	6	C	-1.612103	3.635297	-0.222301
12	6	C	-0.852502	3.040398	0.957999
13	6	C	-0.439203	4.073099	2.000299
14	8	O	2.863998	2.883702	-0.089601
15	8	O	5.725002	-0.716694	-0.836101
16	8	O	-0.028799	0.579199	1.722299
17	17	Cl	3.875105	-2.923096	-0.155301
18	8	O	-2.884303	3.400596	-2.185101
19	6	C	-2.824000	1.458496	-0.731301
20	6	C	-2.526499	0.186496	-1.012501
21	6	C	0.044603	-1.827601	-0.345401
22	8	O	0.184703	-1.318501	-1.441001
23	6	C	-1.212796	-2.602102	0.033499
24	6	C	-3.225098	-1.009105	-0.420301
25	6	C	-2.342597	-1.735304	0.642999
26	8	O	-3.587297	-1.861005	-1.501901
27	8	O	-3.141396	-2.634304	1.420599
28	6	C	-3.782596	-2.036205	2.531399
29	6	C	-4.700396	-2.710706	-1.266601
30	1	H	5.017499	1.752705	-0.746001
31	1	H	1.563504	-2.757199	0.814299
32	1	H	0.686303	-1.5115	1.683299
33	1	H	-1.177801	2.252598	-1.826901
34	1	H	-2.511103	4.123396	0.176399

35	1	H	-1.018504	4.420098	-0.707001
36	1	H	-1.438601	2.249697	1.426399
37	1	H	0.192096	4.852799	1.558599
38	1	H	0.111597	3.596899	2.817599
39	1	H	-1.327004	4.555398	2.423699
40	1	H	1.922098	3.100601	0.059899
41	1	H	5.753003	-1.689794	-0.819201
42	1	H	-3.204202	2.791095	-2.869201
43	1	H	-3.662301	1.701395	-0.074201
44	1	H	-1.706399	-0.048003	-1.687401
45	1	H	-0.967095	-3.357802	0.786199
46	1	H	-1.592095	-3.098803	-0.863601
47	1	H	-4.135198	-0.671106	0.102099
48	1	H	-1.912598	-0.970003	1.304799
49	1	H	-3.053697	-1.578304	3.217599
50	1	H	-4.511997	-1.266406	2.236899
51	1	H	-4.314195	-2.834806	3.055499
52	1	H	-4.934595	-3.182707	-2.224901
53	1	H	-4.486095	-3.482006	-0.518901
54	1	H	-5.576996	-2.132607	-0.933301

Standard orientation of **5f**-1

Center	Atomic	Atomic	Coordinates (Angstroms)		
Number	Number	Type	X	Y	Z
1	6	C	4.2487	1.057304	-0.430601
2	6	C	4.502502	-0.304996	-0.438901
3	6	C	3.478403	-1.197397	-0.050001
4	6	C	2.230302	-0.753698	0.375199
5	6	C	1.980001	0.651201	0.395899
6	6	C	2.9996	1.541803	-0.042201
7	6	C	1.145703	-1.7492	0.722599
8	8	O	0.362599	2.3973	0.440799
9	6	C	0.6929	1.1566	0.929499
10	6	C	-2.063002	2.631397	-1.294701

11	6	C	-1.612103	3.635297	-0.222301
12	6	C	-0.852502	3.040398	0.957999
13	6	C	-0.439203	4.073099	2.000299
14	8	O	2.863998	2.883702	-0.089601
15	8	O	5.725002	-0.716694	-0.836101
16	8	O	-0.028799	0.579199	1.722299
17	17	Cl	3.875105	-2.923096	-0.155301
18	8	O	-2.884303	3.400596	-2.185101
19	6	C	-2.824	1.458496	-0.731301
20	6	C	-2.526499	0.186496	-1.012501
21	6	C	0.044603	-1.827601	-0.345401
22	8	O	0.184703	-1.318501	-1.441001
23	6	C	-1.212796	-2.602102	0.033499
24	6	C	-3.225098	-1.009105	-0.420301
25	6	C	-2.342597	-1.735304	0.642999
26	8	O	-3.587297	-1.861005	-1.501901
27	8	O	-3.141396	-2.634304	1.420599
28	6	C	-3.782596	-2.036205	2.531399
29	6	C	-4.700396	-2.710706	-1.266601
30	1	H	5.017499	1.752705	-0.746001
31	1	H	1.563504	-2.757199	0.814299
32	1	H	0.686303	-1.5115	1.683299
33	1	H	-1.177801	2.252598	-1.826901
34	1	H	-2.511103	4.123396	0.176399
35	1	H	-1.018504	4.420098	-0.707001
36	1	H	-1.438601	2.249697	1.426399
37	1	H	0.192096	4.852799	1.558599
38	1	H	0.111597	3.596899	2.817599
39	1	H	-1.327004	4.555398	2.423699
40	1	H	1.922098	3.100601	0.059899
41	1	H	5.753003	-1.689794	-0.819201
42	1	H	-3.204202	2.791095	-2.869201
43	1	H	-3.662301	1.701395	-0.074201

44	1	H	-1.706399	-0.048003	-1.687401
45	1	H	-0.967095	-3.357802	0.786199
46	1	H	-1.592095	-3.098803	-0.863601
47	1	H	-4.135198	-0.671106	0.102099
48	1	H	-1.912598	-0.970003	1.304799
49	1	H	-3.053697	-1.578304	3.217599
50	1	H	-4.511997	-1.266406	2.236899
51	1	H	-4.314195	-2.834806	3.055499
52	1	H	-4.934595	-3.182707	-2.224901
53	1	H	-4.486095	-3.482006	-0.518901
54	1	H	-5.576996	-2.132607	-0.933301

Standard orientation of **5g**-1

Center	Atomic	Atomic	Coordinates (Angstroms)		
Number	Number	Type	X	Y	Z
1	6	C	4.320392	1.118697	0.013693
2	6	C	4.58979	-0.234903	-0.123407
3	6	C	3.531589	-1.163702	-0.004807
4	6	C	2.230289	-0.7661	0.289693
5	6	C	1.970191	0.6216	0.459693
6	6	C	3.022392	1.556599	0.276293
7	6	C	1.105588	-1.774899	0.343293
8	8	O	0.295493	2.302802	0.419793
9	6	C	0.619492	1.055601	0.889093
10	6	C	-2.161107	2.482205	-1.436707
11	6	C	-1.688906	3.482304	-0.347107
12	6	C	-0.967706	2.905303	0.869693
13	6	C	-0.634005	3.952603	1.926993
14	8	O	2.862894	2.894299	0.371393
15	8	O	5.86309	-0.600405	-0.385707
16	8	O	-0.145509	0.409402	1.579693
17	17	Cl	3.958387	-2.865903	-0.264107
18	8	O	-1.122607	2.113804	-2.329707
19	6	C	-2.890108	1.298906	-0.836907

20	6	C	-2.75631	0.042406	-1.266807
21	6	C	0.039088	-1.566498	-0.740007
22	8	O	0.159389	-0.723598	-1.611207
23	6	C	-1.163813	-2.499396	-0.677607
24	6	C	-3.380411	-1.160294	-0.600307
25	6	C	-2.347712	-1.996595	0.202593
26	8	O	-4.412311	-0.801193	0.310593
27	8	O	-2.977314	-3.164694	0.713893
28	6	C	-3.404513	-3.082794	2.065893
29	6	C	-5.67481	-0.615091	-0.306607
30	1	H	5.117892	1.842596	-0.104407
31	1	H	1.492187	-2.7903	0.212793
32	1	H	0.605988	-1.756899	1.315993
33	1	H	-2.863506	3.047706	-2.066407
34	1	H	-2.573205	4.007605	0.040593
35	1	H	-1.059105	4.235803	-0.834807
36	1	H	-1.549707	2.097104	1.313693
37	1	H	-0.095506	3.499302	2.765493
38	1	H	-1.554904	4.399004	2.317893
39	1	H	-0.018904	4.757902	1.508493
40	1	H	1.904094	3.0859	0.368093
41	1	H	5.902688	-1.568305	-0.482507
42	1	H	-0.561808	1.442203	-1.905607
43	1	H	-3.542208	1.503506	0.011493
44	1	H	-2.11141	-0.160295	-2.116307
45	1	H	-1.505113	-2.687396	-1.701107
46	1	H	-0.860814	-3.456097	-0.242007
47	1	H	-3.790812	-1.839593	-1.367007
48	1	H	-1.971211	-1.372895	1.025193
49	1	H	-2.560113	-2.871995	2.740693
50	1	H	-4.171812	-2.314593	2.203493
51	1	H	-3.817415	-4.063893	2.317193
52	1	H	-6.028312	-1.544391	-0.778007

53	1	H	-5.64661	0.178109	-1.066307
54	1	H	-6.37441	-0.32879	0.483193

Standard orientation of **5h**-1

Center	Atomic	Atomic	Coordinates (Angstroms)		
Number	Number	Type	X	Y	Z
1	6	C	4.2487	1.057304	-0.430601
2	6	C	4.502502	-0.304996	-0.438901
3	6	C	3.478403	-1.197397	-0.050001
4	6	C	2.230302	-0.753698	0.375199
5	6	C	1.980001	0.651201	0.395899
6	6	C	2.9996	1.541803	-0.042201
7	6	C	1.145703	-1.7492	0.722599
8	8	O	0.362599	2.3973	0.440799
9	6	C	0.6929	1.1566	0.929499
10	6	C	-2.063002	2.631397	-1.294701
11	6	C	-1.612103	3.635297	-0.222301
12	6	C	-0.852502	3.040398	0.957999
13	6	C	-0.439203	4.073099	2.000299
14	8	O	2.863998	2.883702	-0.089601
15	8	O	5.725002	-0.716694	-0.836101
16	8	O	-0.028799	0.579199	1.722299
17	17	Cl	3.875105	-2.923096	-0.155301
18	8	O	-2.884303	3.400596	-2.185101
19	6	C	-2.824	1.458496	-0.731301
20	6	C	-2.526499	0.186496	-1.012501
21	6	C	0.044603	-1.827601	-0.345401
22	8	O	0.184703	-1.318501	-1.441001
23	6	C	-1.212796	-2.602102	0.033499
24	6	C	-3.225098	-1.009105	-0.420301
25	6	C	-2.342597	-1.735304	0.642999
26	8	O	-3.587297	-1.861005	-1.501901
27	8	O	-3.141396	-2.634304	1.420599
28	6	C	-3.782596	-2.036205	2.531399

29	6	C	-4.700396	-2.710706	-1.266601
30	1	H	5.017499	1.752705	-0.746001
31	1	H	1.563504	-2.757199	0.814299
32	1	H	0.686303	-1.5115	1.683299
33	1	H	-1.177801	2.252598	-1.826901
34	1	H	-2.511103	4.123396	0.176399
35	1	H	-1.018504	4.420098	-0.707001
36	1	H	-1.438601	2.249697	1.426399
37	1	H	0.192096	4.852799	1.558599
38	1	H	0.111597	3.596899	2.817599
39	1	H	-1.327004	4.555398	2.423699
40	1	H	1.922098	3.100601	0.059899
41	1	H	5.753003	-1.689794	-0.819201
42	1	H	-3.204202	2.791095	-2.869201
43	1	H	-3.662301	1.701395	-0.074201
44	1	H	-1.706399	-0.048003	-1.687401
45	1	H	-0.967095	-3.357802	0.786199
46	1	H	-1.592095	-3.098803	-0.863601
47	1	H	-4.135198	-0.671106	0.102099
48	1	H	-1.912598	-0.970003	1.304799
49	1	H	-3.053697	-1.578304	3.217599
50	1	H	-4.511997	-1.266406	2.236899
51	1	H	-4.314195	-2.834806	3.055499
52	1	H	-4.934595	-3.182707	-2.224901
53	1	H	-4.486095	-3.482006	-0.518901
54	1	H	-5.576996	-2.132607	-0.933301

Standard orientation of **6a**-1

Center	Atomic	Atomic	Coordinates (Angstroms)		
Number	Number	Type	X	Y	Z
1	6	C	-3.163505	-1.883683	-1.06509
2	6	C	-3.776399	-0.69708	-0.67479
3	6	C	-3.064095	0.197717	0.139811
4	6	C	-1.759496	-0.068889	0.568711

5	6	C	-1.151602	-1.291092	0.191811
6	6	C	-1.874006	-2.185789	-0.63599
7	6	C	-1.026291	0.966507	1.394211
8	8	O	1.109101	-0.748603	0.719811
9	6	C	0.191496	-1.746498	0.680911
10	6	C	3.741497	-1.436815	-1.02369
11	6	C	3.275195	-1.927413	0.336911
12	6	C	2.446699	-1.015609	1.250911
13	6	C	2.405797	-1.518909	2.696111
14	8	O	-1.268812	-3.332192	-1.03629
15	8	O	-5.035798	-0.469874	-1.10849
16	8	O	0.427891	-2.890899	1.007711
17	17	Cl	-3.911688	1.681721	0.601111
18	6	C	3.912204	-0.051516	-1.53459
19	6	C	3.58051	1.211786	-0.81999
20	6	C	-0.312787	1.999404	0.509211
21	8	O	-0.764185	2.295606	-0.58579
22	6	C	0.944016	2.675298	1.041211
23	6	C	2.360412	1.752391	-0.82949
24	6	C	1.956218	3.005293	-0.09109
25	8	O	1.431223	3.995296	-0.96439
26	8	O	2.8209	-0.877511	-1.96639
27	1	H	-3.714908	-2.56318	-1.70889
28	1	H	-1.738589	1.529711	2.010511
29	1	H	-0.312194	0.506304	2.074111
30	1	H	4.411594	-2.170818	-1.48399
31	1	H	4.188194	-2.186217	0.892611
32	1	H	2.733191	-2.86851	0.194511
33	1	H	2.881504	-0.016411	1.245611
34	1	H	2.061492	-2.551807	2.759811
35	1	H	3.411297	-1.450613	3.127411
36	1	H	1.7419	-0.890906	3.301011
37	1	H	-1.862914	-3.802389	-1.64209

38	1	H	-5.327294	0.401327	-0.78549
39	1	H	4.697604	0.063181	-2.29029
40	1	H	4.410912	1.707482	-0.31359
41	1	H	1.412214	2.062096	1.818111
42	1	H	0.639421	3.6229	1.509011
43	1	H	1.57241	1.235595	-1.37189
44	1	H	2.84092	3.455589	0.374411
45	1	H	0.574221	3.6422	-1.26969

Standard orientation of **6b**-1

Center	Atomic	Atomic	Coordinates (Angstroms)		
Number	Number	Type	X	Y	Z
1	6	C	-4.137808	-1.311277	0.1369
2	6	C	-4.200004	0.067823	-0.0057
3	6	C	-3.024802	0.79472	-0.295
4	6	C	-1.787004	0.163116	-0.3909
5	6	C	-1.715308	-1.240884	-0.1671
6	6	C	-2.91291	-1.97738	0.0373
7	6	C	-0.602101	0.961113	-0.8941
8	8	O	0.565292	-1.237291	0.3885
9	6	C	-0.45131	-1.995788	-0.0271
10	6	C	4.115691	-1.615001	-0.503
11	6	C	2.66689	-2.058197	-0.6204
12	6	C	1.892691	-1.774995	0.6847
13	6	C	1.876887	-2.921995	1.6901
14	8	O	-2.938314	-3.31388	0.1671
15	8	O	-5.362002	0.756127	0.086
16	8	O	-0.363513	-3.214488	-0.1998
17	17	Cl	-3.187297	2.52402	-0.5573
18	6	C	4.483195	-0.176302	-0.4455
19	6	C	3.470199	0.905601	-0.5334
20	6	C	0.200301	1.77591	0.1177
21	8	O	0.103101	1.619611	1.3224
22	6	C	1.128604	2.827408	-0.4897

23	6	C	3.427302	1.953901	0.2964
24	6	C	2.421805	3.090604	0.3092
25	8	O	2.134606	3.491605	1.6413
26	8	O	4.709993	-0.966003	-1.6308
27	1	H	-5.03311	-1.898274	0.3213
28	1	H	-0.944099	1.653414	-1.6724
29	1	H	0.121897	0.301411	-1.3829
30	1	H	4.787389	-2.311903	0.0042
31	1	H	2.209492	-1.494695	-1.4382
32	1	H	2.603687	-3.117797	-0.8903
33	1	H	2.340693	-0.902596	1.1642
34	1	H	1.476985	-3.840393	1.2593
35	1	H	1.283688	-2.652693	2.57
36	1	H	2.905287	-3.104898	2.0246
37	1	H	-2.020715	-3.642483	-0.0032
38	1	H	-6.081904	0.134929	0.2805
39	1	H	5.394096	0.079795	0.0993
40	1	H	2.726798	0.783403	-1.318
41	1	H	1.352804	2.586007	-1.5349
42	1	H	0.551007	3.762509	-0.5069
43	1	H	4.194202	2.063099	1.0642
44	1	H	2.913008	3.961202	-0.1488
45	1	H	1.495604	2.835907	1.9821

Standard orientation of **6c**-1

Center Number	Atomic Number	Atomic Type	Coordinates (Angstroms)		
			X	Y	Z
1	6	C	-2.804575	-2.446842	-0.722393
2	6	C	-3.582789	-1.328453	-0.433793
3	6	C	-2.992704	-0.232645	0.214608
4	6	C	-1.640004	-0.242127	0.567608
5	6	C	-0.847589	-1.380516	0.268008
6	6	C	-1.454574	-2.481824	-0.371693
7	6	C	-1.04012	0.985381	1.211008

8	8	O	1.38642	-2.025787	-0.191493
9	6	C	0.573913	-1.484597	0.735708
10	6	C	3.732996	-0.202955	0.333308
11	6	C	3.811714	-1.607454	-0.226493
12	6	C	2.686527	-2.531669	0.245508
13	6	C	2.806445	-3.940568	-0.324093
14	8	O	-0.719059	-3.607015	-0.598693
15	8	O	-4.89929	-1.25807	-0.746993
16	8	O	0.952908	-1.134892	1.841208
17	17	Cl	-4.022422	1.137842	0.582608
18	6	C	3.42358	0.96154	-0.521693
19	6	C	2.715165	2.144831	0.004608
20	6	C	-0.808335	2.148084	0.248608
21	8	O	-1.159235	2.11758	-0.920093
22	6	C	-0.077452	3.362894	0.811808
23	6	C	1.848855	2.84432	-0.734893
24	6	C	0.917041	3.936407	-0.232593
25	8	O	0.223033	4.557098	-1.300793
26	8	O	4.769484	0.702058	-0.064892
27	1	H	-3.260063	-3.308148	-1.209493
28	1	H	-0.088417	0.745594	1.689008
29	1	H	-1.689125	1.359073	2.014608
30	1	H	3.466795	-0.136959	1.390008
31	1	H	3.836514	-1.573954	-1.322693
32	1	H	4.76042	-2.057542	0.097708
33	1	H	2.671127	-2.55747	1.338408
34	1	H	3.751551	-4.395955	-0.008692
35	1	H	1.980254	-4.564679	0.027108
36	1	H	2.779745	-3.919568	-1.418693
37	1	H	-1.29515	-4.282722	-0.988693
38	1	H	-5.165079	-2.074074	-1.199093
39	1	H	3.275583	0.768338	-1.586293
40	1	H	2.882761	2.376633	1.057008

41	1	H	-0.819862	4.137484	1.048208
42	1	H	0.437852	3.104401	1.743008
43	1	H	1.721059	2.591418	-1.786993
44	1	H	1.49543	4.732515	0.251208
45	1	H	-0.402858	3.88169	-1.624793

Standard orientation of **6d**-1

Center	Atomic	Atomic	Coordinates (Angstroms)		
Number	Number	Type	X	Y	Z
1	6	C	-3.747894	0.891859	-0.399001
2	6	C	-3.735417	-0.495341	-0.527701
3	6	C	-2.630528	-1.21046	-0.039001
4	6	C	-1.533018	-0.573878	0.549499
5	6	C	-1.531995	0.838822	0.631099
6	6	C	-2.671983	1.544141	0.193999
7	6	C	-0.393232	-1.399797	1.096199
8	8	O	0.180835	2.629494	0.829499
9	6	C	-0.466082	1.582905	1.399699
10	6	C	2.913424	1.962249	0.053399
11	6	C	2.064742	3.043263	-0.572901
12	6	C	0.561036	2.716388	-0.575301
13	6	C	-0.231046	3.810901	-1.282401
14	8	O	-2.704761	2.891342	0.415899
15	8	O	-4.809126	-1.082024	-1.101101
16	8	O	-0.209087	1.3068	2.552399
17	17	Cl	-2.718657	-2.972458	-0.146301
18	6	C	3.520406	0.897539	-0.774901
19	6	C	3.648984	-0.473963	-0.242401
20	6	C	0.491957	-2.073811	0.041299
21	8	O	0.284459	-1.974108	-1.152101
22	6	C	1.669144	-2.86223	0.604899
23	6	C	3.144867	-1.516455	-0.910801
24	6	C	2.911644	-2.885451	-0.311601
25	8	O	3.977137	-3.318369	0.534099

26	8	O	4.319924	1.961726	-0.231701
27	1	H	-4.622385	1.437973	-0.741801
28	1	H	-0.780945	-2.19119	1.753099
29	1	H	0.246179	-0.788907	1.738199
30	1	H	2.67702	1.725553	1.091799
31	1	H	2.395844	3.207958	-1.605201
32	1	H	2.207057	3.987561	-0.031601
33	1	H	0.37872	1.742991	-1.046901
34	1	H	-0.09083	4.767998	-0.768501
35	1	H	0.126856	3.916395	-2.312701
36	1	H	-1.296649	3.585818	-1.300701
37	1	H	-3.577155	3.233156	0.164499
38	1	H	-4.666742	-2.045126	-1.123001
39	1	H	3.364307	0.966442	-1.854201
40	1	H	4.039782	-0.58477	0.768199
41	1	H	1.979851	-2.432136	1.563899
42	1	H	1.334727	-3.887725	0.818199
43	1	H	2.731169	-1.361748	-1.905101
44	1	H	2.747132	-3.607748	-1.124001
45	1	H	4.798138	-3.239682	0.022499

Standard orientation of **6e**-1

Center Number	Atomic Number	Atomic Type	Coordinates (Angstroms)		
			X	Y	Z
1	6	C	-3.111692	-1.787104	-1.259288
2	6	C	-3.629792	-0.681904	-0.605888
3	6	C	-2.838192	-0.002704	0.343813
4	6	C	-1.525492	-0.391104	0.623713
5	6	C	-0.980092	-1.519405	-0.069388
6	6	C	-1.815192	-2.231604	-0.986188
7	6	C	-0.762491	0.362795	1.693213
8	8	O	1.290608	-1.240606	0.668913
9	6	C	0.394108	-2.052005	0.087912
10	6	C	4.122208	-0.192107	-0.582888

11	6	C	3.610908	-0.565106	0.811613
12	6	C	2.654408	-1.764506	0.857213
13	6	C	2.695207	-2.508606	2.187613
14	8	O	-1.431093	-3.333004	-1.642488
15	8	O	-4.885692	-0.229203	-0.827688
16	8	O	0.720607	-3.173005	-0.316488
17	17	Cl	-3.604491	1.346396	1.177113
18	6	C	3.457609	0.803694	-1.460388
19	6	C	2.202009	1.442894	-1.025788
20	6	C	0.029009	1.626495	1.287413
21	8	O	0.901209	2.026495	2.035413
22	6	C	-0.354191	2.383295	0.022012
23	6	C	1.92881	2.747594	-1.071588
24	6	C	0.70261	3.384595	-0.471588
25	8	O	0.15451	4.242795	-1.480488
26	8	O	3.375308	-0.613506	-1.729288
27	1	H	-3.702892	-2.345903	-1.979088
28	1	H	-0.050492	-0.281605	2.203413
29	1	H	-1.474191	0.709096	2.451513
30	1	H	5.200408	-0.307407	-0.715288
31	1	H	3.128509	0.290894	1.297713
32	1	H	4.483108	-0.815407	1.426113
33	1	H	2.846908	-2.448106	0.030412
34	1	H	3.686607	-2.950406	2.336713
35	1	H	2.491508	-1.830206	3.023613
36	1	H	1.959707	-3.318006	2.198713
37	1	H	-0.531093	-3.576905	-1.300388
38	1	H	-5.309592	-0.793503	-1.494188
39	1	H	4.083409	1.359293	-2.160988
40	1	H	1.500309	0.748294	-0.579088
41	1	H	-0.627591	1.691495	-0.780988
42	1	H	-1.27759	2.932296	0.260013
43	1	H	2.62681	3.450194	-1.526188

44	1	H	1.03331	3.984495	0.390813
45	1	H	-0.47299	4.841695	-1.045988

Standard orientation of **6f**-1

Center Number	Atomic Number	Atomic Type	Coordinates (Angstroms)		
			X	Y	Z
1	6	C	-3.137999	-2.227307	-0.600901
2	6	C	-3.9066	-1.075508	-0.550501
3	6	C	-3.358801	0.099393	0.018899
4	6	C	-2.065101	0.144094	0.532299
5	6	C	-1.2646	-1.042306	0.444099
6	6	C	-1.829599	-2.225506	-0.117001
7	6	C	-1.574702	1.395394	1.238999
8	8	O	0.873401	-2.120704	0.697399
9	6	C	0.1556	-0.953604	0.826899
10	6	C	4.4699	-0.822001	0.295899
11	6	C	3.245901	-1.331102	1.062999
12	6	C	2.235101	-2.006803	0.123099
13	6	C	2.626602	-3.420702	-0.273601
14	8	O	-1.174598	-3.395205	-0.219701
15	8	O	-5.158	-1.134309	-1.046101
16	8	O	0.704399	0.071396	1.186599
17	17	Cl	-4.434502	1.509292	0.044499
18	6	C	4.551799	0.515999	-0.354201
19	6	C	3.489098	1.557898	-0.367701
20	6	C	-0.399502	2.213295	0.673399
21	8	O	0.164897	2.982096	1.437799
22	6	C	-0.095702	2.211195	-0.818501
23	6	C	2.361498	1.504597	-1.079601
24	6	C	1.353197	2.630797	-1.212501
25	8	O	1.750896	3.828597	-0.580601
26	8	O	4.3669	-0.676801	-1.133101
27	1	H	-3.550498	-3.134407	-1.026301
28	1	H	-1.273101	1.148494	2.260299

29	1	H	-2.404302	2.103194	1.327099
30	1	H	5.421101	-1.2625	0.602199
31	1	H	3.574201	-2.046602	1.826199
32	1	H	2.7595	-0.499902	1.569899
33	1	H	2.125601	-1.392303	-0.770401
34	1	H	2.694003	-4.070502	0.605999
35	1	H	1.904803	-3.850803	-0.976001
36	1	H	3.604102	-3.397702	-0.765401
37	1	H	-0.269898	-3.268905	0.140399
38	1	H	-5.5863	-0.266009	-0.939501
39	1	H	5.562399	0.9269	-0.440201
40	1	H	3.711498	2.460098	0.198999
41	1	H	-0.352501	1.244995	-1.263601
42	1	H	-0.790203	2.946095	-1.253801
43	1	H	2.160899	0.628297	-1.695501
44	1	H	1.308597	2.859197	-2.288801
45	1	H	1.426896	3.766897	0.341099

Standard orientation of **6g**-1

Center	Atomic	Atomic	Coordinates (Angstroms)		
Number	Number	Type	X	Y	Z
1	6	C	3.611901	-1.752001	-0.4112
2	6	C	3.906099	-0.434101	-0.1065
3	6	C	2.847998	0.446198	0.208601
4	6	C	1.517498	0.036595	0.270701
5	6	C	1.220801	-1.333105	-0.005399
6	6	C	2.291002	-2.201603	-0.3979
7	6	C	0.465097	1.099494	0.512801
8	8	O	-1.004499	-1.361409	0.894201
9	6	C	-0.108398	-1.976207	0.105301
10	6	C	-3.1779	-0.862112	-0.875
11	6	C	-3.208198	-2.126612	-0.045799
12	6	C	-2.299298	-1.998511	1.190401
13	6	C	-2.125796	-3.29211	1.979001

14	8	O	2.112804	-3.484204	-0.7479
15	8	O	5.198399	-0.046699	-0.1467
16	8	O	-0.375997	-3.055108	-0.4351
17	17	Cl	3.306295	2.141398	0.473101
18	6	C	-3.775402	0.394187	-0.3749
19	6	C	-3.032704	1.641788	-0.6435
20	6	C	0.006096	1.754493	-0.8004
21	8	O	0.024397	1.137093	-1.8486
22	6	C	-0.489307	3.204892	-0.775
23	6	C	-2.672606	2.480889	0.333301
24	6	C	-1.595607	3.54439	0.243101
25	8	O	-0.949307	3.698291	1.513301
26	8	O	-4.383201	-0.339214	-1.4509
27	1	H	4.405002	-2.4365	-0.6873
28	1	H	-0.430103	0.670592	0.964901
29	1	H	0.820795	1.865094	1.203101
30	1	H	-2.3122	-0.757711	-1.53
31	1	H	-4.230698	-2.295114	0.314601
32	1	H	-2.917097	-2.990412	-0.6495
33	1	H	-2.735199	-1.241311	1.848801
34	1	H	-1.713695	-4.08971	1.359001
35	1	H	-1.465596	-3.129609	2.837201
36	1	H	-3.102896	-3.611712	2.359101
37	1	H	1.136804	-3.643105	-0.7916
38	1	H	5.250397	0.904301	0.055801
39	1	H	-4.336102	0.356686	0.562101
40	1	H	-2.624404	1.731689	-1.6476
41	1	H	-0.833307	3.429092	-1.7888
42	1	H	0.362192	3.862394	-0.5568
43	1	H	-3.100305	2.345088	1.329201
44	1	H	-2.029309	4.51239	-0.058799
45	1	H	-1.619708	3.96469	2.161801

Standard orientation of **6h**-1

Center Number	Atomic Number	Atomic Type	Coordinates (Angstroms)		
			X	Y	Z
1	6	C	3.714299	0.92973	0.477197
2	6	C	3.796908	-0.266969	-0.235103
3	6	C	2.614213	-0.955978	-0.546803
4	6	C	1.35681	-0.485188	-0.152803
5	6	C	1.2875	0.728211	0.557097
6	6	C	2.472595	1.413121	0.875597
7	6	C	0.104016	-1.238098	-0.520903
8	8	O	-0.648612	2.281397	0.721497
9	6	C	-0.005903	1.187201	1.191197
10	6	C	-2.650207	1.721881	-1.369303
11	6	C	-1.888817	3.016987	-1.180403
12	6	C	-0.472116	2.834698	-0.610303
13	6	C	0.278474	4.158604	-0.513003
14	8	O	2.347786	2.56562	1.599997
15	8	O	5.027111	-0.69566	-0.598403
16	8	O	-0.473499	0.607698	2.145697
17	17	Cl	2.778925	-2.459377	-1.461603
18	6	C	-3.359102	1.066876	-0.245603
19	6	C	-3.475491	-0.410025	-0.191503
20	6	C	-0.278775	-2.410701	0.400997
21	8	O	0.400127	-2.759695	1.341597
22	6	C	-1.57827	-3.121811	0.023497
23	6	C	-2.944785	-1.128321	0.805497
24	6	C	-2.829874	-2.63562	0.801097
25	8	O	-3.924769	-3.275329	0.147997
26	8	O	-4.078608	1.76977	-1.275403
27	1	H	4.633895	1.454337	0.720597
28	1	H	-0.76039	-0.568004	-0.555603
29	1	H	0.194219	-1.650897	-1.533703
30	1	H	-2.289402	1.077884	-2.176603
31	1	H	-1.812421	3.542788	-2.141403

32	1	H	-2.457622	3.663683	-0.502603
33	1	H	0.09969	2.119802	-1.214203
34	1	H	-0.288231	4.869799	0.097597
35	1	H	1.257775	4.015811	-0.052003
36	1	H	0.413871	4.590405	-1.510903
37	1	H	3.228984	2.887026	1.847197
38	1	H	4.939318	-1.547961	-1.061003
39	1	H	-3.325006	1.583176	0.713497
40	1	H	-3.928187	-0.898829	-1.053503
41	1	H	-1.798971	-2.974312	-1.039803
42	1	H	-1.449962	-4.19241	0.209997
43	1	H	-2.466489	-0.616517	1.640097
44	1	H	-2.740471	-2.98772	1.839897
45	1	H	-4.737471	-2.936635	0.557197

Standard orientation of **7a**-1

Center	Atomic	Atomic	Coordinates (Angstroms)		
Number	Number	Type	X	Y	Z
1	6	C	3.839794	1.39816	-0.08024
2	6	C	4.158725	0.053389	-0.165251
3	6	C	3.176165	-0.932818	-0.000709
4	6	C	1.83722	-0.579976	0.281274
5	6	C	1.512156	0.806603	0.352409
6	6	C	2.523073	1.772604	0.163017
7	6	C	0.775964	-1.654311	0.458835
8	8	O	-0.180881	2.395374	0.046015
9	6	C	0.127599	1.245039	0.70163
10	6	C	-0.204394	-1.746502	-0.699249
11	6	C	-1.364819	-2.703225	-0.526422
12	8	O	-0.093874	-1.06472	-1.719457
13	6	C	-2.586228	-1.941168	-0.010384
14	6	C	-3.563808	-1.619507	-1.152846
15	6	C	-4.58521	-0.542085	-0.757767
16	6	C	-4.084587	0.878238	-0.638399

17	6	C	-2.870982	1.344903	-0.976943
18	6	C	-2.442994	2.779102	-0.827691
19	6	C	-1.46684	2.993829	0.341325
20	6	C	-1.205947	4.485967	0.535654
21	8	O	2.311247	3.125015	0.218637
22	8	O	5.468843	-0.25733	-0.415364
23	17	Cl	3.684701	-2.585761	-0.156153
24	8	O	-0.596327	0.647143	1.480168
25	8	O	-3.280888	-2.767778	0.937153
26	6	C	-2.92449	-2.469932	2.282651
27	1	H	4.610682	2.152787	-0.210255
28	1	H	0.232219	-1.491923	1.392914
29	1	H	1.216567	-2.64836	0.572483
30	1	H	-1.556657	-3.190744	-1.48949
31	1	H	-1.059805	-3.500569	0.160518
32	1	H	-2.279909	-1.011524	0.478352
33	1	H	-4.120588	-2.529305	-1.413095
34	1	H	-3.028398	-1.305273	-2.056009
35	1	H	-5.050257	-0.819782	0.196483
36	1	H	-5.389962	-0.540092	-1.503349
37	1	H	-4.814817	1.574536	-0.22685
38	1	H	-2.121414	0.685293	-1.406636
39	1	H	-1.971781	3.084475	-1.77043
40	1	H	-3.326106	3.415465	-0.690111
41	1	H	-1.890271	2.598228	1.272624
42	1	H	-0.492079	4.645119	1.351061
43	1	H	-0.75982	4.92637	-0.363343
44	1	H	-2.128535	5.02556	0.770009
45	1	H	1.359644	3.292697	0.078714
46	1	H	5.535775	-1.229311	-0.463713
47	1	H	-3.257787	-1.461133	2.544765
48	1	H	-1.844522	-2.561561	2.429619
49	1	H	-3.429439	-3.184505	2.937774

Standard orientation of **7a**-2

Center	Atomic	Atomic	Coordinates (Angstroms)		
Number	Number	Type	X	Y	Z
1	6	C	-3.667953	1.512406	-0.316597
2	6	C	-4.052125	0.184122	-0.28203
3	6	C	-3.139655	-0.816599	0.072967
4	6	C	-1.807414	-0.493955	0.426544
5	6	C	-1.405118	0.873012	0.363397
6	6	C	-2.353993	1.852487	-0.009381
7	6	C	-0.831912	-1.608436	0.764236
8	8	O	0.345747	2.412358	0.080959
9	6	C	-0.017107	1.288993	0.754852
10	6	C	0.010727	-2.035188	-0.425222
11	6	C	1.201508	-2.917694	-0.128026
12	8	O	-0.235143	-1.671148	-1.57624
13	6	C	2.449225	-2.043175	-0.010503
14	6	C	3.149617	-1.901633	-1.373249
15	6	C	4.121815	-0.718159	-1.399727
16	6	C	3.394216	0.596598	-1.498012
17	6	C	3.345776	1.502851	-0.510014
18	6	C	2.653793	2.834176	-0.619644
19	6	C	1.589312	3.049024	0.46882
20	6	C	1.271737	4.537324	0.605534
21	8	O	-2.087287	3.194605	-0.089472
22	8	O	-5.350582	-0.096087	-0.613635
23	17	Cl	-3.71724	-2.455146	0.050765
24	8	O	0.663745	0.716499	1.588255
25	8	O	3.380472	-2.6557	0.894394
26	6	C	3.174918	-2.261838	2.246695
27	1	H	-4.383811	2.281246	-0.594588
28	1	H	-0.184007	-1.328835	1.595883
29	1	H	-1.3496	-2.499148	1.133534
30	1	H	1.295962	-3.668091	-0.921326

31	1	H	1.001737	-3.47892	0.791213
32	1	H	2.181718	-1.050653	0.366053
33	1	H	3.718015	-2.816303	-1.584744
34	1	H	2.416051	-1.780297	-2.179229
35	1	H	4.780967	-0.743753	-0.523972
36	1	H	4.769862	-0.806833	-2.280044
37	1	H	2.894899	0.799389	-2.444292
38	1	H	3.851825	1.294386	0.431439
39	1	H	2.197683	2.94592	-1.611271
40	1	H	3.432554	3.6018	-0.529825
41	1	H	1.941663	2.687935	1.442713
42	1	H	2.155178	5.107242	0.908847
43	1	H	0.895695	4.947129	-0.338601
44	1	H	0.485174	4.69202	1.352118
45	1	H	-1.120897	3.310377	-0.160647
46	1	H	-5.456021	-1.065573	-0.585985
47	1	H	3.892331	-2.799837	2.872102
48	1	H	2.164297	-2.513783	2.58008
49	1	H	3.351983	-1.188054	2.359635
